# Supplementary figures and images for: Chaetoglobosin A induces apoptosis in T-24 human bladder cancer cells through oxidative stress and MAPK/PI3K-AKT-mTOR pathway (part 1 of 2)
Source: PeerJ. 2025 Mar 31;13:e19085. doi: 10.7717/peerj.19085 (PMC11967413; doi:10.7717/peerj.19085)

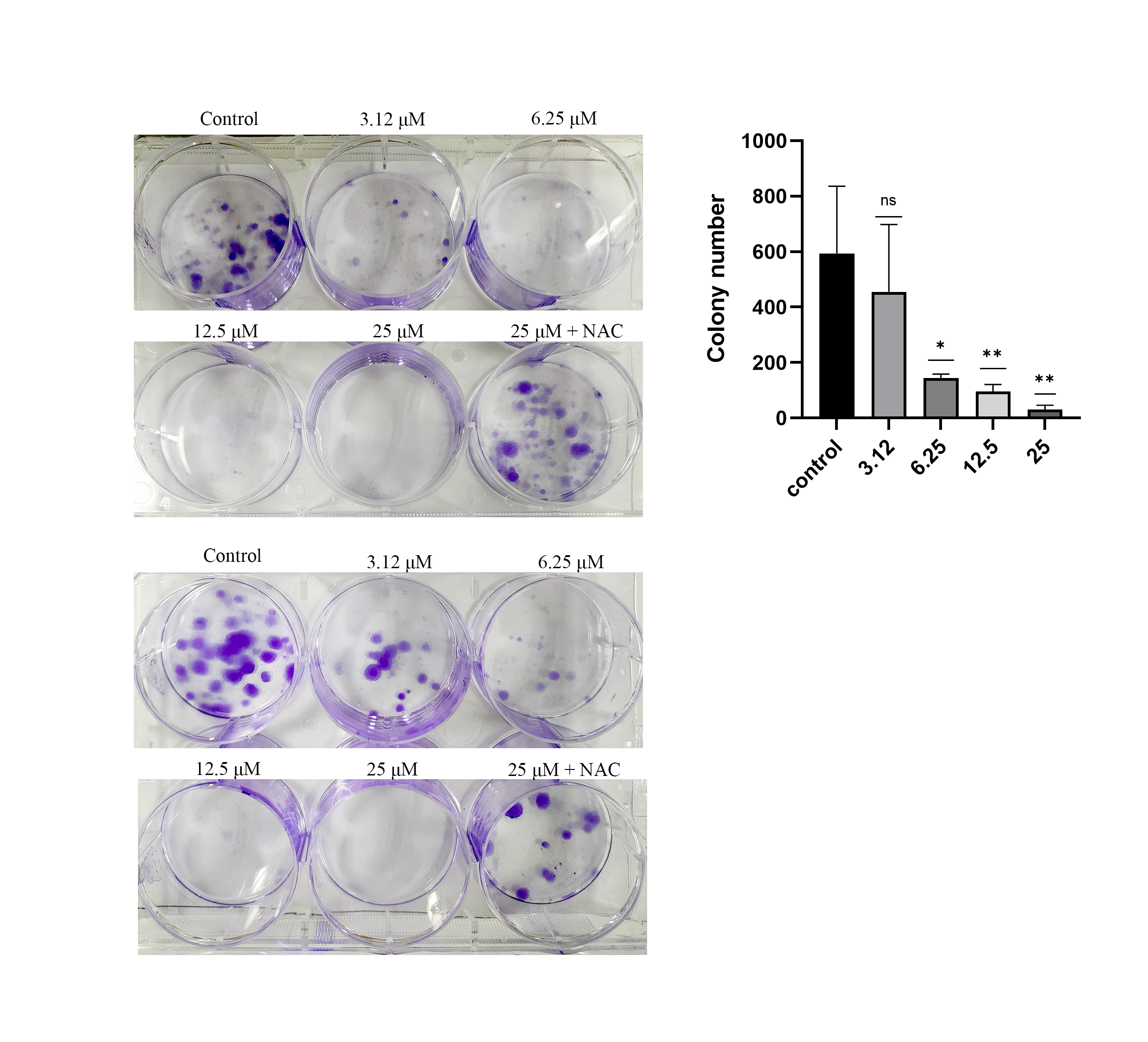

Supplement: Supplemental Information 1 [file peerj-13-19085-s001.png]

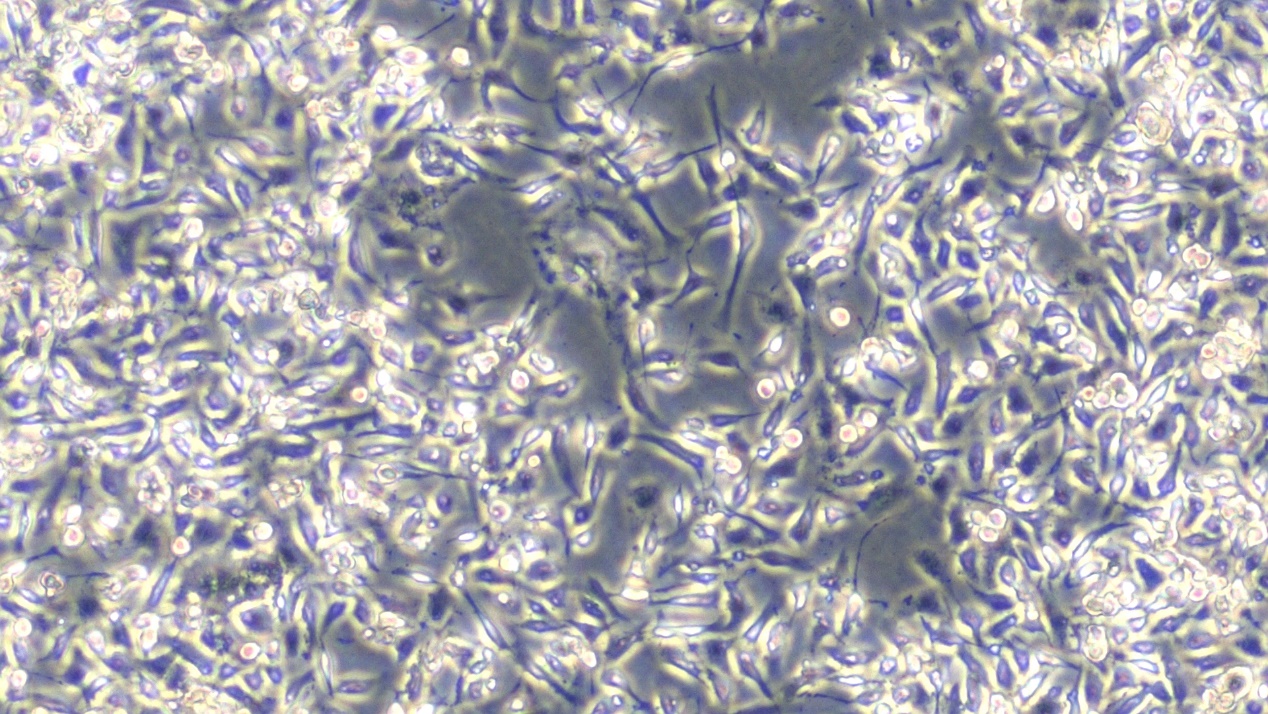

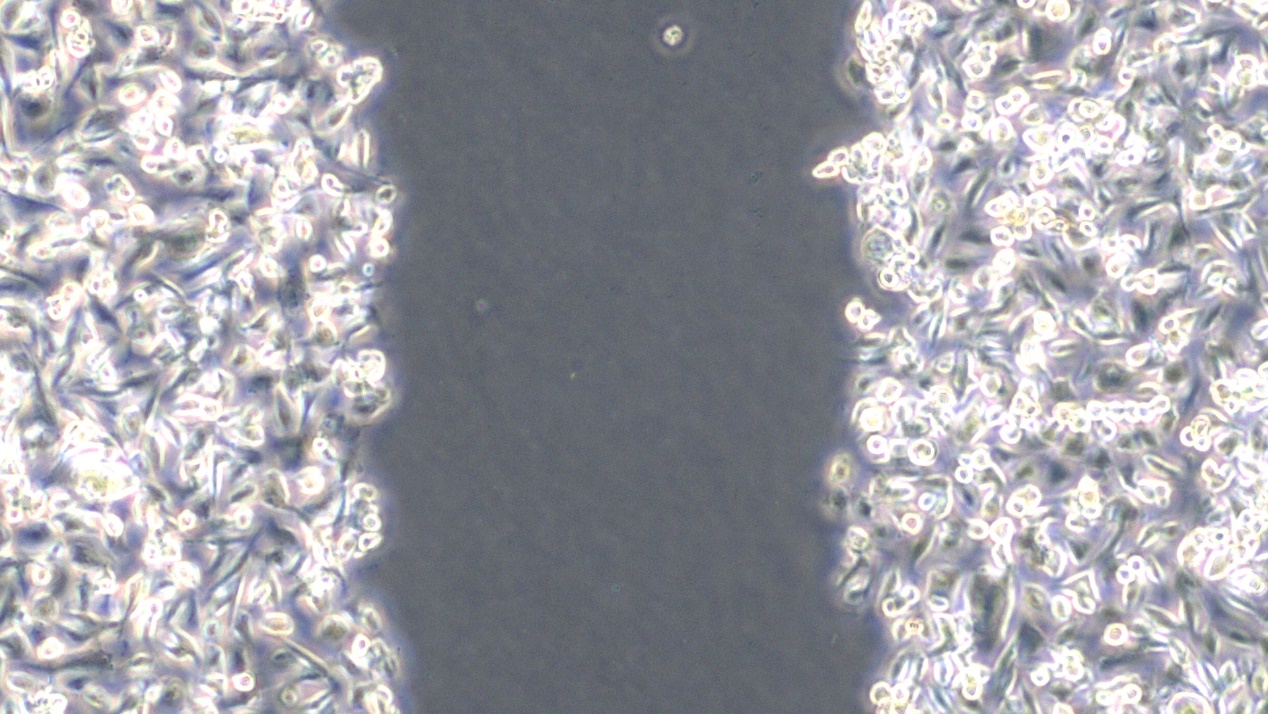

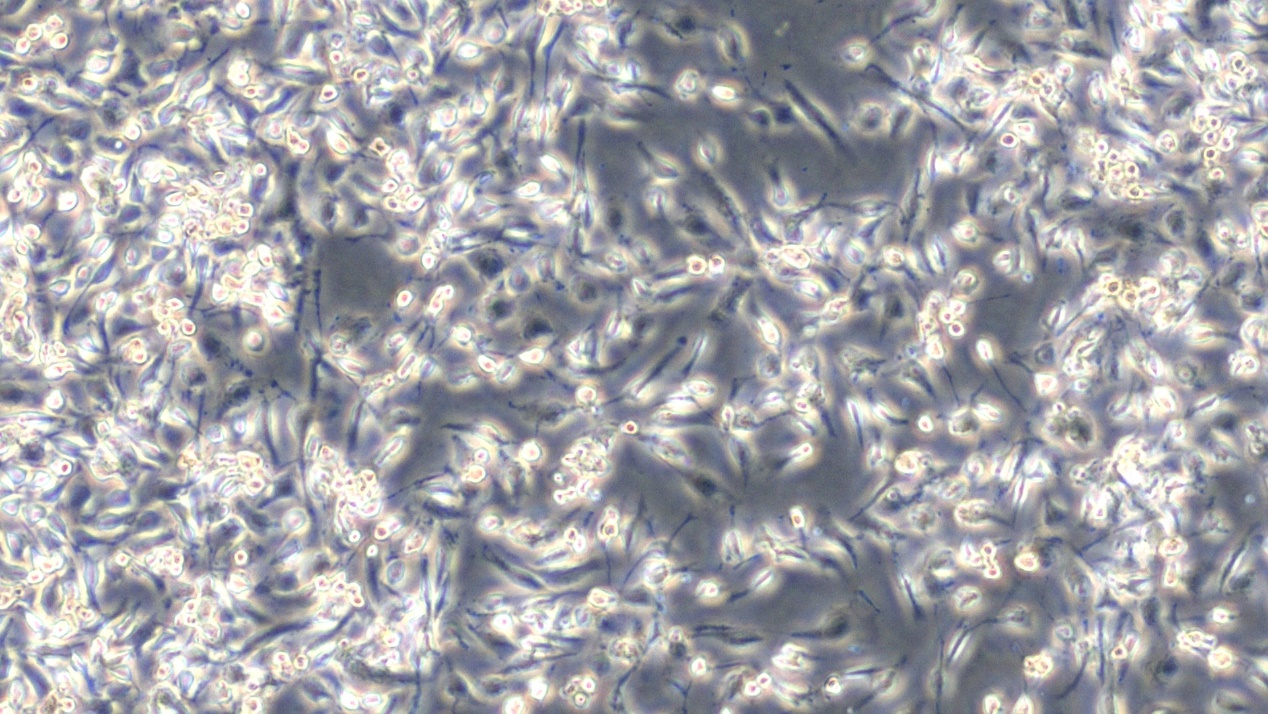

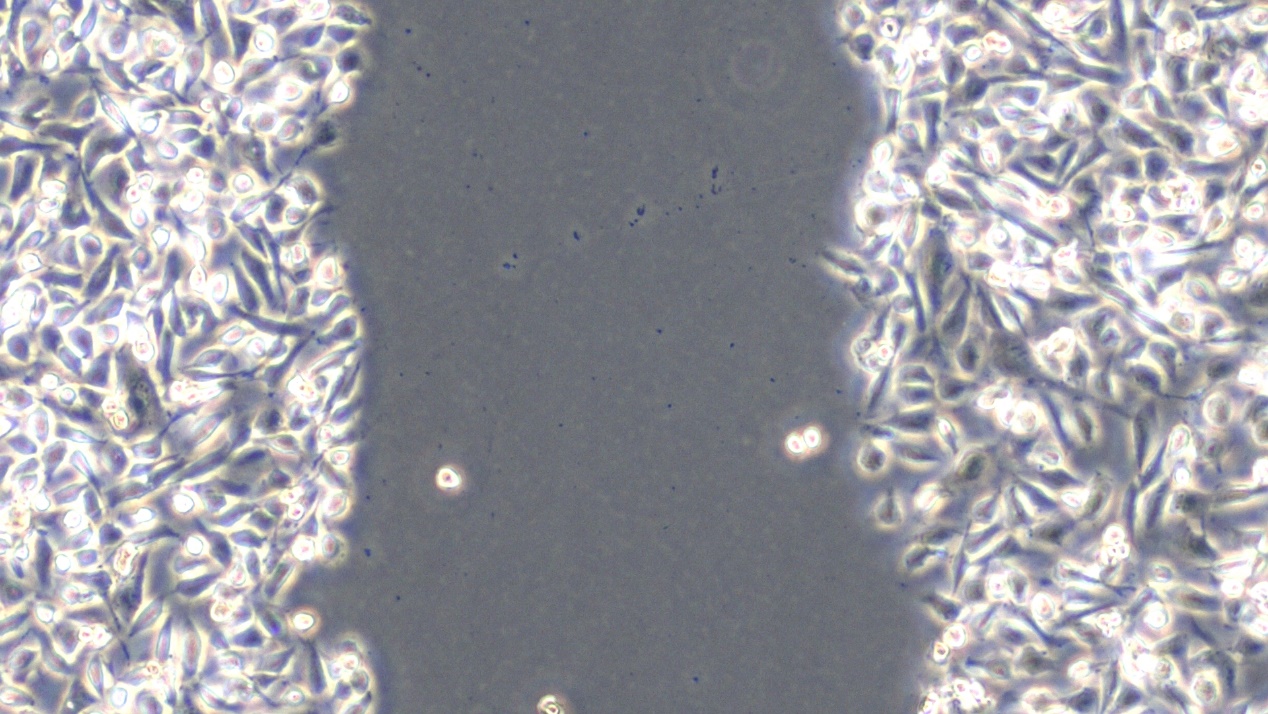

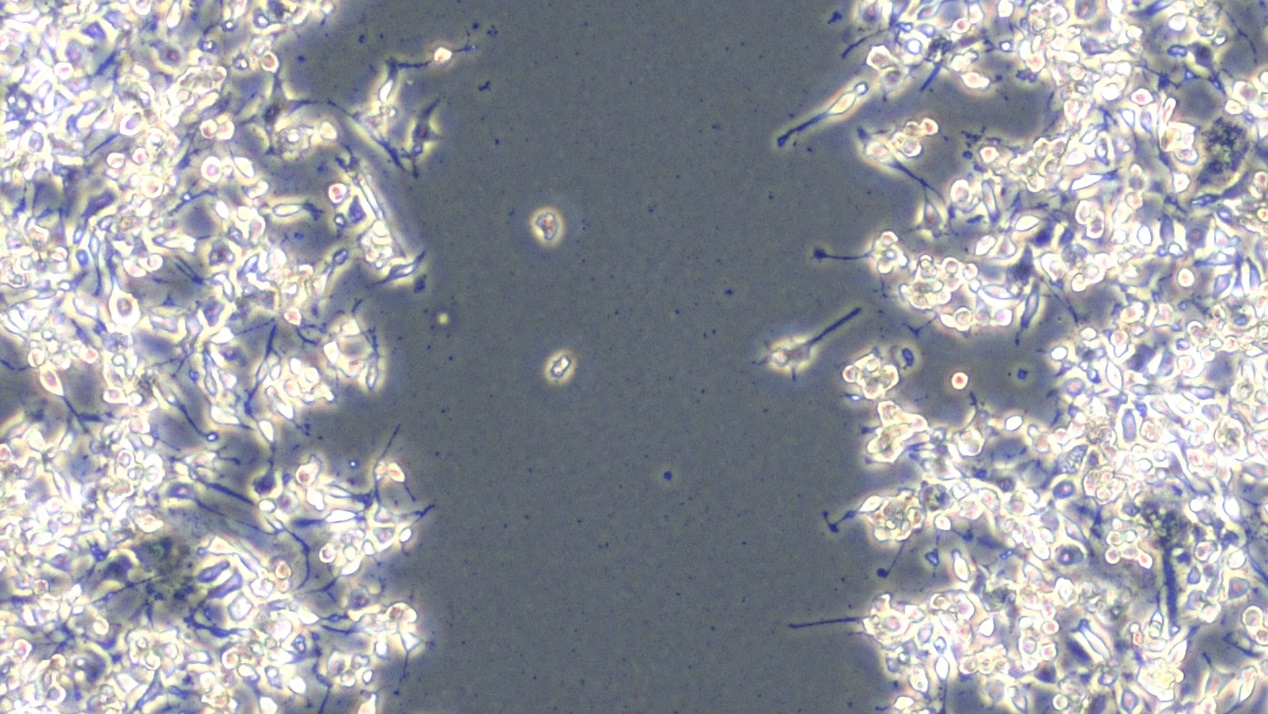

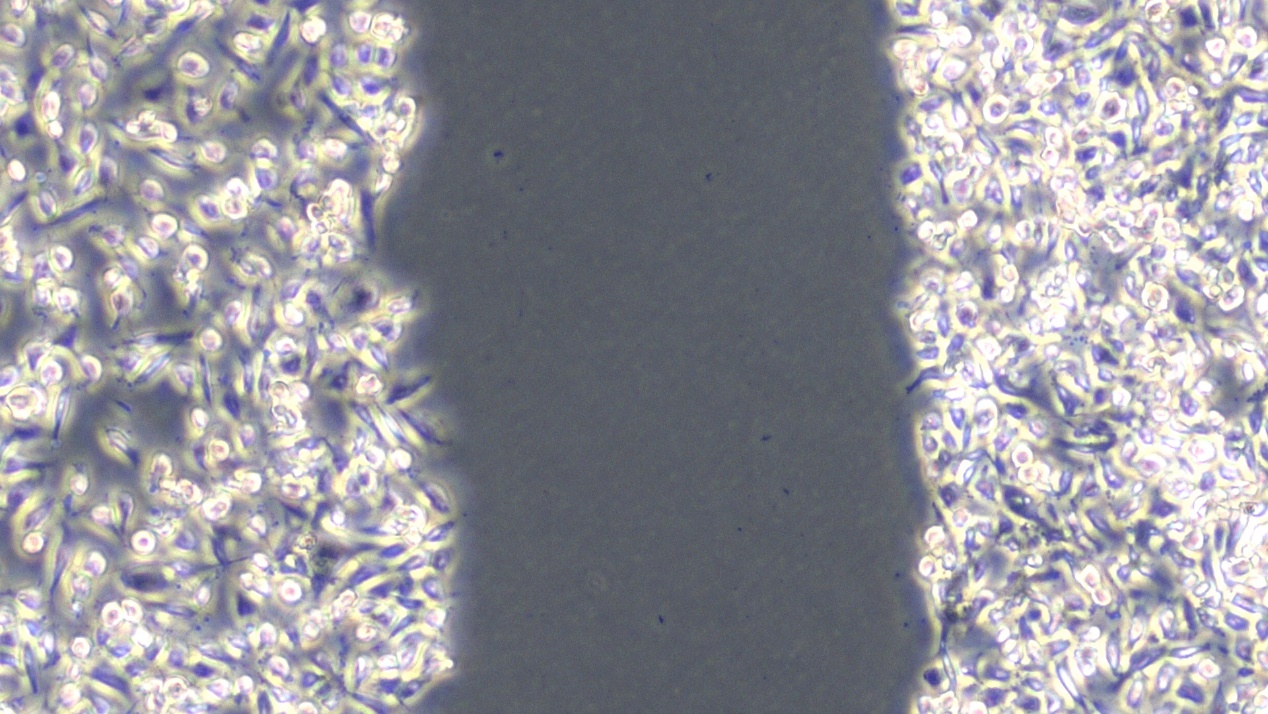

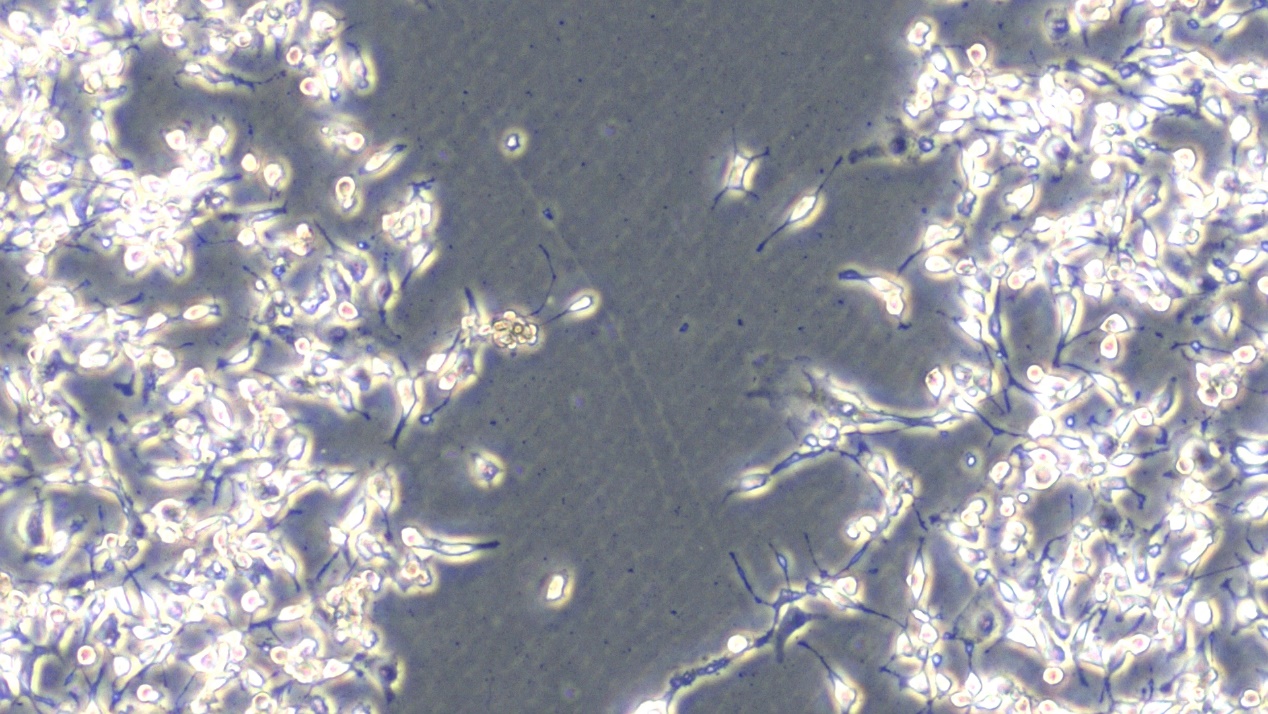

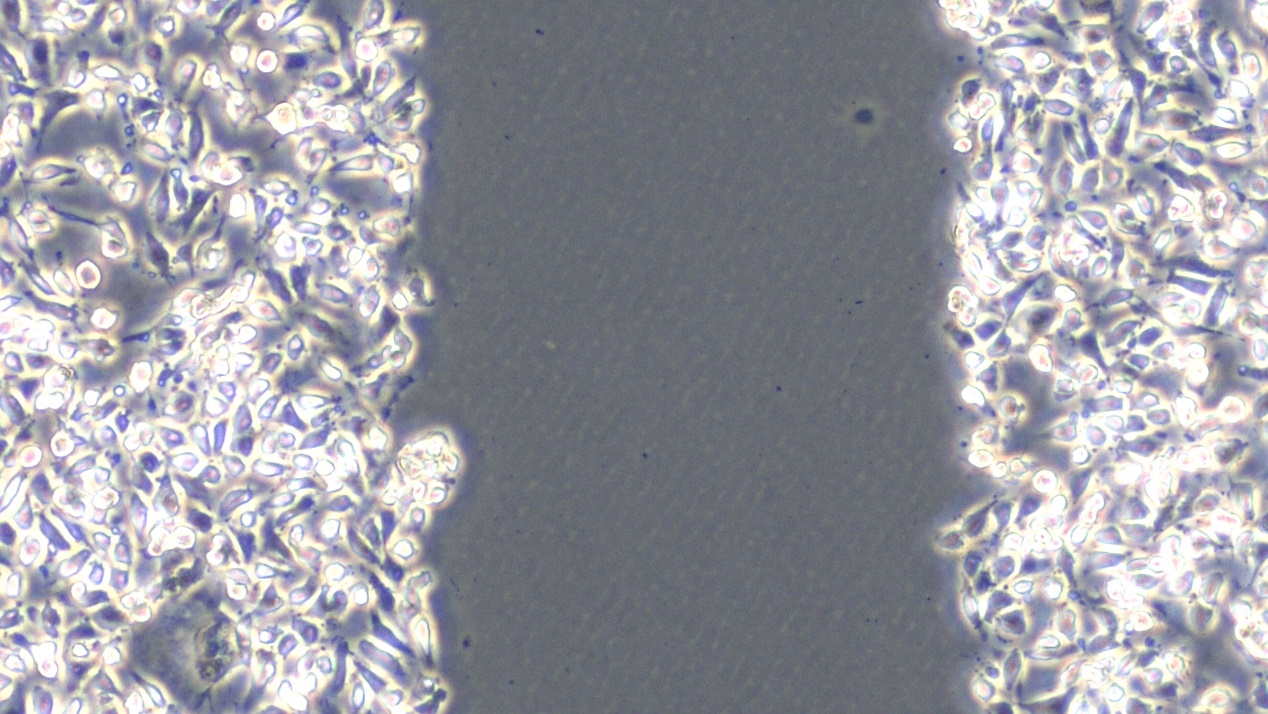

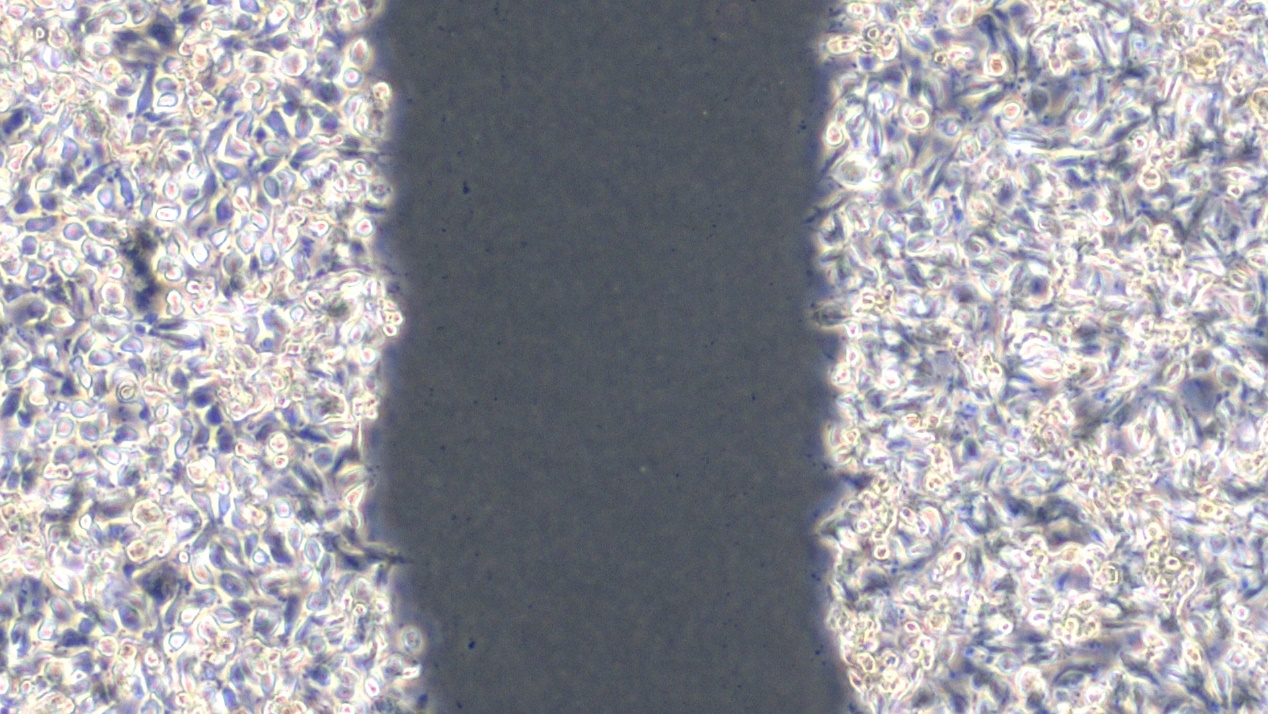

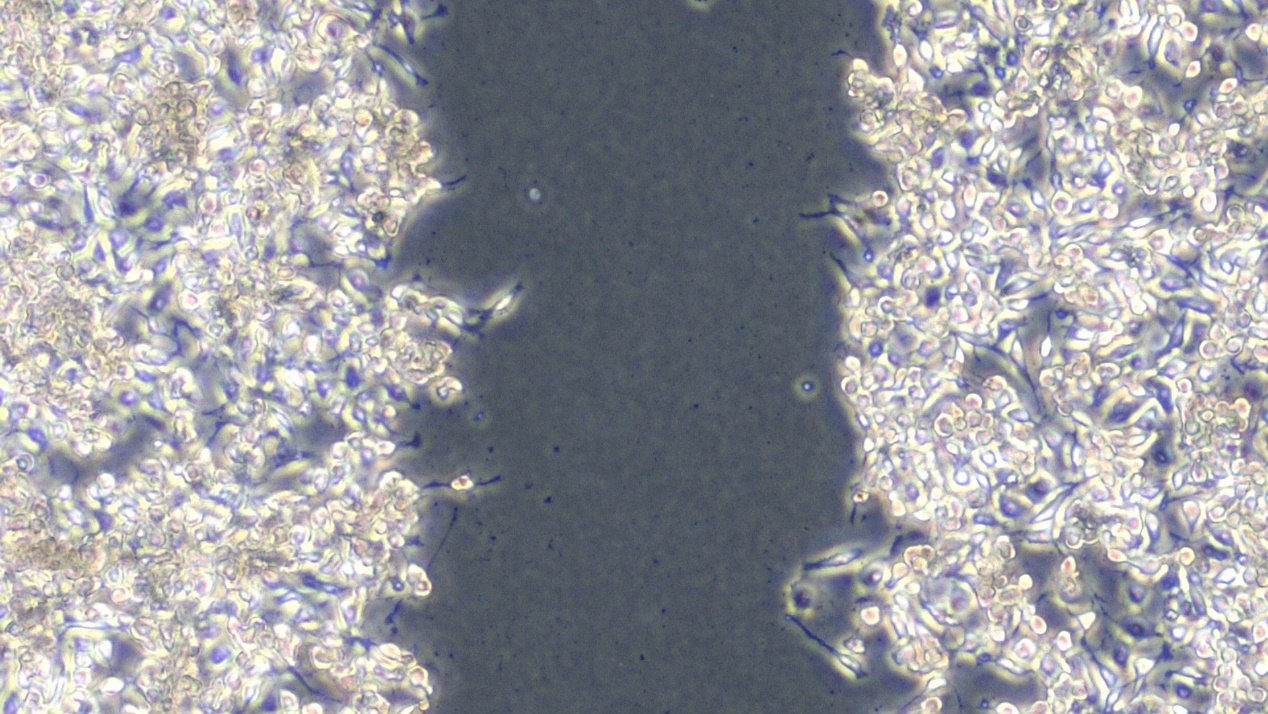

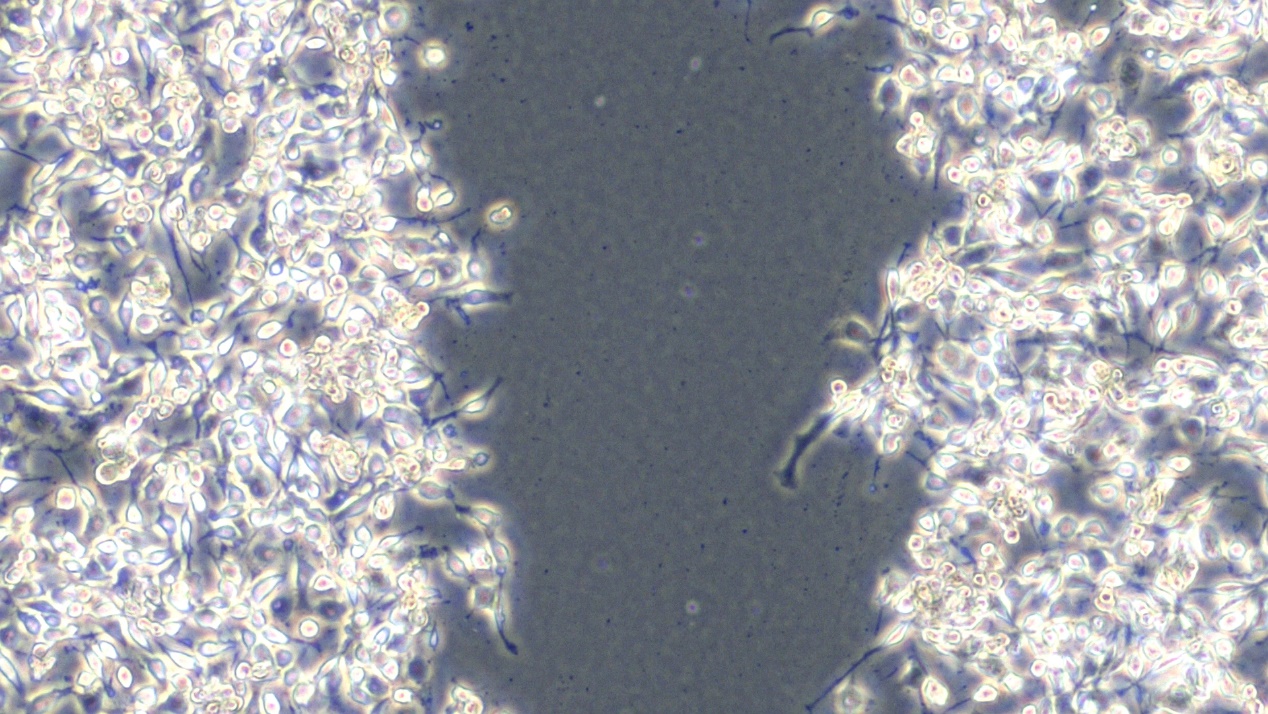

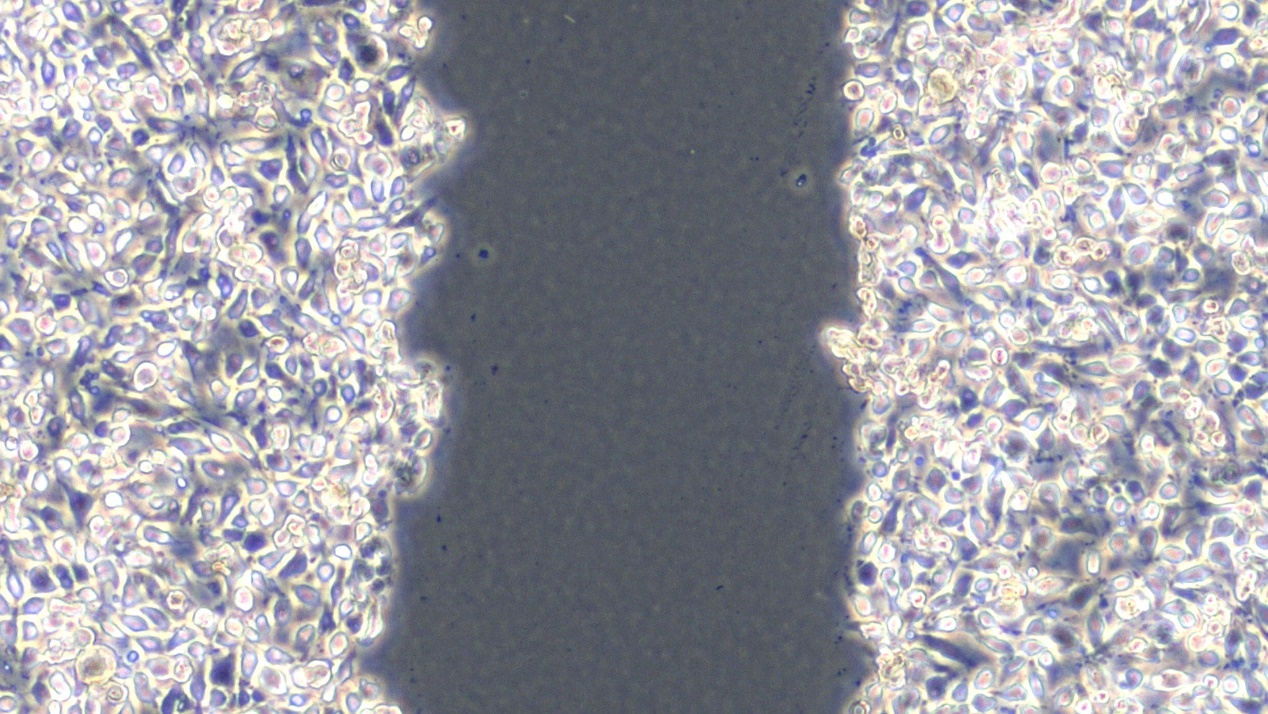

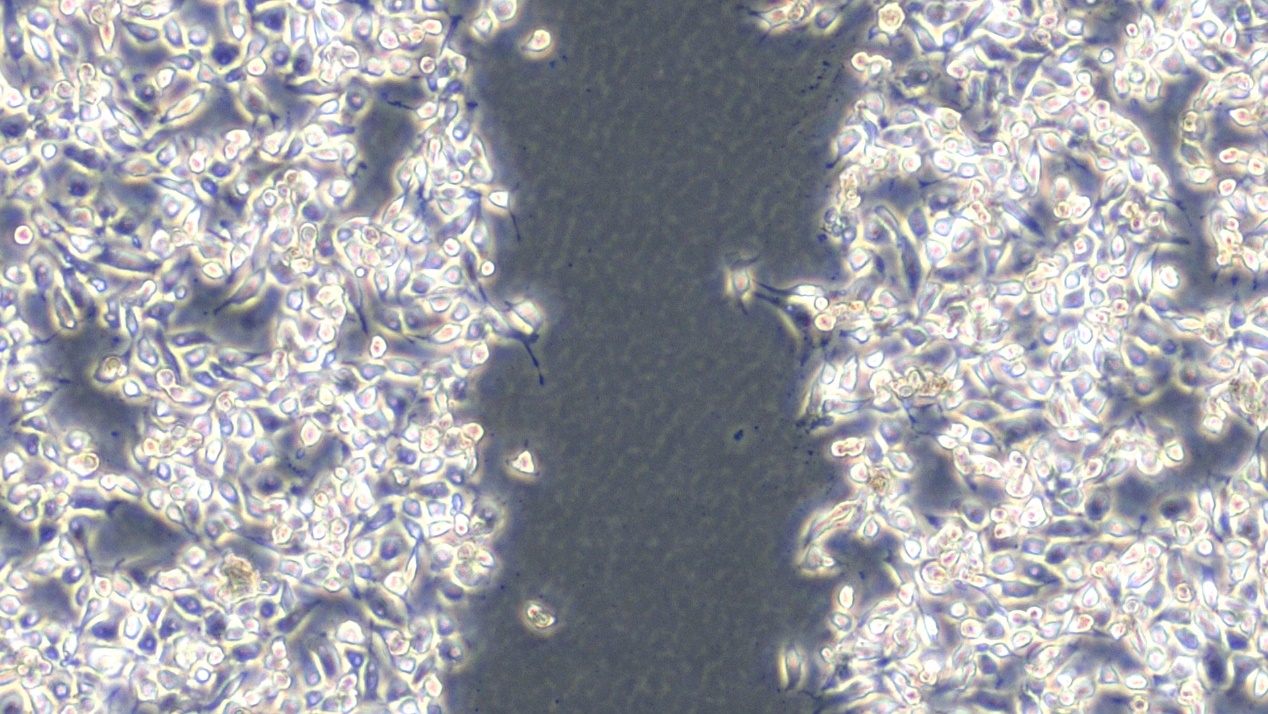

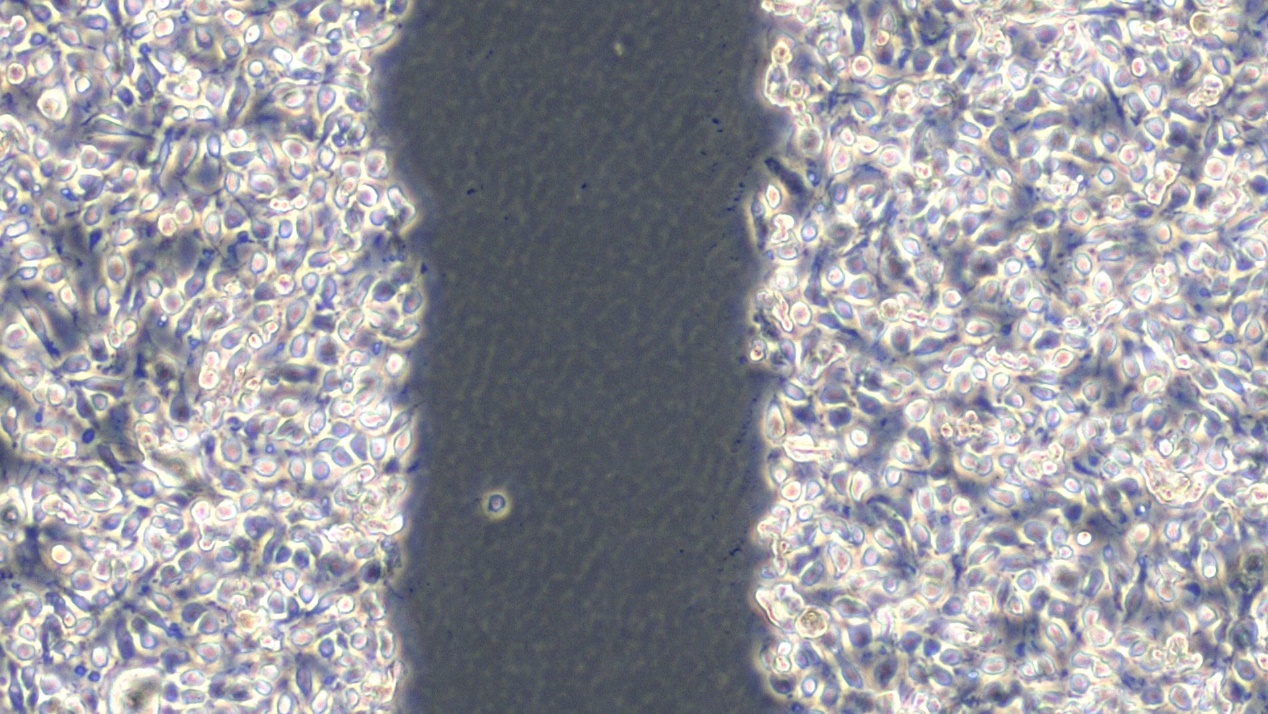

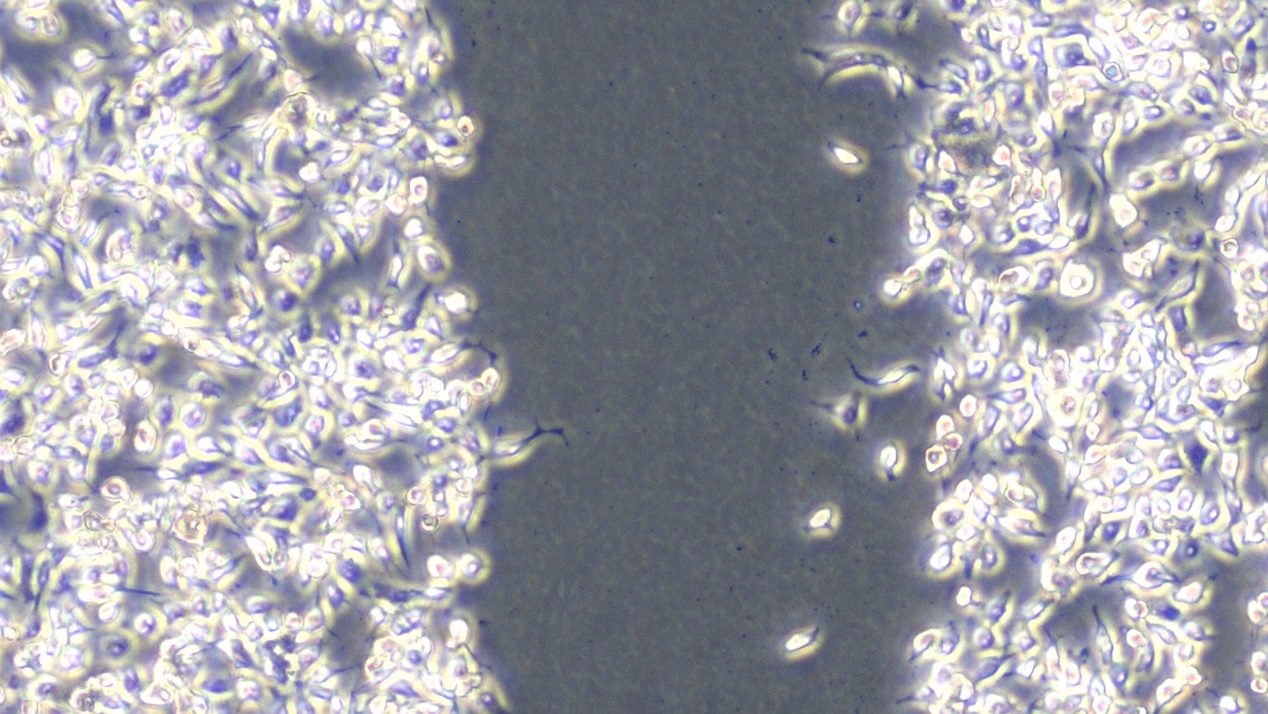

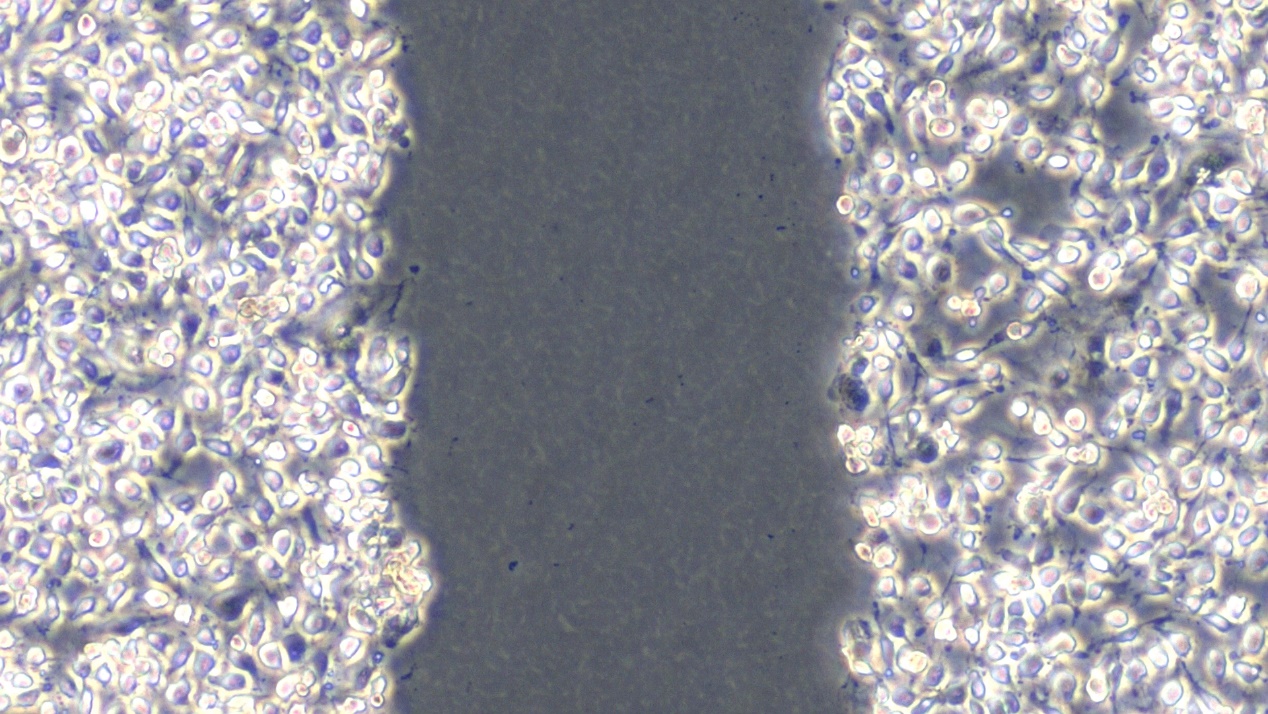

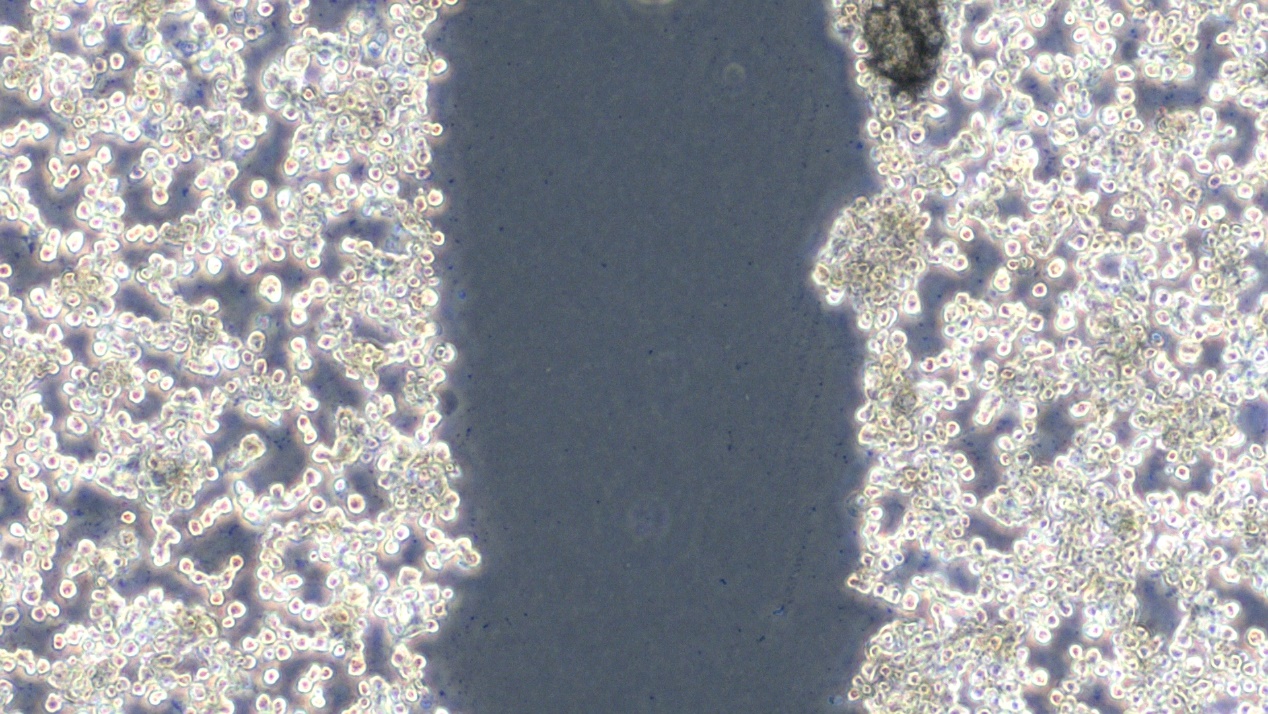

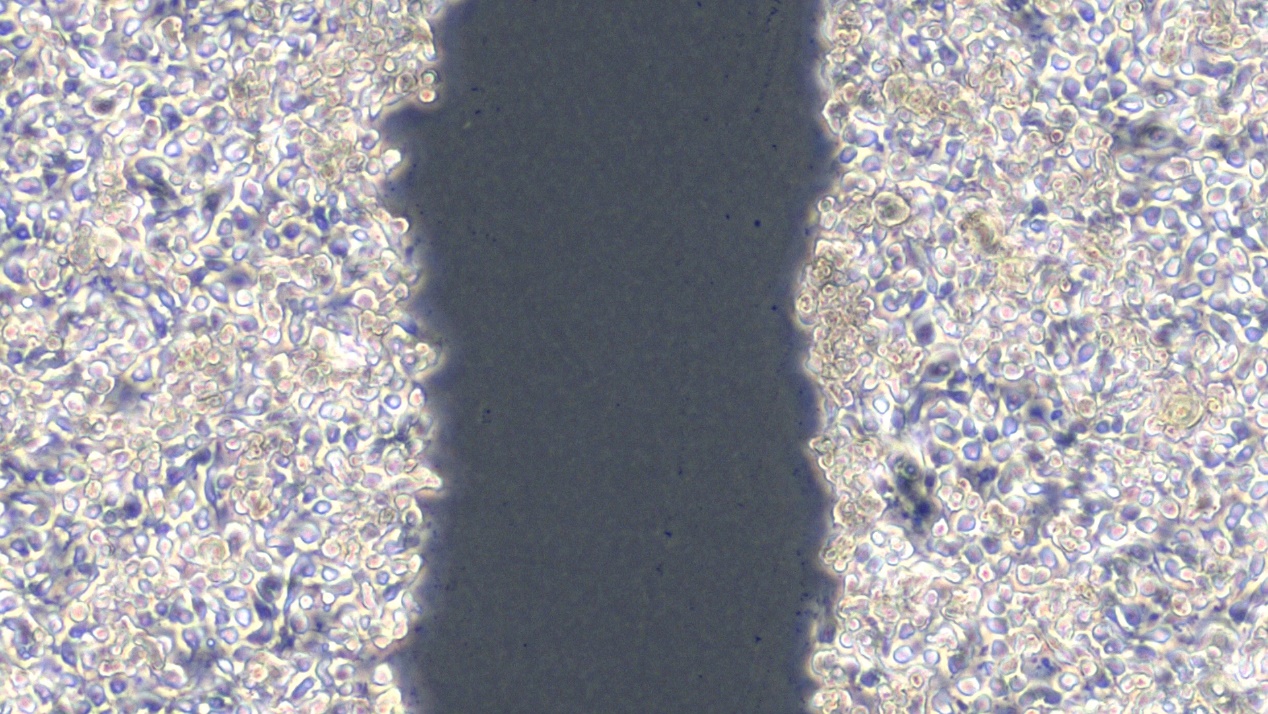

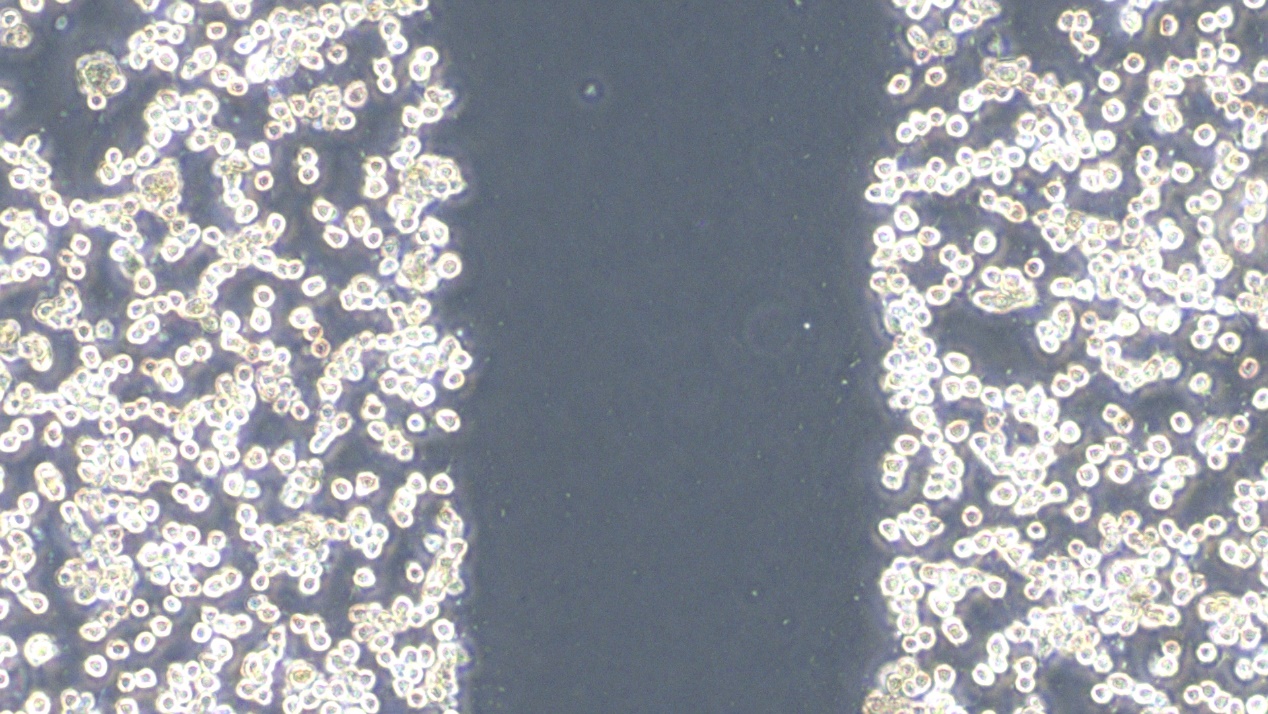

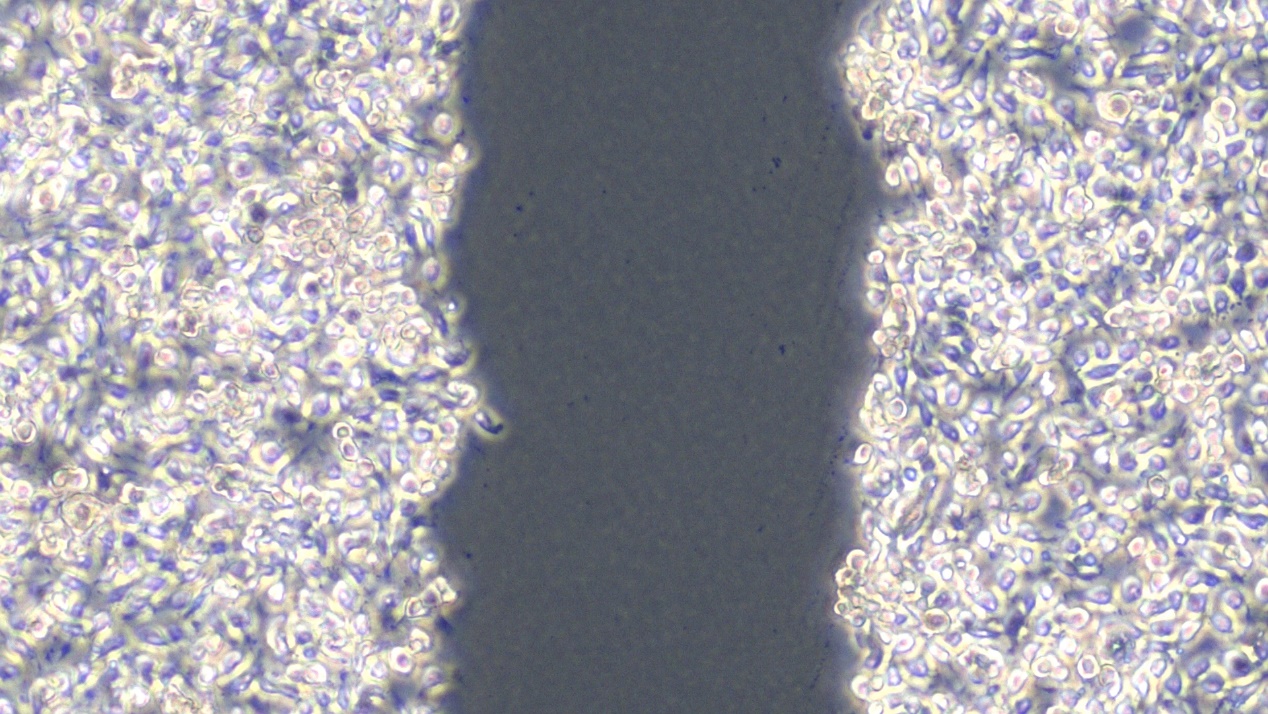

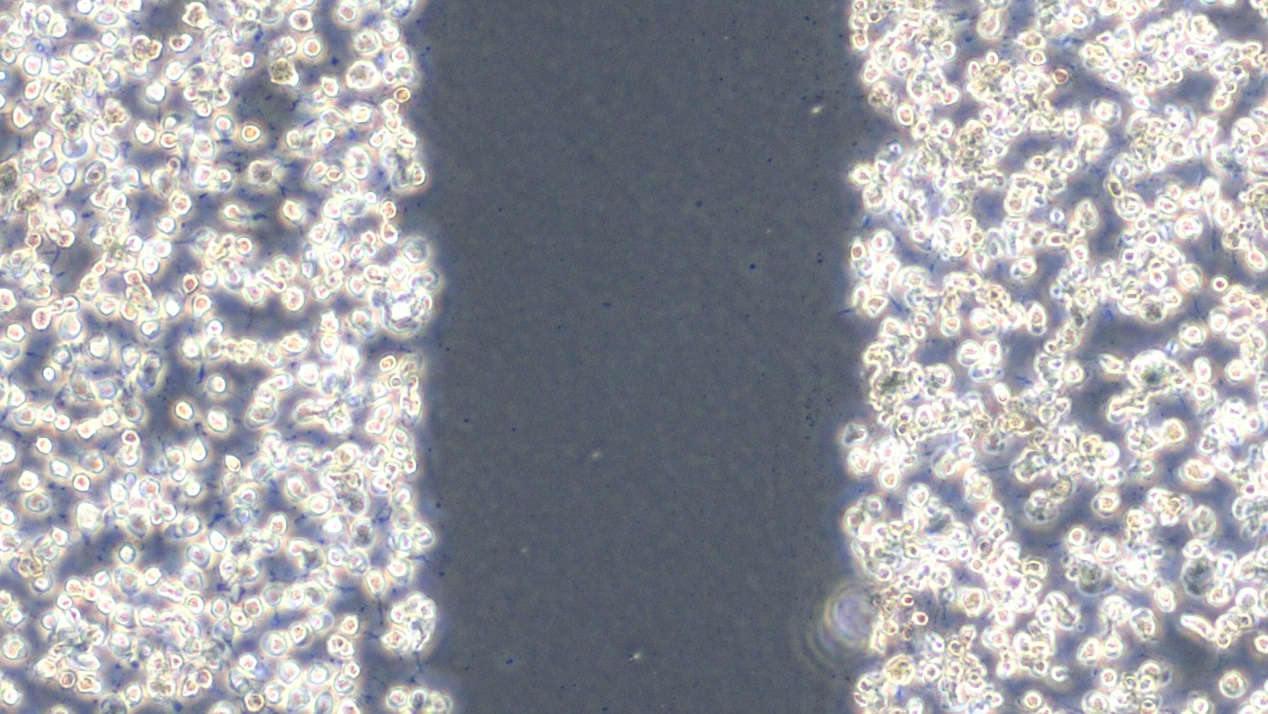

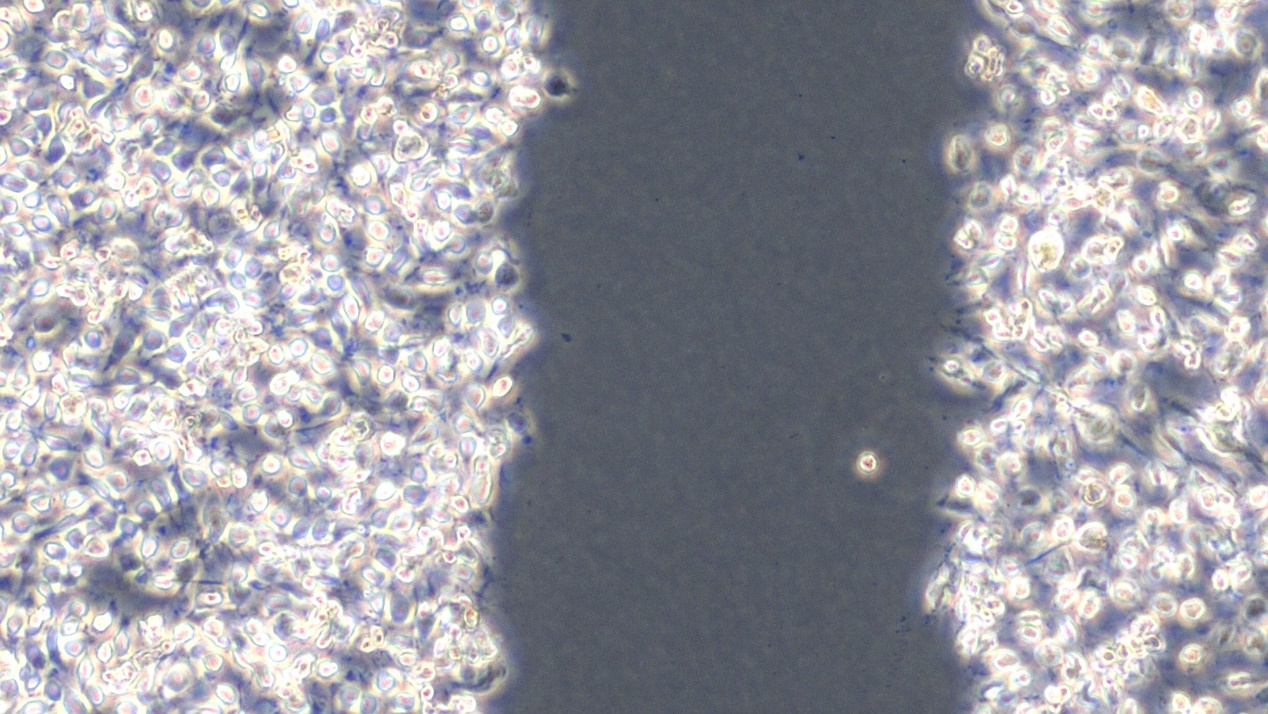

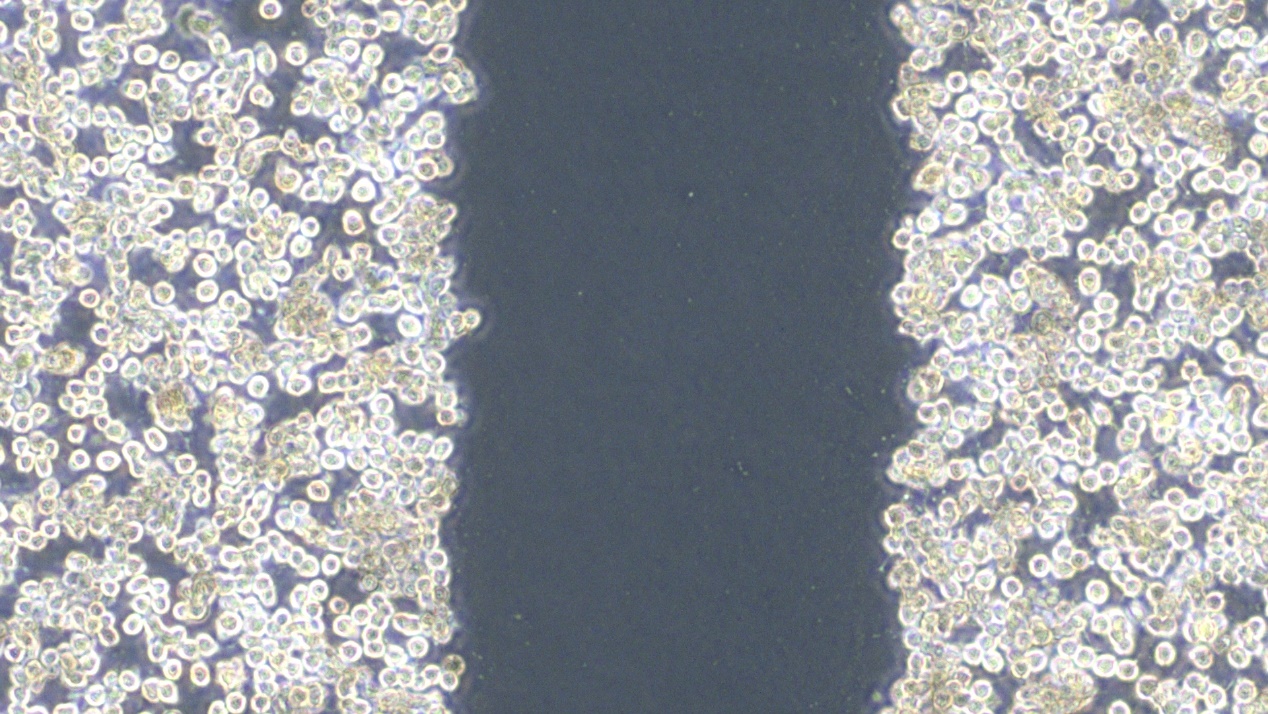

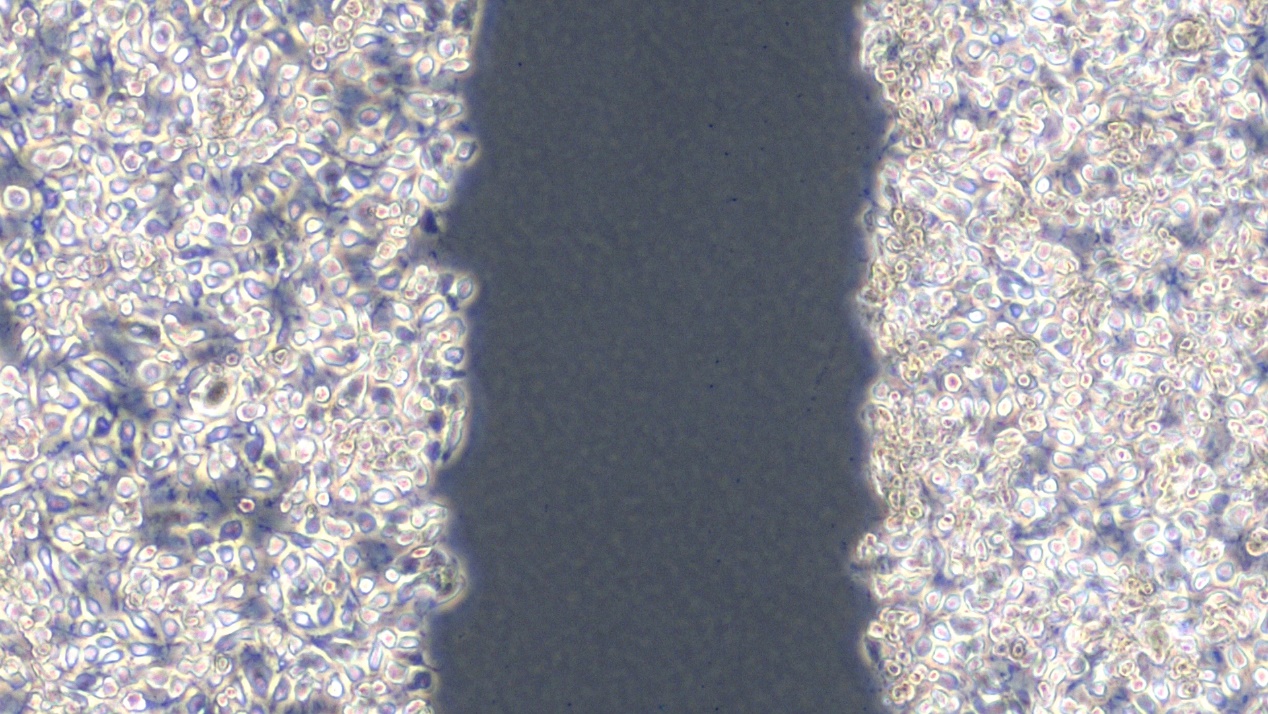

Supplement: Supplemental Information 2 [file peerj-13-19085-s002.zip › Chaetoglobosin A induces T-24 apoptosis in human bladder cancer/2. cell migration/2.docx]

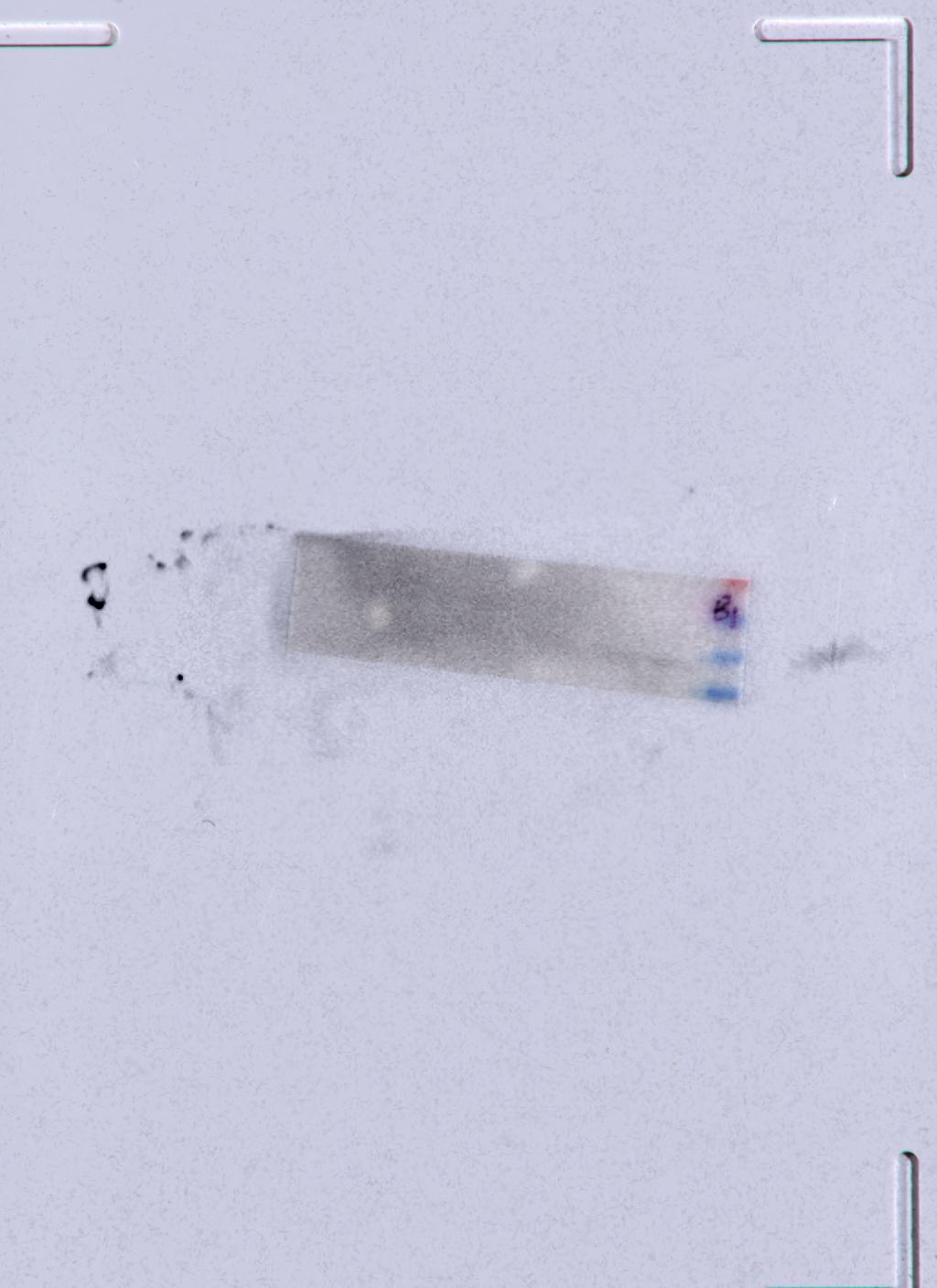

Supplement: Supplemental Information 2 [file peerj-13-19085-s002.zip › Chaetoglobosin A induces T-24 apoptosis in human bladder cancer/5.cells cycle/WB/CCNB1/22.5.3 ccnb1 2022.05.03_13.52.27_Ch/22.5.3 ccnb1 2022.05.03_13.52.27_Ch+Marker.jpg]

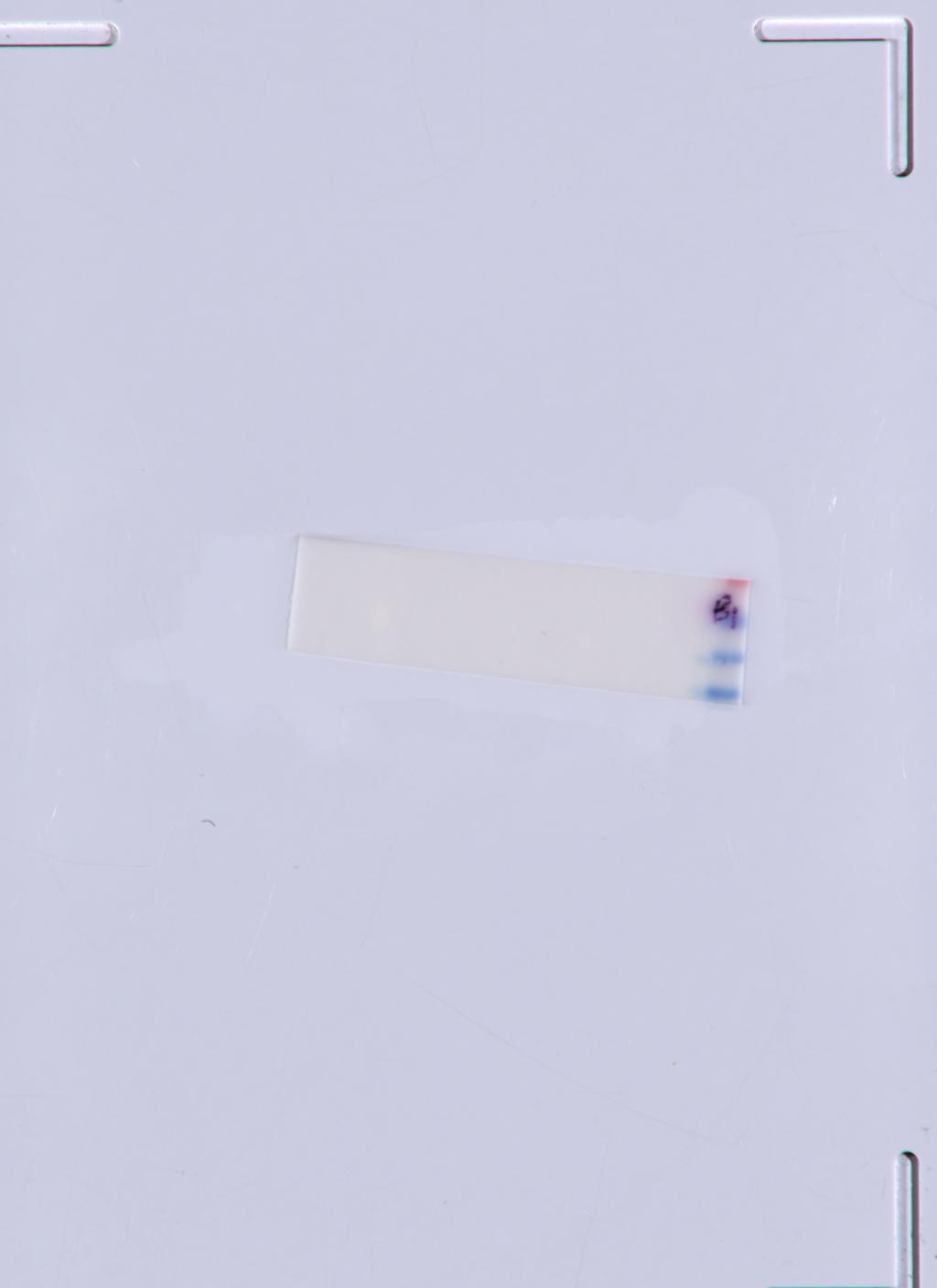

Supplement: Supplemental Information 2 [file peerj-13-19085-s002.zip › Chaetoglobosin A induces T-24 apoptosis in human bladder cancer/5.cells cycle/WB/CCNB1/22.5.3 ccnb1 2022.05.03_13.52.27_Ch/22.5.3 ccnb1 2022.05.03_13.52.27_Ch-Marker.jpg]

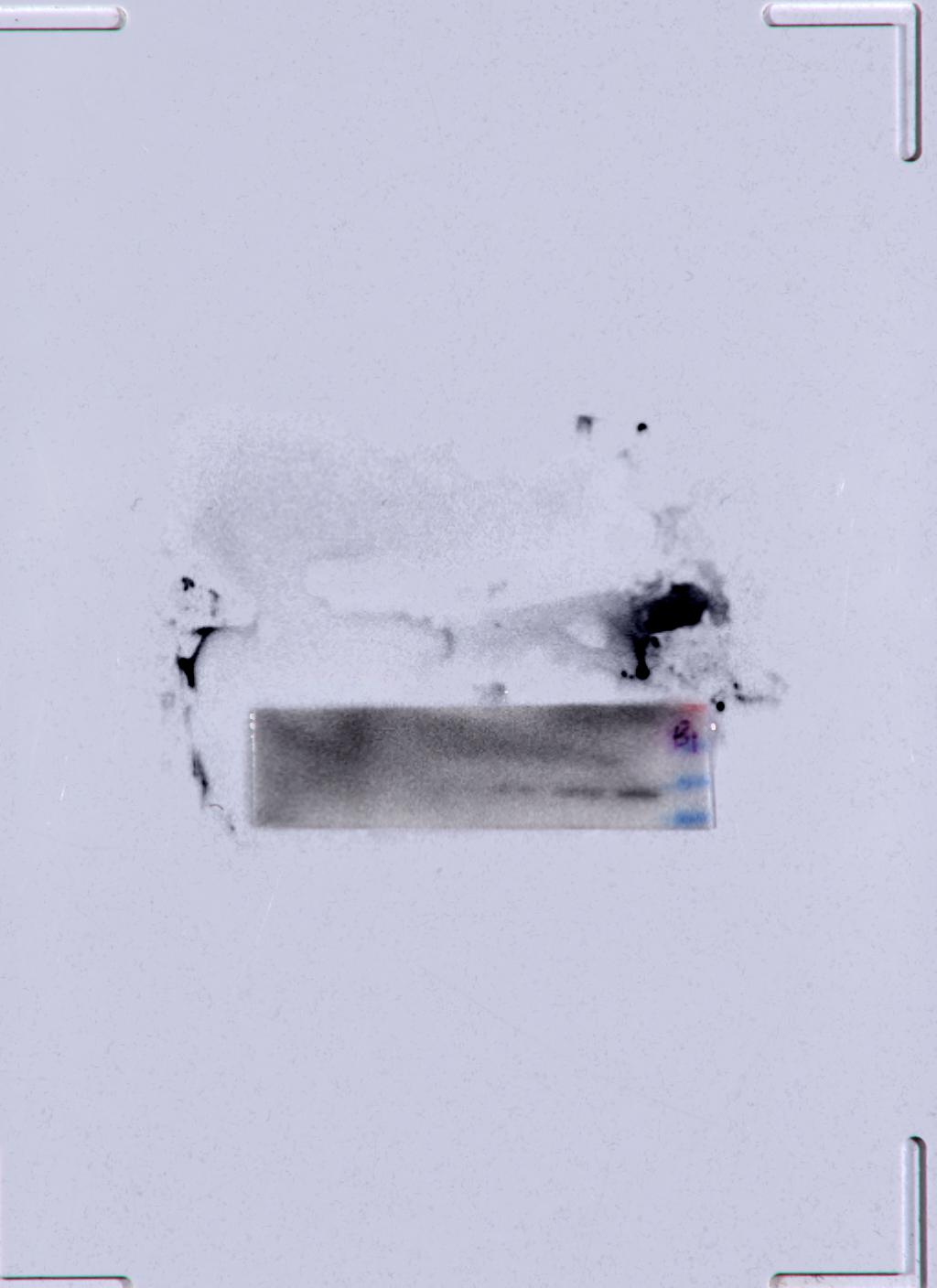

Supplement: Supplemental Information 2 [file peerj-13-19085-s002.zip › Chaetoglobosin A induces T-24 apoptosis in human bladder cancer/5.cells cycle/WB/CCNB1/22.5.3 ccnb1-1 2022.05.03_14.42.26_Ch/22.5.3 ccnb1-1 2022.05.03_14.42.26_Ch+Marker.jpg]

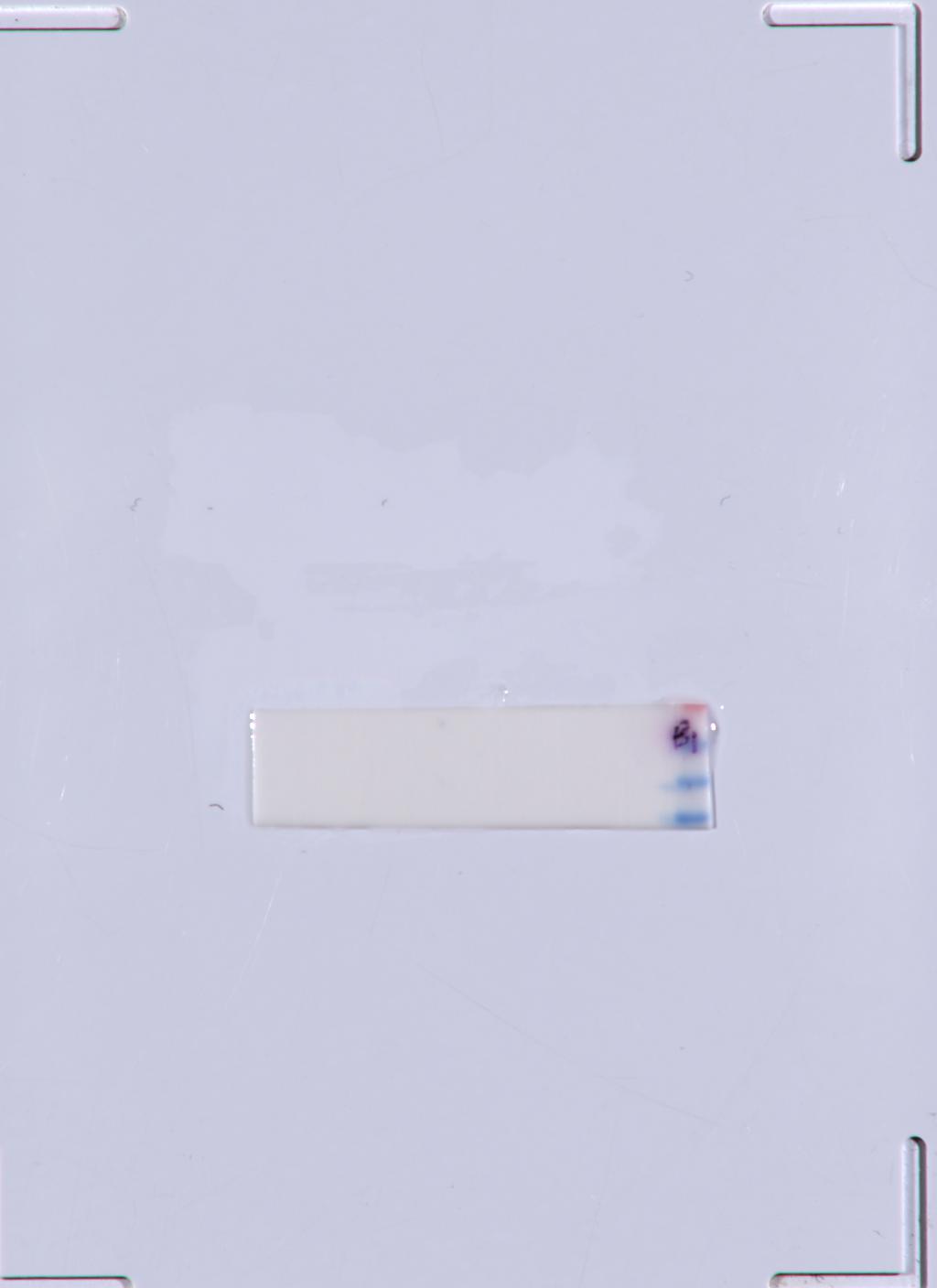

Supplement: Supplemental Information 2 [file peerj-13-19085-s002.zip › Chaetoglobosin A induces T-24 apoptosis in human bladder cancer/5.cells cycle/WB/CCNB1/22.5.3 ccnb1-1 2022.05.03_14.42.26_Ch/22.5.3 ccnb1-1 2022.05.03_14.42.26_Ch-Marker.jpg]

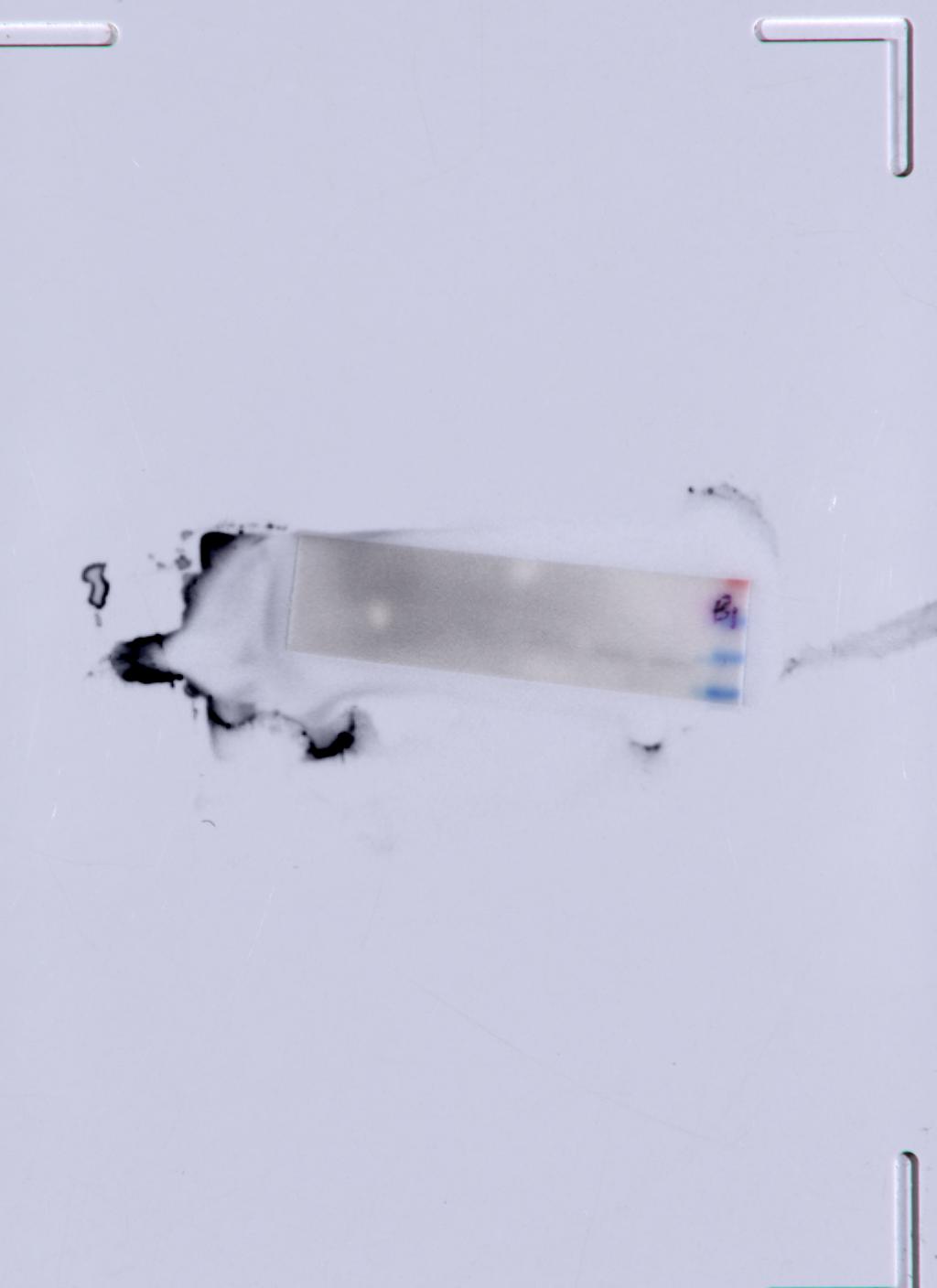

Supplement: Supplemental Information 2 [file peerj-13-19085-s002.zip › Chaetoglobosin A induces T-24 apoptosis in human bladder cancer/5.cells cycle/WB/CCNB1/22.5.3 ccnb1-2 2022.05.03_13.54.08_Ch/22.5.3 ccnb1-2 2022.05.03_13.54.08_Ch+Marker.jpg]

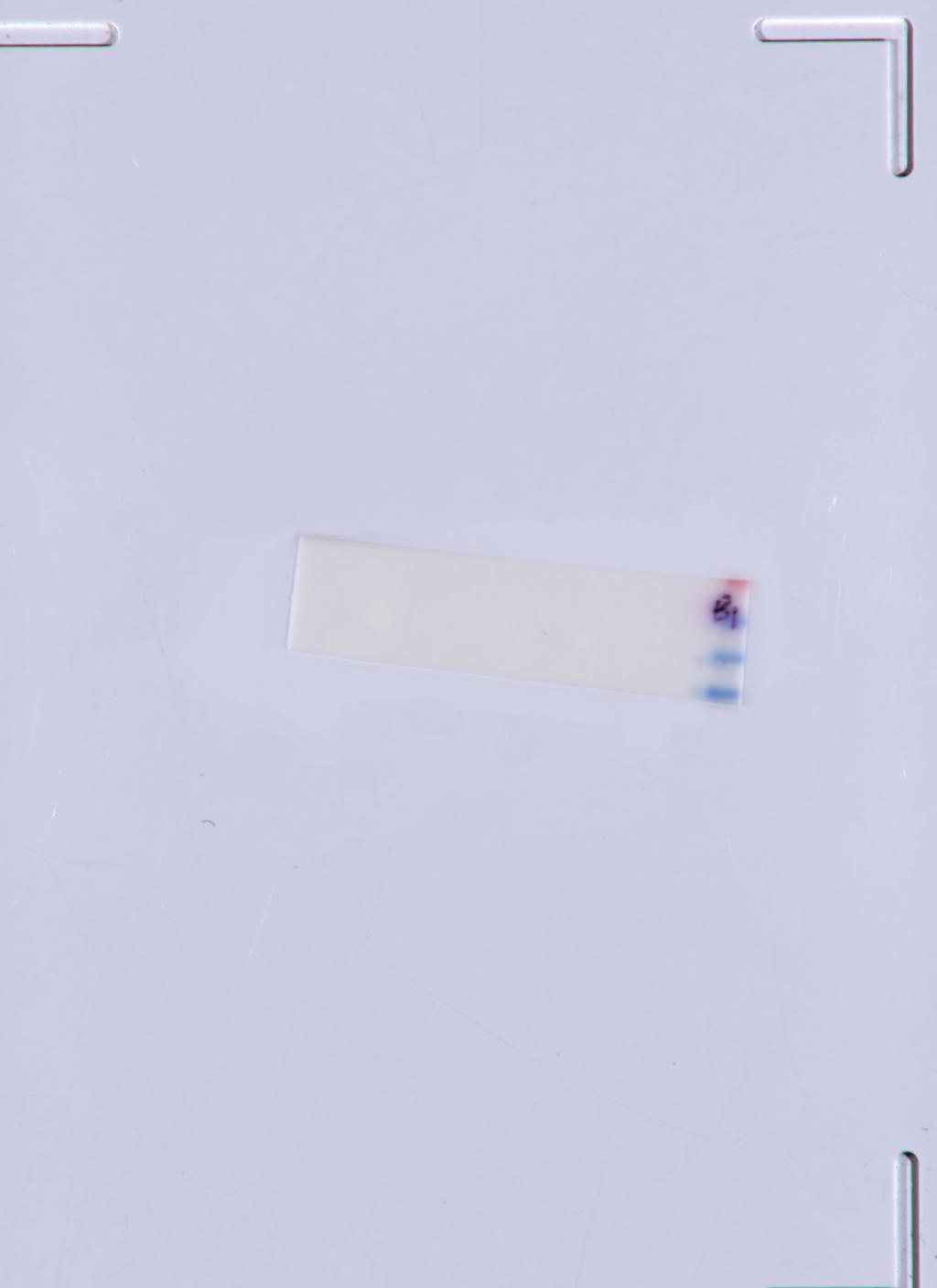

Supplement: Supplemental Information 2 [file peerj-13-19085-s002.zip › Chaetoglobosin A induces T-24 apoptosis in human bladder cancer/5.cells cycle/WB/CCNB1/22.5.3 ccnb1-2 2022.05.03_13.54.08_Ch/22.5.3 ccnb1-2 2022.05.03_13.54.08_Ch-Marker.jpg]

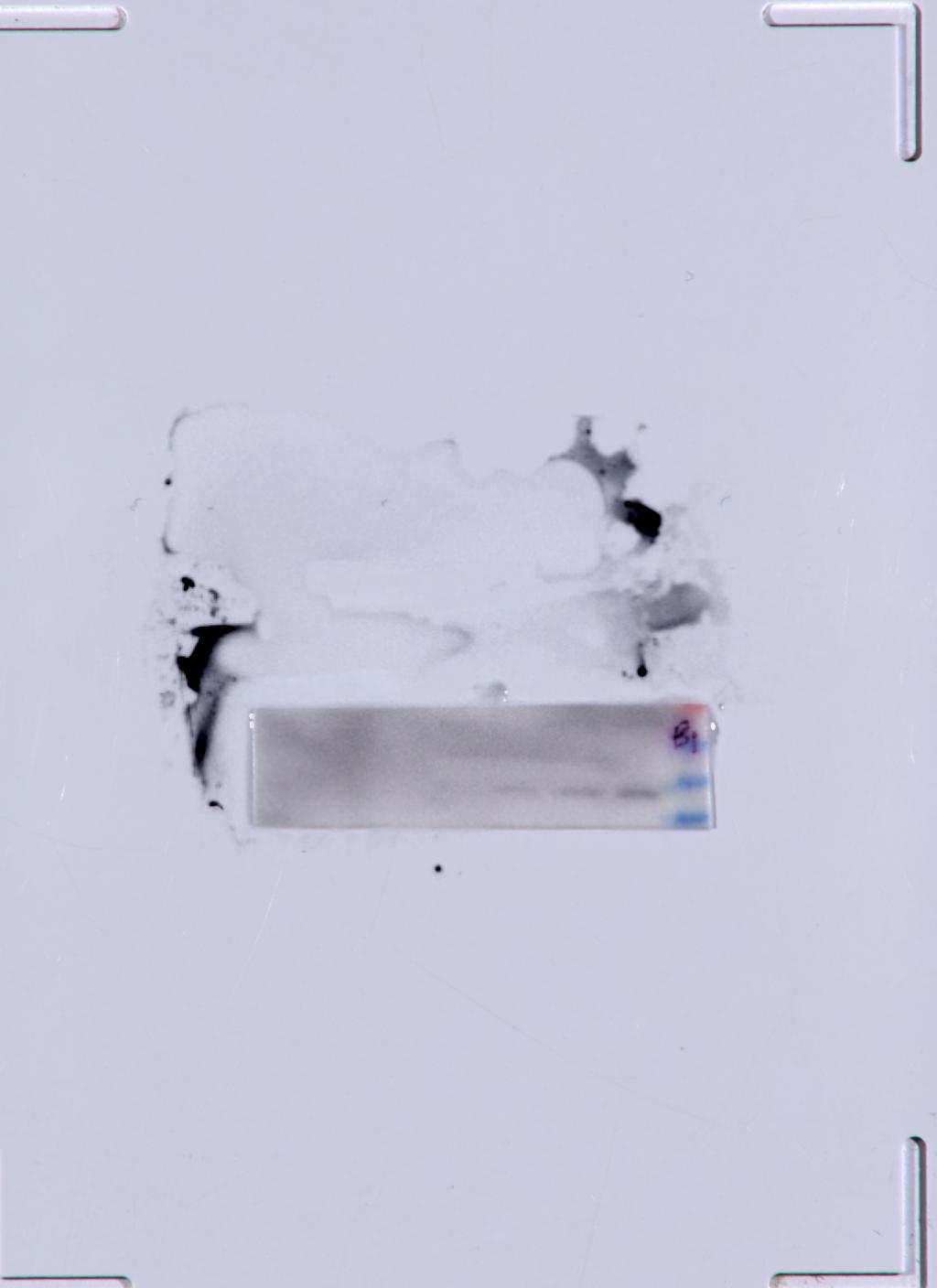

Supplement: Supplemental Information 2 [file peerj-13-19085-s002.zip › Chaetoglobosin A induces T-24 apoptosis in human bladder cancer/5.cells cycle/WB/CCNB1/22.5.3 ccnb1-3 2022.05.03_14.44.10_Ch/22.5.3 ccnb1-3 2022.05.03_14.44.10_Ch+Marker.jpg]

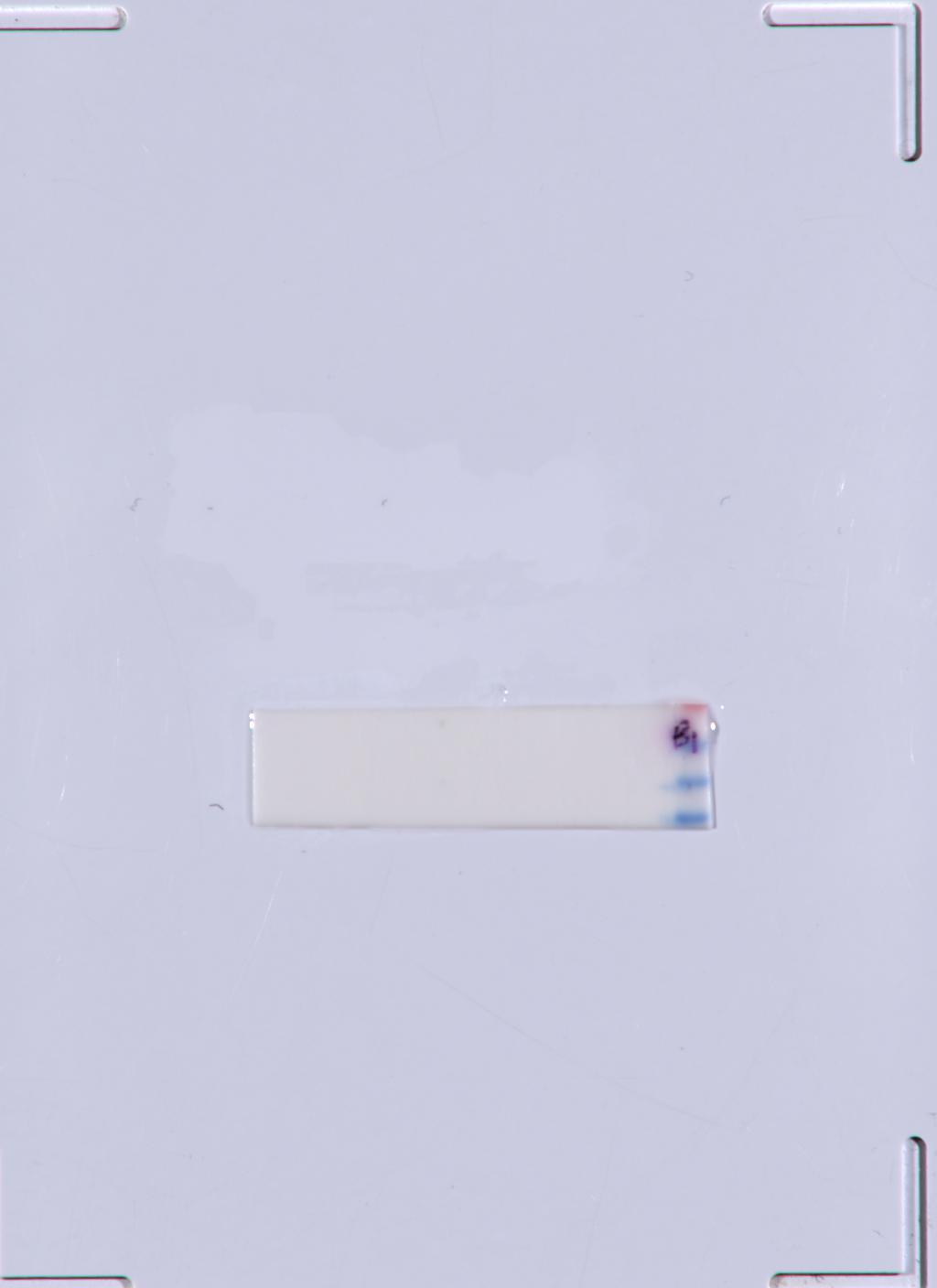

Supplement: Supplemental Information 2 [file peerj-13-19085-s002.zip › Chaetoglobosin A induces T-24 apoptosis in human bladder cancer/5.cells cycle/WB/CCNB1/22.5.3 ccnb1-3 2022.05.03_14.44.10_Ch/22.5.3 ccnb1-3 2022.05.03_14.44.10_Ch-Marker.jpg]

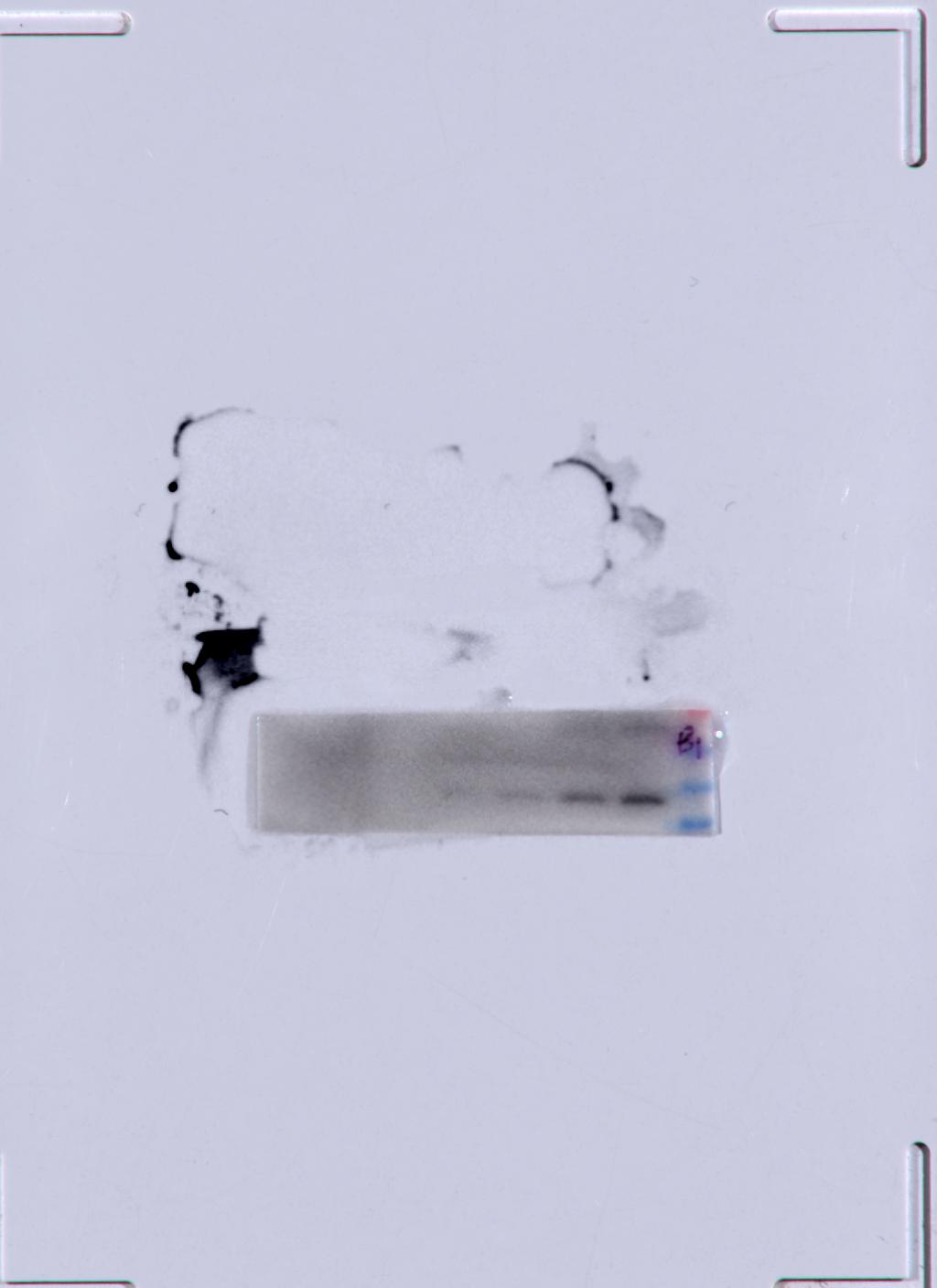

Supplement: Supplemental Information 2 [file peerj-13-19085-s002.zip › Chaetoglobosin A induces T-24 apoptosis in human bladder cancer/5.cells cycle/WB/CCNB1/22.5.3 ccnb1-4 2022.05.03_14.47.23_Ch/22.5.3 ccnb1-4 2022.05.03_14.47.23_Ch+Marker.jpg]

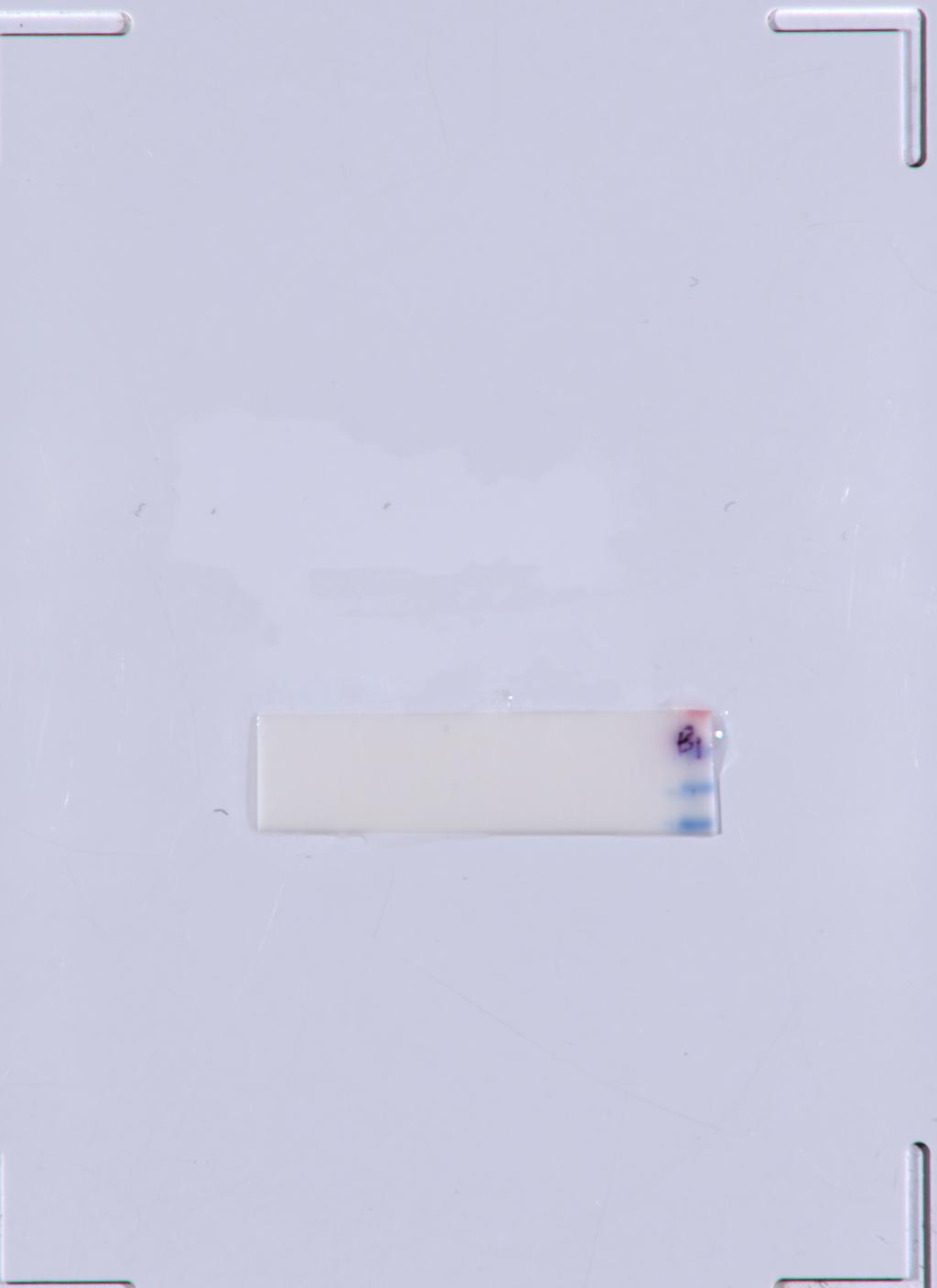

Supplement: Supplemental Information 2 [file peerj-13-19085-s002.zip › Chaetoglobosin A induces T-24 apoptosis in human bladder cancer/5.cells cycle/WB/CCNB1/22.5.3 ccnb1-4 2022.05.03_14.47.23_Ch/22.5.3 ccnb1-4 2022.05.03_14.47.23_Ch-Marker.jpg]

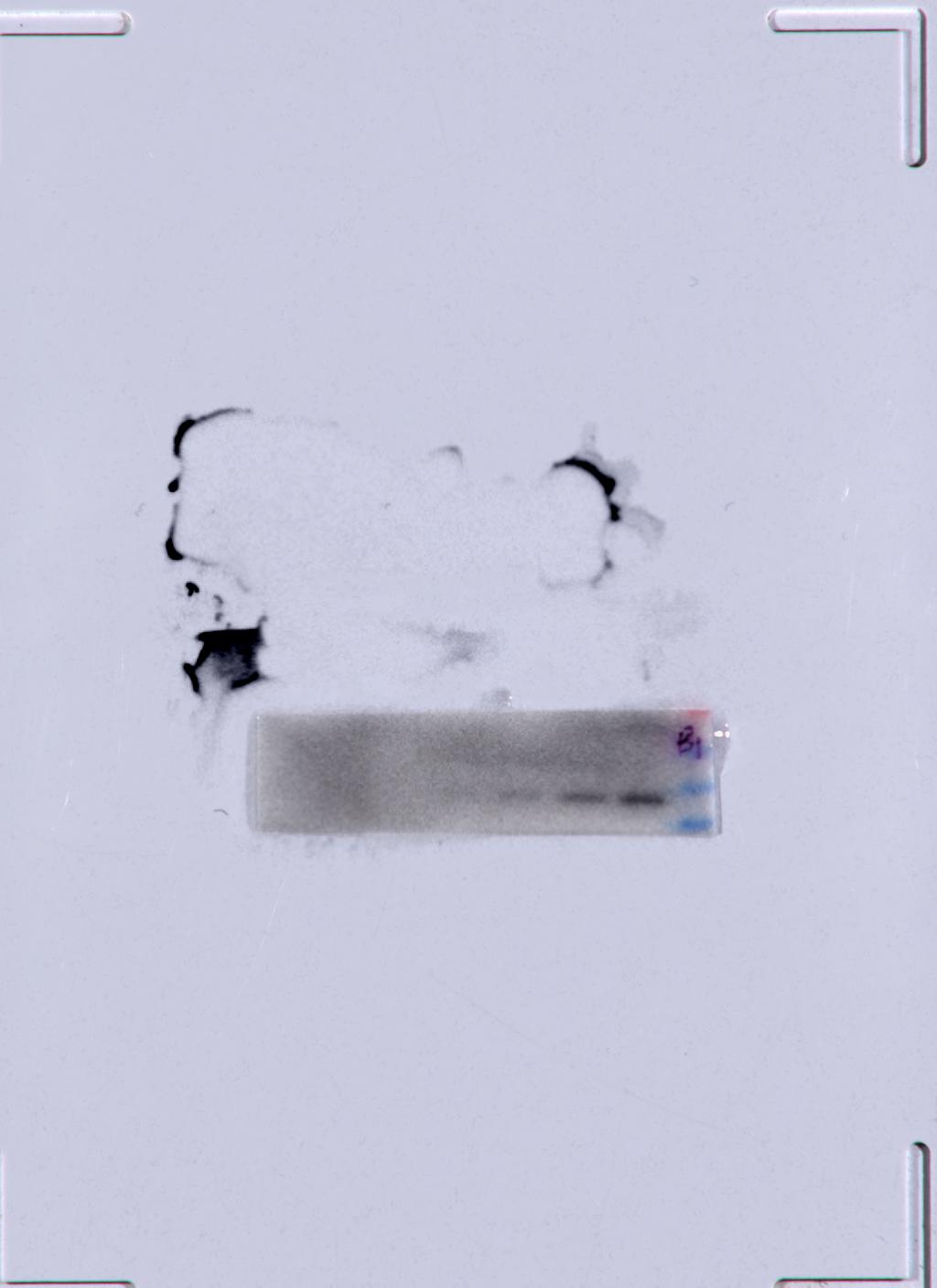

Supplement: Supplemental Information 2 [file peerj-13-19085-s002.zip › Chaetoglobosin A induces T-24 apoptosis in human bladder cancer/5.cells cycle/WB/CCNB1/22.5.3 ccnb1-5 2022.05.03_14.48.58_Ch/22.5.3 ccnb1-5 2022.05.03_14.48.58_Ch+Marker.jpg]

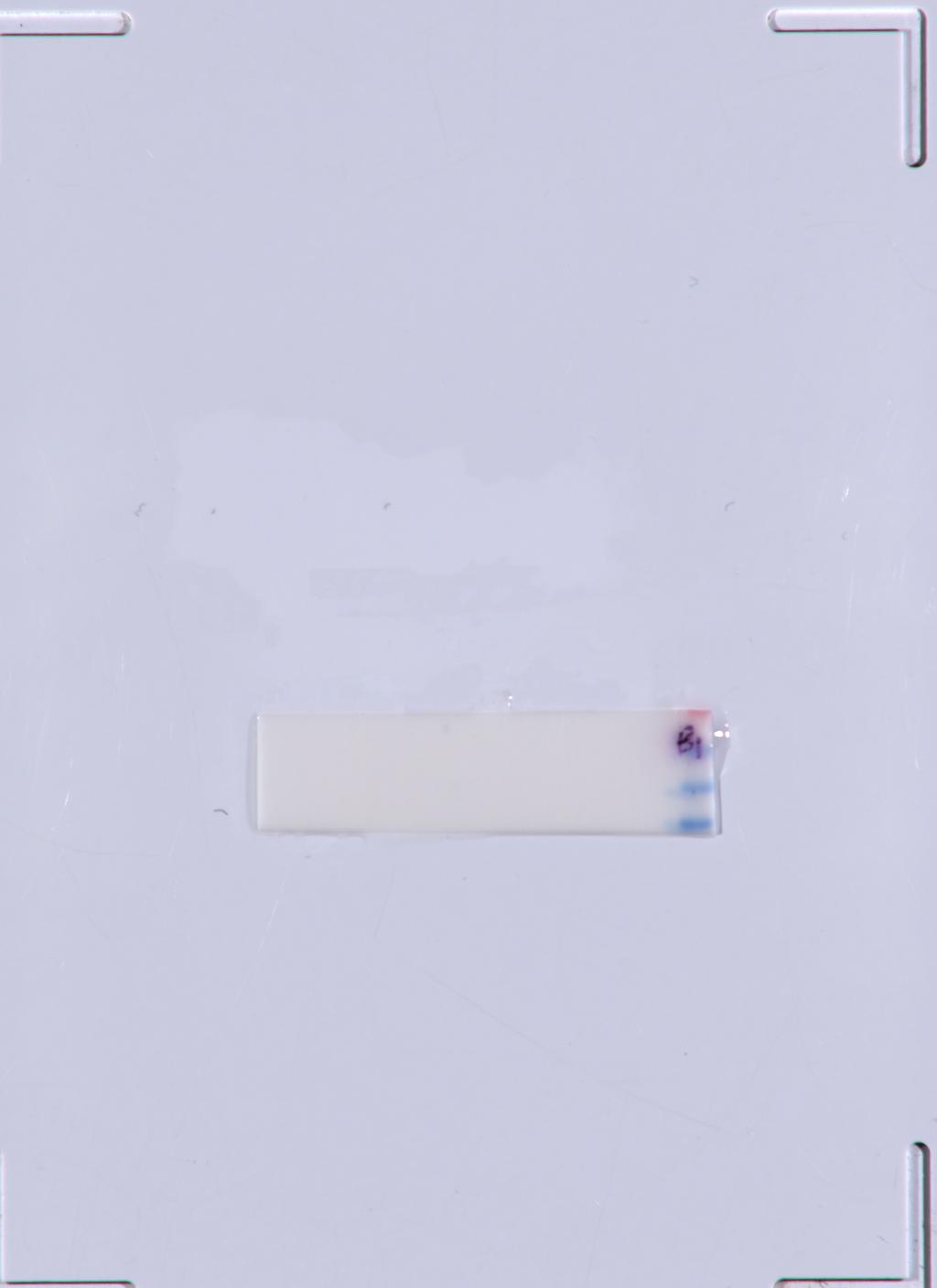

Supplement: Supplemental Information 2 [file peerj-13-19085-s002.zip › Chaetoglobosin A induces T-24 apoptosis in human bladder cancer/5.cells cycle/WB/CCNB1/22.5.3 ccnb1-5 2022.05.03_14.48.58_Ch/22.5.3 ccnb1-5 2022.05.03_14.48.58_Ch-Marker.jpg]

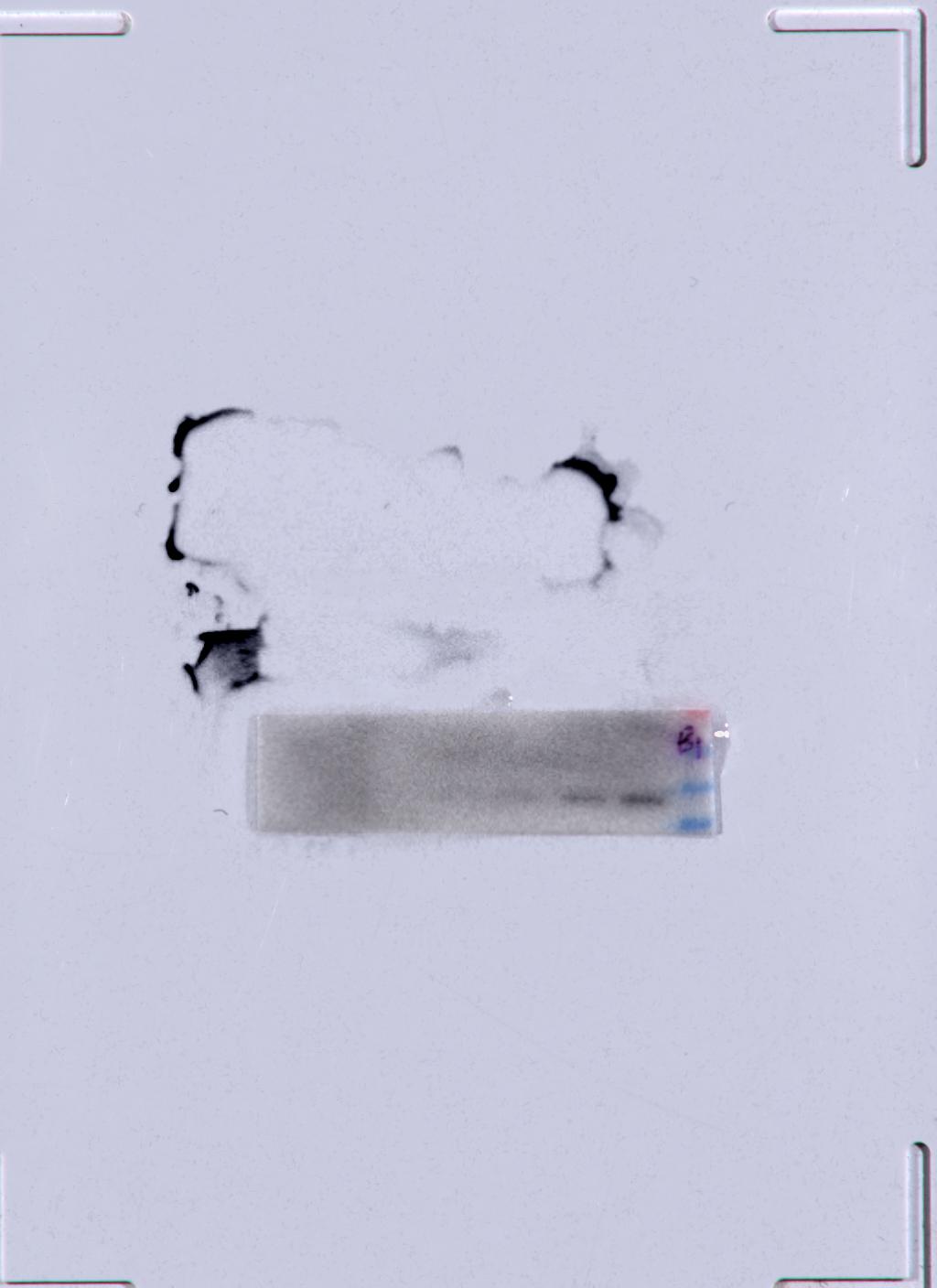

Supplement: Supplemental Information 2 [file peerj-13-19085-s002.zip › Chaetoglobosin A induces T-24 apoptosis in human bladder cancer/5.cells cycle/WB/CCNB1/22.5.3 ccnb1-6 2022.05.03_14.50.14_Ch/22.5.3 ccnb1-6 2022.05.03_14.50.14_Ch+Marker.jpg]

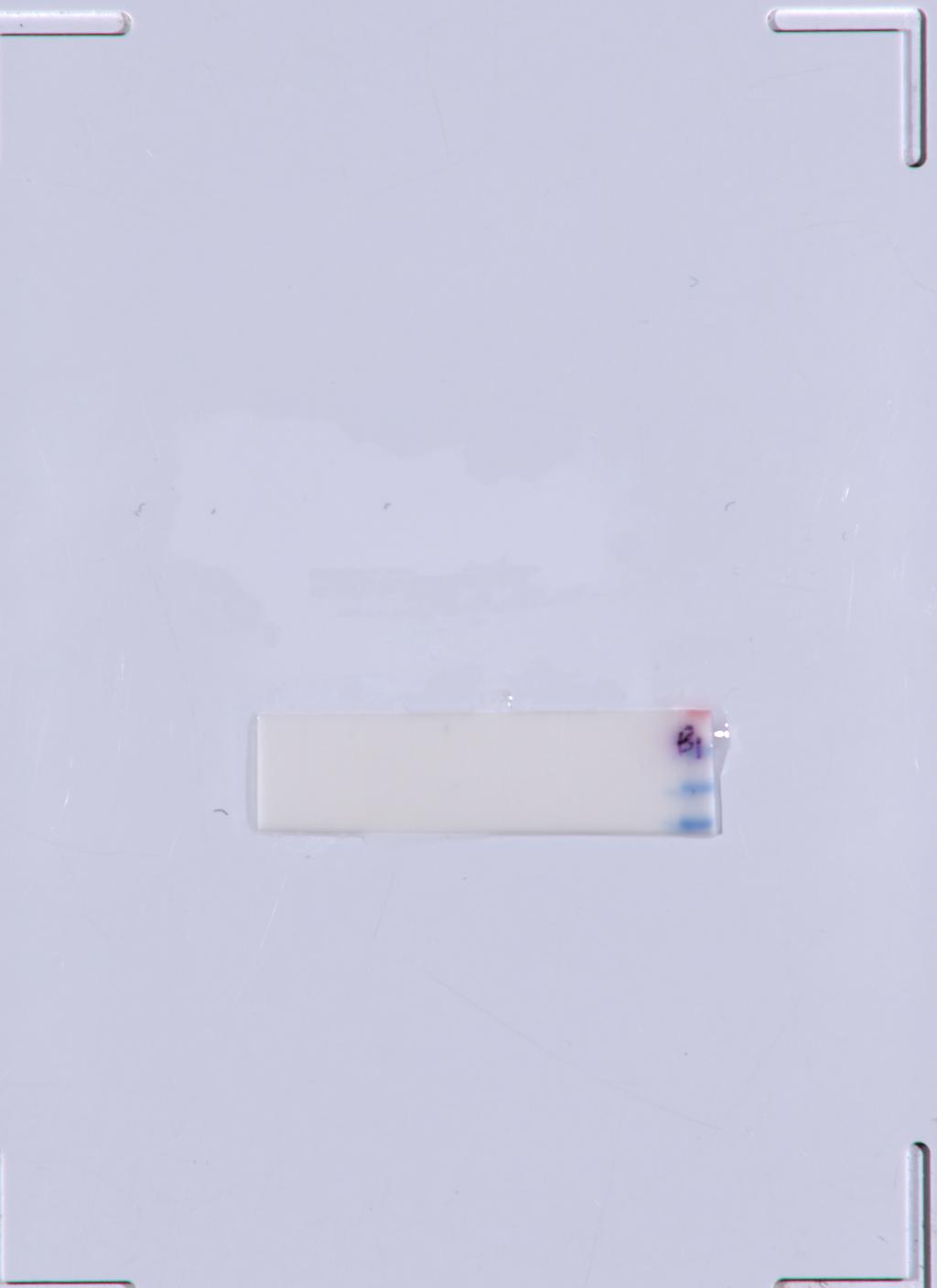

Supplement: Supplemental Information 2 [file peerj-13-19085-s002.zip › Chaetoglobosin A induces T-24 apoptosis in human bladder cancer/5.cells cycle/WB/CCNB1/22.5.3 ccnb1-6 2022.05.03_14.50.14_Ch/22.5.3 ccnb1-6 2022.05.03_14.50.14_Ch-Marker.jpg]

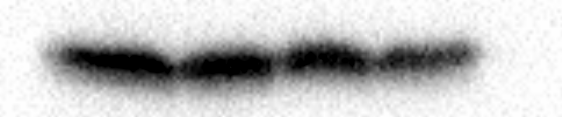

Supplement: Supplemental Information 2 [file peerj-13-19085-s002.zip › Chaetoglobosin A induces T-24 apoptosis in human bladder cancer/5.cells cycle/WB/CDK/HP 2022-11-22_11h31m45s_Exposure_1.0sec.tif]

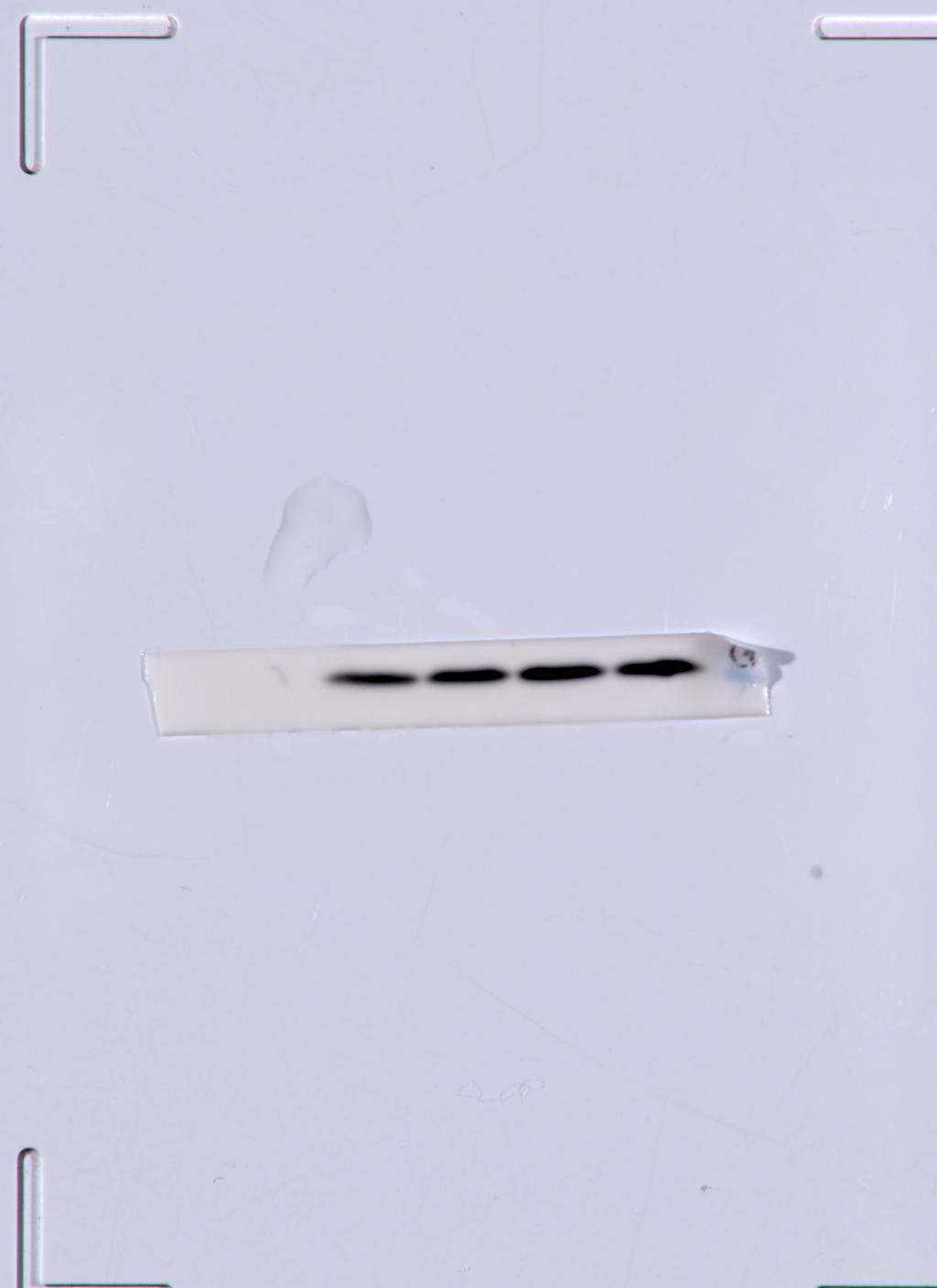

Supplement: Supplemental Information 2 [file peerj-13-19085-s002.zip › Chaetoglobosin A induces T-24 apoptosis in human bladder cancer/5.cells cycle/WB/GAPDH/22.3.16 gapdh-1 2022.03.16_19.21.42_Ch+Marker.jpg]

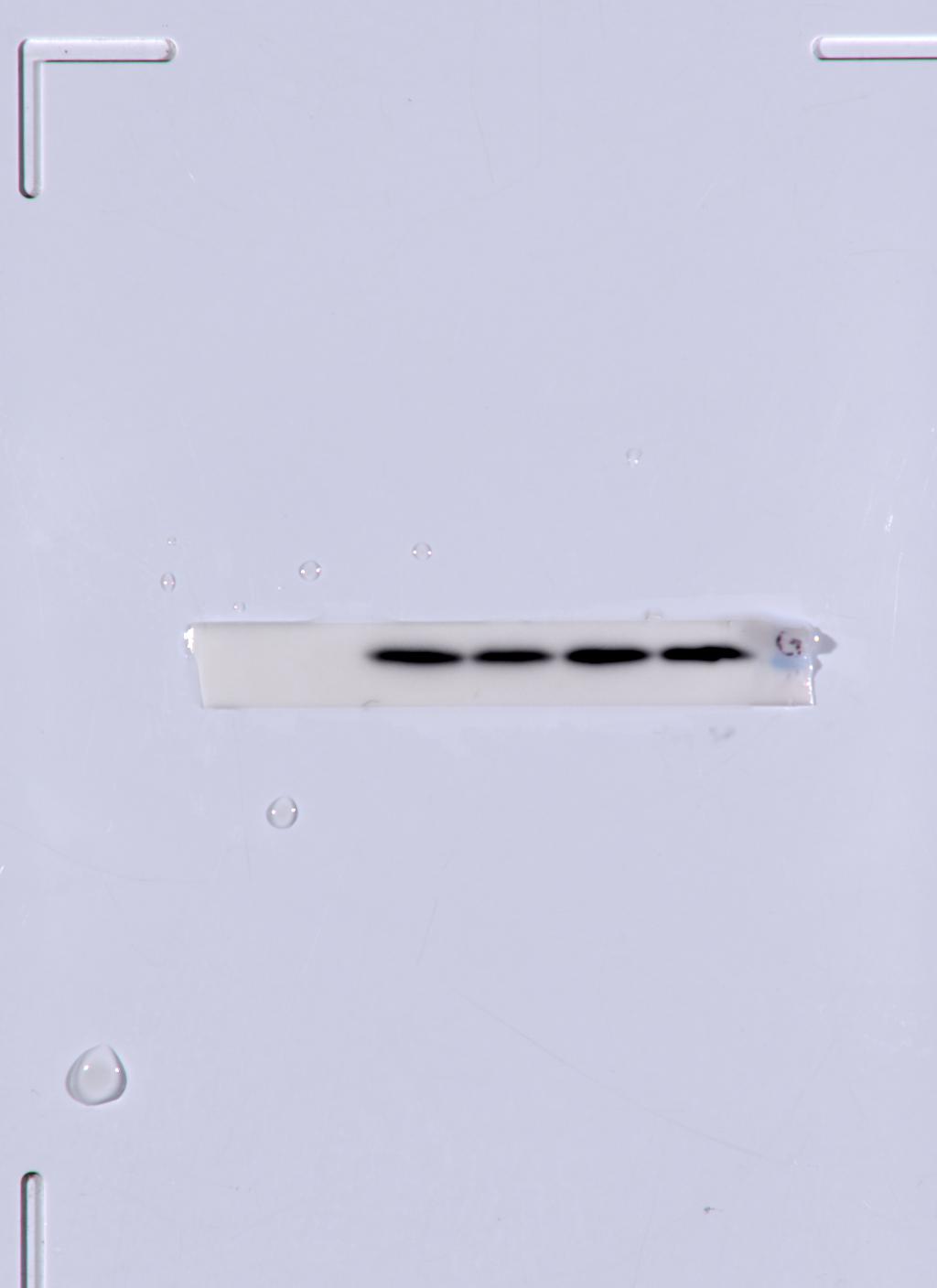

Supplement: Supplemental Information 2 [file peerj-13-19085-s002.zip › Chaetoglobosin A induces T-24 apoptosis in human bladder cancer/5.cells cycle/WB/GAPDH/22.3.16 gapdh-5 2022.03.16_20.05.47_Ch+Marker.jpg]

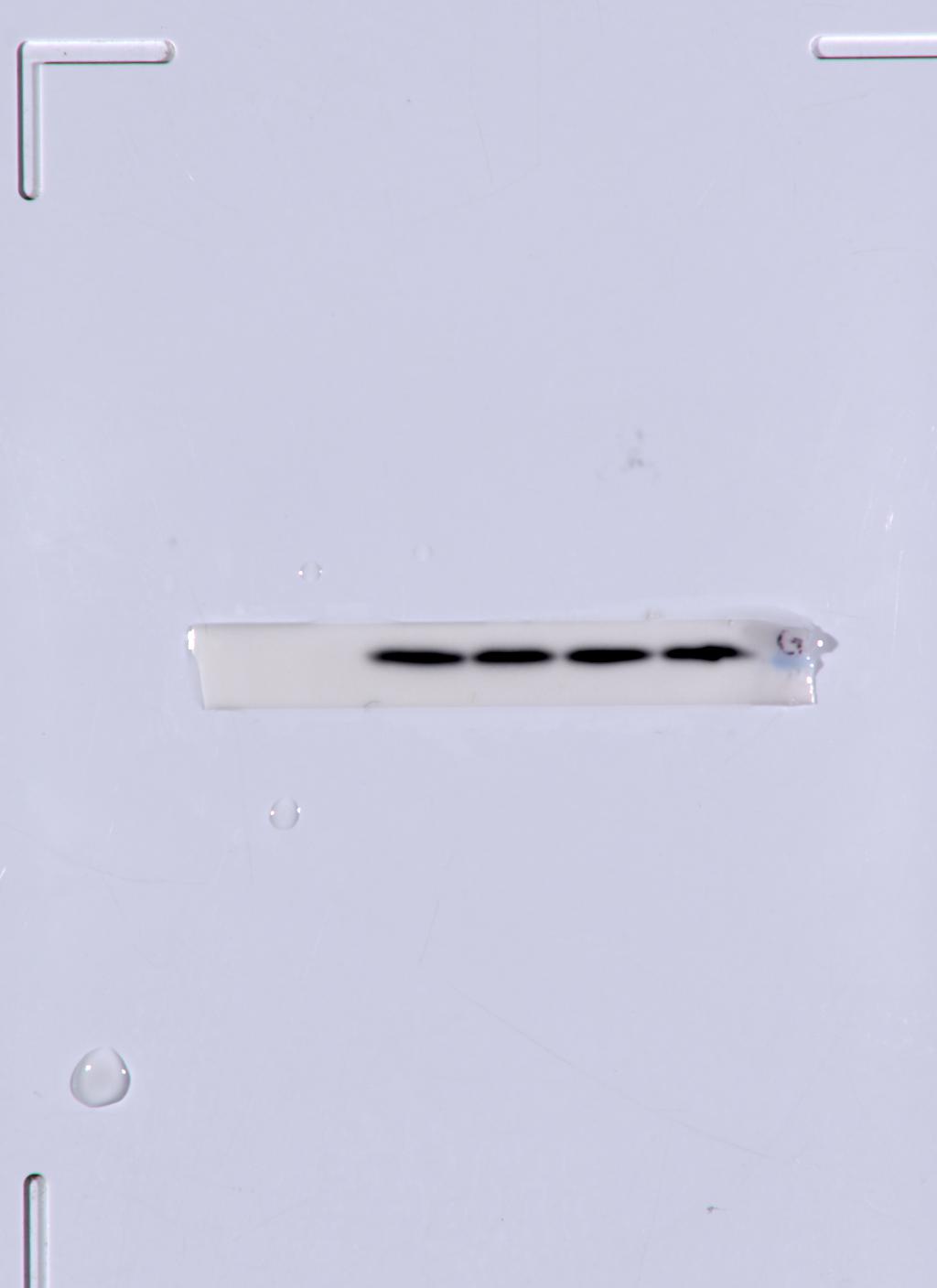

Supplement: Supplemental Information 2 [file peerj-13-19085-s002.zip › Chaetoglobosin A induces T-24 apoptosis in human bladder cancer/5.cells cycle/WB/GAPDH/22.3.16 gapdh8 2022.03.16_20.11.08_Ch+Marker.jpg]

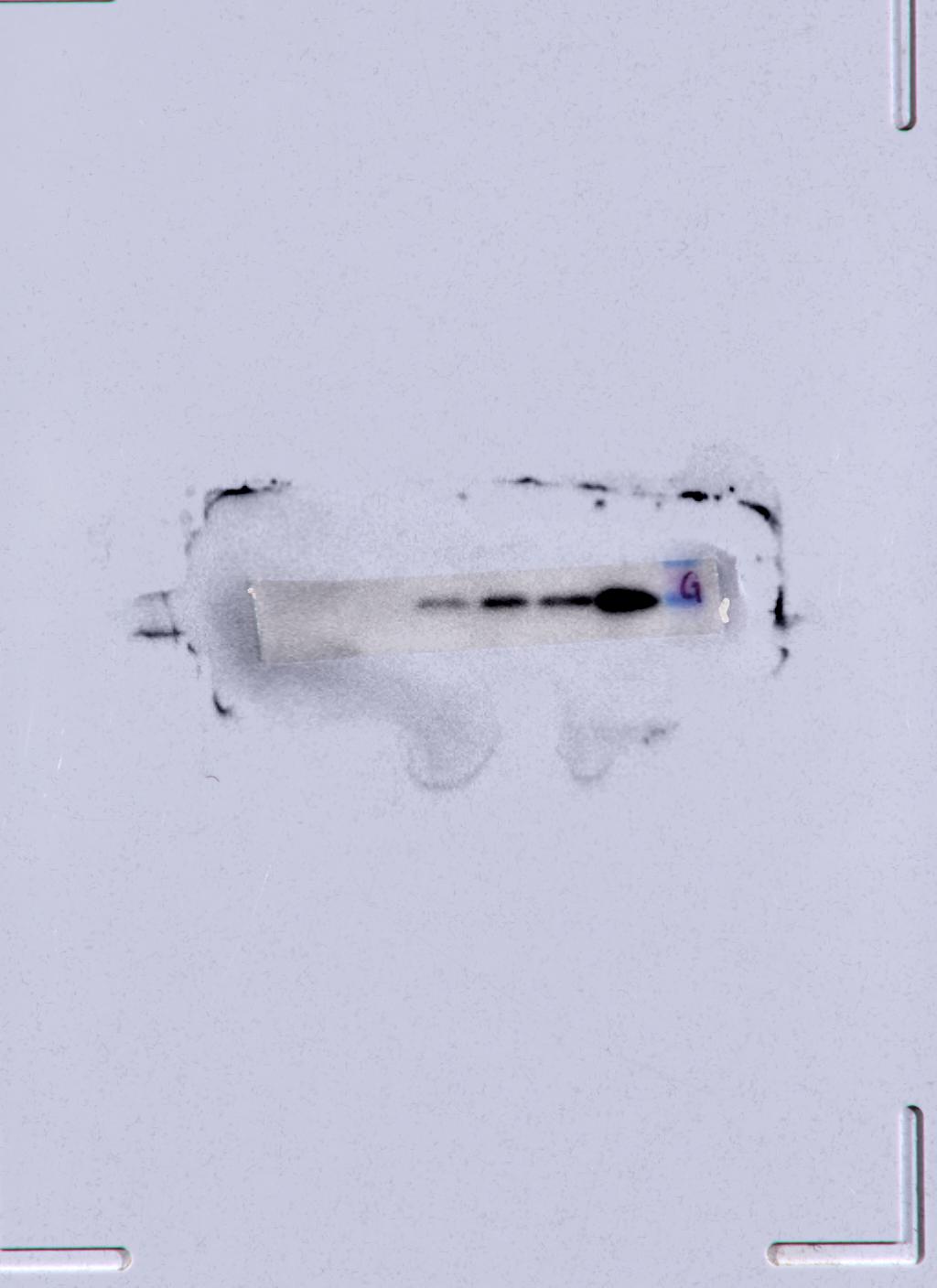

Supplement: Supplemental Information 2 [file peerj-13-19085-s002.zip › Chaetoglobosin A induces T-24 apoptosis in human bladder cancer/6.apoptosis protein/BAX/22.5.3 BAX 2022.05.03_14.04.04_Ch/22.5.3 BAX 2022.05.03_14.04.04_Ch+Marker.jpg]

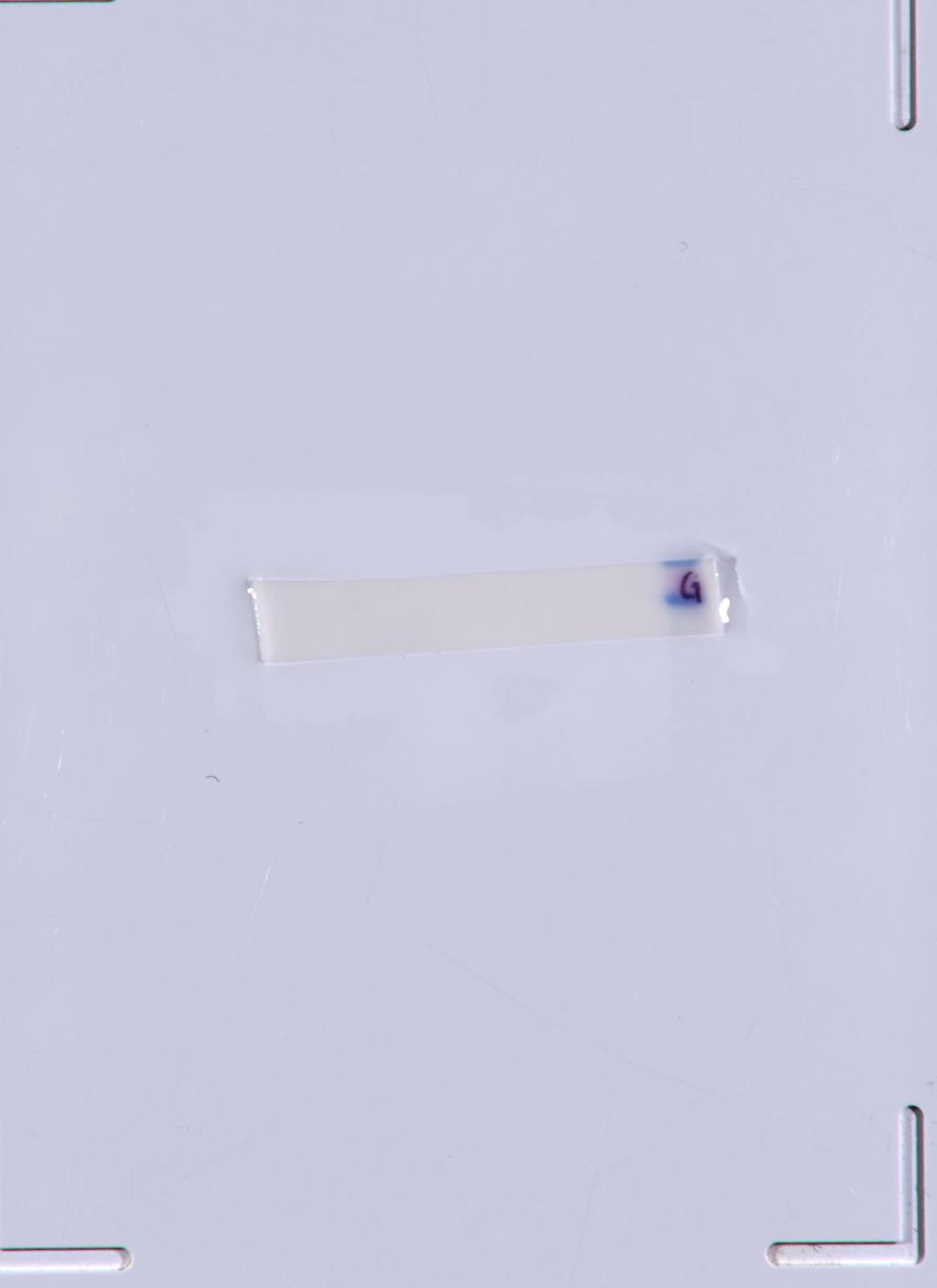

Supplement: Supplemental Information 2 [file peerj-13-19085-s002.zip › Chaetoglobosin A induces T-24 apoptosis in human bladder cancer/6.apoptosis protein/BAX/22.5.3 BAX 2022.05.03_14.04.04_Ch/22.5.3 BAX 2022.05.03_14.04.04_Ch-Marker.jpg]

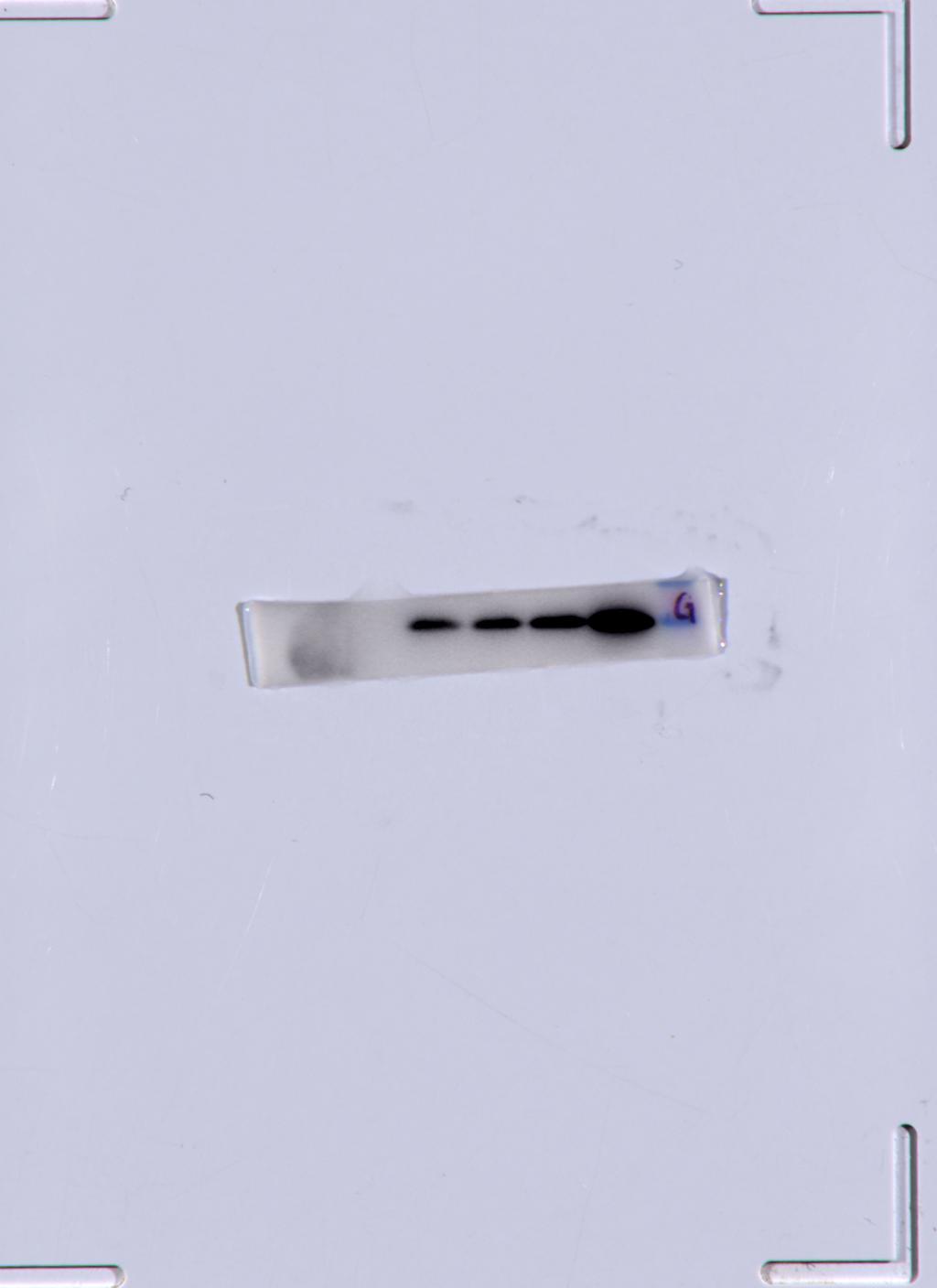

Supplement: Supplemental Information 2 [file peerj-13-19085-s002.zip › Chaetoglobosin A induces T-24 apoptosis in human bladder cancer/6.apoptosis protein/BAX/22.5.3 BAX2 2022.05.03_14.10.19_Ch/22.5.3 BAX2 2022.05.03_14.10.19_Ch+Marker.jpg]

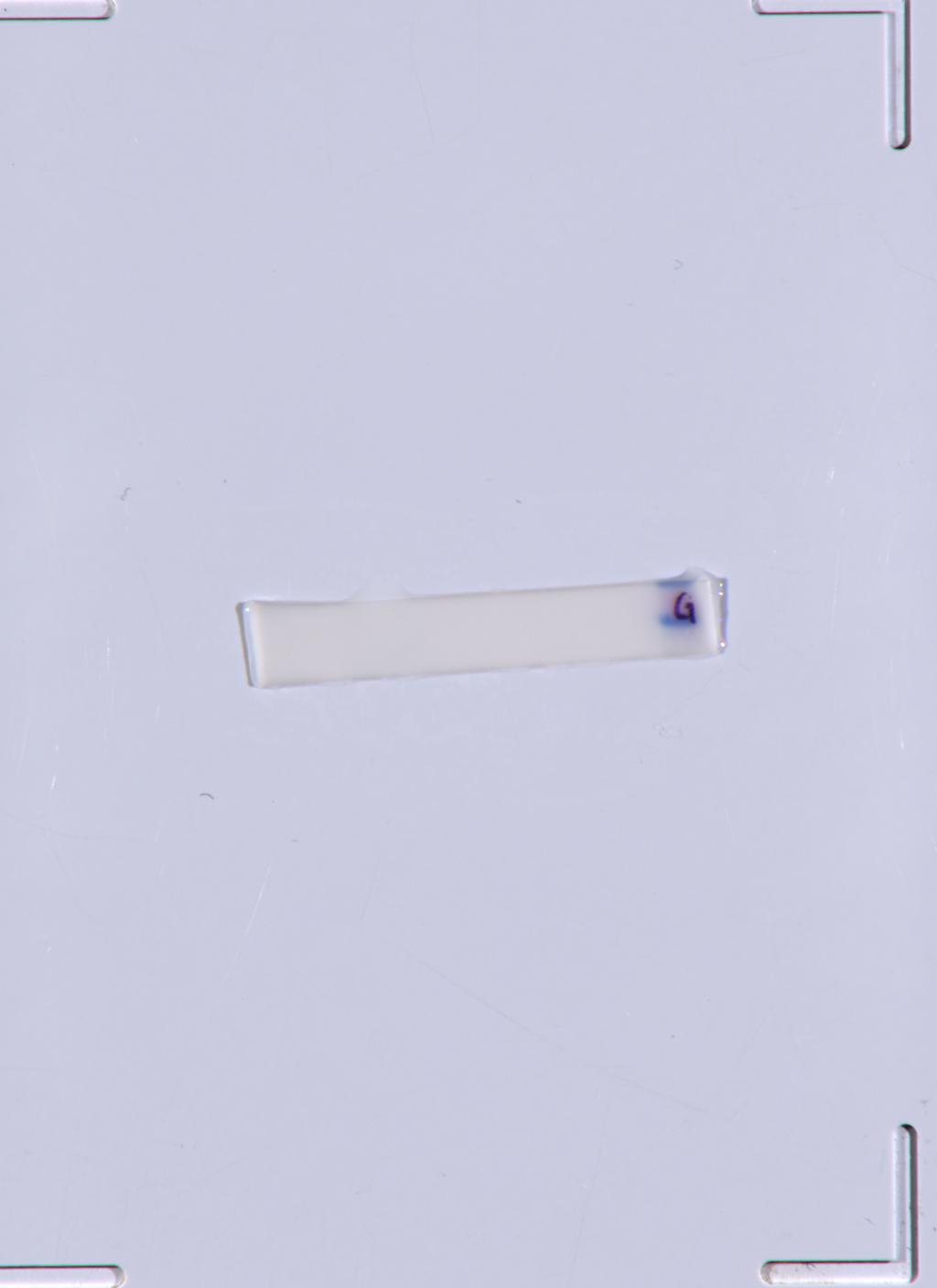

Supplement: Supplemental Information 2 [file peerj-13-19085-s002.zip › Chaetoglobosin A induces T-24 apoptosis in human bladder cancer/6.apoptosis protein/BAX/22.5.3 BAX2 2022.05.03_14.10.19_Ch/22.5.3 BAX2 2022.05.03_14.10.19_Ch-Marker.jpg]

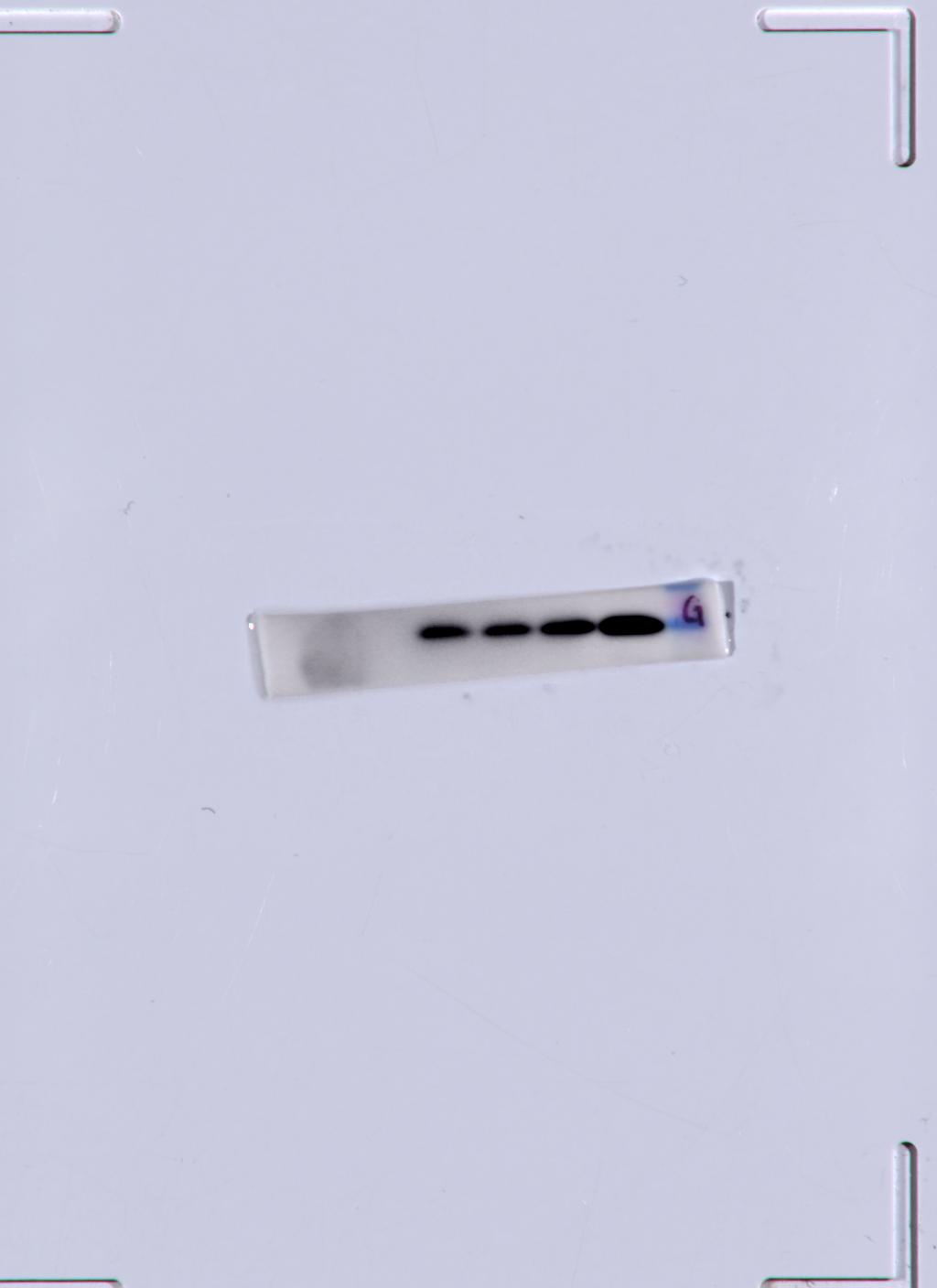

Supplement: Supplemental Information 2 [file peerj-13-19085-s002.zip › Chaetoglobosin A induces T-24 apoptosis in human bladder cancer/6.apoptosis protein/BAX/22.5.3 BAX3 2022.05.03_14.12.25_Ch/22.5.3 BAX3 2022.05.03_14.12.25_Ch+Marker.jpg]

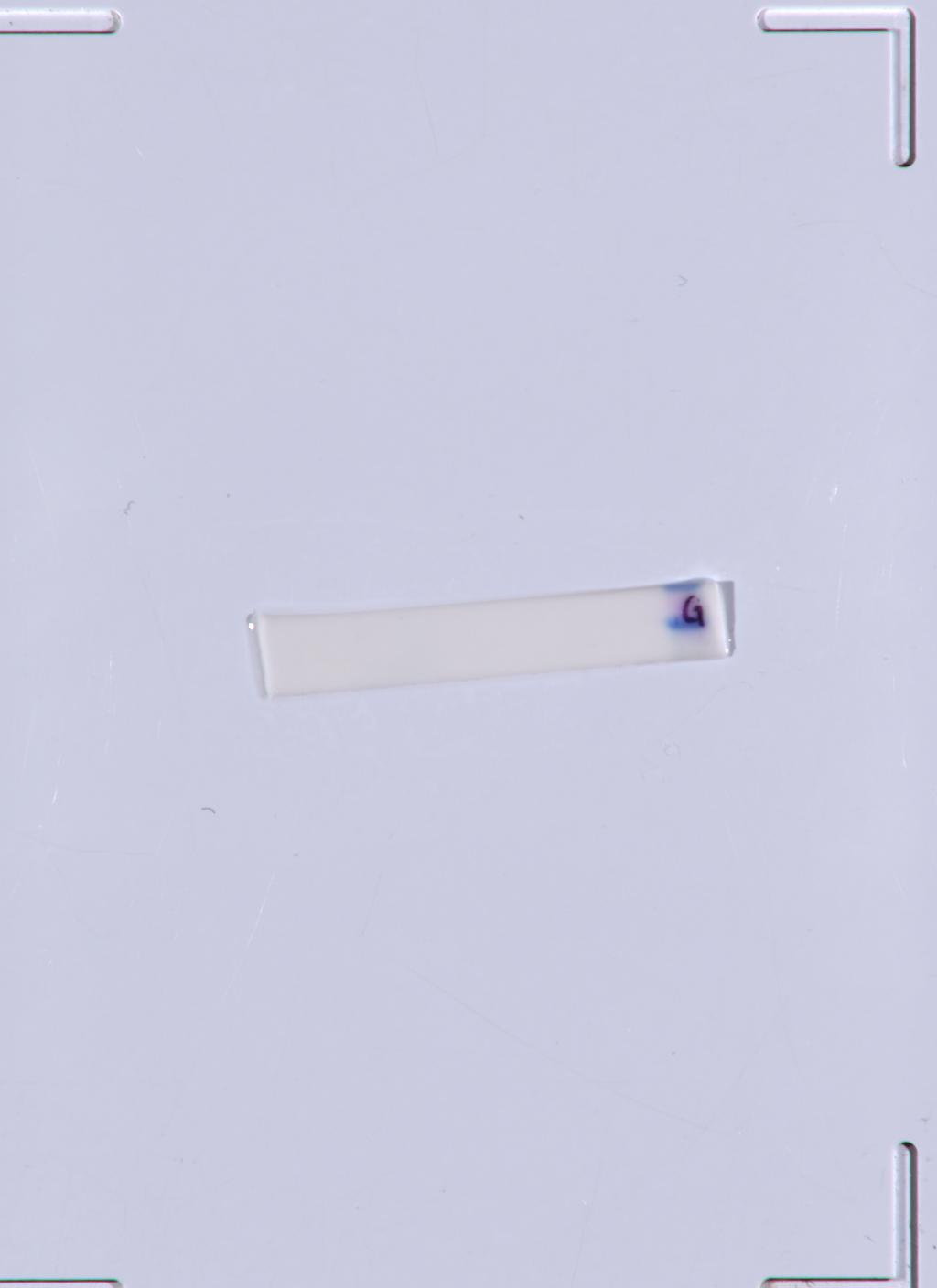

Supplement: Supplemental Information 2 [file peerj-13-19085-s002.zip › Chaetoglobosin A induces T-24 apoptosis in human bladder cancer/6.apoptosis protein/BAX/22.5.3 BAX3 2022.05.03_14.12.25_Ch/22.5.3 BAX3 2022.05.03_14.12.25_Ch-Marker.jpg]

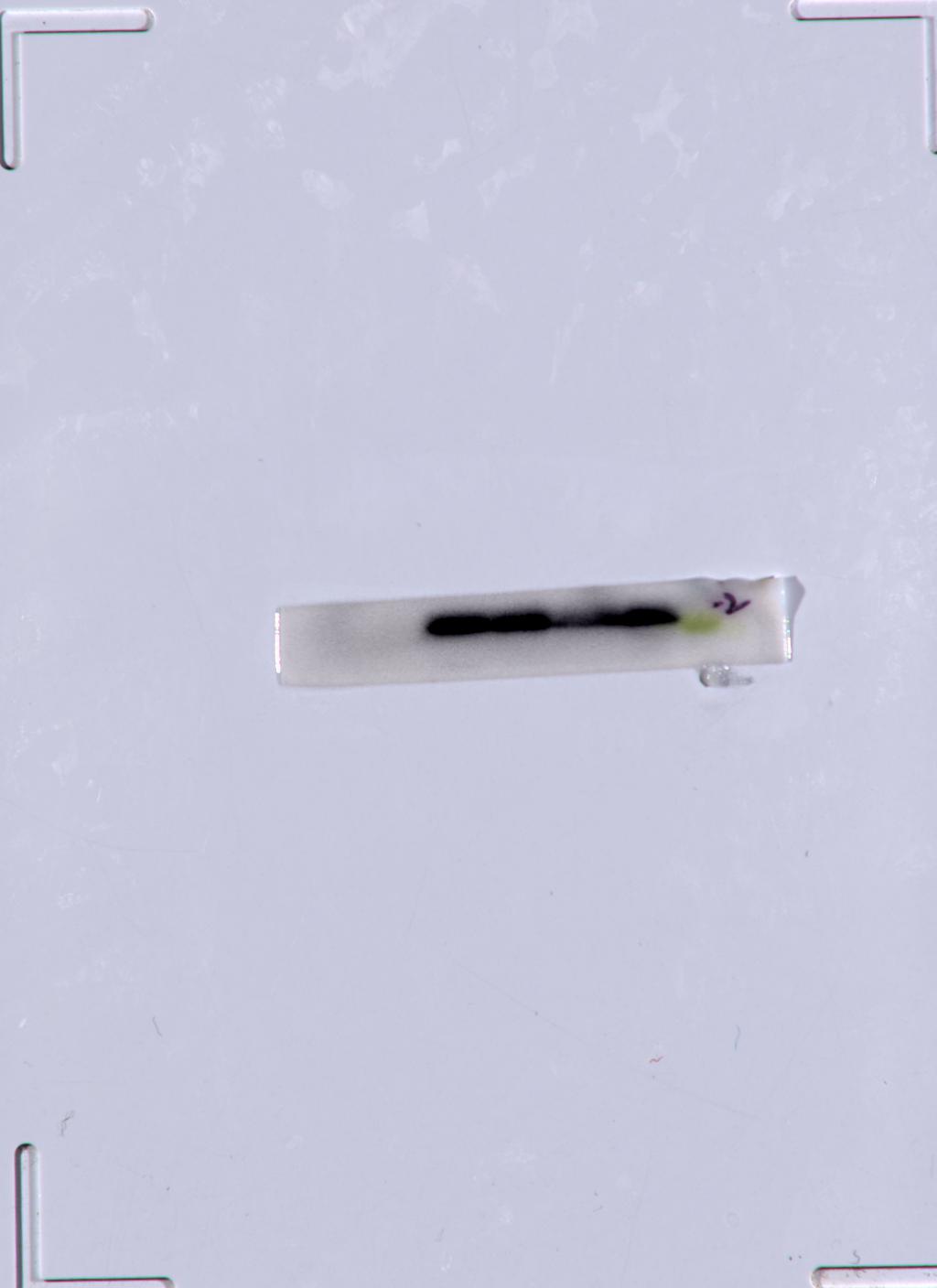

Supplement: Supplemental Information 2 [file peerj-13-19085-s002.zip › Chaetoglobosin A induces T-24 apoptosis in human bladder cancer/6.apoptosis protein/BCL-2/22.4.28bcl-2 2 2022.04.28_13.49.48_Ch+Marker.jpg]

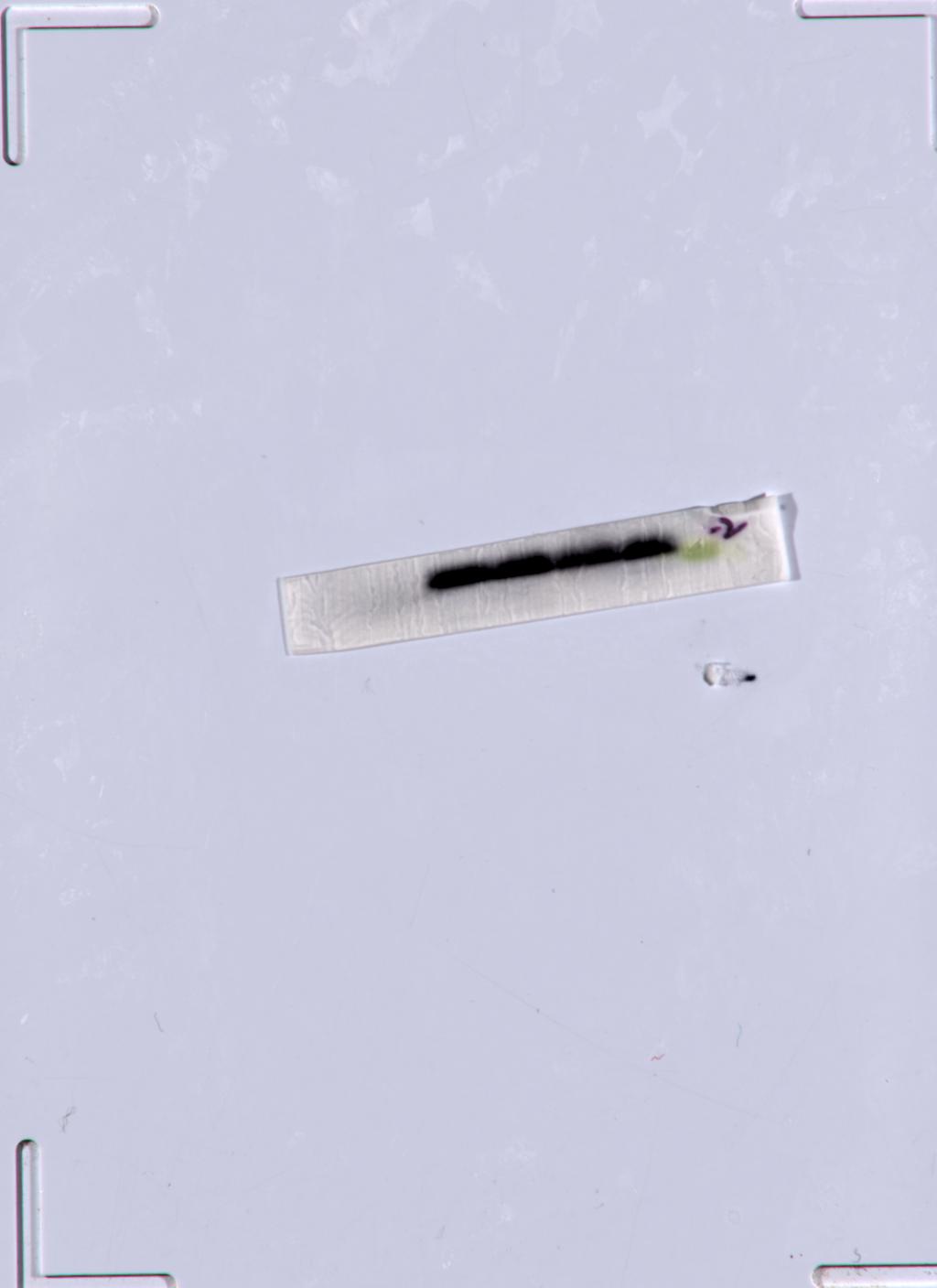

Supplement: Supplemental Information 2 [file peerj-13-19085-s002.zip › Chaetoglobosin A induces T-24 apoptosis in human bladder cancer/6.apoptosis protein/BCL-2/22.4.28bcl-211 2022.04.28_14.04.31_Ch+Marker.jpg]

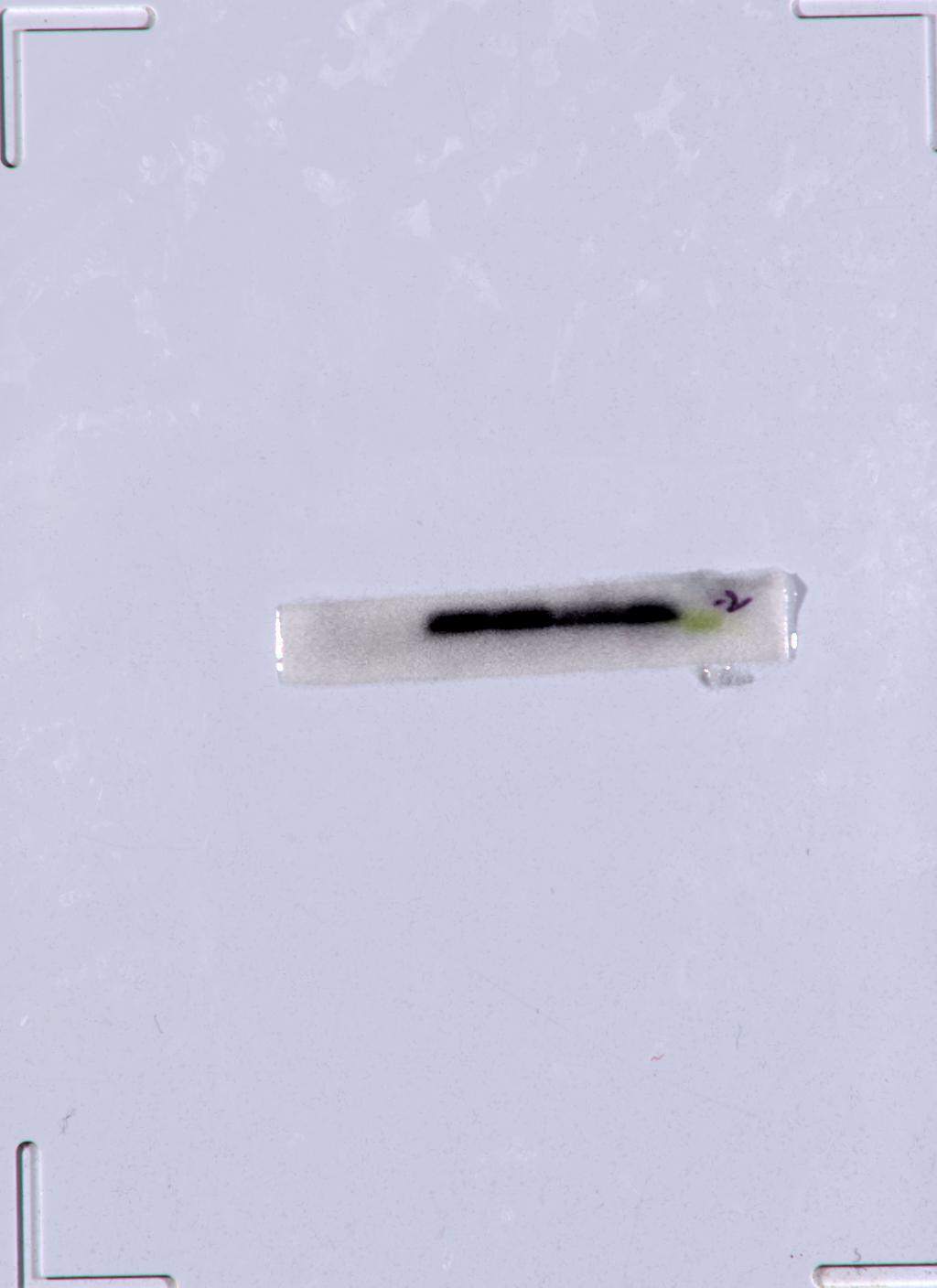

Supplement: Supplemental Information 2 [file peerj-13-19085-s002.zip › Chaetoglobosin A induces T-24 apoptosis in human bladder cancer/6.apoptosis protein/BCL-2/22.4.28bcl-26 2022.04.28_13.56.41_Ch+Marker.jpg]

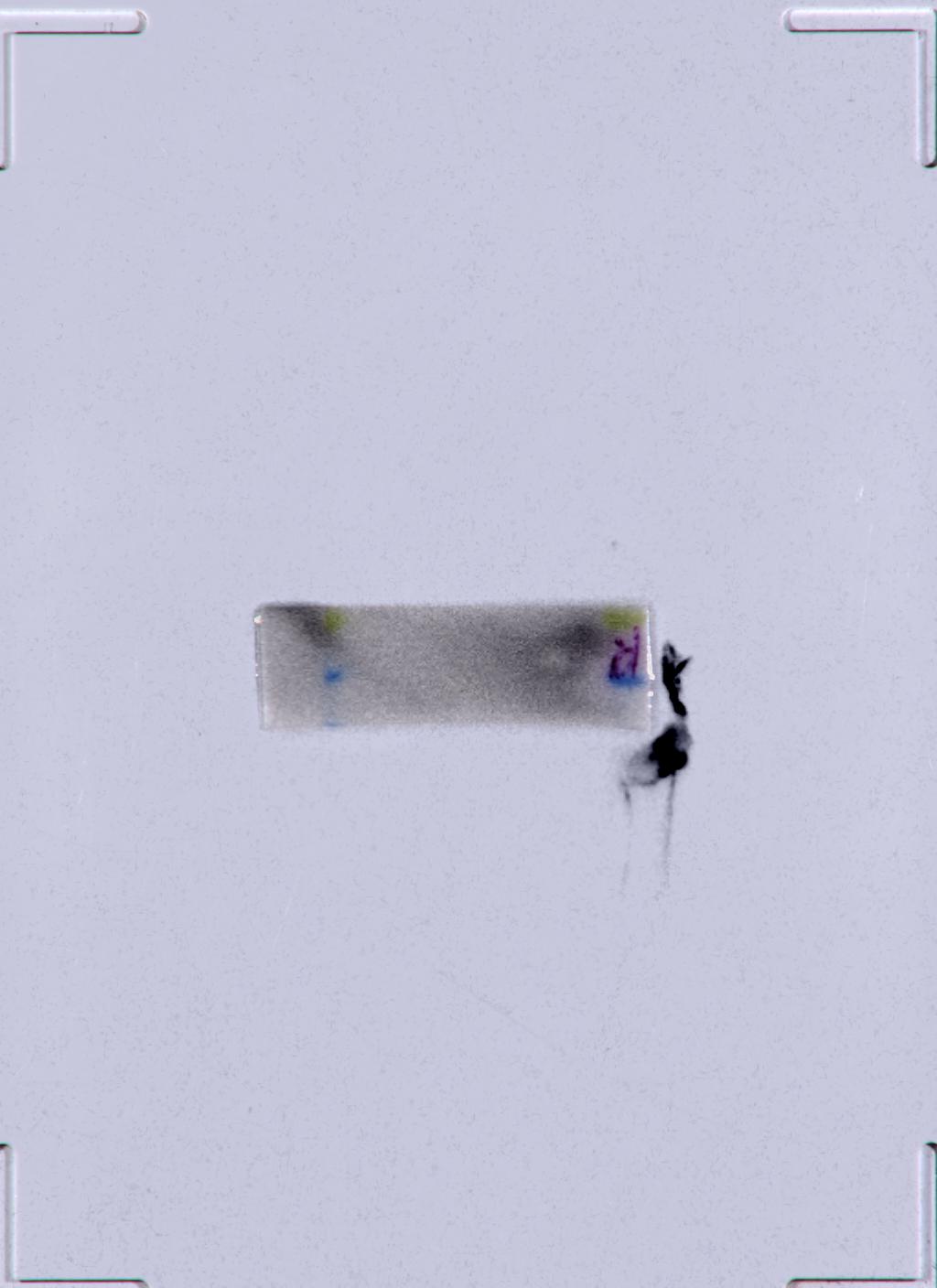

Supplement: Supplemental Information 2 [file peerj-13-19085-s002.zip › Chaetoglobosin A induces T-24 apoptosis in human bladder cancer/6.apoptosis protein/BID/22.5.7bid 2022.05.07_14.26.13_Ch/22.5.7bid 2022.05.07_14.26.13_Ch+Marker.jpg]

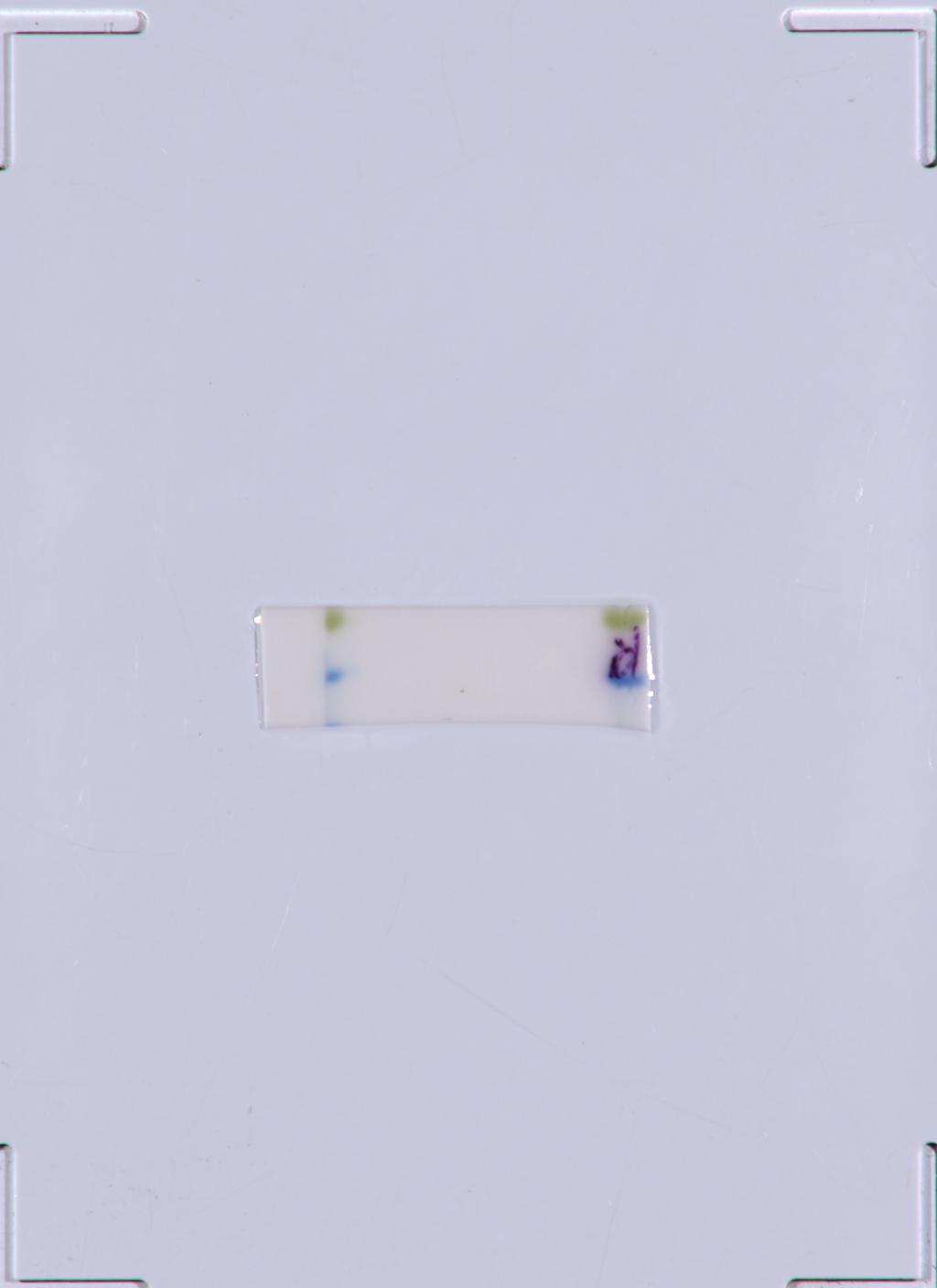

Supplement: Supplemental Information 2 [file peerj-13-19085-s002.zip › Chaetoglobosin A induces T-24 apoptosis in human bladder cancer/6.apoptosis protein/BID/22.5.7bid 2022.05.07_14.26.13_Ch/22.5.7bid 2022.05.07_14.26.13_Ch-Marker.jpg]

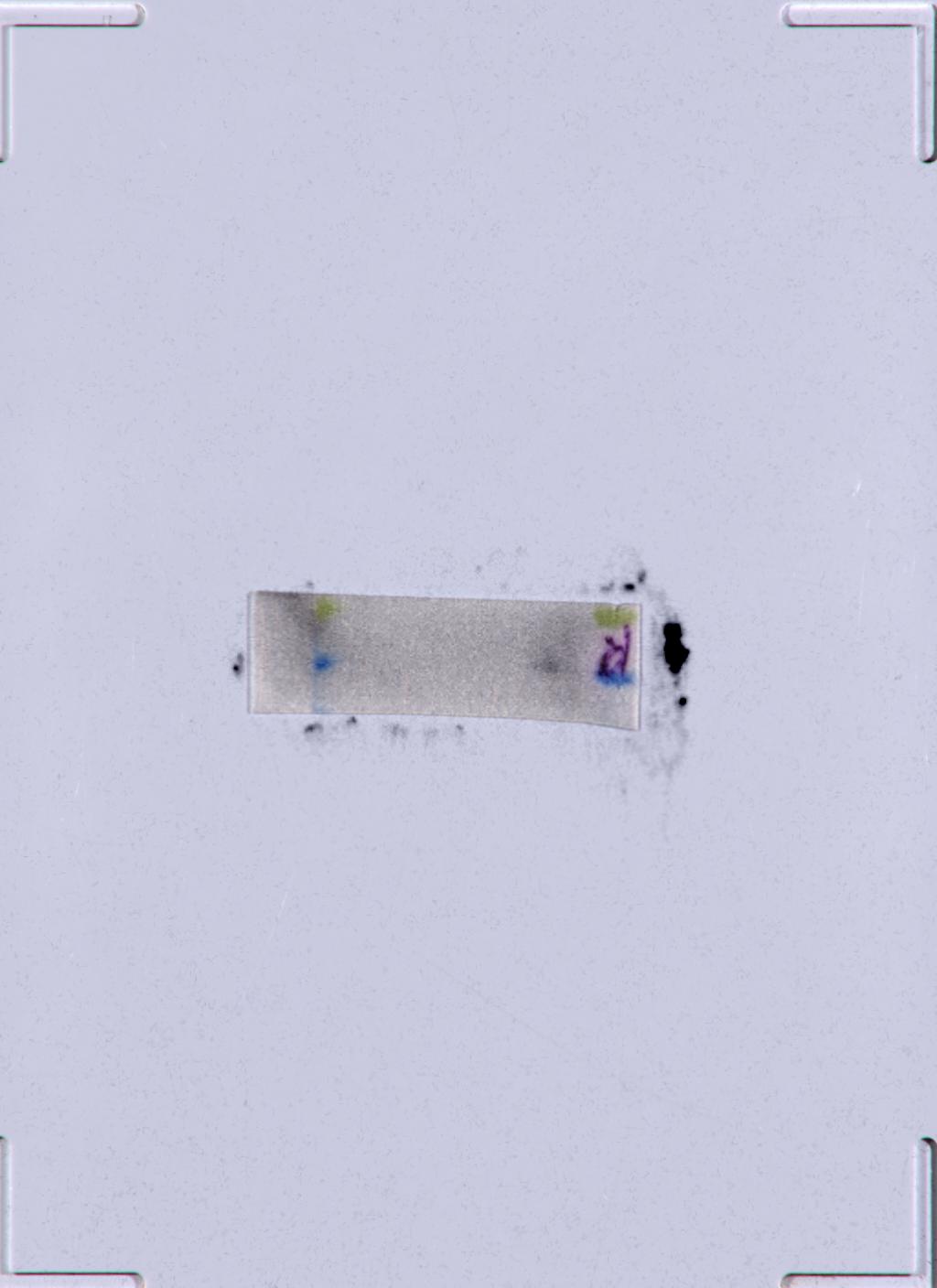

Supplement: Supplemental Information 2 [file peerj-13-19085-s002.zip › Chaetoglobosin A induces T-24 apoptosis in human bladder cancer/6.apoptosis protein/BID/22.5.7bid-2 2022.05.07_14.32.24_Ch/22.5.7bid-2 2022.05.07_14.32.24_Ch+Marker.jpg]

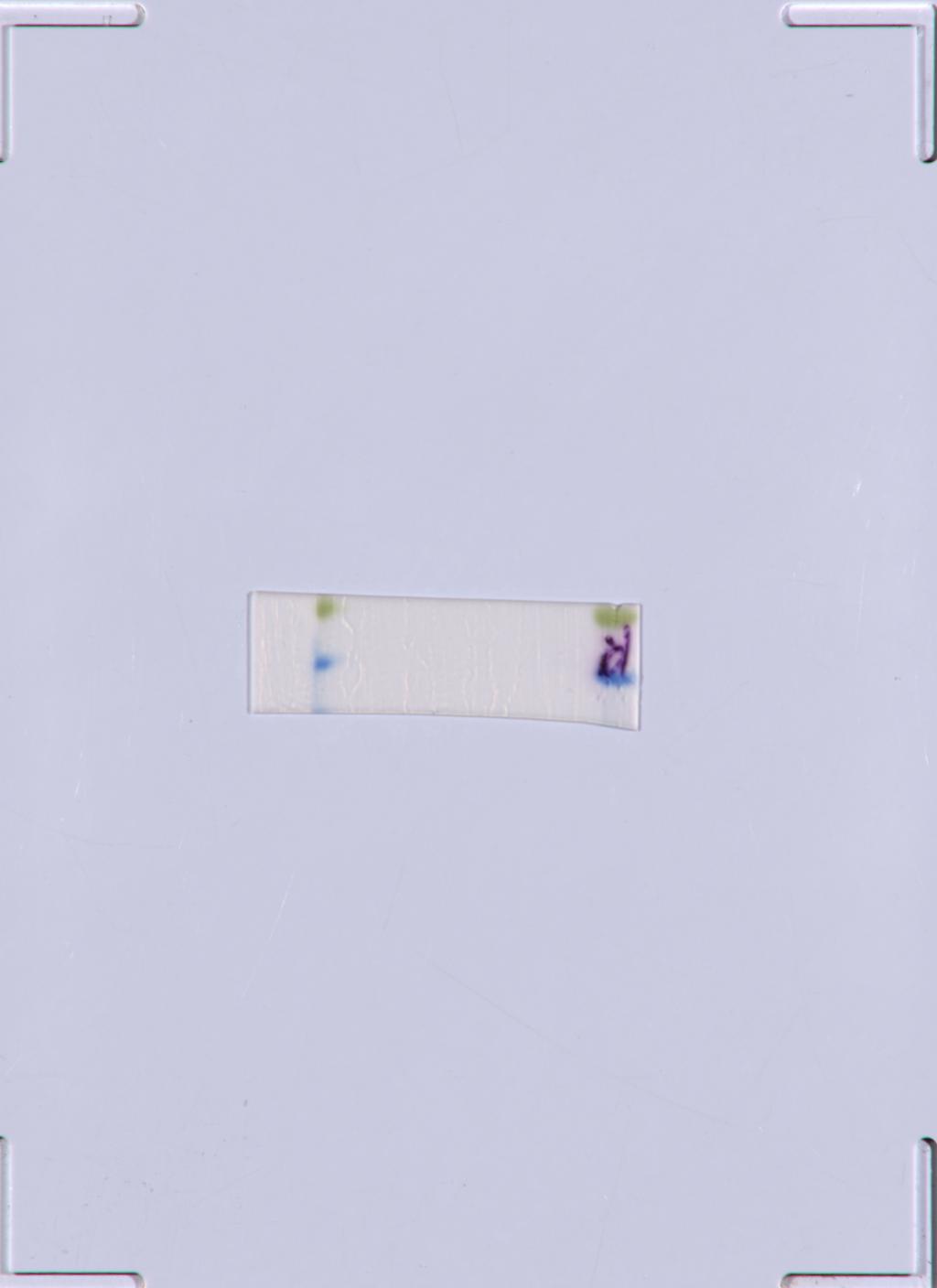

Supplement: Supplemental Information 2 [file peerj-13-19085-s002.zip › Chaetoglobosin A induces T-24 apoptosis in human bladder cancer/6.apoptosis protein/BID/22.5.7bid-2 2022.05.07_14.32.24_Ch/22.5.7bid-2 2022.05.07_14.32.24_Ch-Marker.jpg]

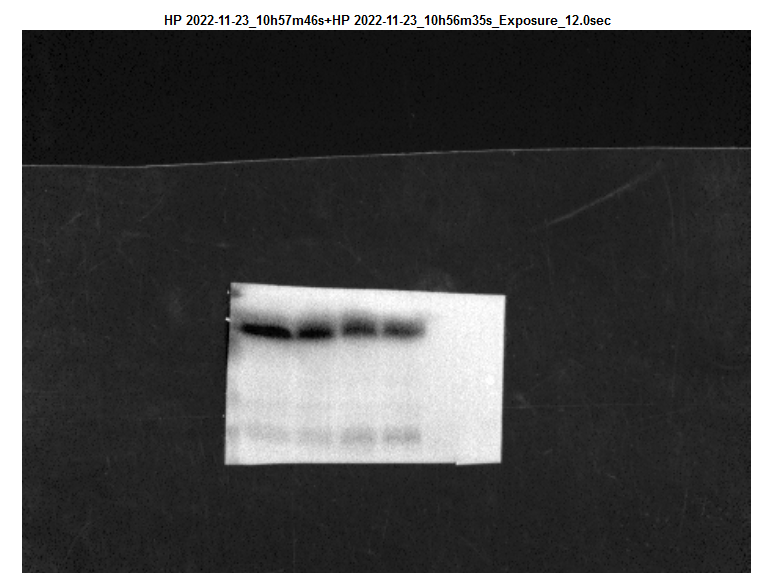

Supplement: Supplemental Information 2 [file peerj-13-19085-s002.zip › Chaetoglobosin A induces T-24 apoptosis in human bladder cancer/6.apoptosis protein/caspase3/HP 2022-11-23_10h57m46s+HP 2022-11-23_10h56m35s_Exposure_12.0sec.tif]

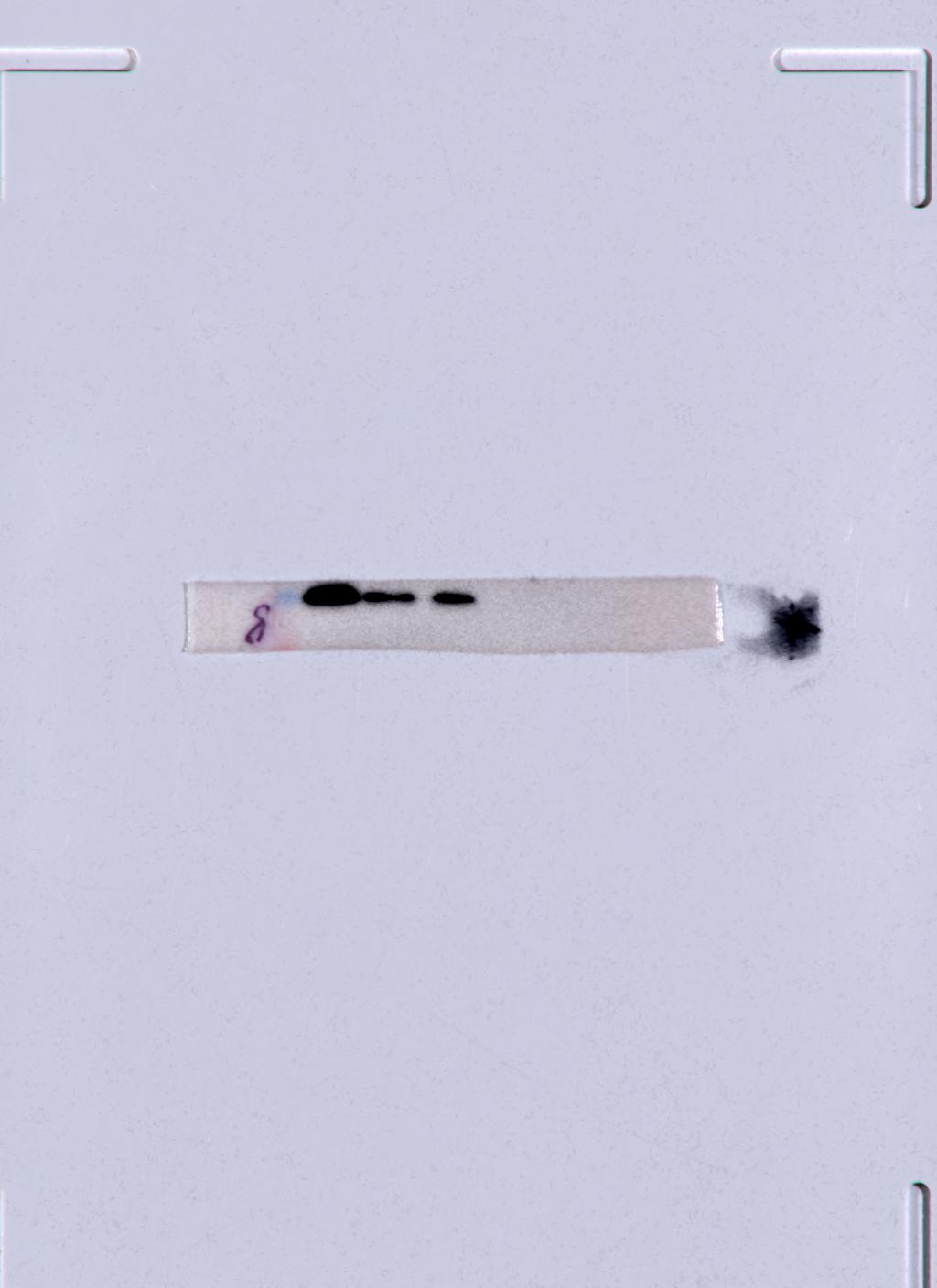

Supplement: Supplemental Information 2 [file peerj-13-19085-s002.zip › Chaetoglobosin A induces T-24 apoptosis in human bladder cancer/6.apoptosis protein/caspase8/22.4.18 caspase8 2022.04.18_14.14.40_Ch+Marker.jpg]

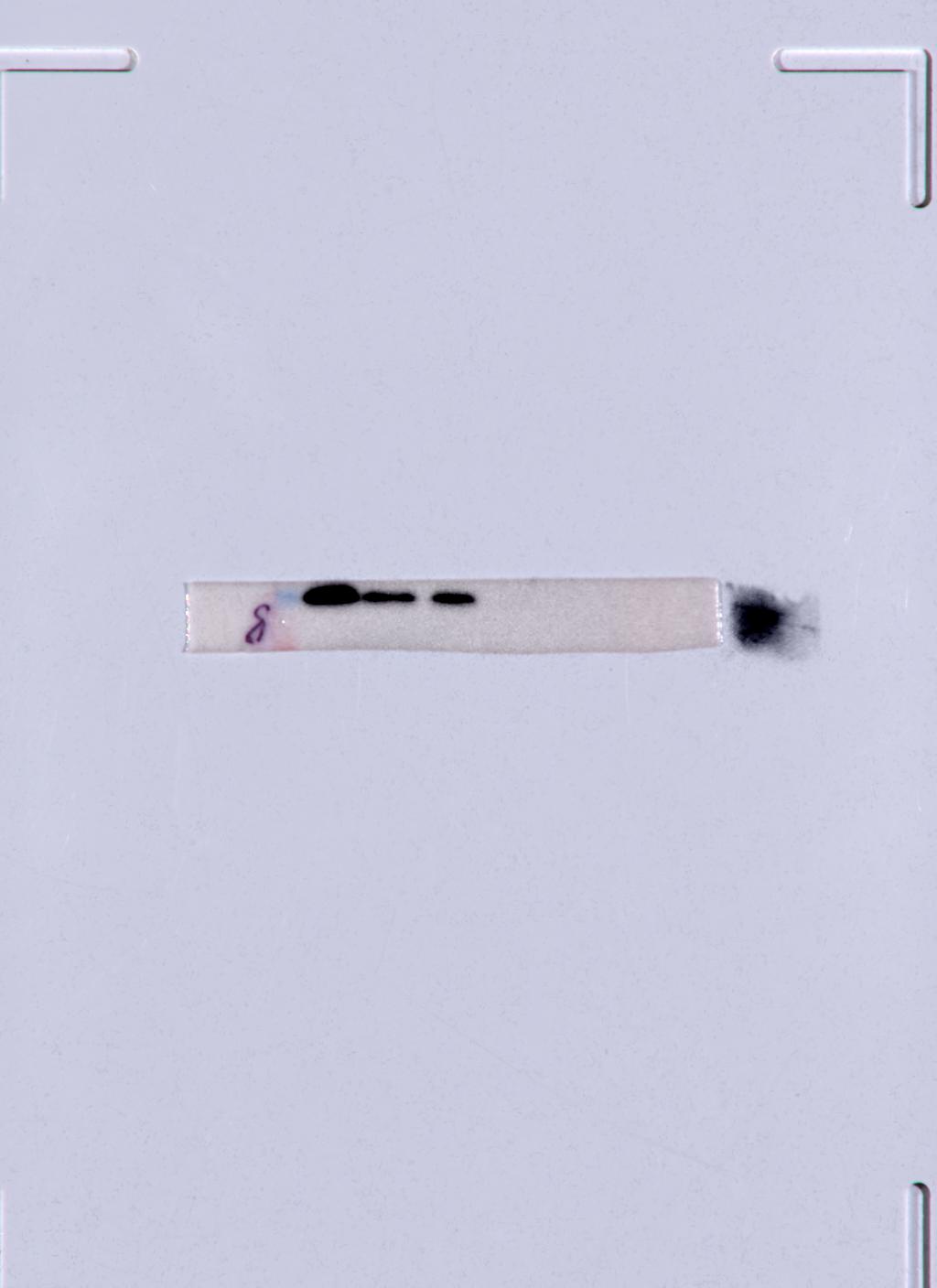

Supplement: Supplemental Information 2 [file peerj-13-19085-s002.zip › Chaetoglobosin A induces T-24 apoptosis in human bladder cancer/6.apoptosis protein/caspase8/22.4.18 caspase8.2 2022.04.18_14.17.18_Ch+Marker.jpg]

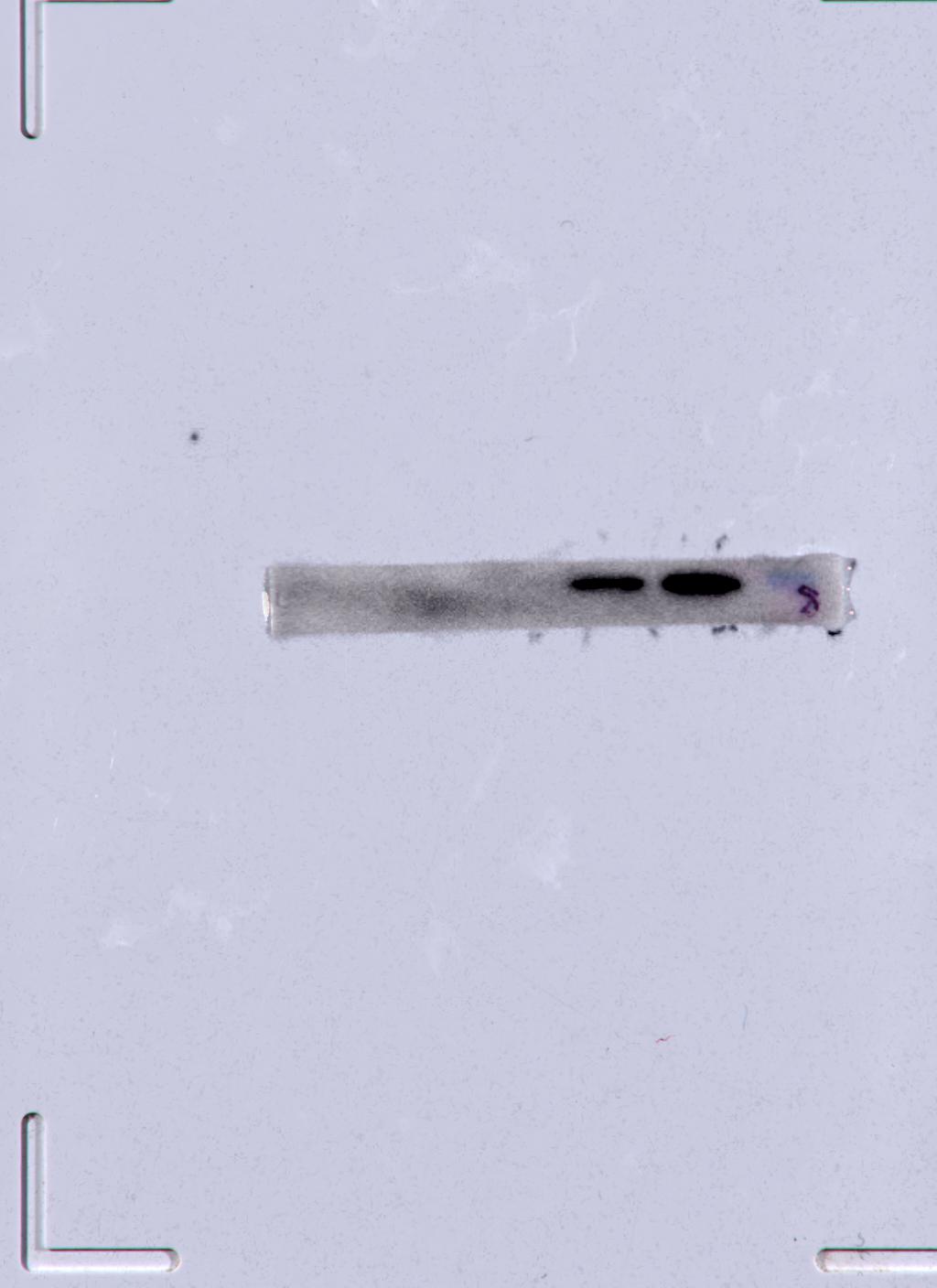

Supplement: Supplemental Information 2 [file peerj-13-19085-s002.zip › Chaetoglobosin A induces T-24 apoptosis in human bladder cancer/6.apoptosis protein/caspase8/22.4.27 c8-5 2022.04.27_13.41.26_Ch+Marker.jpg]

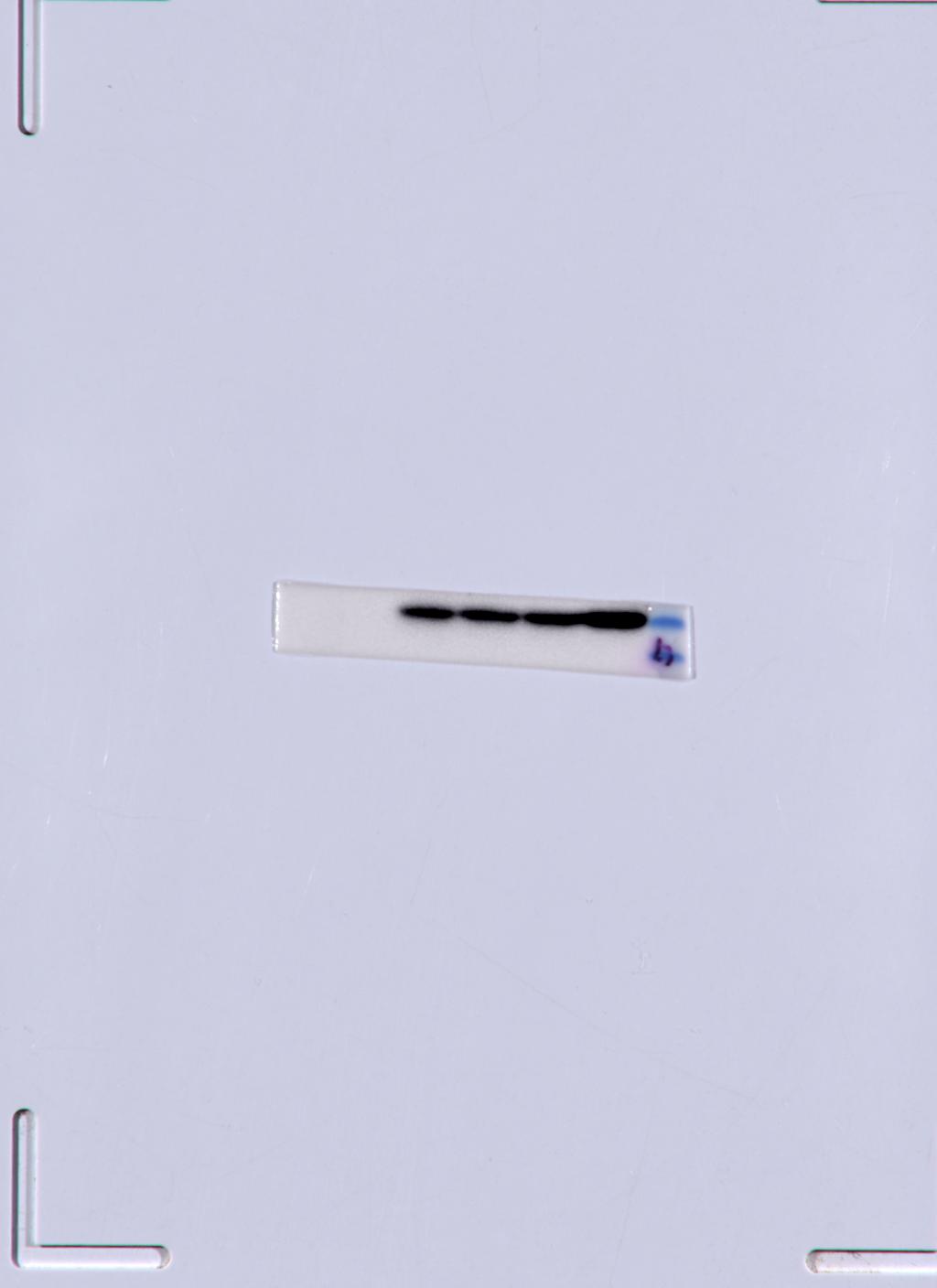

Supplement: Supplemental Information 2 [file peerj-13-19085-s002.zip › Chaetoglobosin A induces T-24 apoptosis in human bladder cancer/6.apoptosis protein/GAPDH/22.5.4 gapdh 2022.05.04_13.19.02_Ch+Marker.jpg]

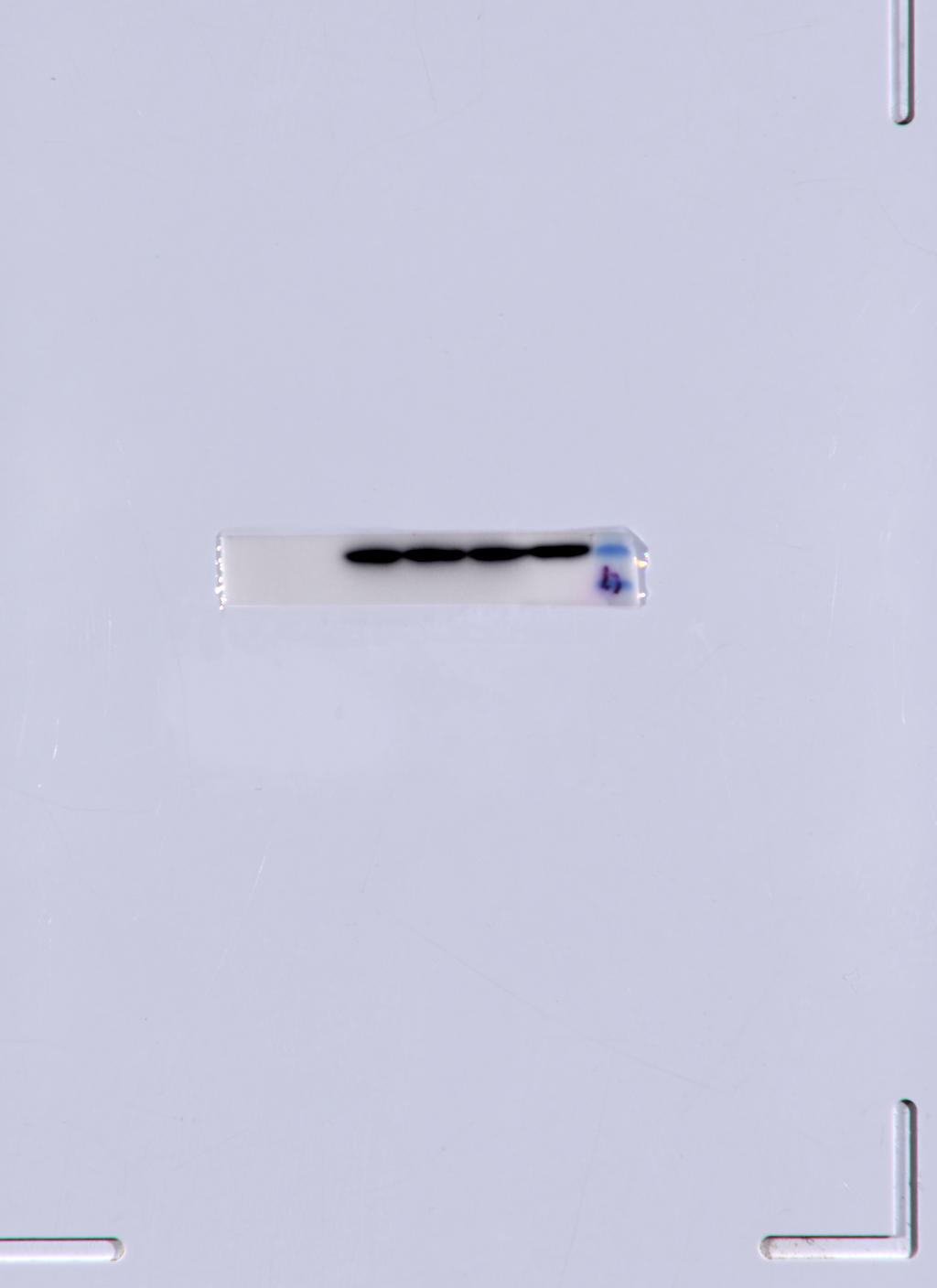

Supplement: Supplemental Information 2 [file peerj-13-19085-s002.zip › Chaetoglobosin A induces T-24 apoptosis in human bladder cancer/6.apoptosis protein/GAPDH/22.5.4gapdh11 2022.05.04_14.08.56_Ch+Marker.jpg]

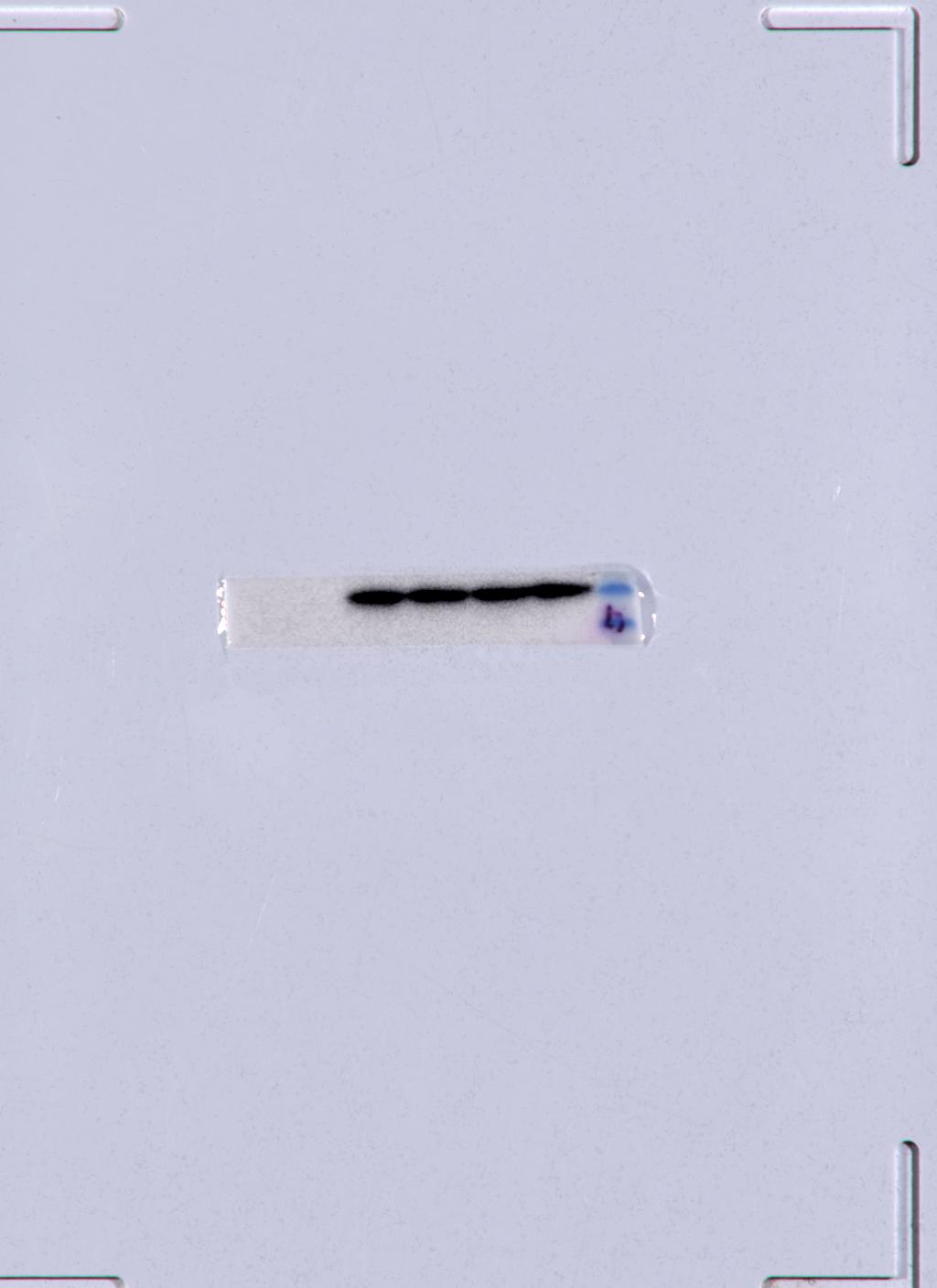

Supplement: Supplemental Information 2 [file peerj-13-19085-s002.zip › Chaetoglobosin A induces T-24 apoptosis in human bladder cancer/6.apoptosis protein/GAPDH/22.5.4gapdh15 2022.05.04_14.14.38_Ch+Marker.jpg]

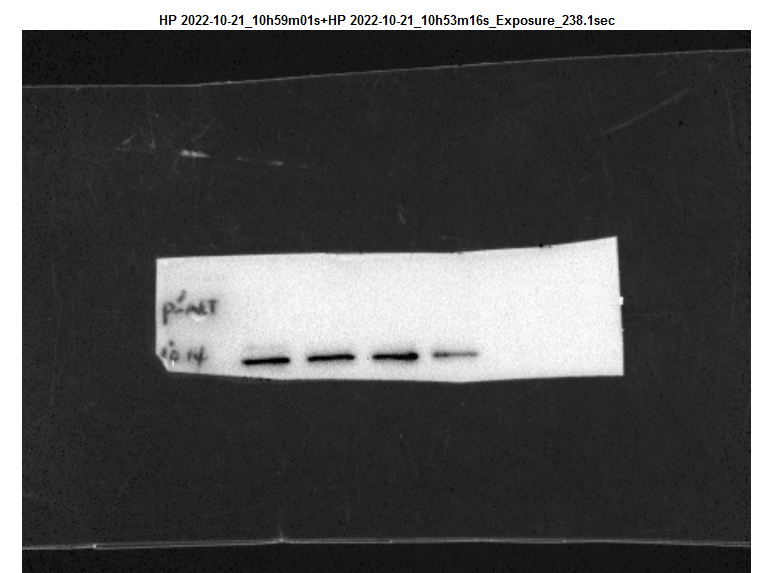

Supplement: Supplemental Information 2 [file peerj-13-19085-s002.zip › Chaetoglobosin A induces T-24 apoptosis in human bladder cancer/7.PI3K ERK pathway/AKT/HP 2022-10-21_10h59m01s+HP 2022-10-21_10h53m16s_Exposure_238.1sec.tif]

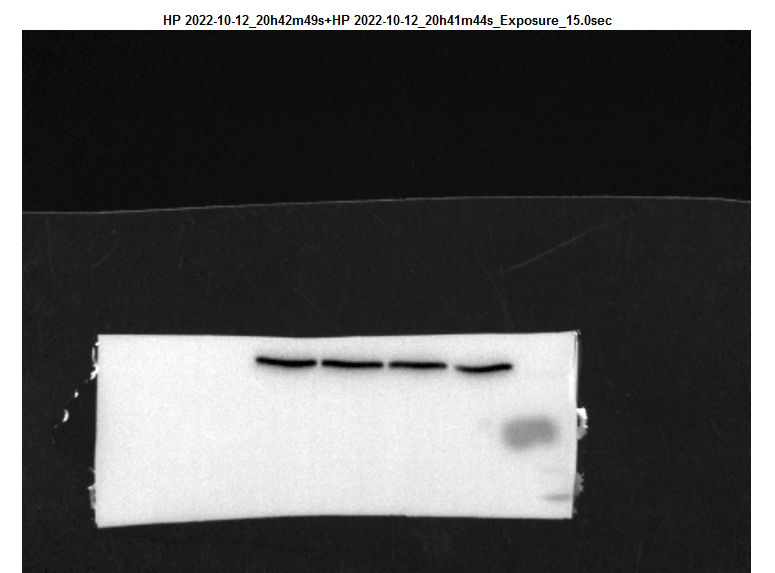

Supplement: Supplemental Information 2 [file peerj-13-19085-s002.zip › Chaetoglobosin A induces T-24 apoptosis in human bladder cancer/7.PI3K ERK pathway/Calnexin/HP 2022-10-12_20h42m49s+HP 2022-10-12_20h41m44s_Exposure_15.0sec.tif]

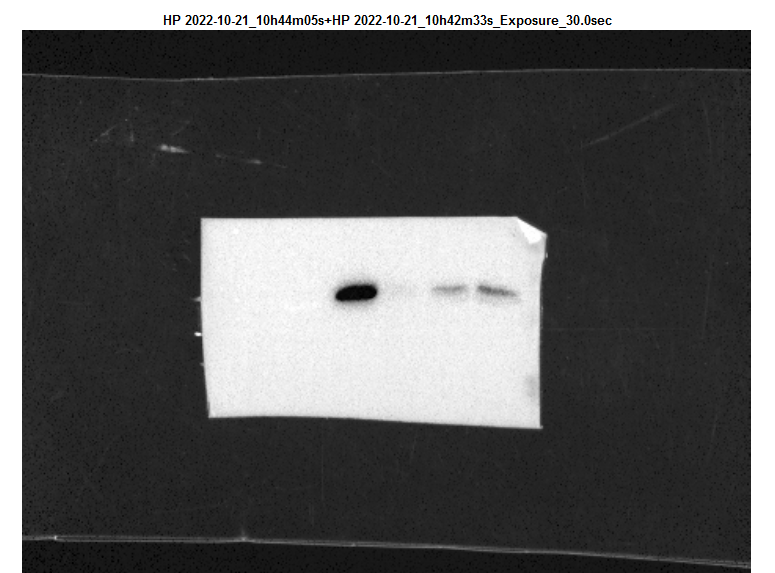

Supplement: Supplemental Information 2 [file peerj-13-19085-s002.zip › Chaetoglobosin A induces T-24 apoptosis in human bladder cancer/7.PI3K ERK pathway/erk/HP 2022-10-21_10h44m05s+HP 2022-10-21_10h42m33s_Exposure_30.0sec.tif]

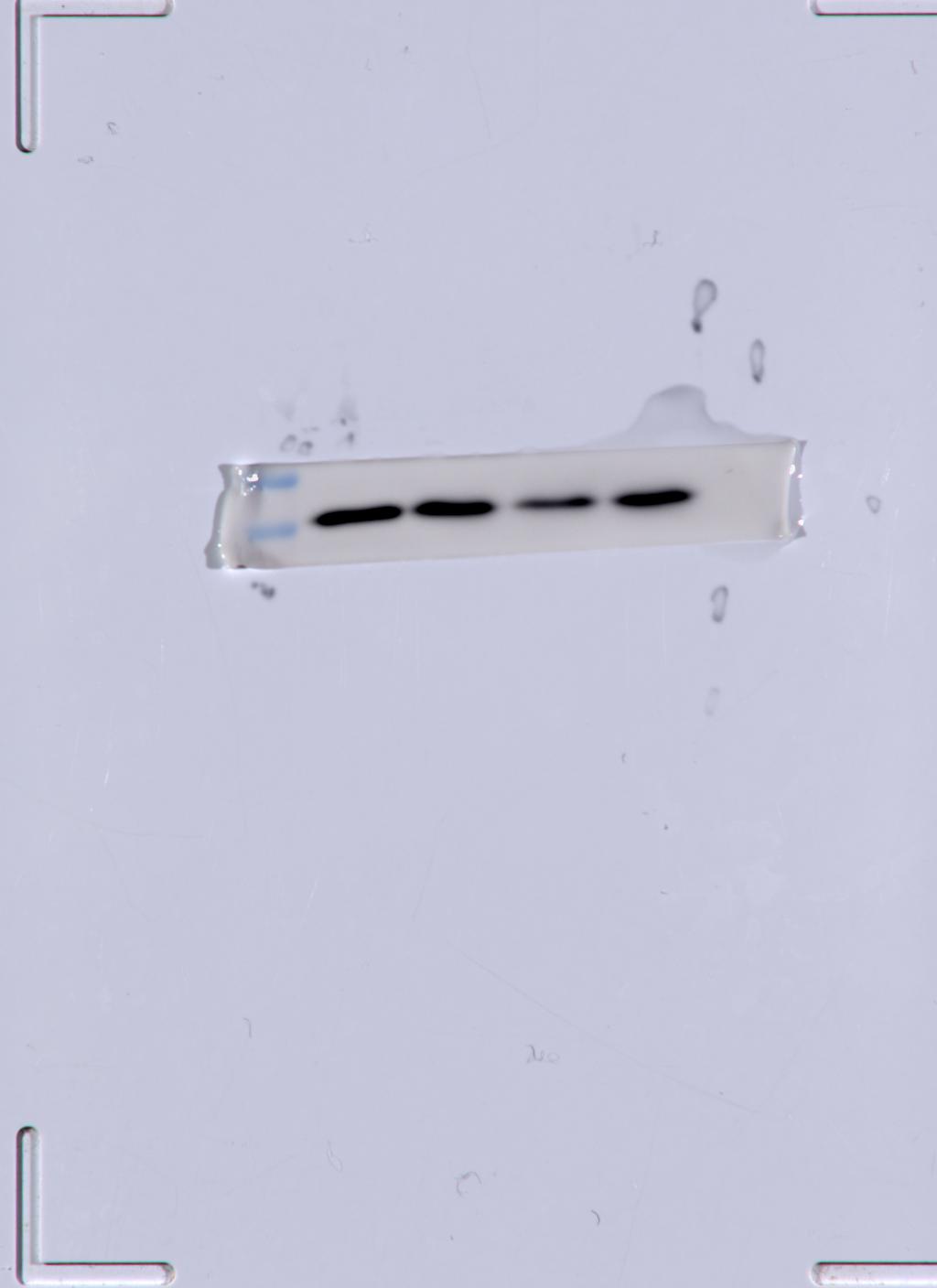

Supplement: Supplemental Information 2 [file peerj-13-19085-s002.zip › Chaetoglobosin A induces T-24 apoptosis in human bladder cancer/7.PI3K ERK pathway/GAPDH/1.11 GAPDH-1 2022.01.11_19.32.49_Ch/1.11 GAPDH-1 2022.01.11_19.32.49_Ch+Marker.jpg]

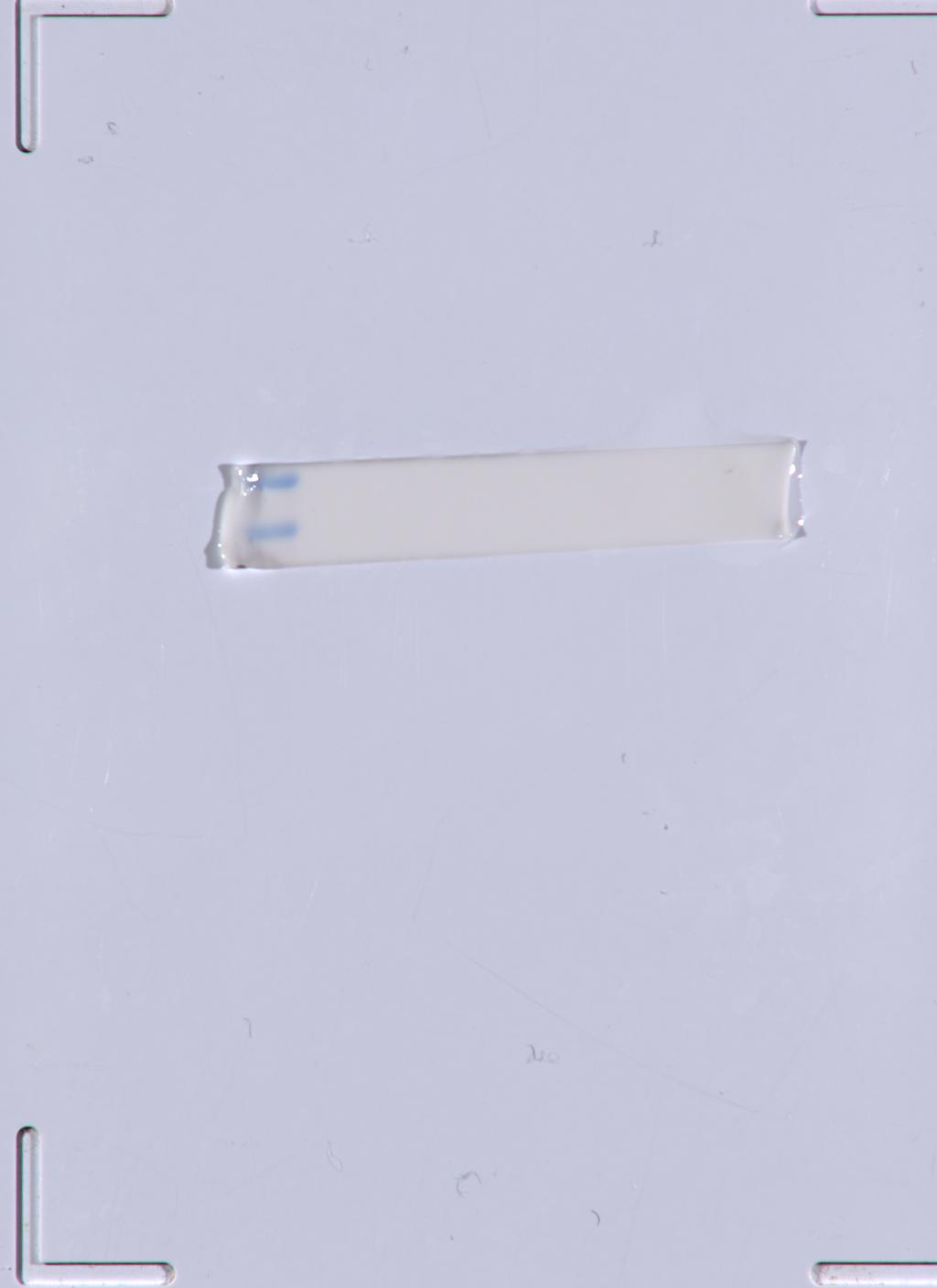

Supplement: Supplemental Information 2 [file peerj-13-19085-s002.zip › Chaetoglobosin A induces T-24 apoptosis in human bladder cancer/7.PI3K ERK pathway/GAPDH/1.11 GAPDH-1 2022.01.11_19.32.49_Ch/1.11 GAPDH-1 2022.01.11_19.32.49_Ch-Marker.jpg]

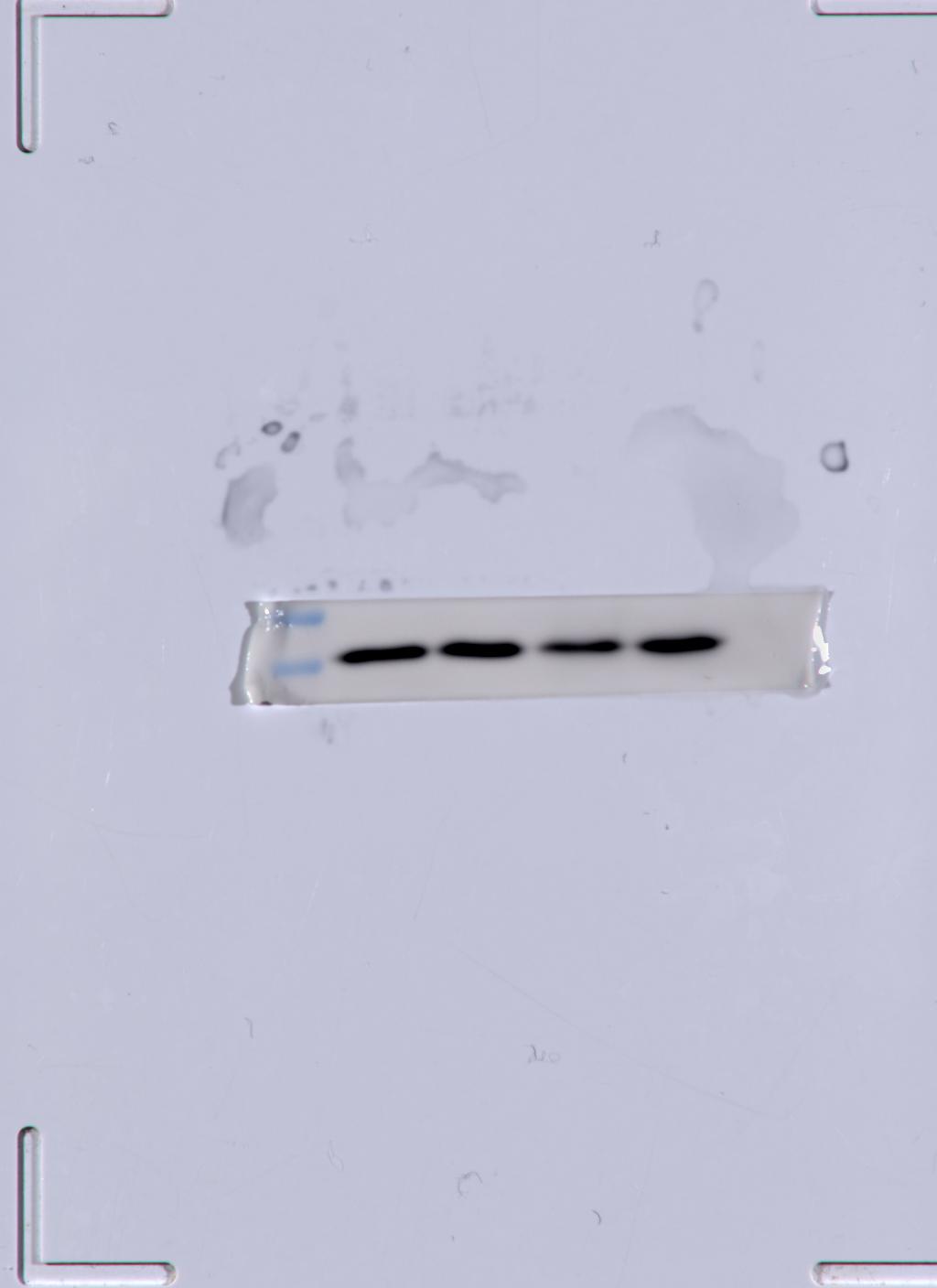

Supplement: Supplemental Information 2 [file peerj-13-19085-s002.zip › Chaetoglobosin A induces T-24 apoptosis in human bladder cancer/7.PI3K ERK pathway/GAPDH/1.11 GAPDH-2 2022.01.11_19.41.09_Ch/1.11 GAPDH-2 2022.01.11_19.41.09_Ch+Marker.jpg]

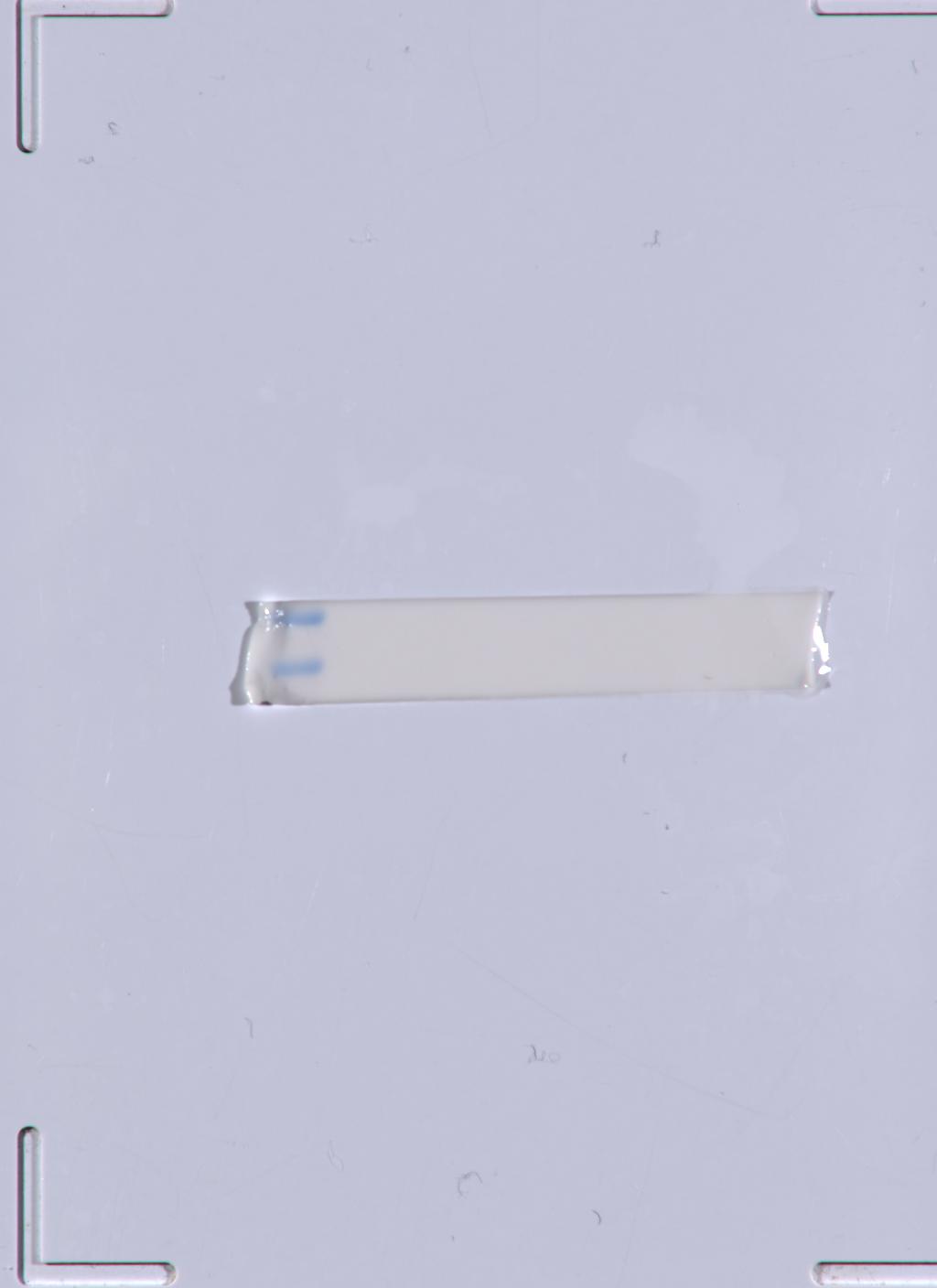

Supplement: Supplemental Information 2 [file peerj-13-19085-s002.zip › Chaetoglobosin A induces T-24 apoptosis in human bladder cancer/7.PI3K ERK pathway/GAPDH/1.11 GAPDH-2 2022.01.11_19.41.09_Ch/1.11 GAPDH-2 2022.01.11_19.41.09_Ch-Marker.jpg]

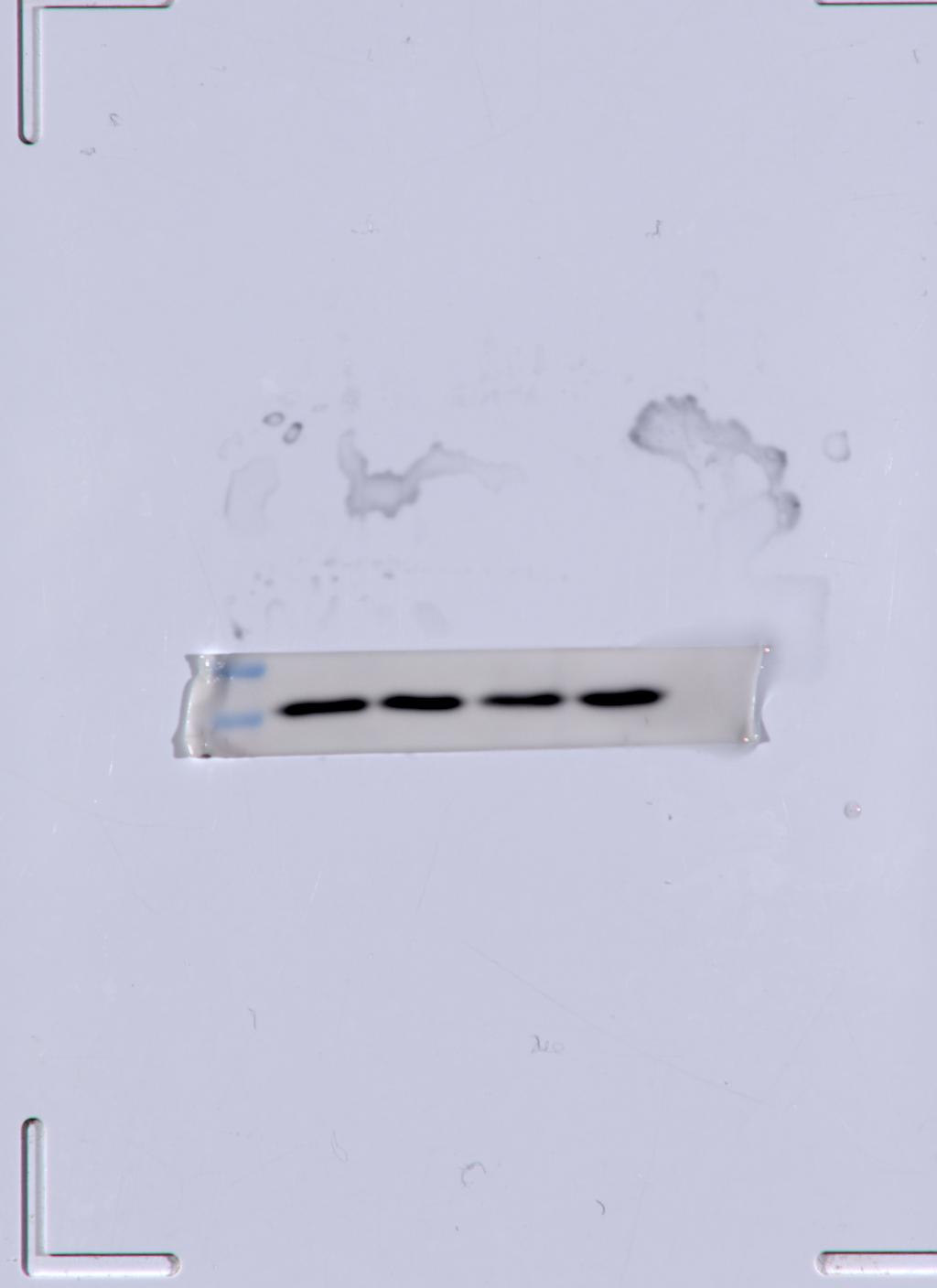

Supplement: Supplemental Information 2 [file peerj-13-19085-s002.zip › Chaetoglobosin A induces T-24 apoptosis in human bladder cancer/7.PI3K ERK pathway/GAPDH/1.11 GAPDH-3 2022.01.11_19.51.03_Ch/1.11 GAPDH-3 2022.01.11_19.51.03_Ch+Marker.jpg]

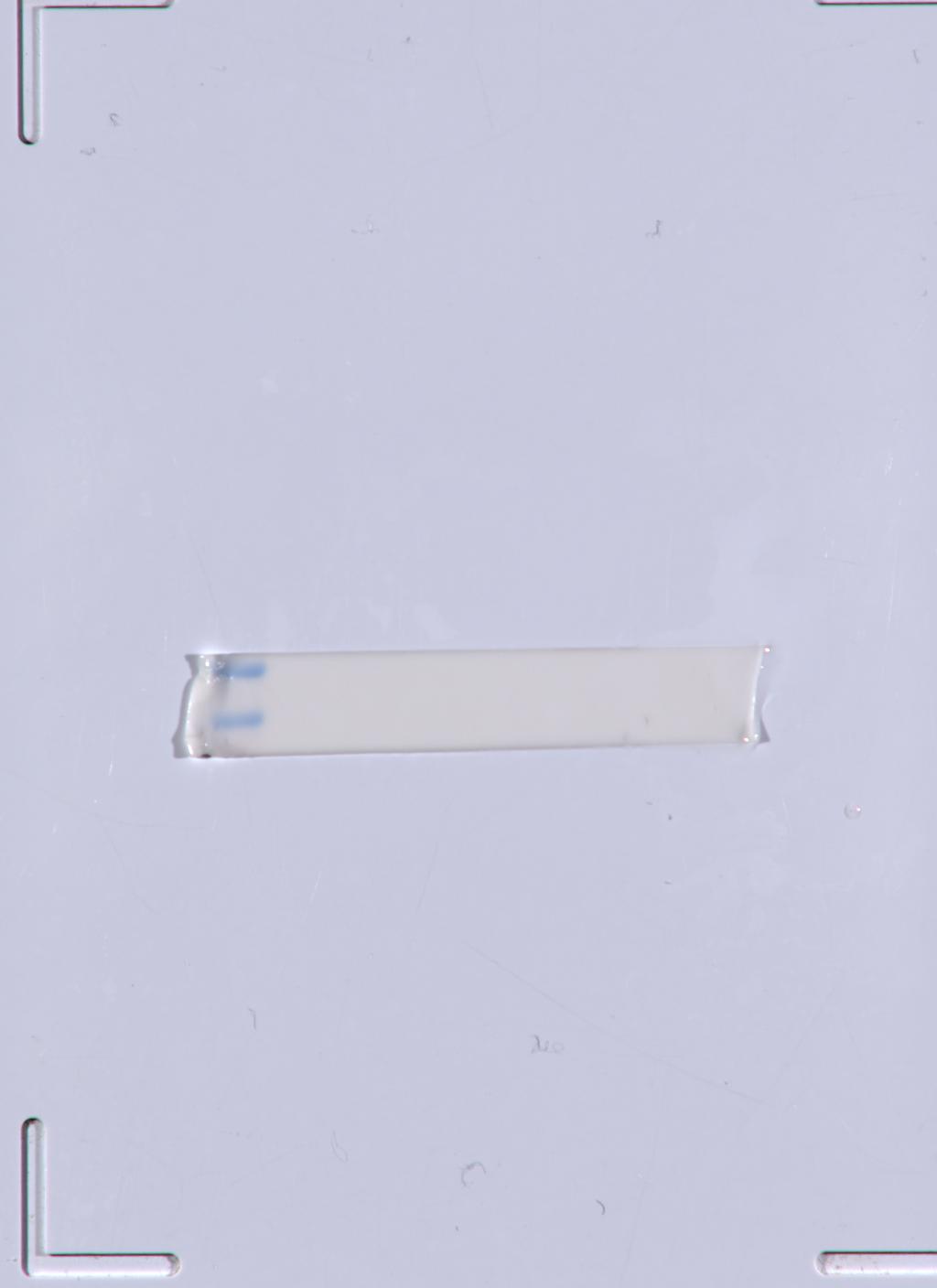

Supplement: Supplemental Information 2 [file peerj-13-19085-s002.zip › Chaetoglobosin A induces T-24 apoptosis in human bladder cancer/7.PI3K ERK pathway/GAPDH/1.11 GAPDH-3 2022.01.11_19.51.03_Ch/1.11 GAPDH-3 2022.01.11_19.51.03_Ch-Marker.jpg]

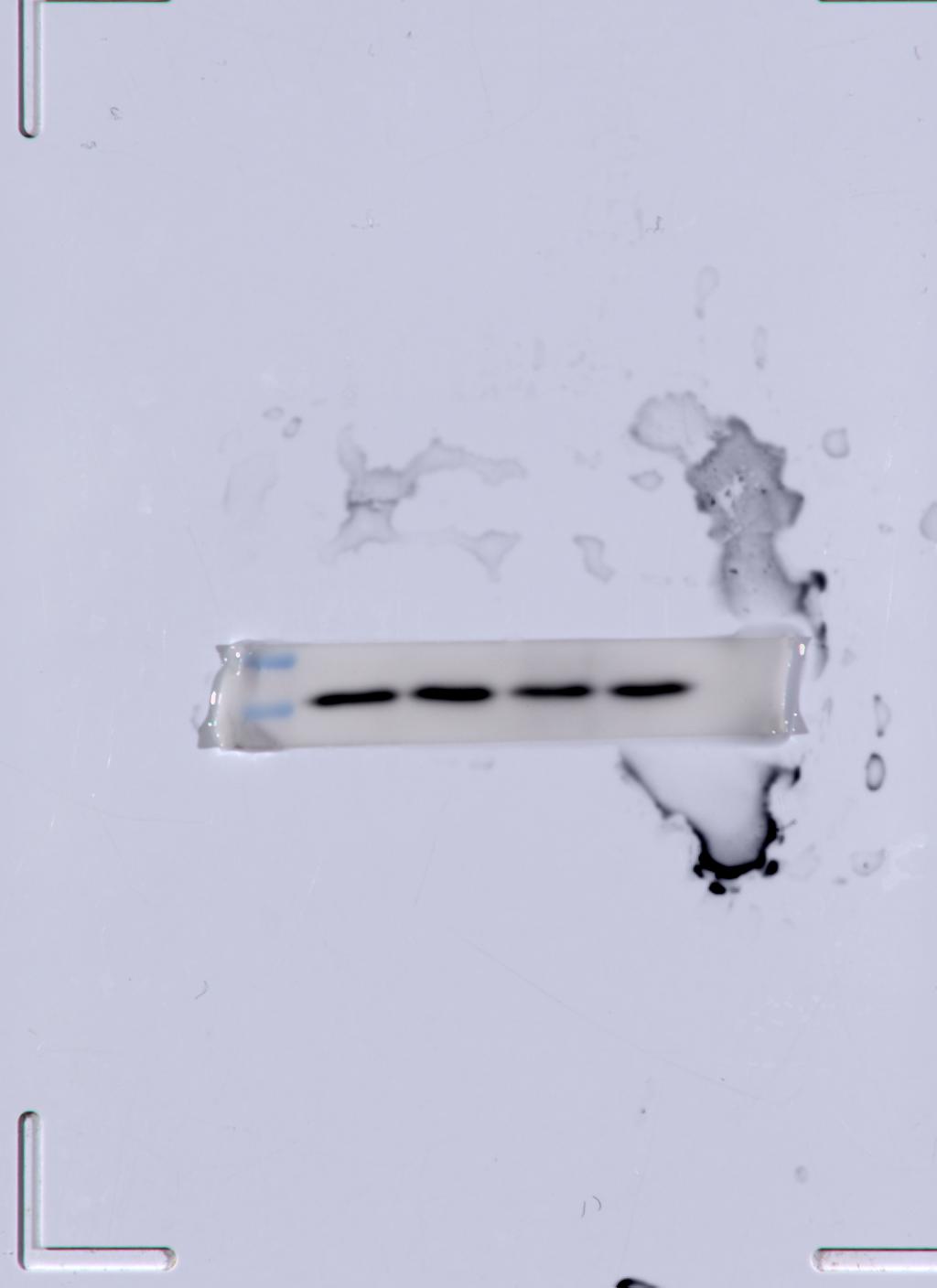

Supplement: Supplemental Information 2 [file peerj-13-19085-s002.zip › Chaetoglobosin A induces T-24 apoptosis in human bladder cancer/7.PI3K ERK pathway/GAPDH/1.11 gapdh-4 2022.01.11_20.24.02_Ch/1.11 gapdh-4 2022.01.11_20.24.02_Ch+Marker.jpg]

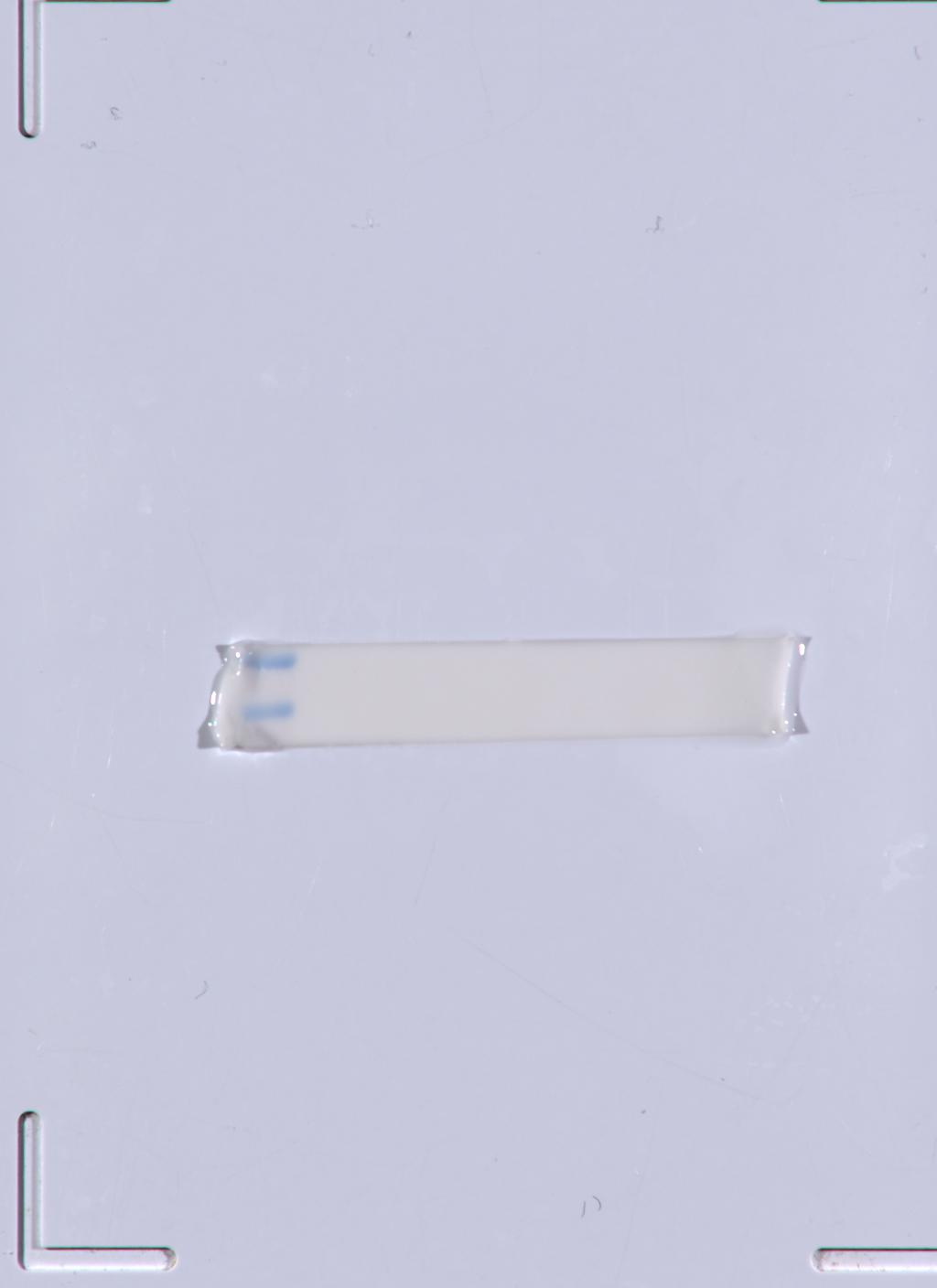

Supplement: Supplemental Information 2 [file peerj-13-19085-s002.zip › Chaetoglobosin A induces T-24 apoptosis in human bladder cancer/7.PI3K ERK pathway/GAPDH/1.11 gapdh-4 2022.01.11_20.24.02_Ch/1.11 gapdh-4 2022.01.11_20.24.02_Ch-Marker.jpg]

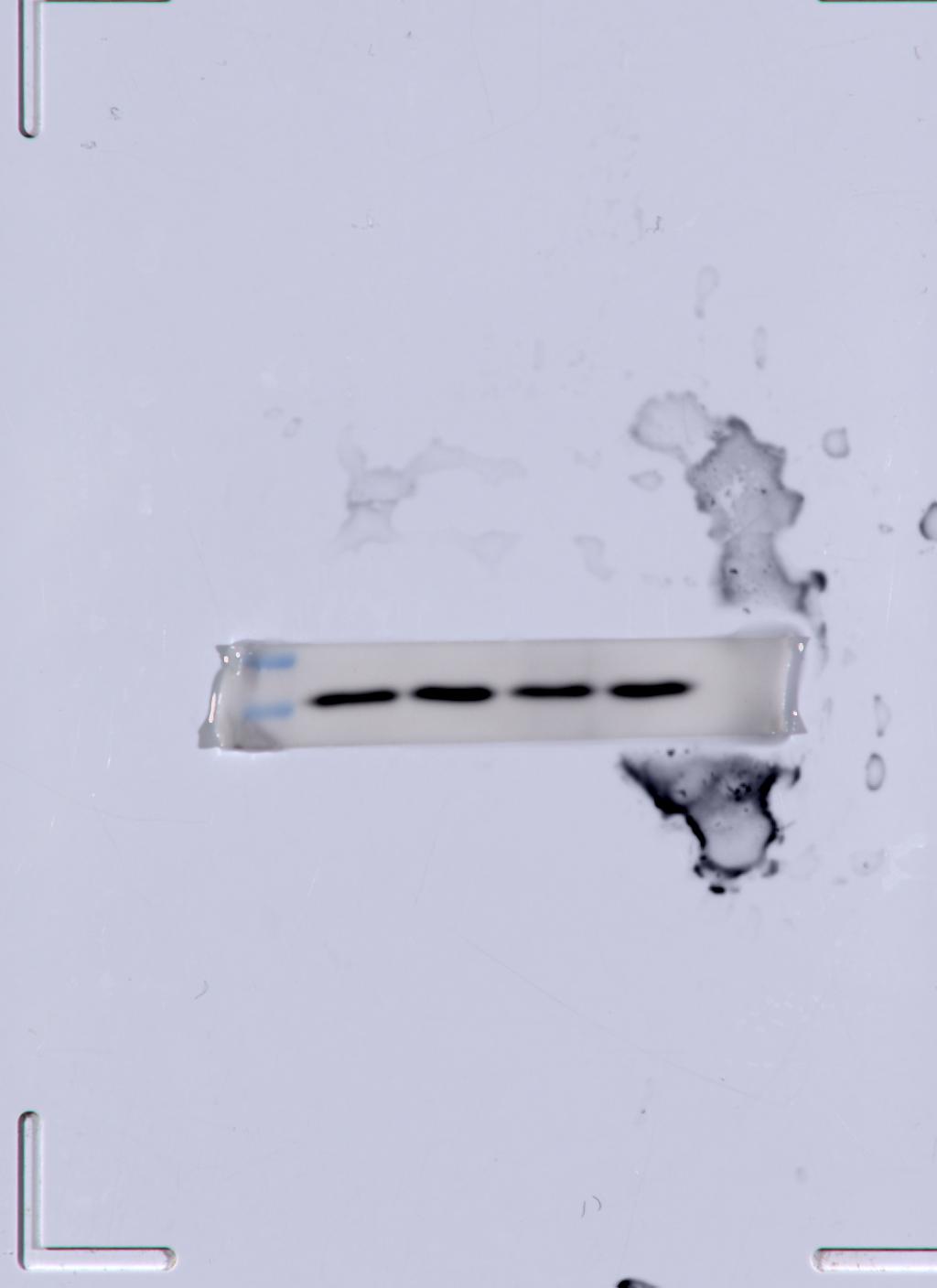

Supplement: Supplemental Information 2 [file peerj-13-19085-s002.zip › Chaetoglobosin A induces T-24 apoptosis in human bladder cancer/7.PI3K ERK pathway/GAPDH/1.11 gapdh-5 2022.01.11_20.29.56_Ch/1.11 gapdh-5 2022.01.11_20.29.56_Ch+Marker.jpg]

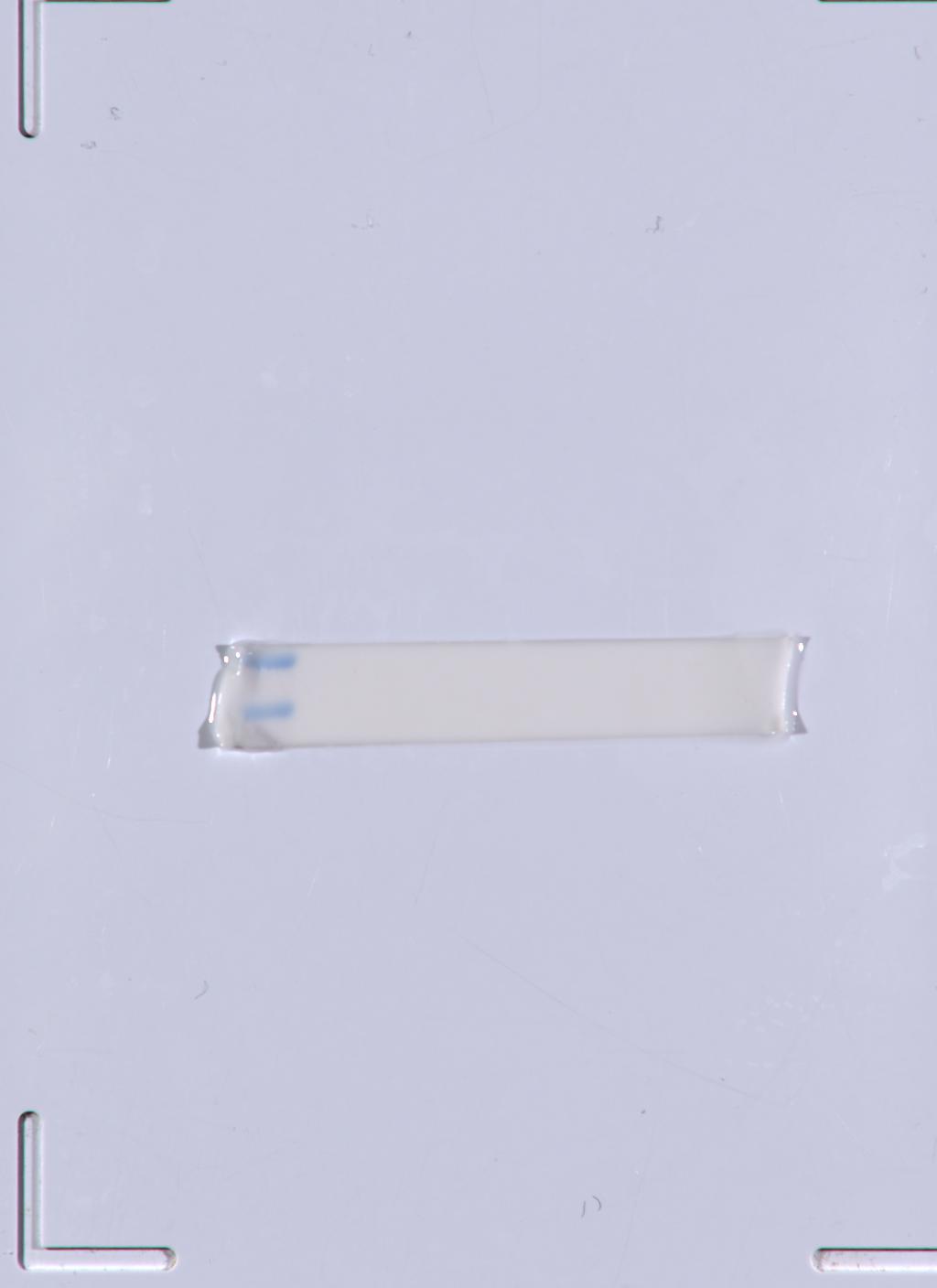

Supplement: Supplemental Information 2 [file peerj-13-19085-s002.zip › Chaetoglobosin A induces T-24 apoptosis in human bladder cancer/7.PI3K ERK pathway/GAPDH/1.11 gapdh-5 2022.01.11_20.29.56_Ch/1.11 gapdh-5 2022.01.11_20.29.56_Ch-Marker.jpg]

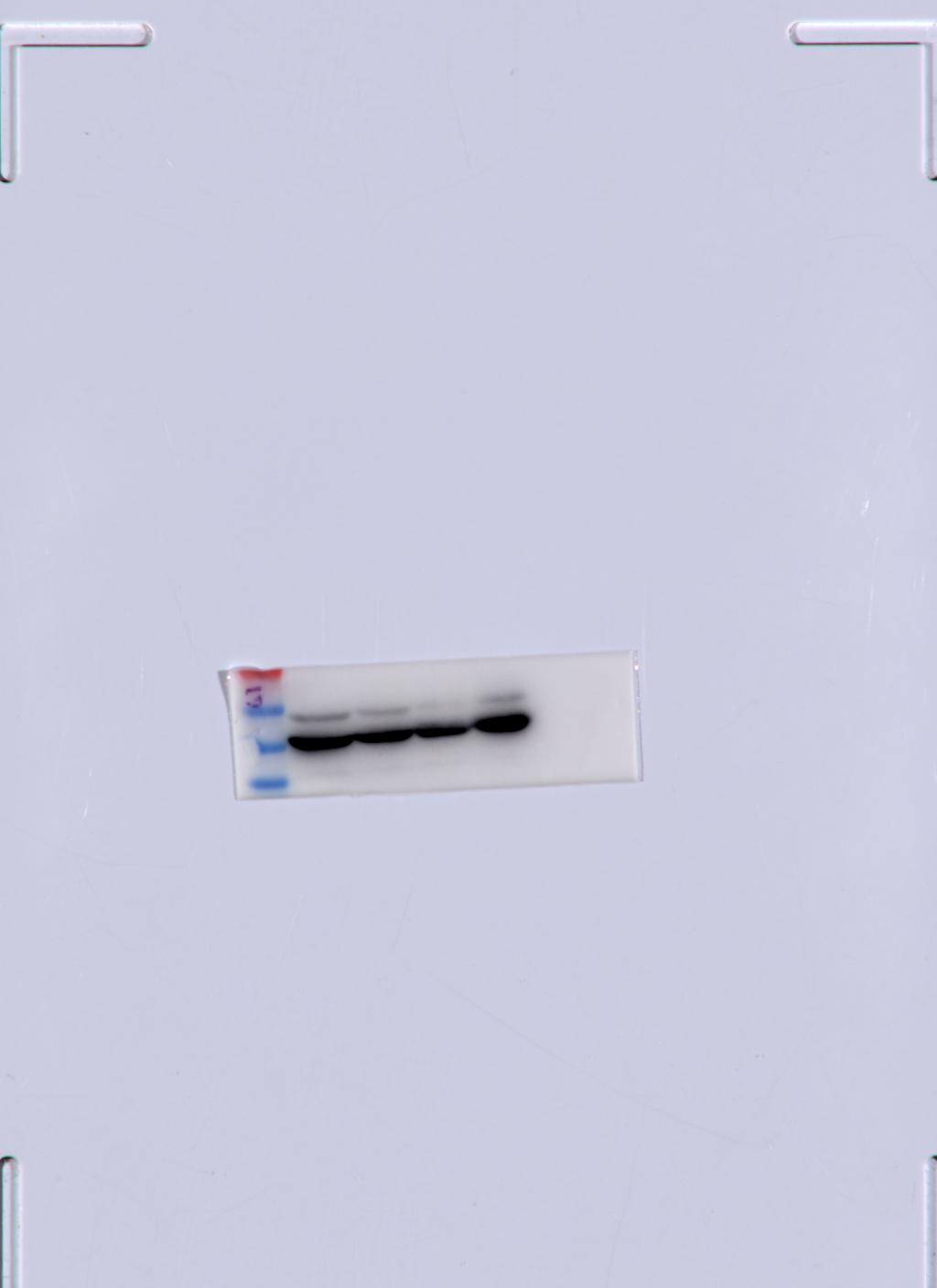

Supplement: Supplemental Information 2 [file peerj-13-19085-s002.zip › Chaetoglobosin A induces T-24 apoptosis in human bladder cancer/7.PI3K ERK pathway/jnk/22.5.4jnk 2022.05.04_13.41.41_Ch/22.5.4jnk 2022.05.04_13.41.41_Ch+Marker.jpg]

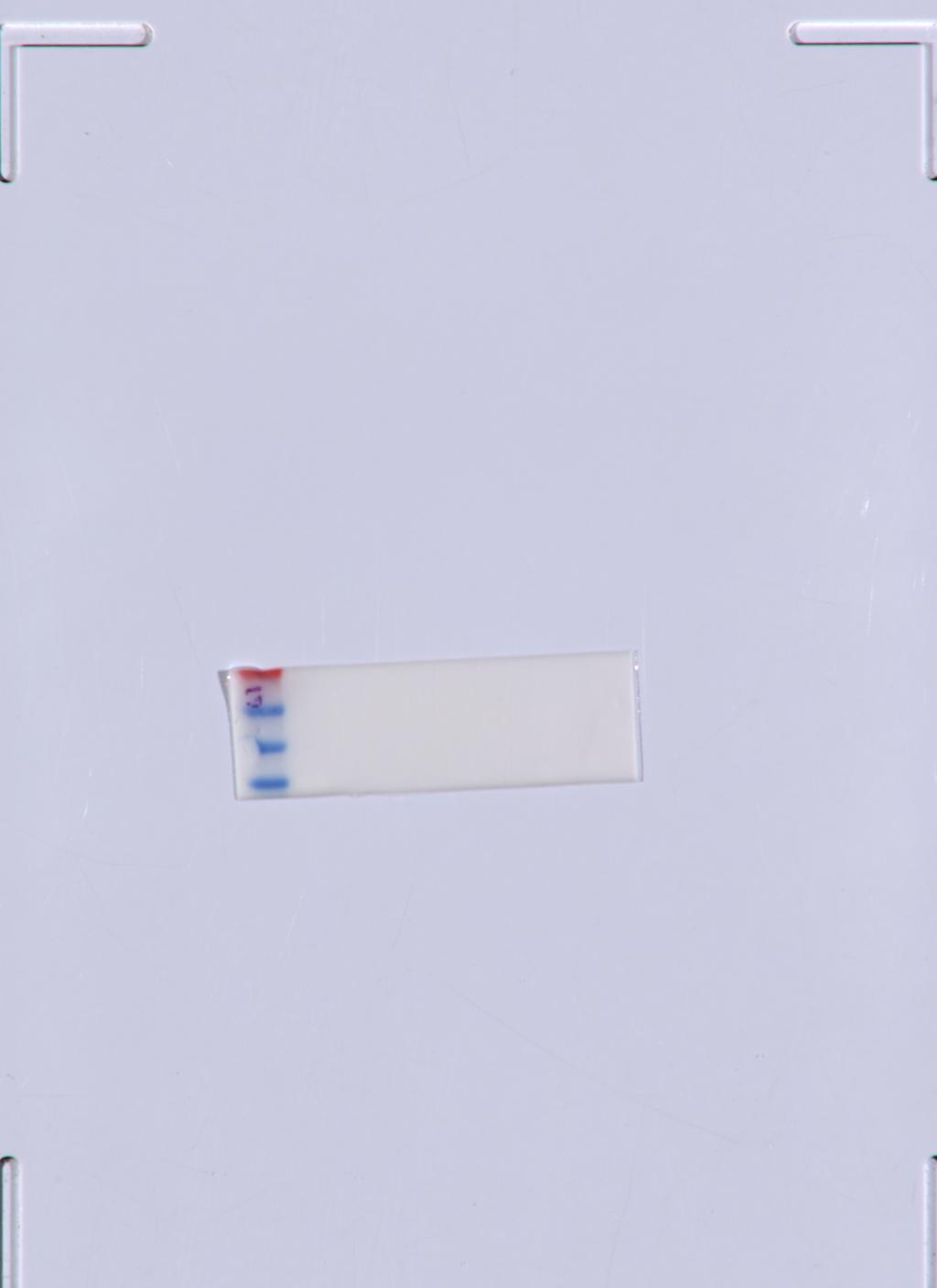

Supplement: Supplemental Information 2 [file peerj-13-19085-s002.zip › Chaetoglobosin A induces T-24 apoptosis in human bladder cancer/7.PI3K ERK pathway/jnk/22.5.4jnk 2022.05.04_13.41.41_Ch/22.5.4jnk 2022.05.04_13.41.41_Ch-Marker.jpg]

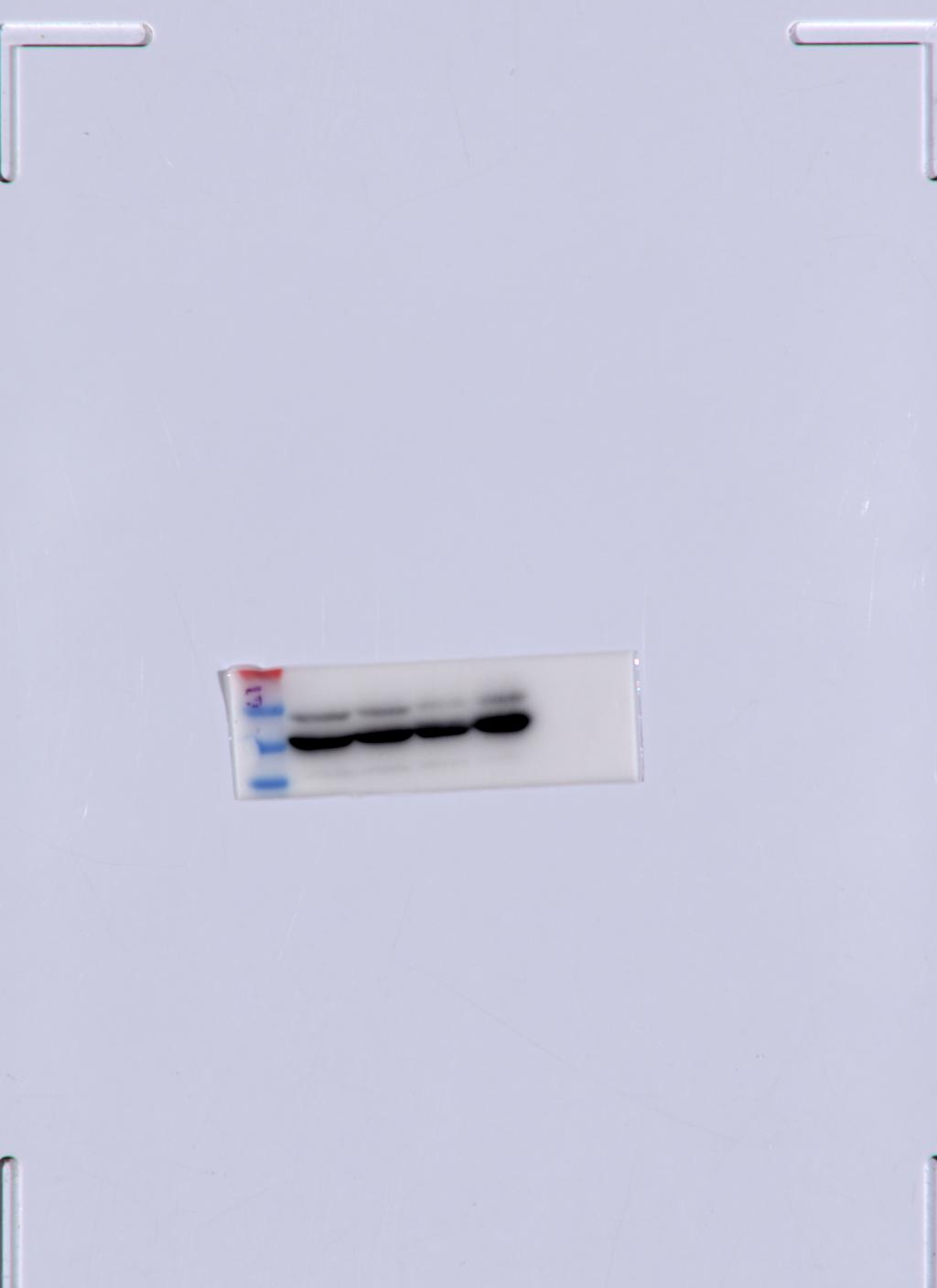

Supplement: Supplemental Information 2 [file peerj-13-19085-s002.zip › Chaetoglobosin A induces T-24 apoptosis in human bladder cancer/7.PI3K ERK pathway/jnk/22.5.4jnk2 2022.05.04_13.43.23_Ch/22.5.4jnk2 2022.05.04_13.43.23_Ch+Marker.jpg]

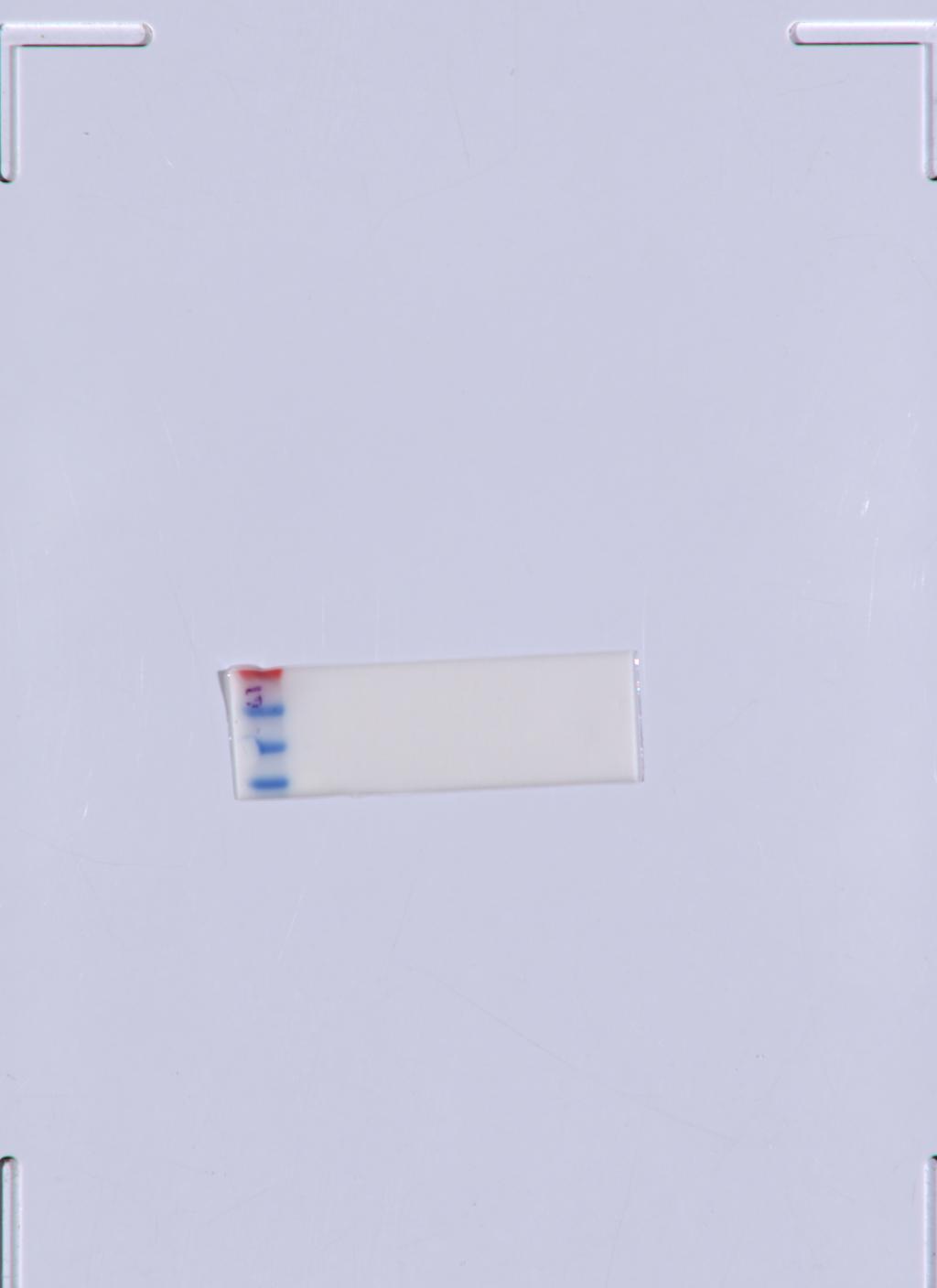

Supplement: Supplemental Information 2 [file peerj-13-19085-s002.zip › Chaetoglobosin A induces T-24 apoptosis in human bladder cancer/7.PI3K ERK pathway/jnk/22.5.4jnk2 2022.05.04_13.43.23_Ch/22.5.4jnk2 2022.05.04_13.43.23_Ch-Marker.jpg]

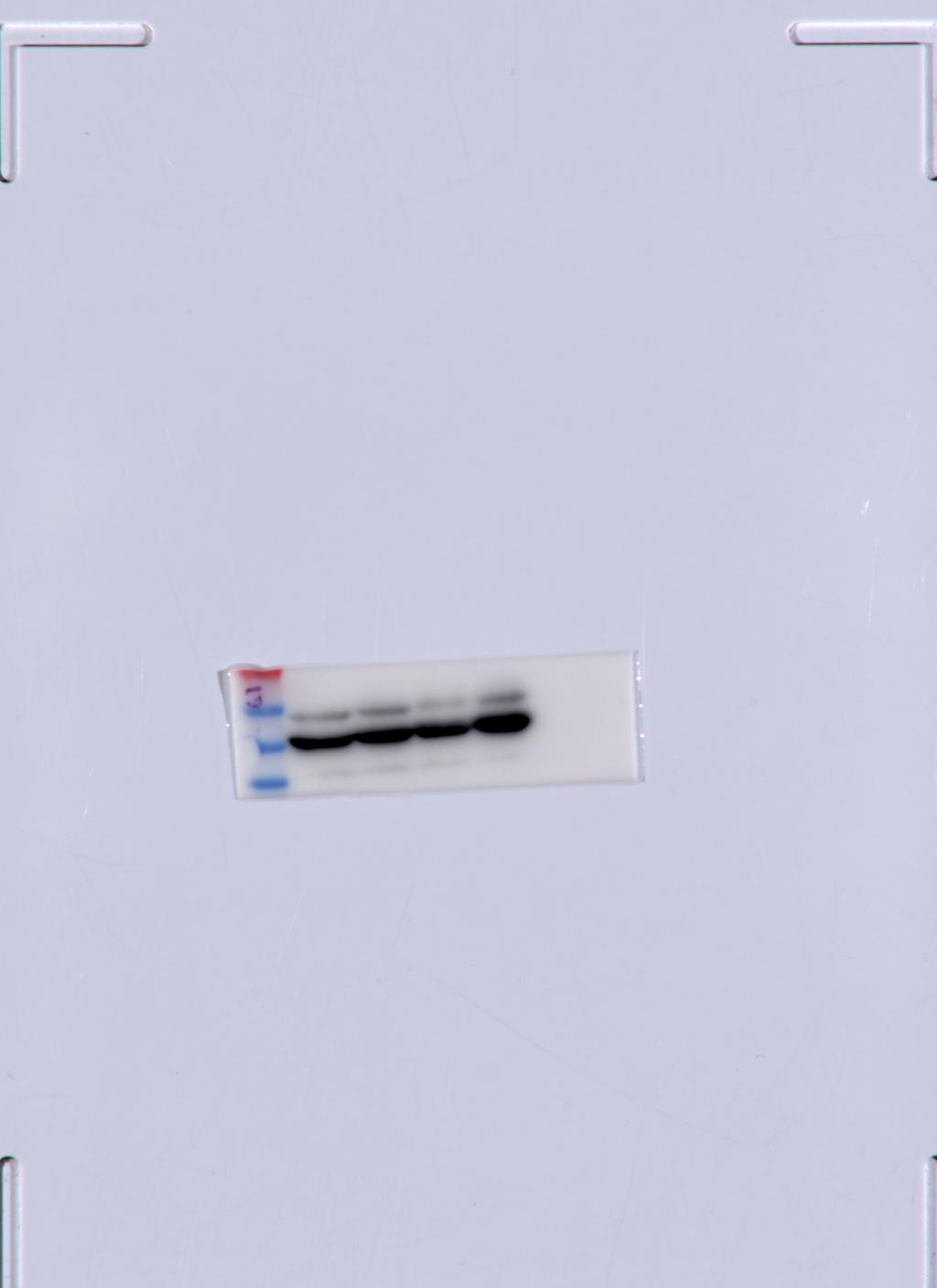

Supplement: Supplemental Information 2 [file peerj-13-19085-s002.zip › Chaetoglobosin A induces T-24 apoptosis in human bladder cancer/7.PI3K ERK pathway/jnk/22.5.4jnk3 2022.05.04_13.45.38_Ch/22.5.4jnk3 2022.05.04_13.45.38_Ch+Marker.jpg]

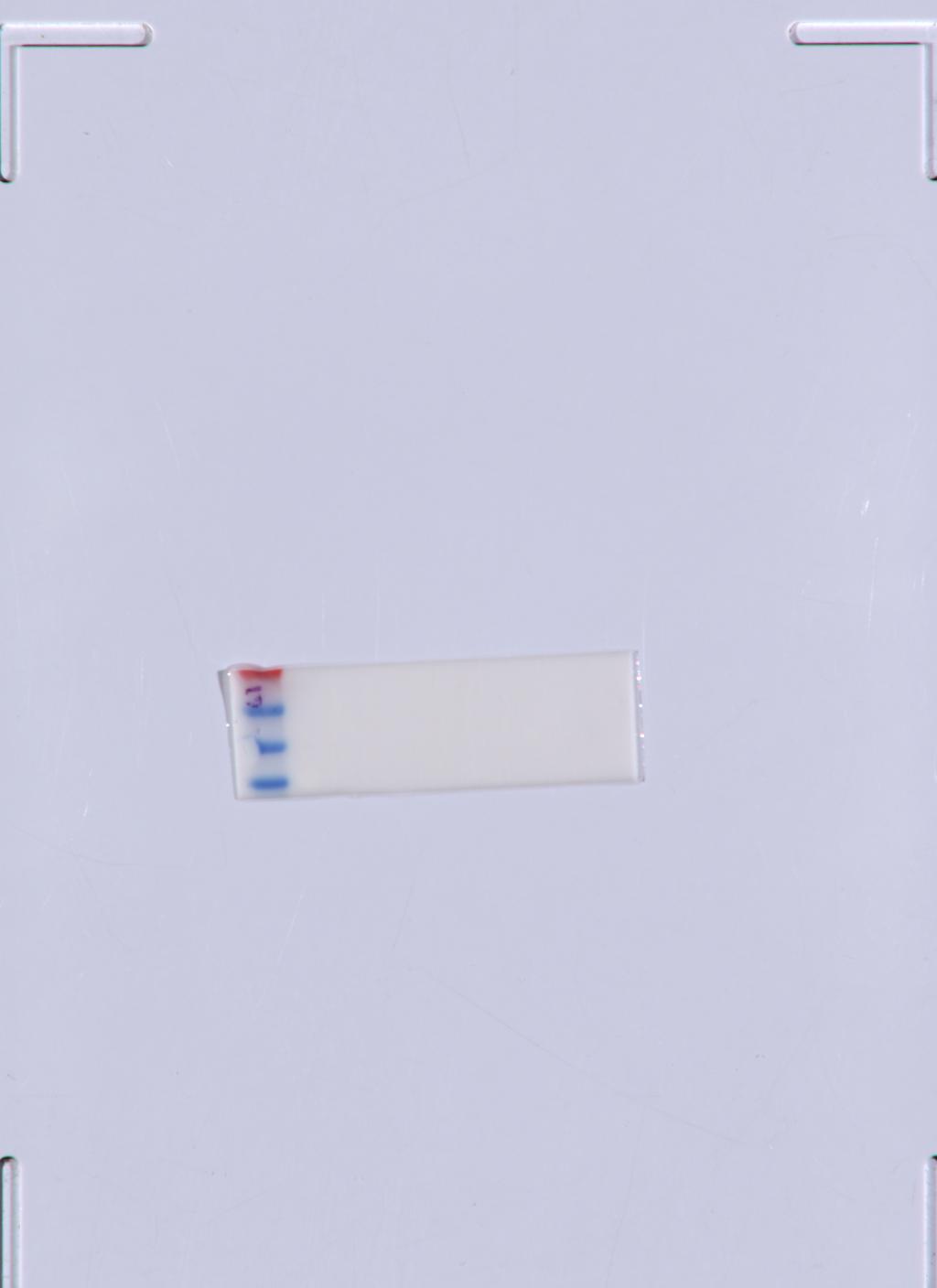

Supplement: Supplemental Information 2 [file peerj-13-19085-s002.zip › Chaetoglobosin A induces T-24 apoptosis in human bladder cancer/7.PI3K ERK pathway/jnk/22.5.4jnk3 2022.05.04_13.45.38_Ch/22.5.4jnk3 2022.05.04_13.45.38_Ch-Marker.jpg]

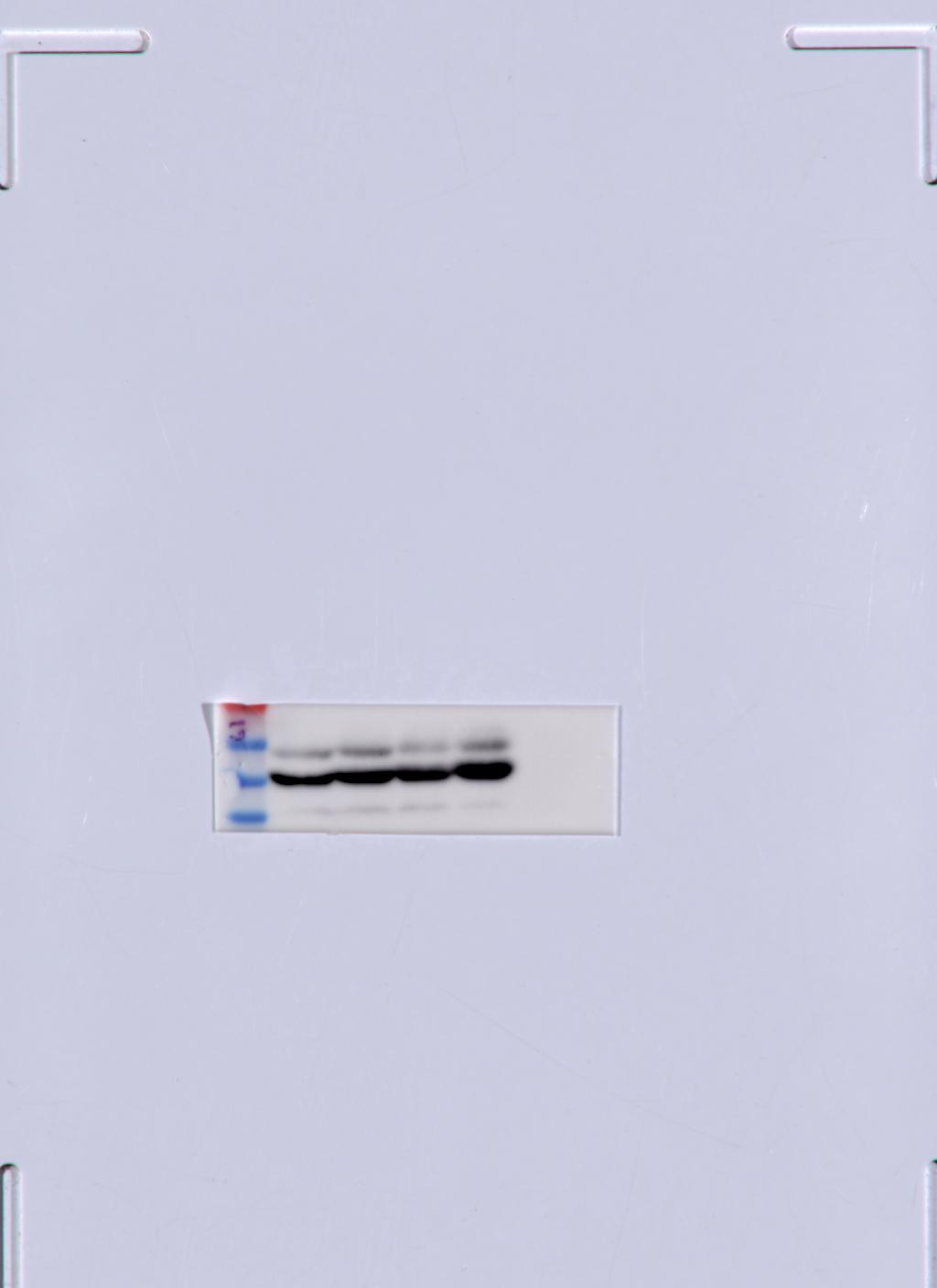

Supplement: Supplemental Information 2 [file peerj-13-19085-s002.zip › Chaetoglobosin A induces T-24 apoptosis in human bladder cancer/7.PI3K ERK pathway/jnk/22.5.4jnk4 2022.05.04_13.48.21_Ch/22.5.4jnk4 2022.05.04_13.48.21_Ch+Marker.jpg]

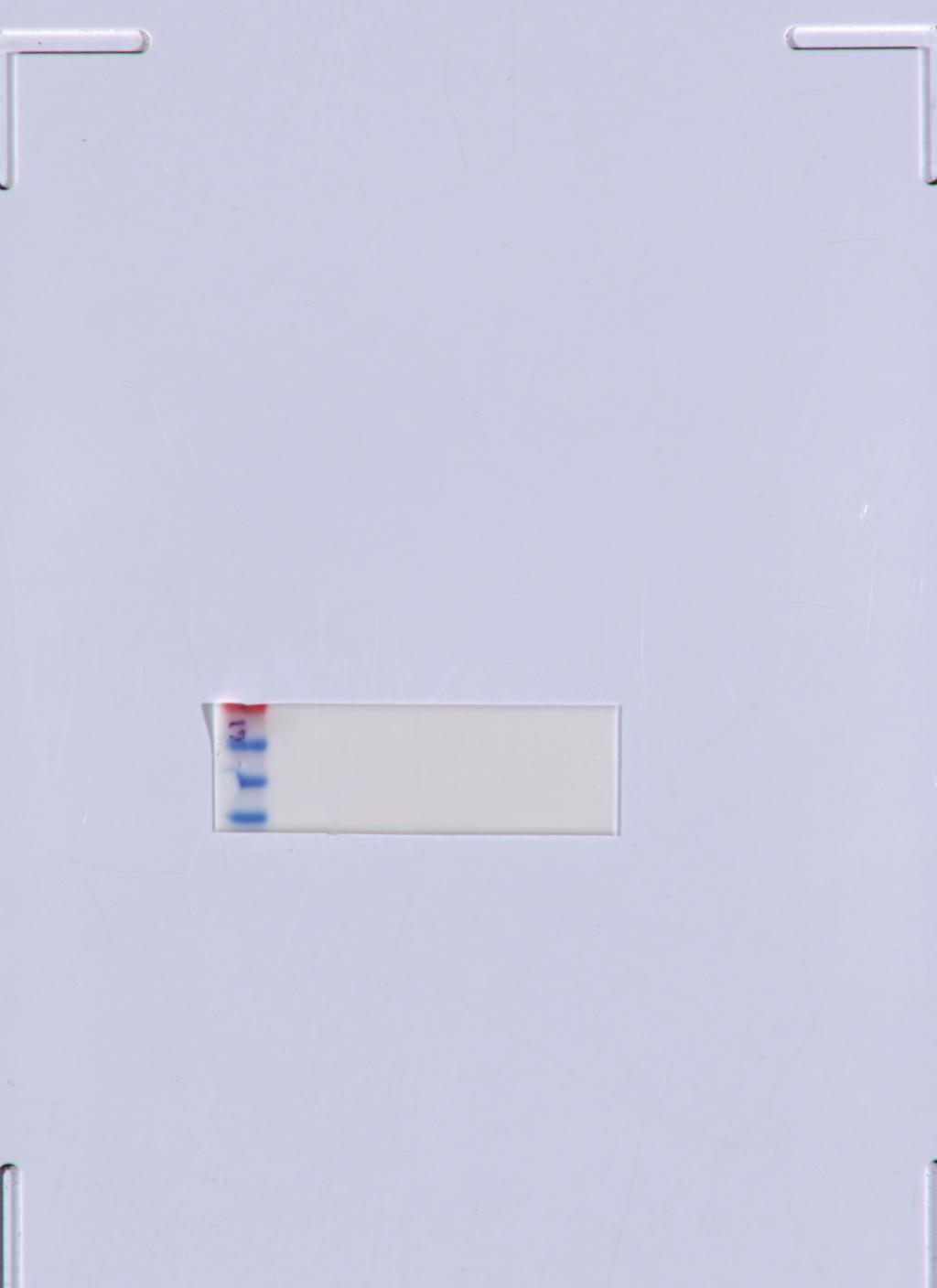

Supplement: Supplemental Information 2 [file peerj-13-19085-s002.zip › Chaetoglobosin A induces T-24 apoptosis in human bladder cancer/7.PI3K ERK pathway/jnk/22.5.4jnk4 2022.05.04_13.48.21_Ch/22.5.4jnk4 2022.05.04_13.48.21_Ch-Marker.jpg]

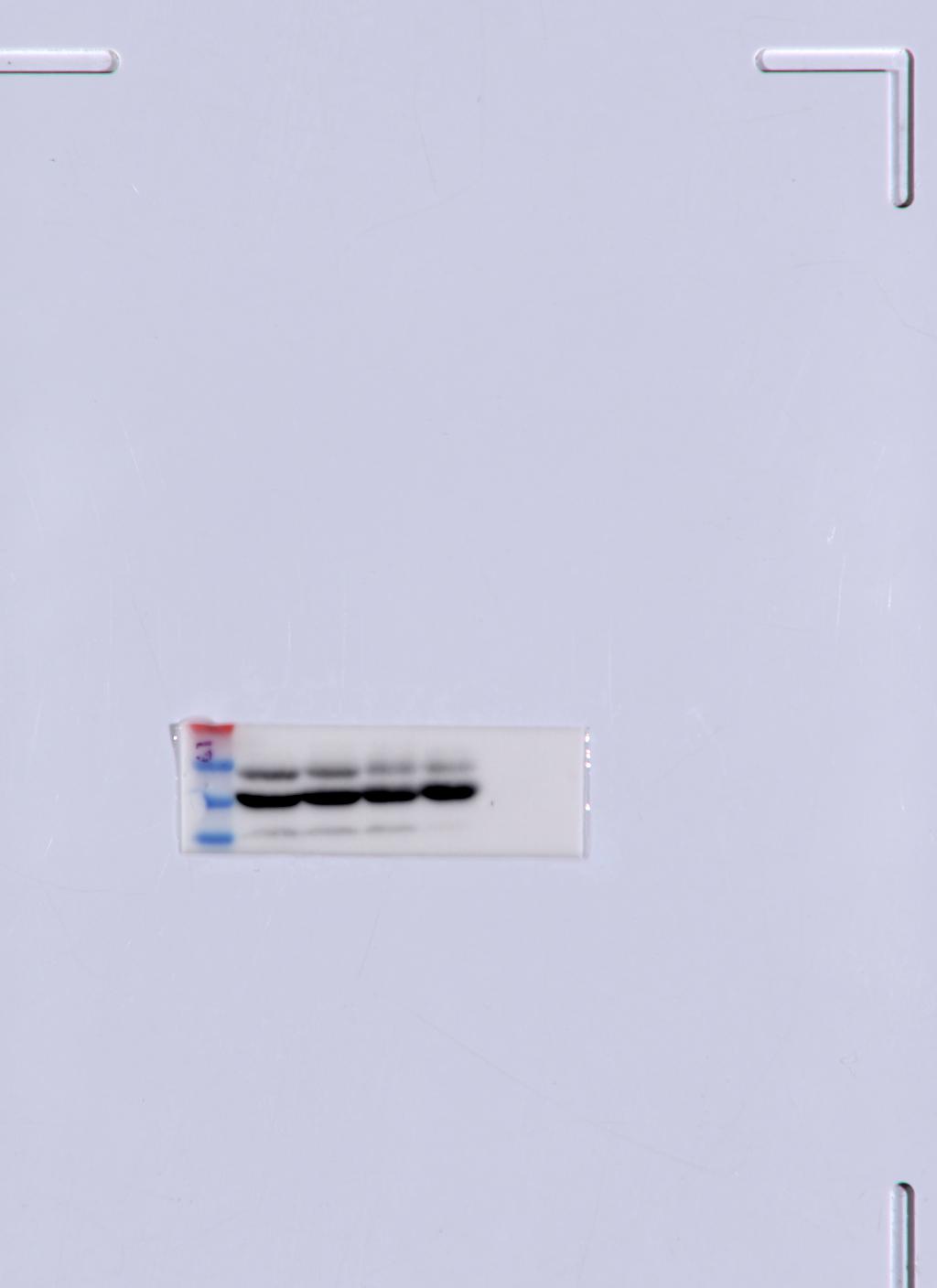

Supplement: Supplemental Information 2 [file peerj-13-19085-s002.zip › Chaetoglobosin A induces T-24 apoptosis in human bladder cancer/7.PI3K ERK pathway/jnk/22.5.4jnk5 2022.05.04_13.54.32_Ch/22.5.4jnk5 2022.05.04_13.54.32_Ch+Marker.jpg]

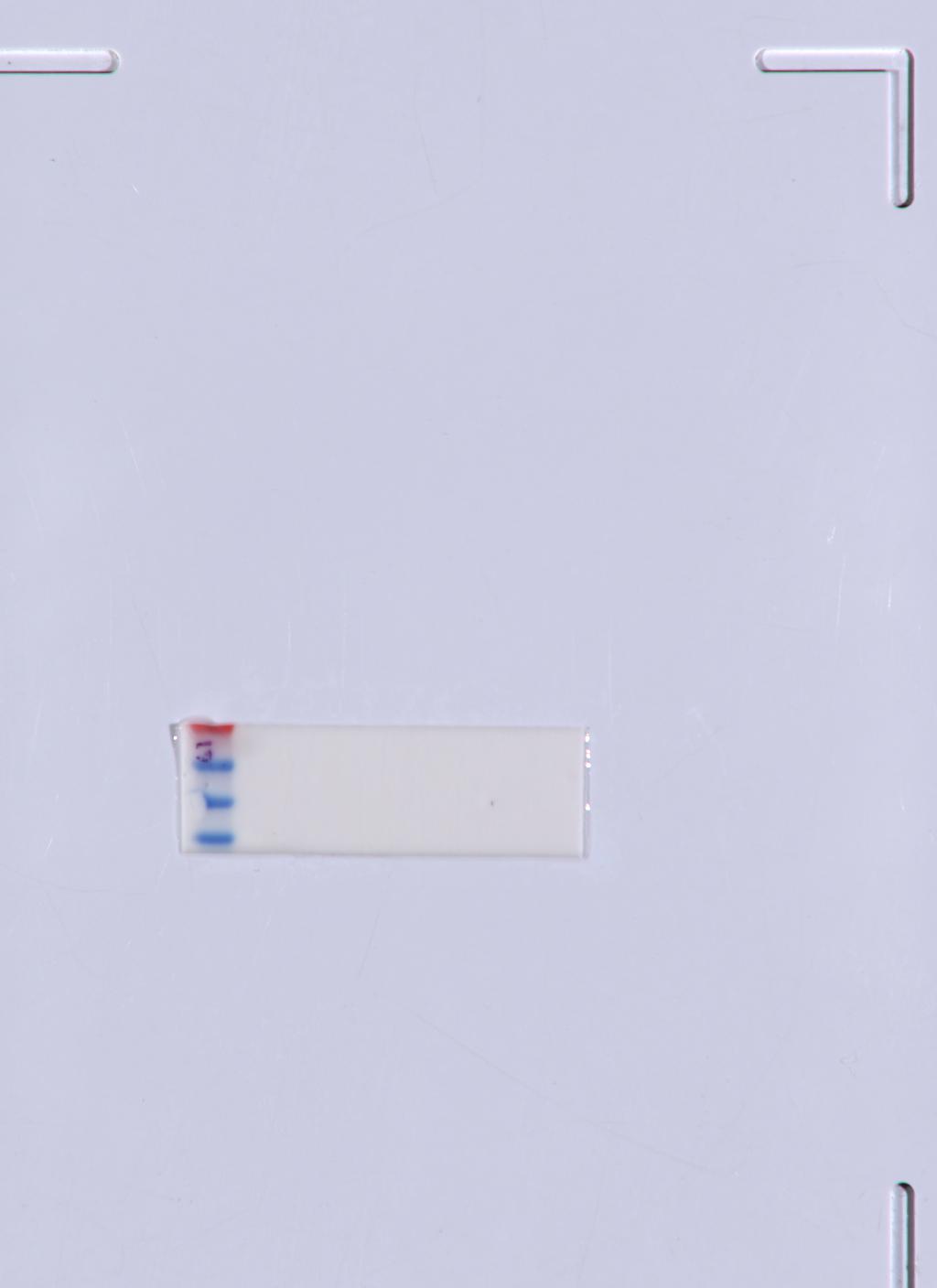

Supplement: Supplemental Information 2 [file peerj-13-19085-s002.zip › Chaetoglobosin A induces T-24 apoptosis in human bladder cancer/7.PI3K ERK pathway/jnk/22.5.4jnk5 2022.05.04_13.54.32_Ch/22.5.4jnk5 2022.05.04_13.54.32_Ch-Marker.jpg]

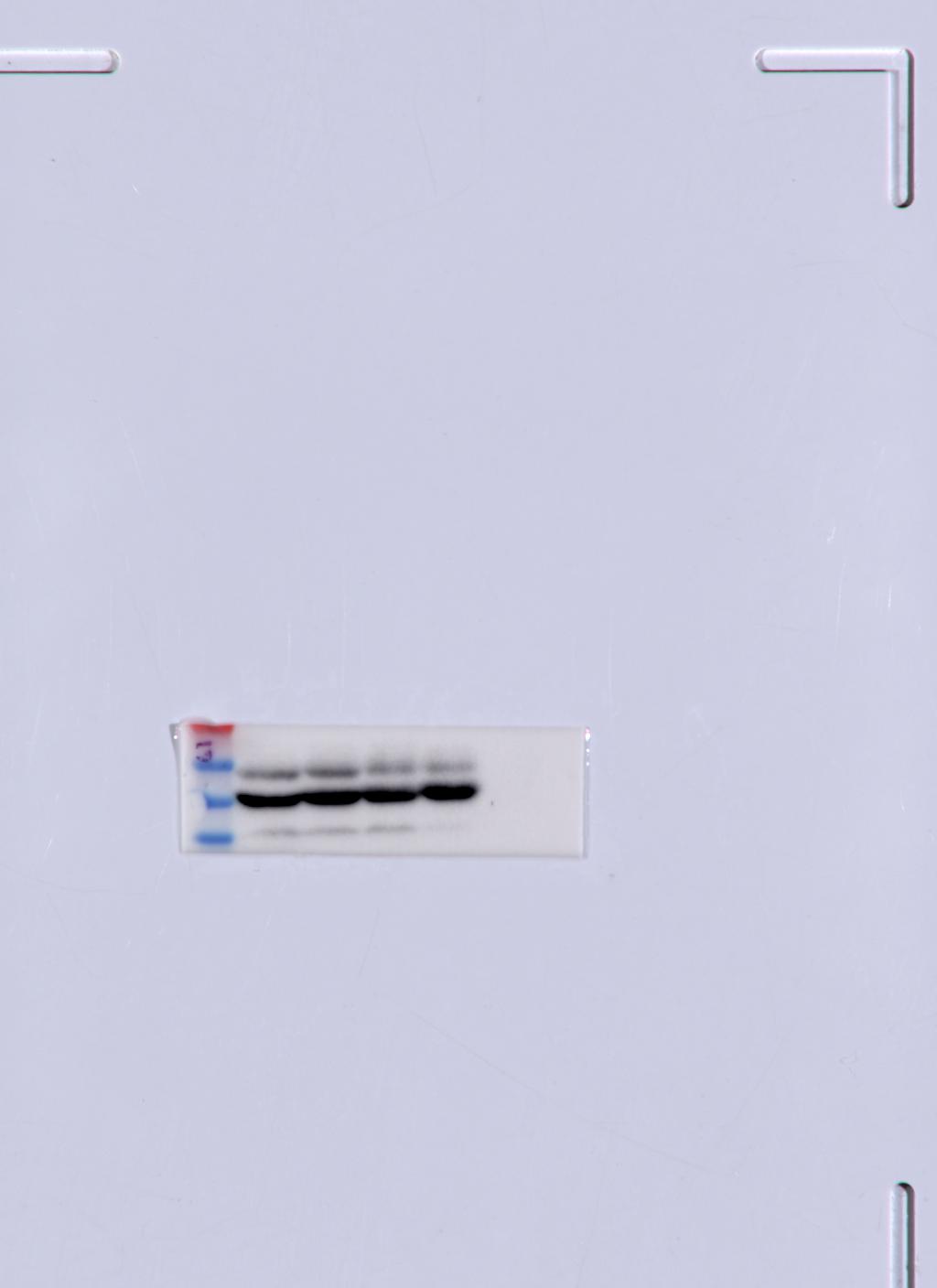

Supplement: Supplemental Information 2 [file peerj-13-19085-s002.zip › Chaetoglobosin A induces T-24 apoptosis in human bladder cancer/7.PI3K ERK pathway/jnk/22.5.4jnk6 2022.05.04_13.56.26_Ch/22.5.4jnk6 2022.05.04_13.56.26_Ch+Marker.jpg]

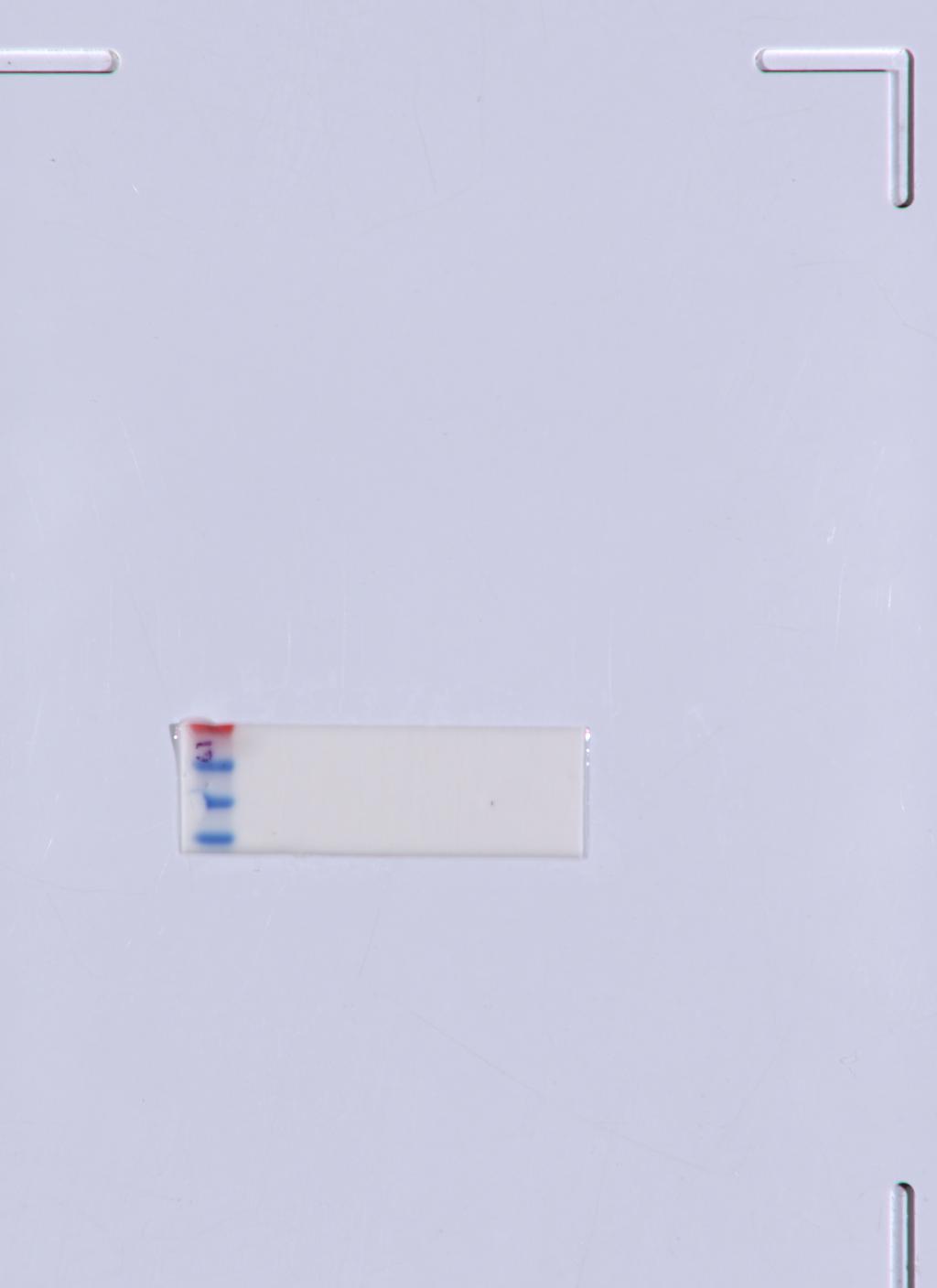

Supplement: Supplemental Information 2 [file peerj-13-19085-s002.zip › Chaetoglobosin A induces T-24 apoptosis in human bladder cancer/7.PI3K ERK pathway/jnk/22.5.4jnk6 2022.05.04_13.56.26_Ch/22.5.4jnk6 2022.05.04_13.56.26_Ch-Marker.jpg]

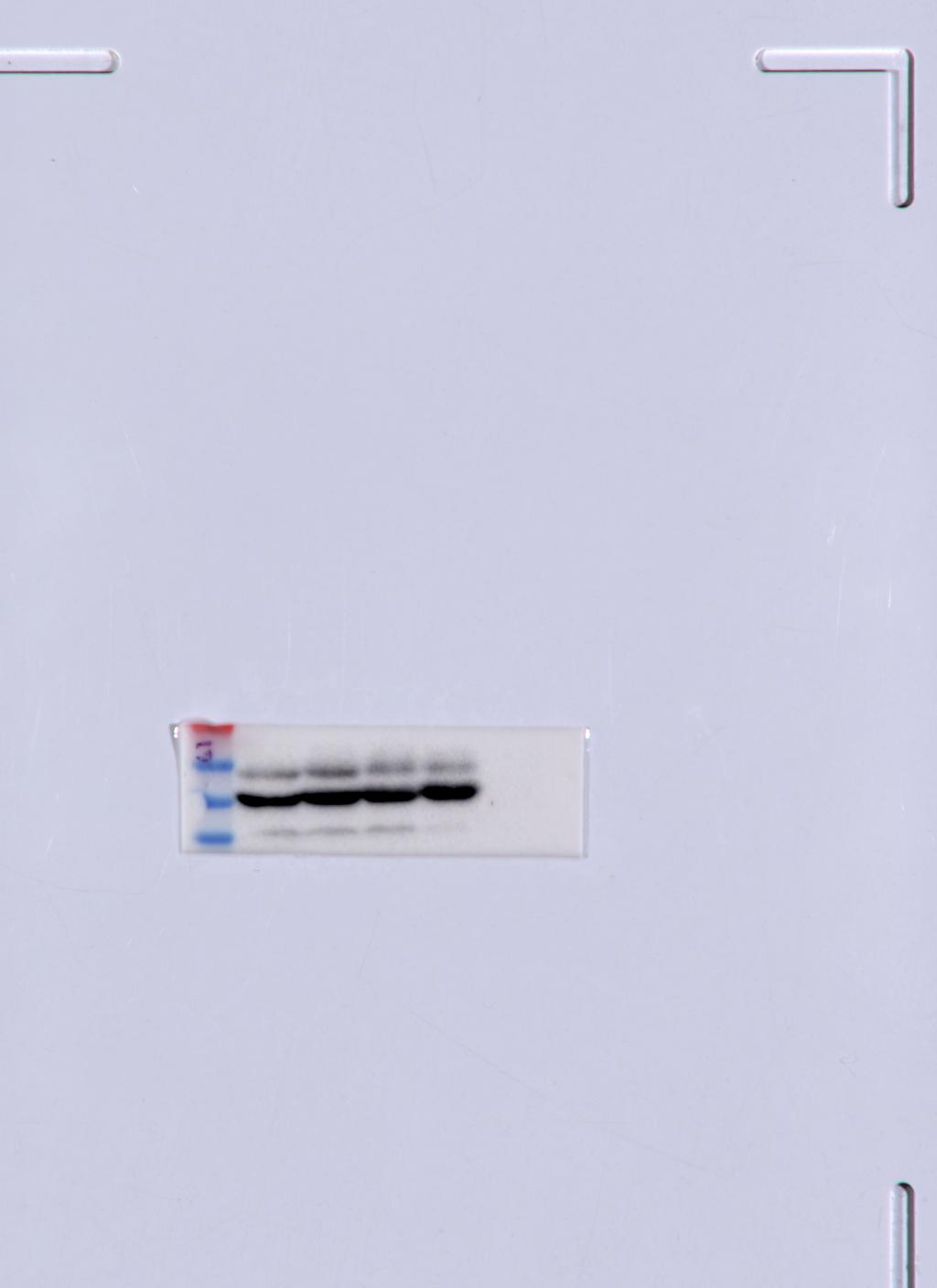

Supplement: Supplemental Information 2 [file peerj-13-19085-s002.zip › Chaetoglobosin A induces T-24 apoptosis in human bladder cancer/7.PI3K ERK pathway/jnk/22.5.4jnk7 2022.05.04_13.57.52_Ch/22.5.4jnk7 2022.05.04_13.57.52_Ch+Marker.jpg]

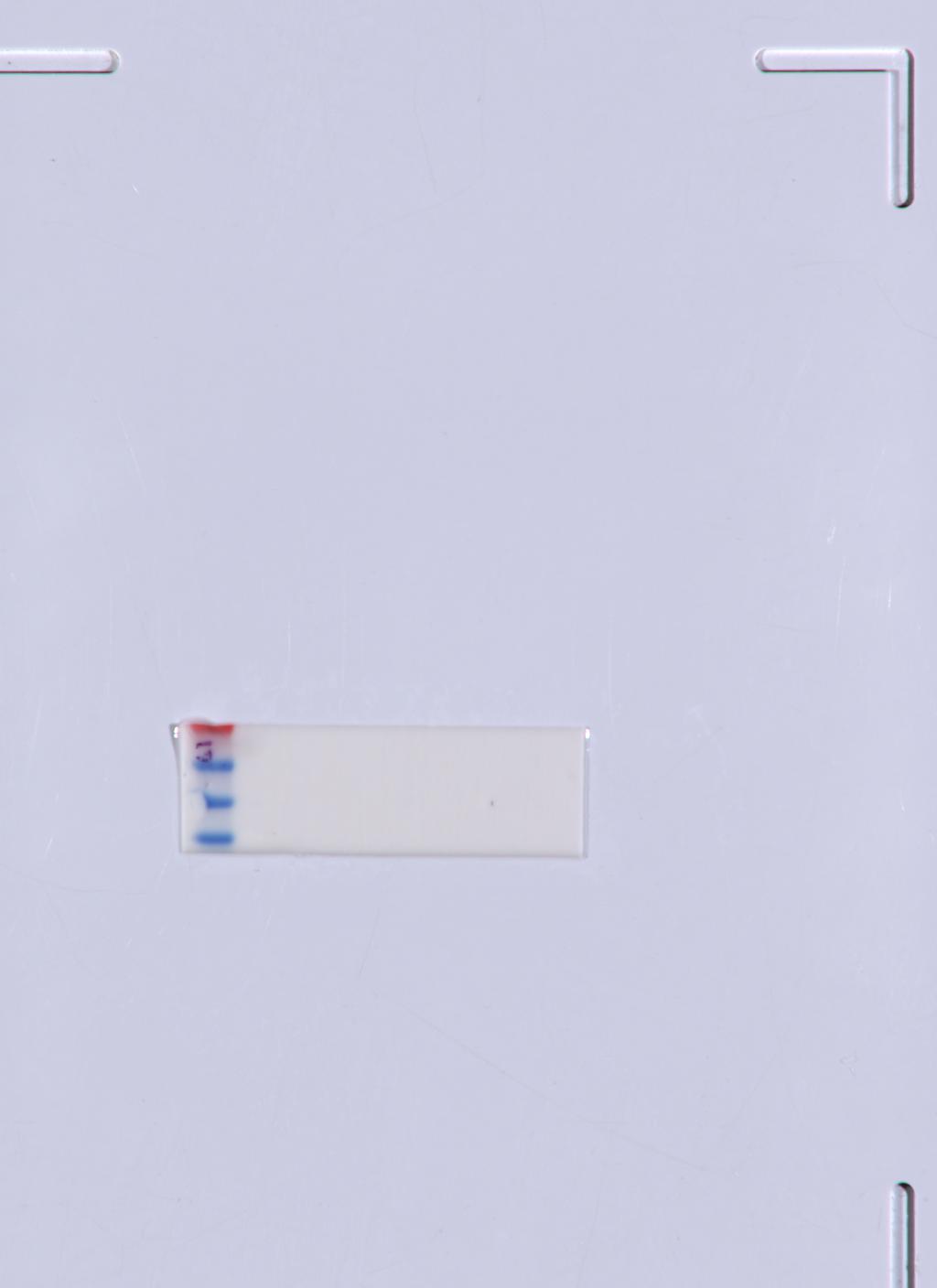

Supplement: Supplemental Information 2 [file peerj-13-19085-s002.zip › Chaetoglobosin A induces T-24 apoptosis in human bladder cancer/7.PI3K ERK pathway/jnk/22.5.4jnk7 2022.05.04_13.57.52_Ch/22.5.4jnk7 2022.05.04_13.57.52_Ch-Marker.jpg]

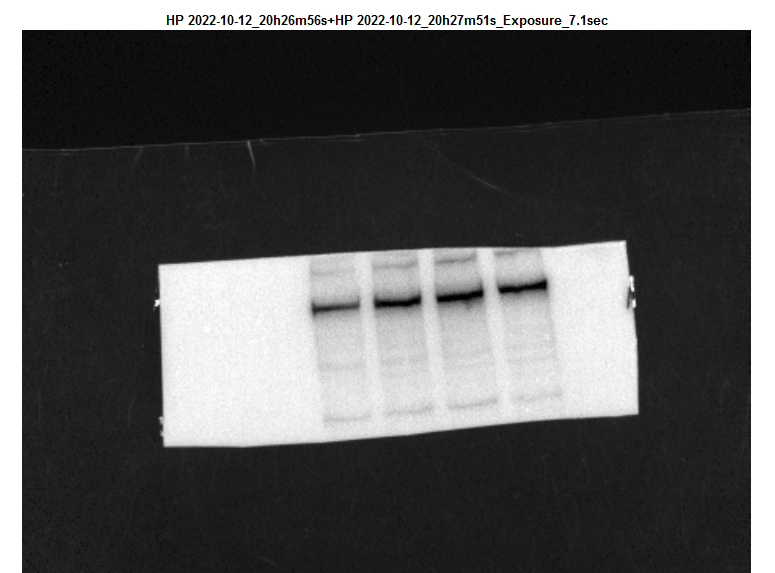

Supplement: Supplemental Information 2 [file peerj-13-19085-s002.zip › Chaetoglobosin A induces T-24 apoptosis in human bladder cancer/7.PI3K ERK pathway/mTOR/HP 2022-10-12_20h26m56s+HP 2022-10-12_20h27m51s_Exposure_7.1sec.tif]

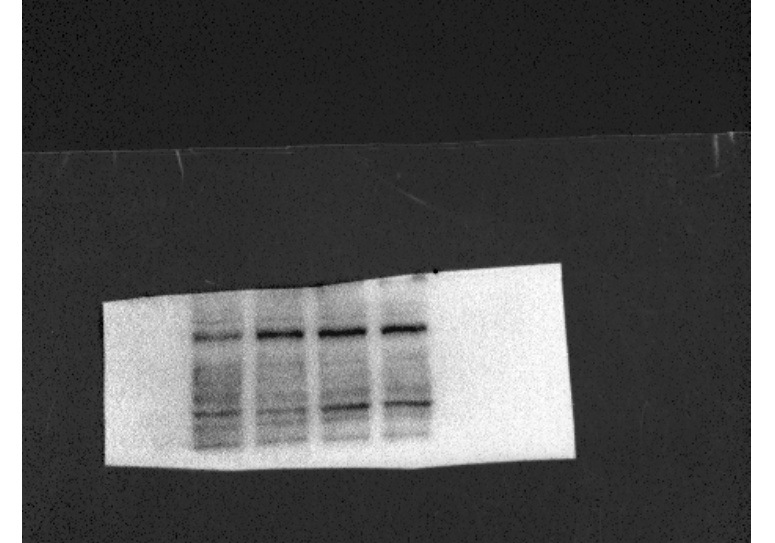

Supplement: Supplemental Information 2 [file peerj-13-19085-s002.zip › Chaetoglobosin A induces T-24 apoptosis in human bladder cancer/7.PI3K ERK pathway/p-AKT/HP 2022-10-14_10h57m31s+HP 2022-10-14_10h53m36s_Exposure_56.9sec.tif]

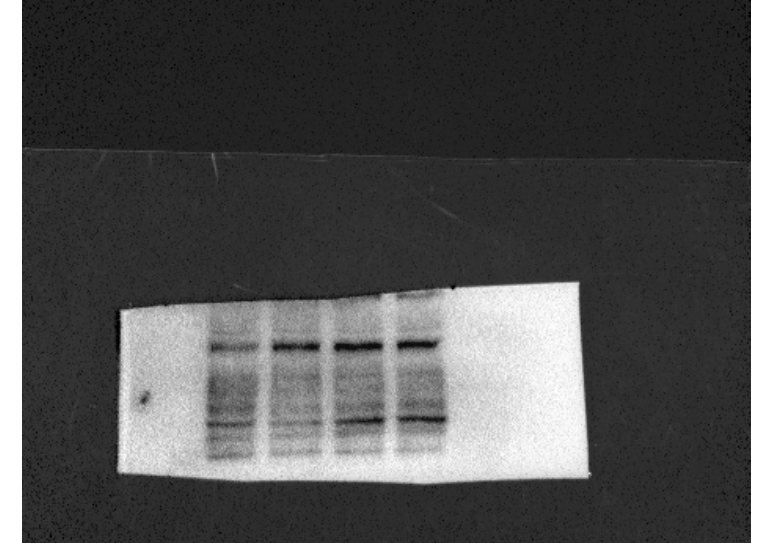

Supplement: Supplemental Information 2 [file peerj-13-19085-s002.zip › Chaetoglobosin A induces T-24 apoptosis in human bladder cancer/7.PI3K ERK pathway/p-AKT/HP 2022-10-14_11h02m22s+HP 2022-10-14_10h58m12s_Exposure_60.0sec.tif]

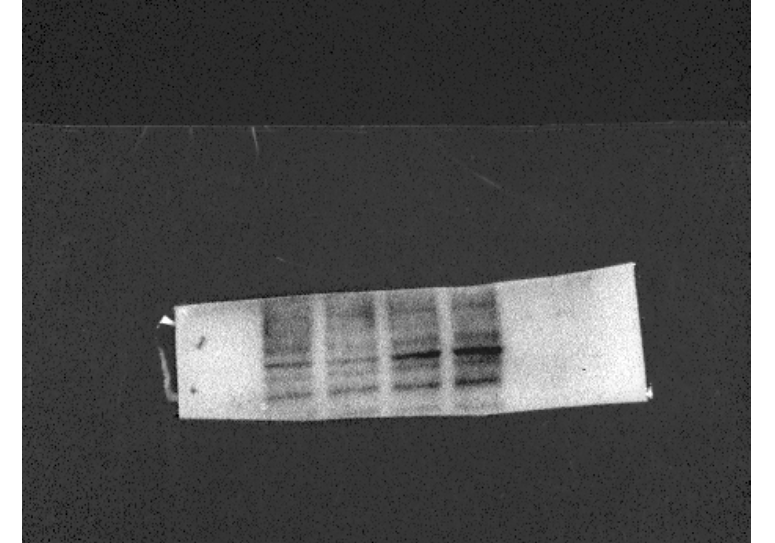

Supplement: Supplemental Information 2 [file peerj-13-19085-s002.zip › Chaetoglobosin A induces T-24 apoptosis in human bladder cancer/7.PI3K ERK pathway/p-AKT/HP 2022-10-14_11h26m10s+HP 2022-10-14_11h24m41s_Exposure_51.6sec.tif]

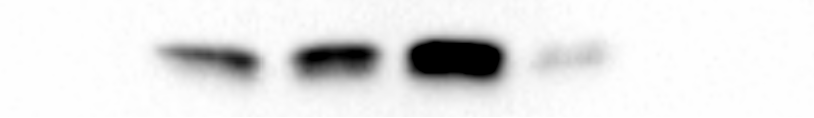

Supplement: Supplemental Information 2 [file peerj-13-19085-s002.zip › Chaetoglobosin A induces T-24 apoptosis in human bladder cancer/7.PI3K ERK pathway/P-ERK/Administrator 2022-10-20 14 时 18 分_Exposure_30.0sec.tif]

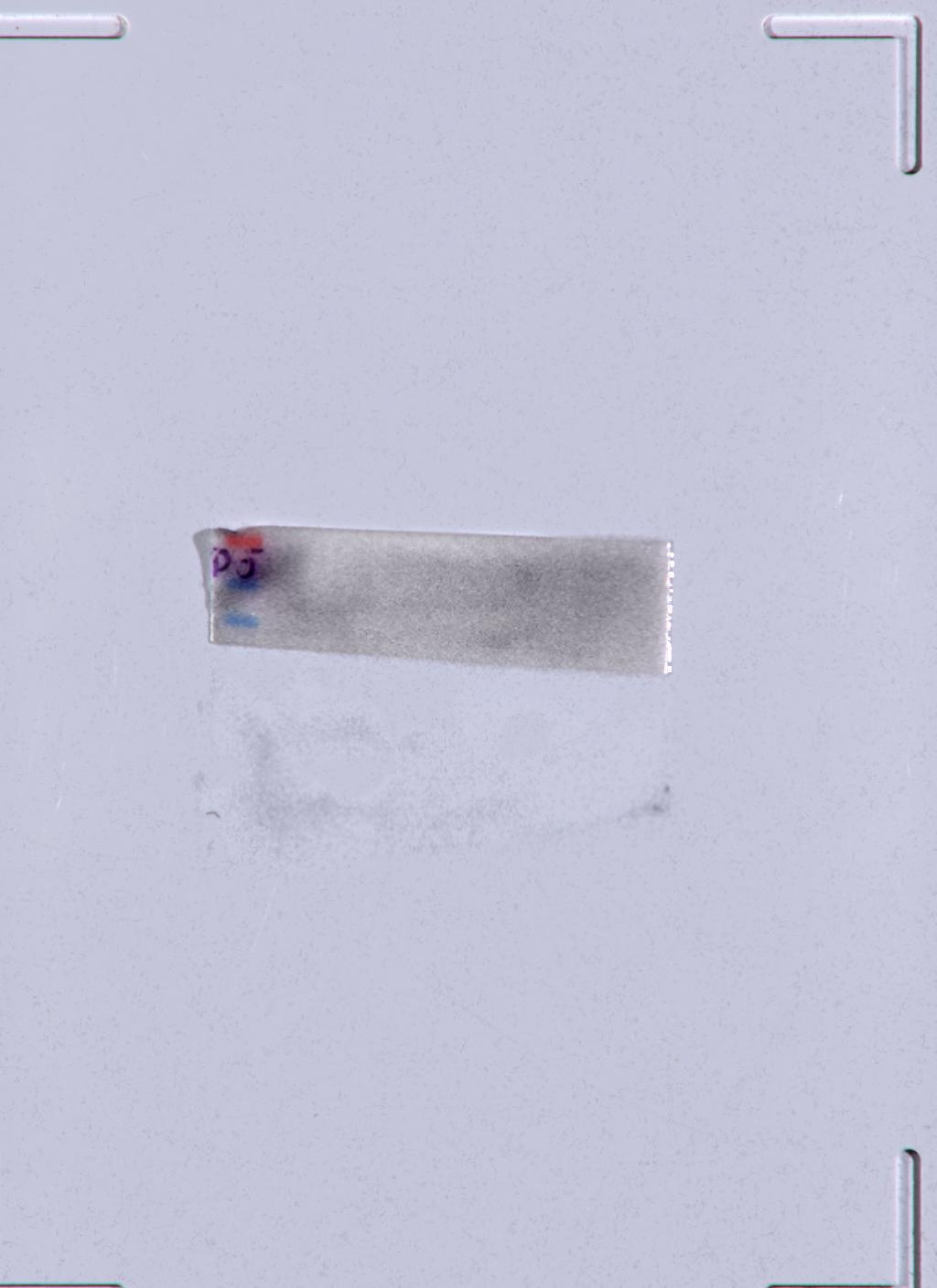

Supplement: Supplemental Information 2 [file peerj-13-19085-s002.zip › Chaetoglobosin A induces T-24 apoptosis in human bladder cancer/7.PI3K ERK pathway/p-jnk/22.5.3 p-jnk 2022.05.03_13.30.31_Ch/22.5.3 p-jnk 2022.05.03_13.30.31_Ch+Marker.jpg]

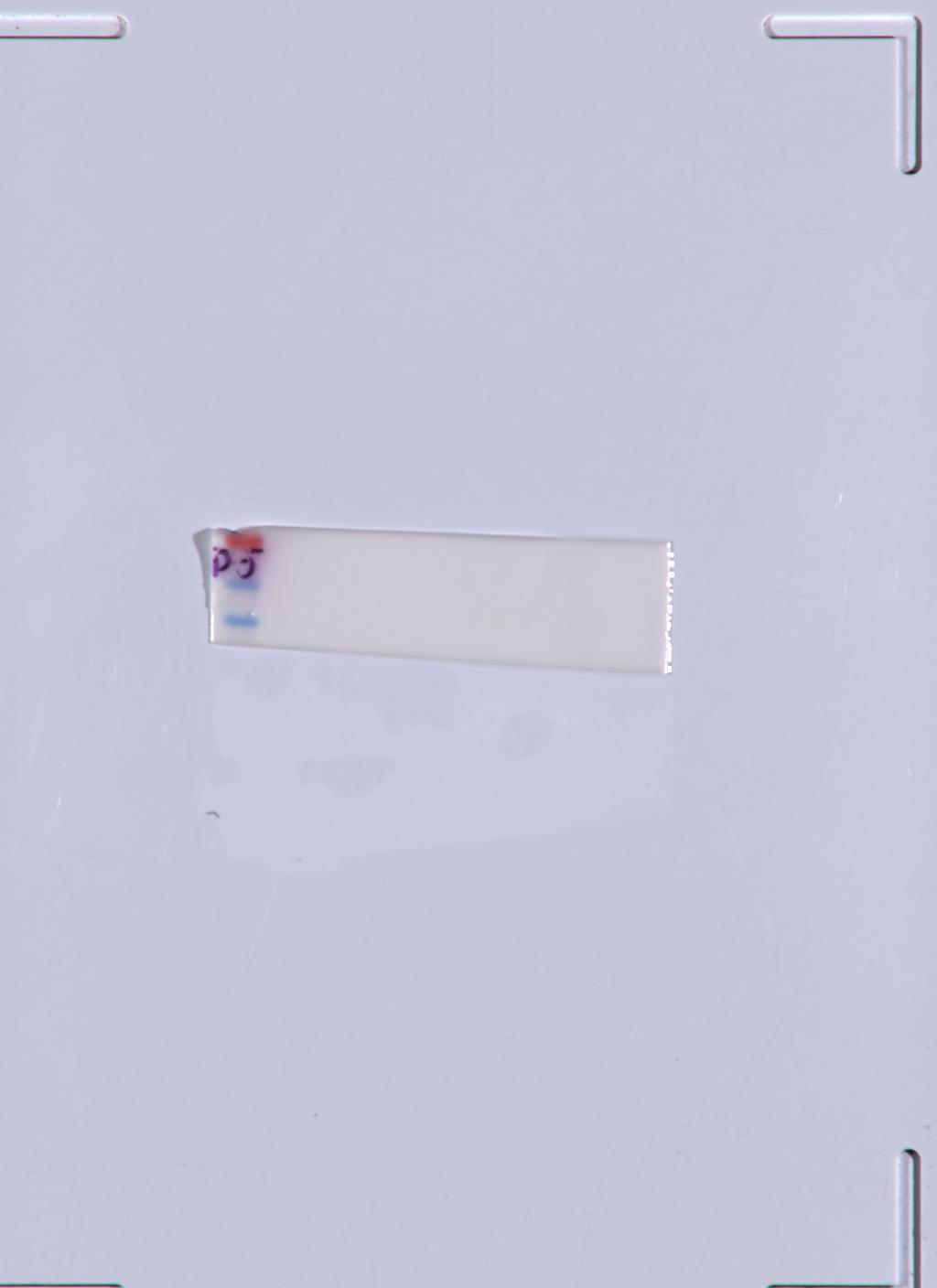

Supplement: Supplemental Information 2 [file peerj-13-19085-s002.zip › Chaetoglobosin A induces T-24 apoptosis in human bladder cancer/7.PI3K ERK pathway/p-jnk/22.5.3 p-jnk 2022.05.03_13.30.31_Ch/22.5.3 p-jnk 2022.05.03_13.30.31_Ch-Marker.jpg]

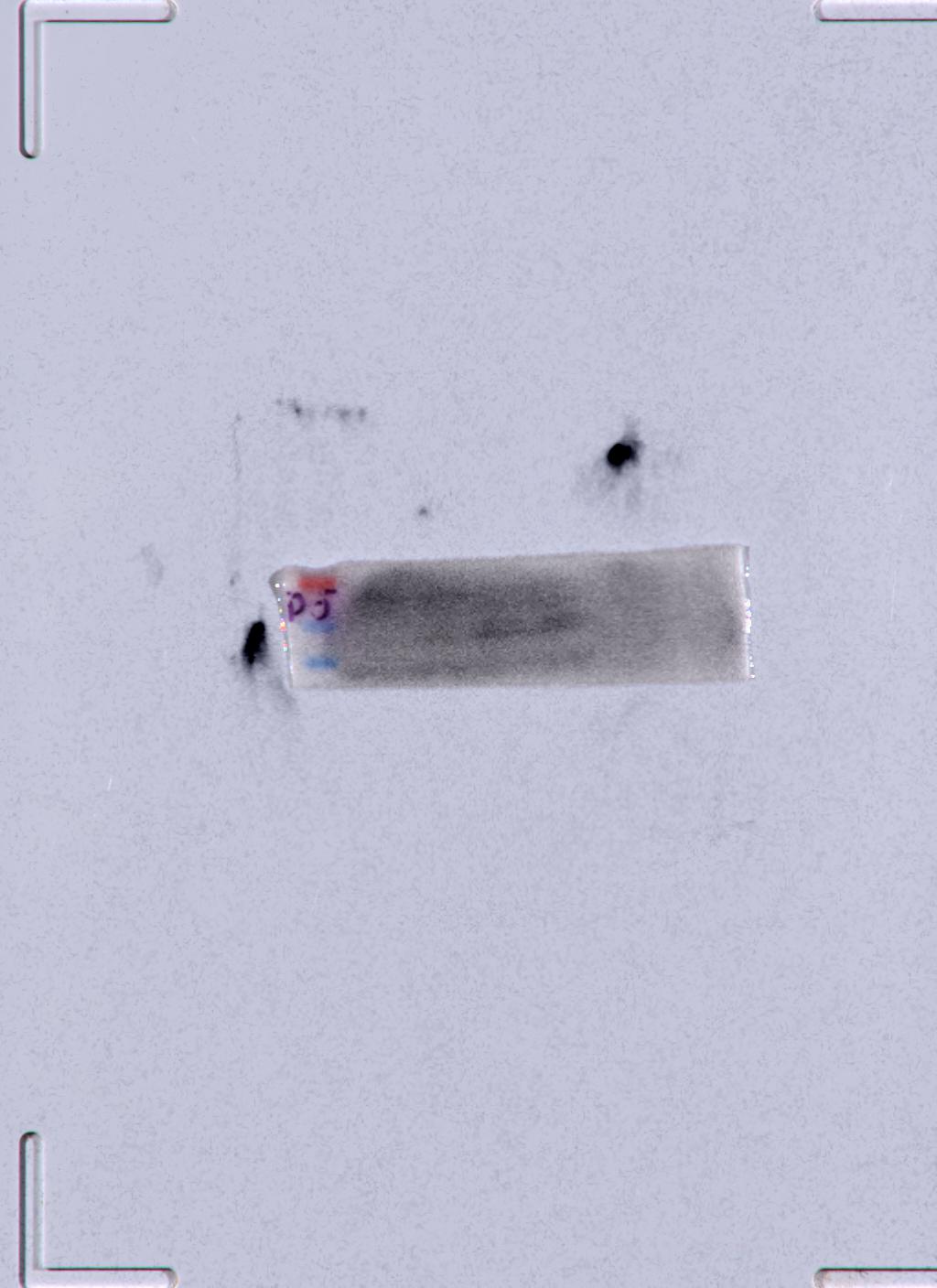

Supplement: Supplemental Information 2 [file peerj-13-19085-s002.zip › Chaetoglobosin A induces T-24 apoptosis in human bladder cancer/7.PI3K ERK pathway/p-jnk/22.5.3 p-jnk11 2022.05.03_14.53.11_Ch/22.5.3 p-jnk11 2022.05.03_14.53.11_Ch+Marker.jpg]

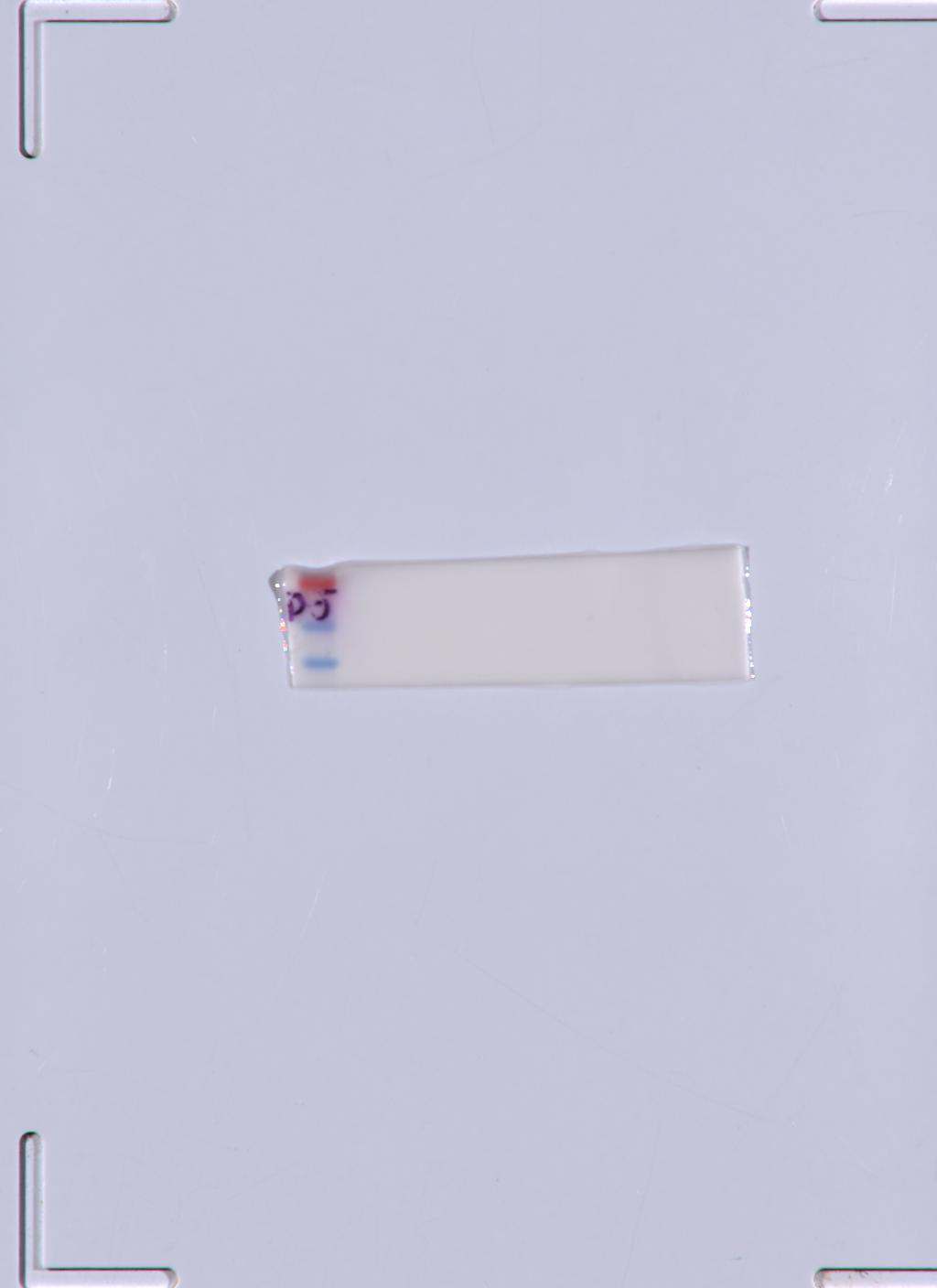

Supplement: Supplemental Information 2 [file peerj-13-19085-s002.zip › Chaetoglobosin A induces T-24 apoptosis in human bladder cancer/7.PI3K ERK pathway/p-jnk/22.5.3 p-jnk11 2022.05.03_14.53.11_Ch/22.5.3 p-jnk11 2022.05.03_14.53.11_Ch-Marker.jpg]

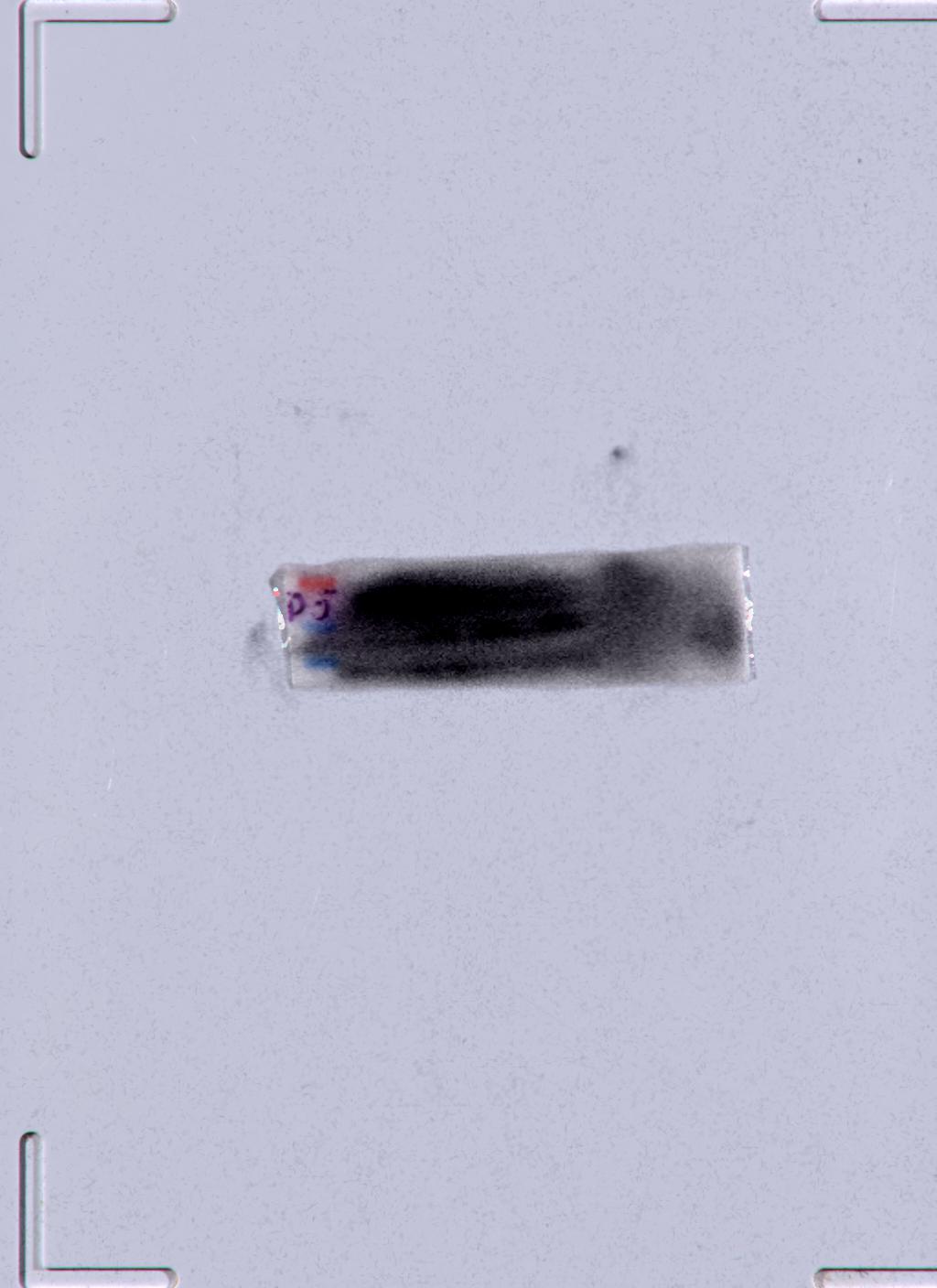

Supplement: Supplemental Information 2 [file peerj-13-19085-s002.zip › Chaetoglobosin A induces T-24 apoptosis in human bladder cancer/7.PI3K ERK pathway/p-jnk/22.5.3 p-jnk12 2022.05.03_14.55.32_Ch/22.5.3 p-jnk12 2022.05.03_14.55.32_Ch+Marker.jpg]

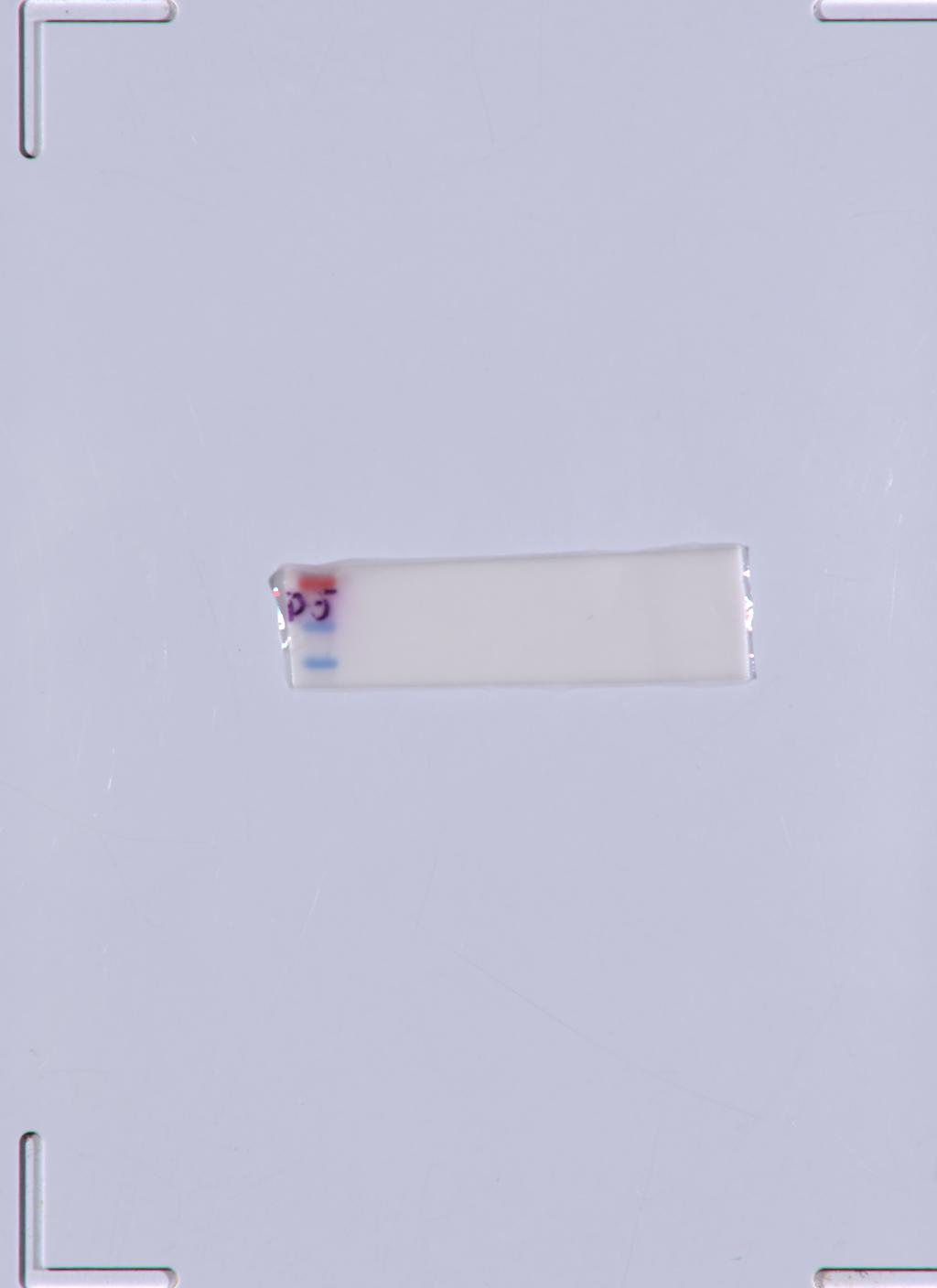

Supplement: Supplemental Information 2 [file peerj-13-19085-s002.zip › Chaetoglobosin A induces T-24 apoptosis in human bladder cancer/7.PI3K ERK pathway/p-jnk/22.5.3 p-jnk12 2022.05.03_14.55.32_Ch/22.5.3 p-jnk12 2022.05.03_14.55.32_Ch-Marker.jpg]

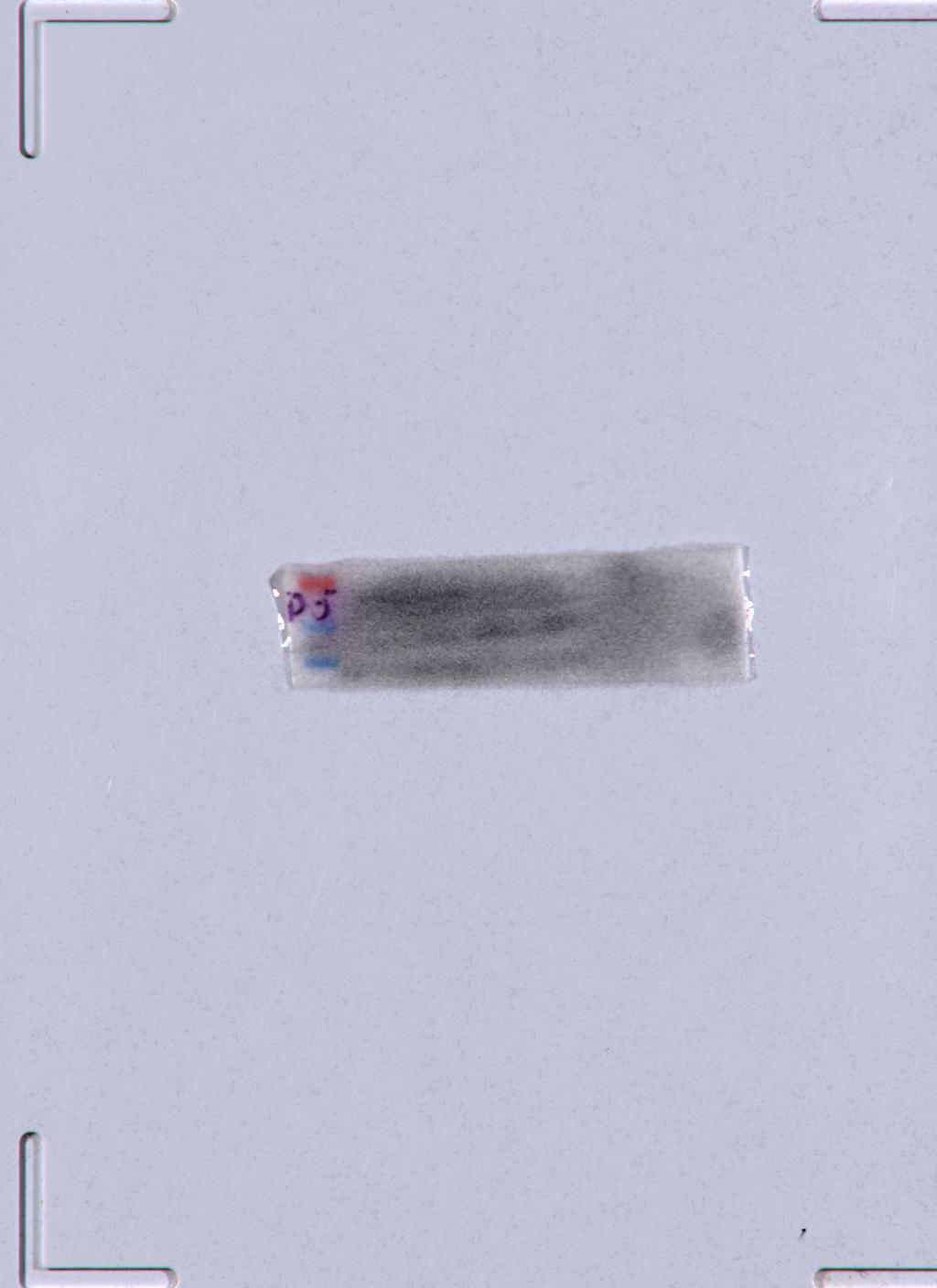

Supplement: Supplemental Information 2 [file peerj-13-19085-s002.zip › Chaetoglobosin A induces T-24 apoptosis in human bladder cancer/7.PI3K ERK pathway/p-jnk/22.5.3 p-jnk13 2022.05.03_14.58.06_Ch/22.5.3 p-jnk13 2022.05.03_14.58.06_Ch+Marker.jpg]

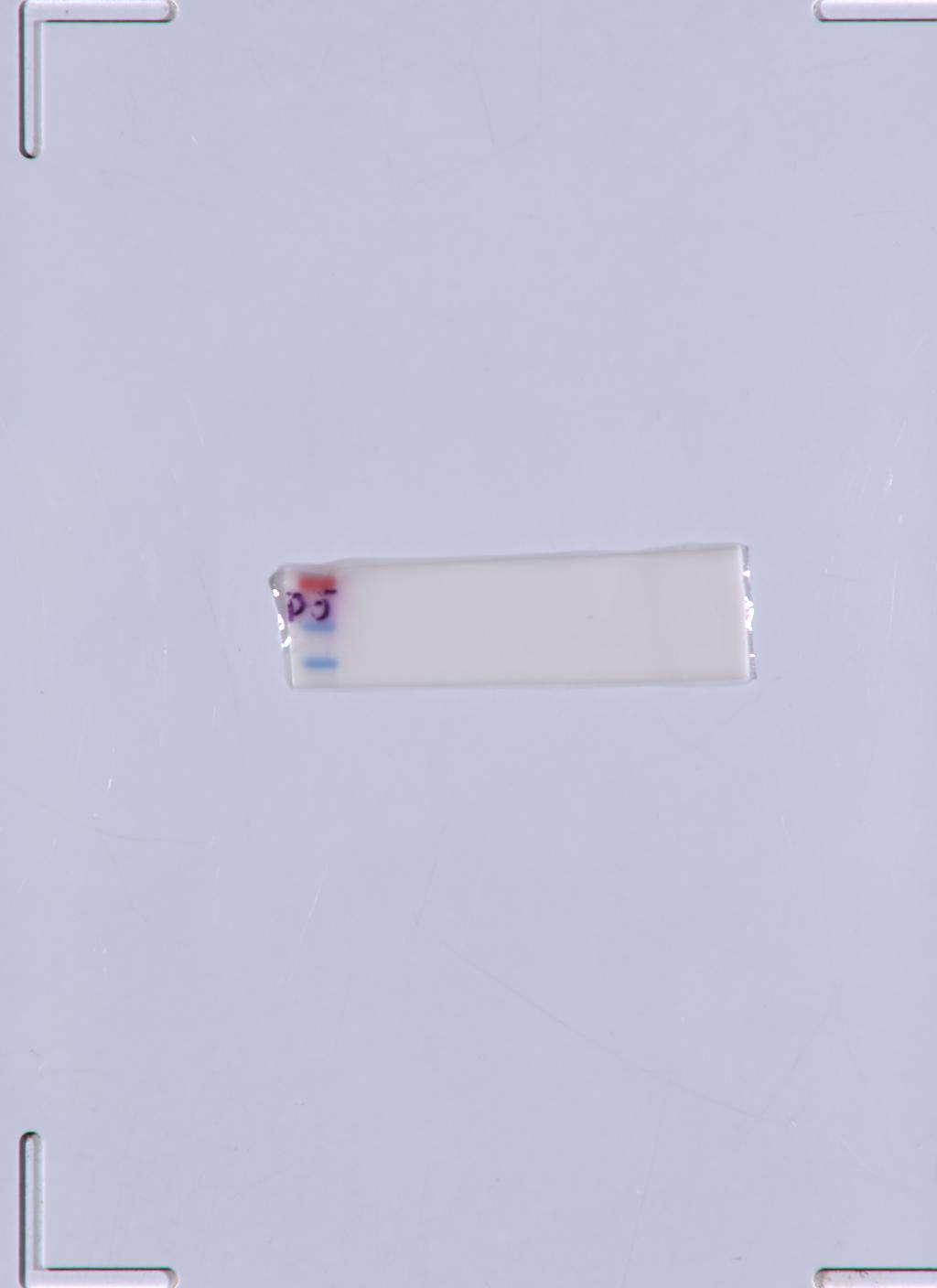

Supplement: Supplemental Information 2 [file peerj-13-19085-s002.zip › Chaetoglobosin A induces T-24 apoptosis in human bladder cancer/7.PI3K ERK pathway/p-jnk/22.5.3 p-jnk13 2022.05.03_14.58.06_Ch/22.5.3 p-jnk13 2022.05.03_14.58.06_Ch-Marker.jpg]

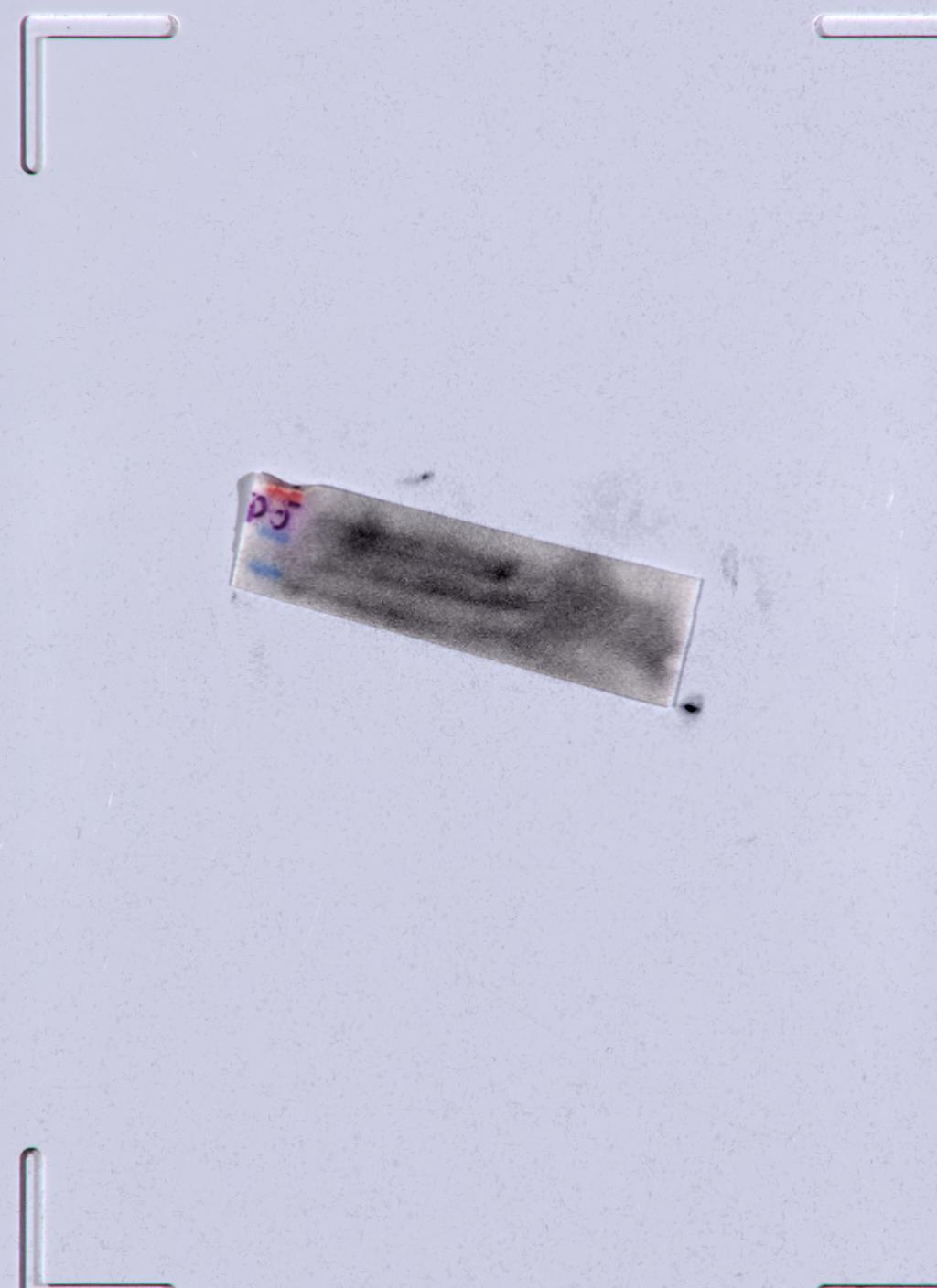

Supplement: Supplemental Information 2 [file peerj-13-19085-s002.zip › Chaetoglobosin A induces T-24 apoptosis in human bladder cancer/7.PI3K ERK pathway/p-jnk/22.5.3 p-jnk14 2022.05.03_15.01.34_Ch/22.5.3 p-jnk14 2022.05.03_15.01.34_Ch+Marker.jpg]

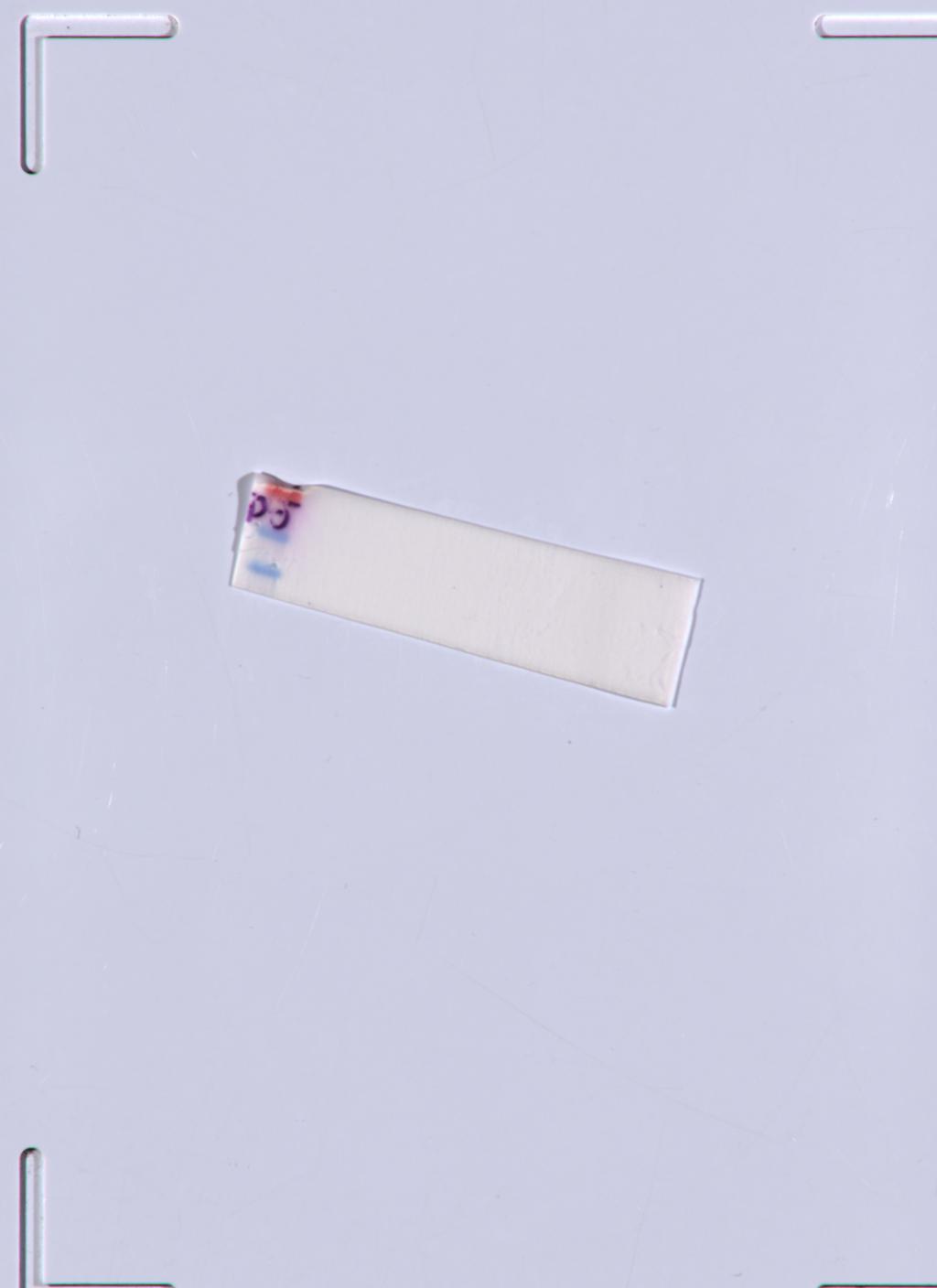

Supplement: Supplemental Information 2 [file peerj-13-19085-s002.zip › Chaetoglobosin A induces T-24 apoptosis in human bladder cancer/7.PI3K ERK pathway/p-jnk/22.5.3 p-jnk14 2022.05.03_15.01.34_Ch/22.5.3 p-jnk14 2022.05.03_15.01.34_Ch-Marker.jpg]

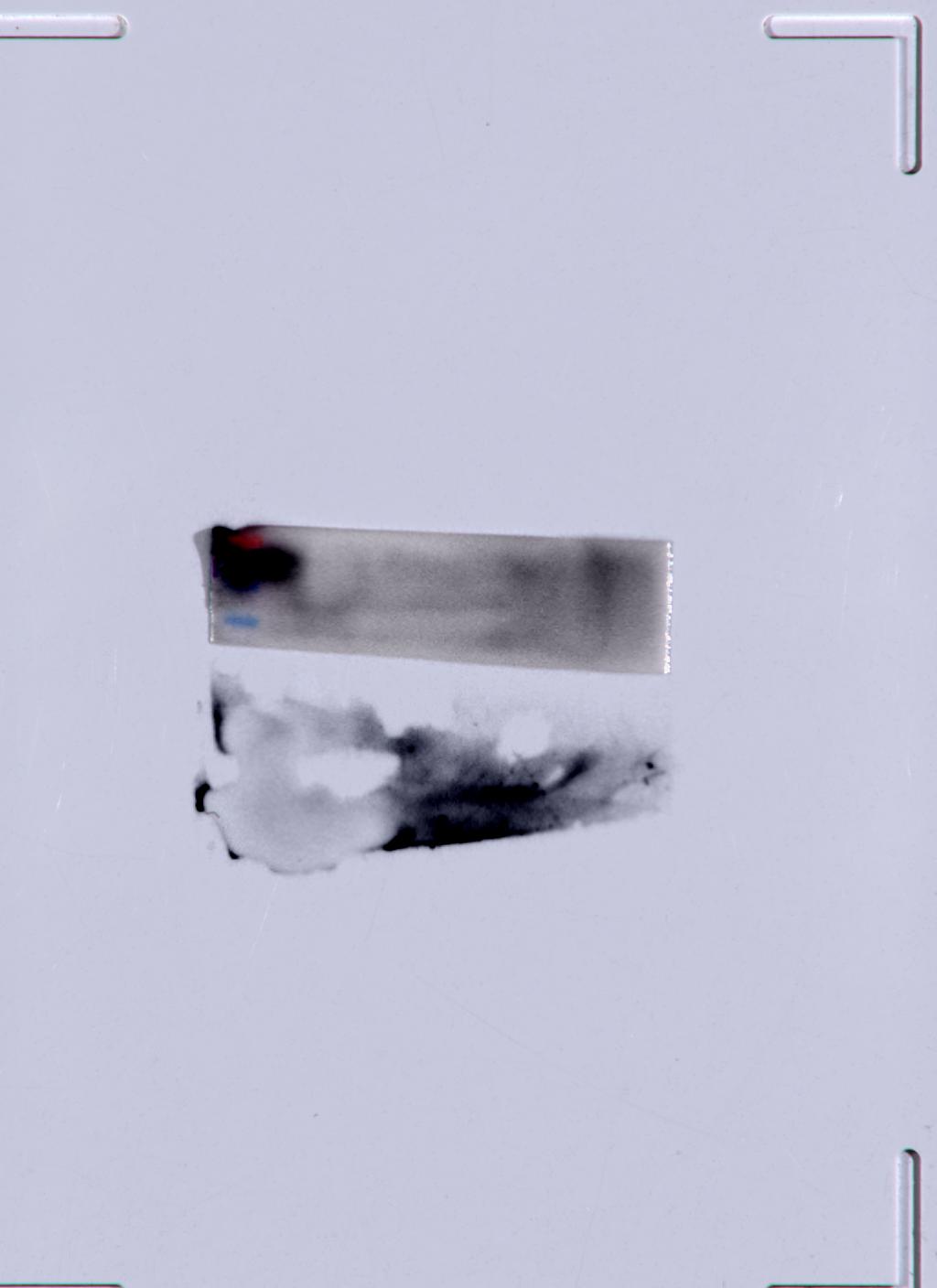

Supplement: Supplemental Information 2 [file peerj-13-19085-s002.zip › Chaetoglobosin A induces T-24 apoptosis in human bladder cancer/7.PI3K ERK pathway/p-jnk/22.5.3 p-jnk2 2022.05.03_13.32.47_Ch/22.5.3 p-jnk2 2022.05.03_13.32.47_Ch+Marker.jpg]

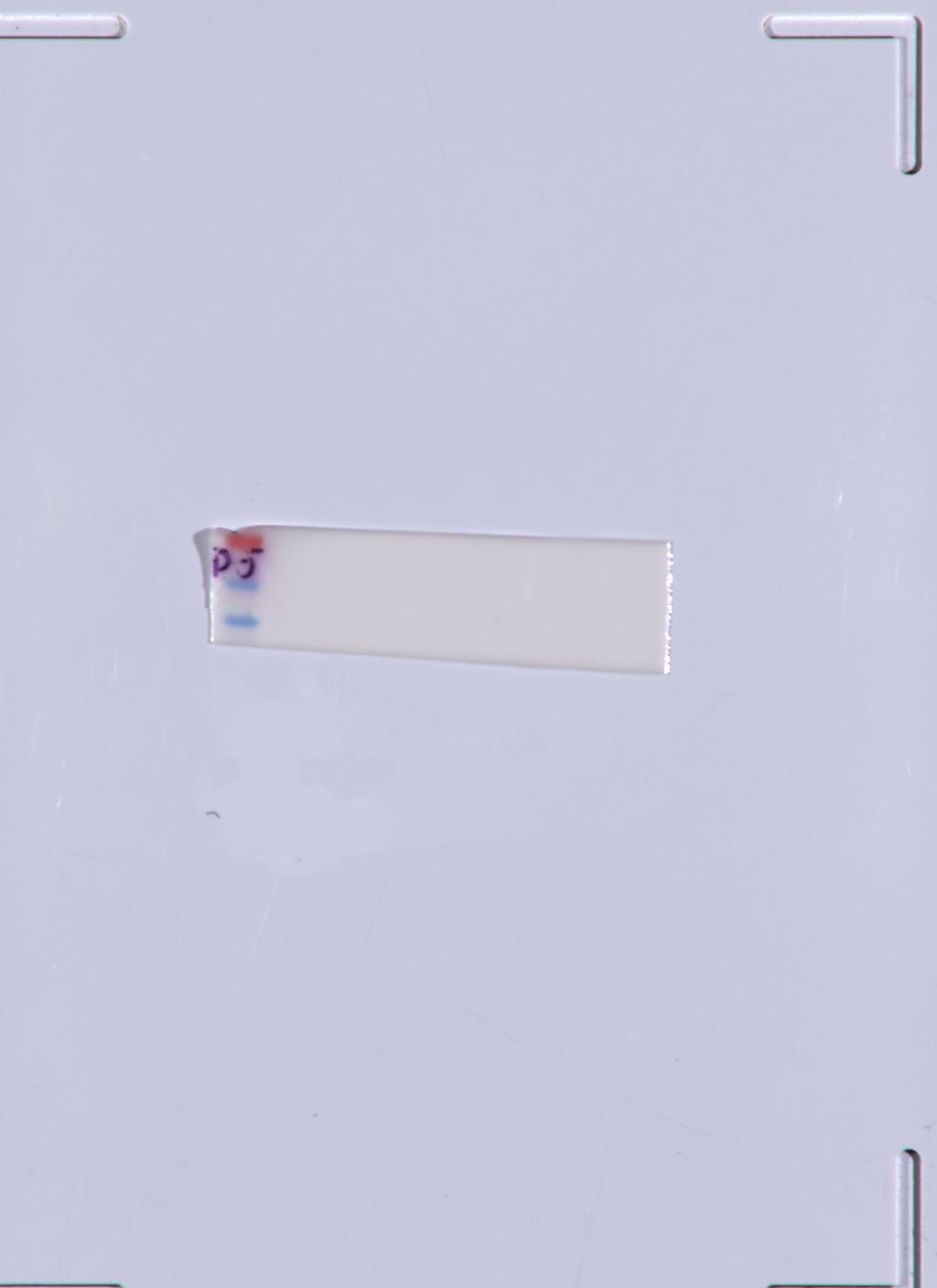

Supplement: Supplemental Information 2 [file peerj-13-19085-s002.zip › Chaetoglobosin A induces T-24 apoptosis in human bladder cancer/7.PI3K ERK pathway/p-jnk/22.5.3 p-jnk2 2022.05.03_13.32.47_Ch/22.5.3 p-jnk2 2022.05.03_13.32.47_Ch-Marker.jpg]

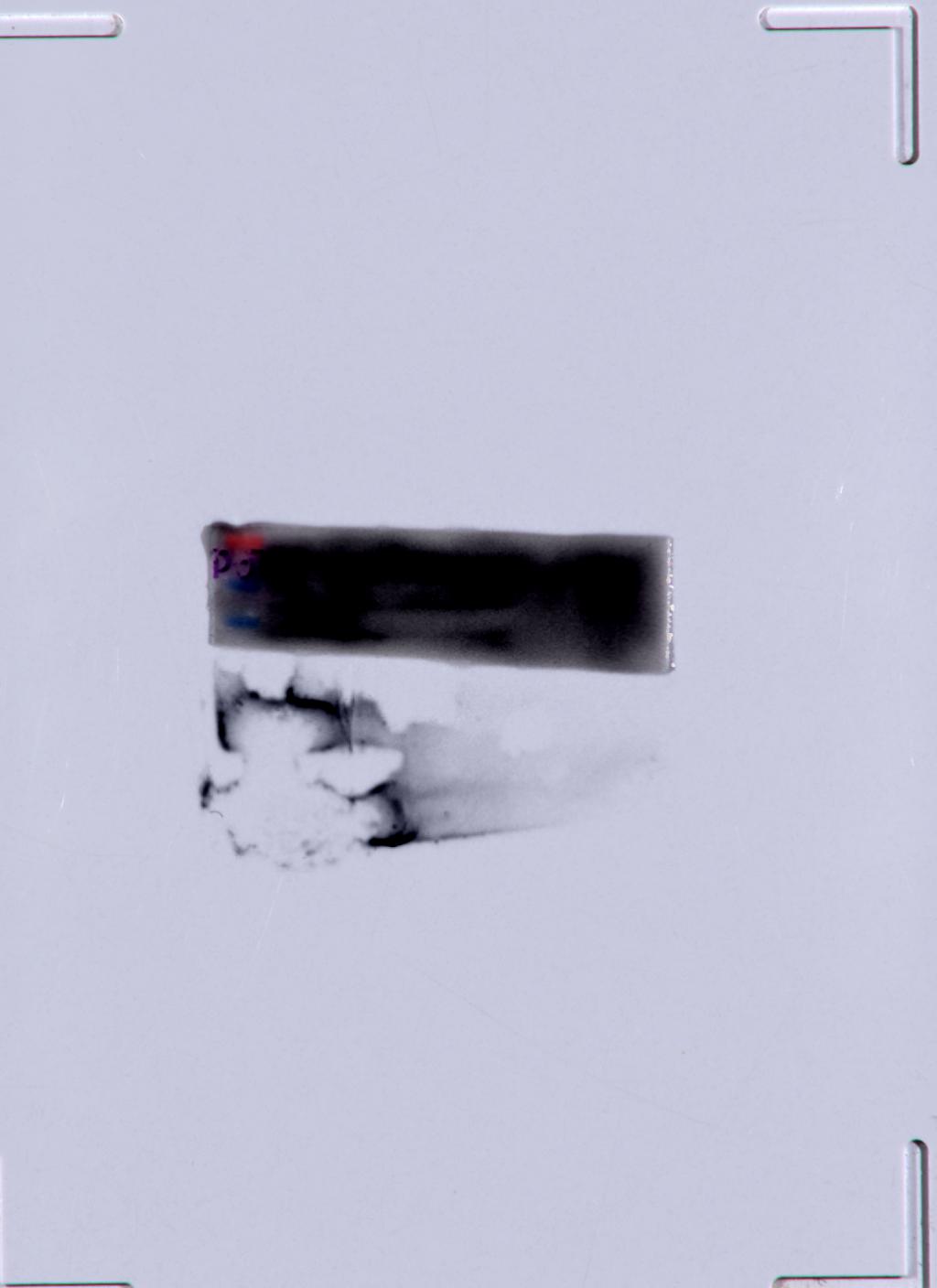

Supplement: Supplemental Information 2 [file peerj-13-19085-s002.zip › Chaetoglobosin A induces T-24 apoptosis in human bladder cancer/7.PI3K ERK pathway/p-jnk/22.5.3 p-jnk3 2022.05.03_13.38.16_Ch/22.5.3 p-jnk3 2022.05.03_13.38.16_Ch+Marker.jpg]

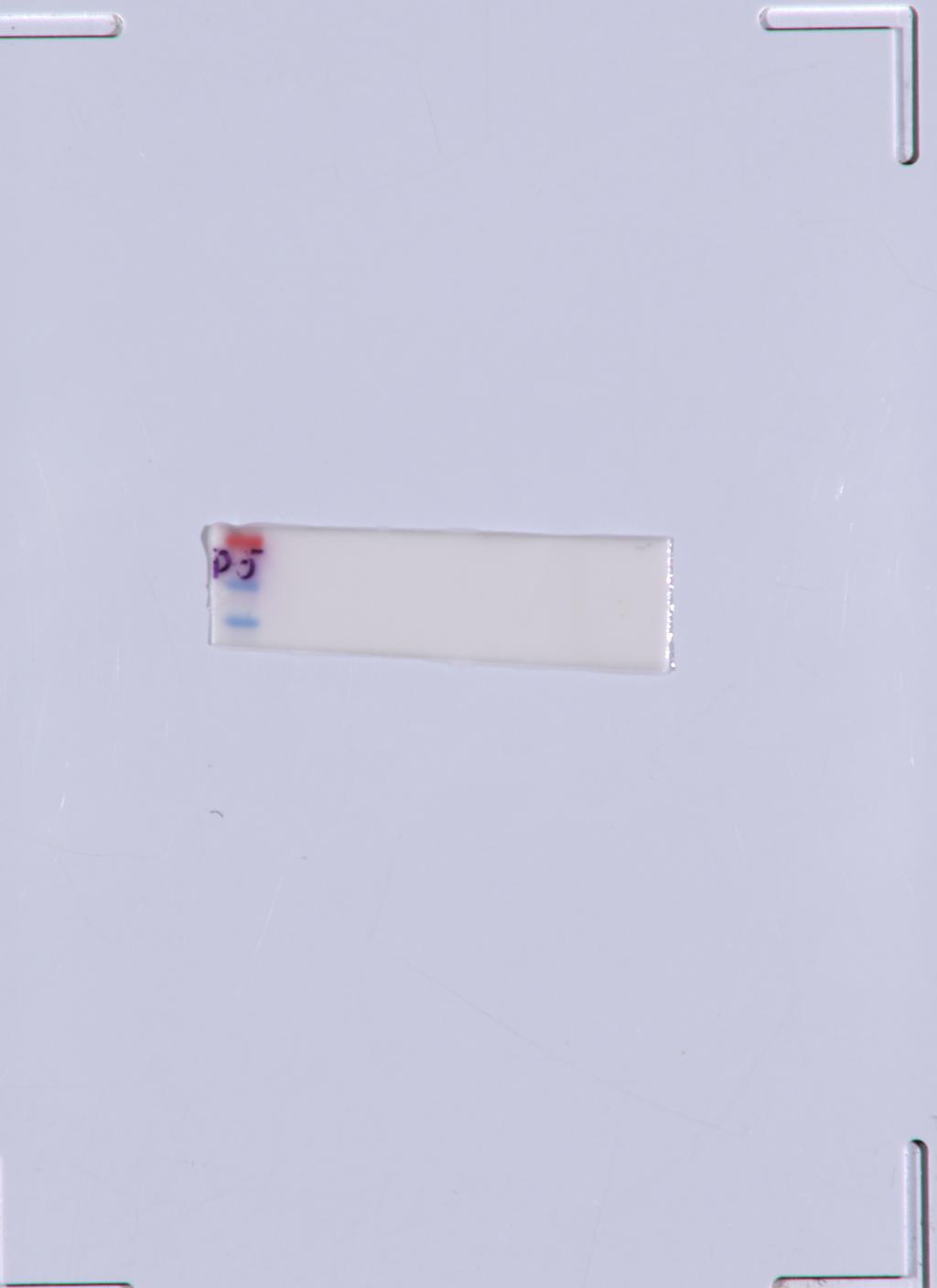

Supplement: Supplemental Information 2 [file peerj-13-19085-s002.zip › Chaetoglobosin A induces T-24 apoptosis in human bladder cancer/7.PI3K ERK pathway/p-jnk/22.5.3 p-jnk3 2022.05.03_13.38.16_Ch/22.5.3 p-jnk3 2022.05.03_13.38.16_Ch-Marker.jpg]

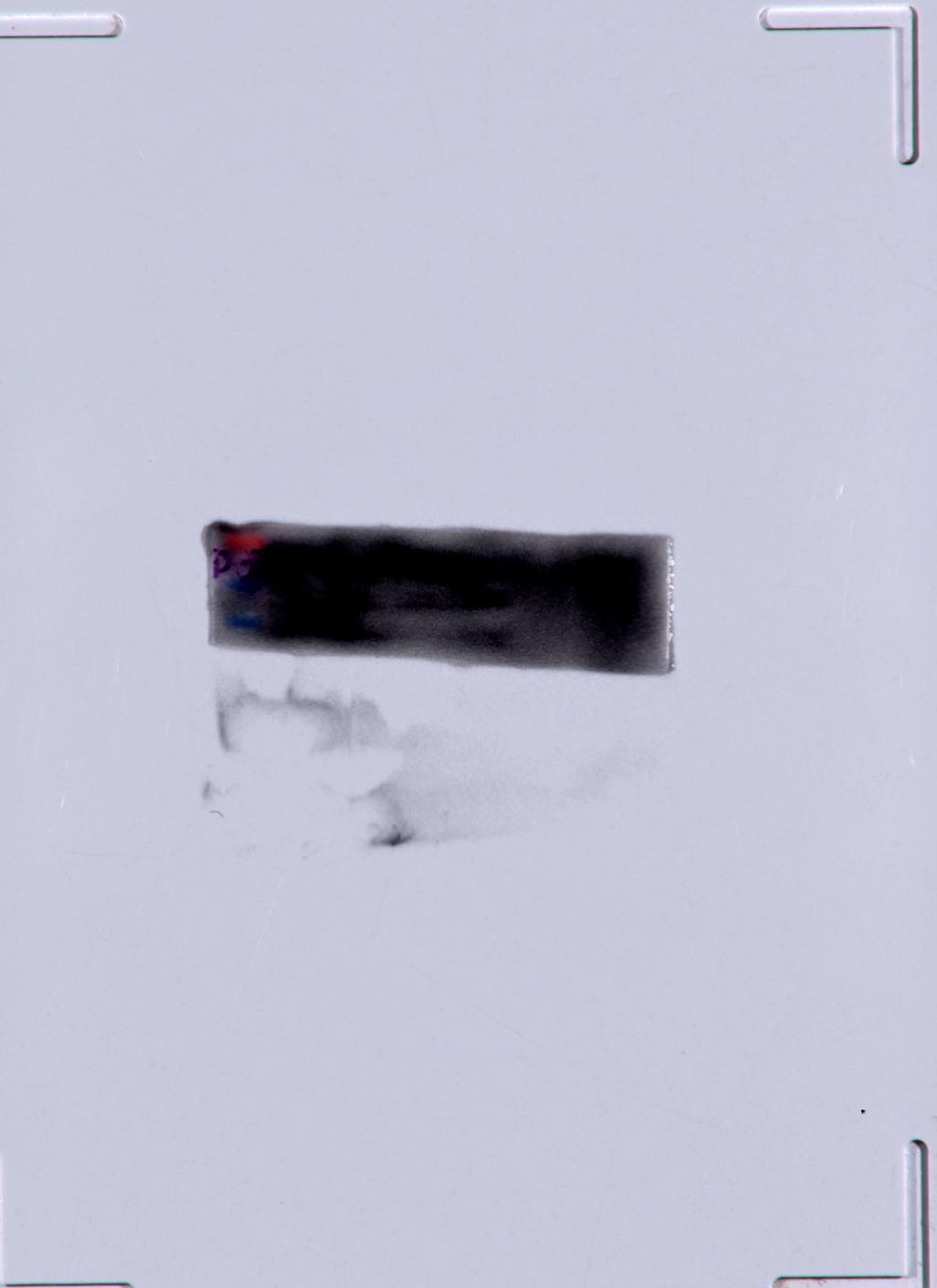

Supplement: Supplemental Information 2 [file peerj-13-19085-s002.zip › Chaetoglobosin A induces T-24 apoptosis in human bladder cancer/7.PI3K ERK pathway/p-jnk/22.5.3 p-jnk4 2022.05.03_13.43.13_Ch/22.5.3 p-jnk4 2022.05.03_13.43.13_Ch+Marker.jpg]

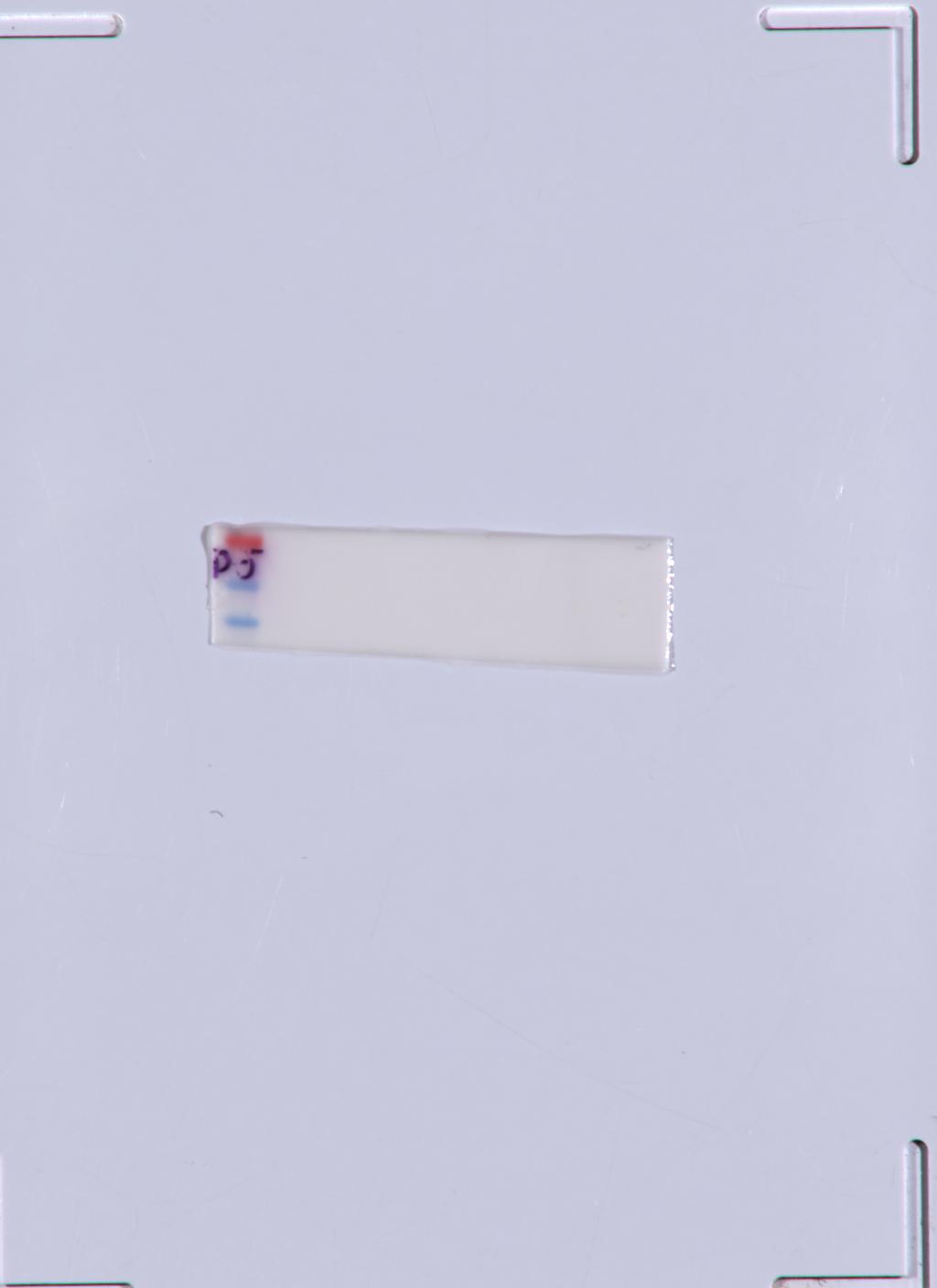

Supplement: Supplemental Information 2 [file peerj-13-19085-s002.zip › Chaetoglobosin A induces T-24 apoptosis in human bladder cancer/7.PI3K ERK pathway/p-jnk/22.5.3 p-jnk4 2022.05.03_13.43.13_Ch/22.5.3 p-jnk4 2022.05.03_13.43.13_Ch-Marker.jpg]

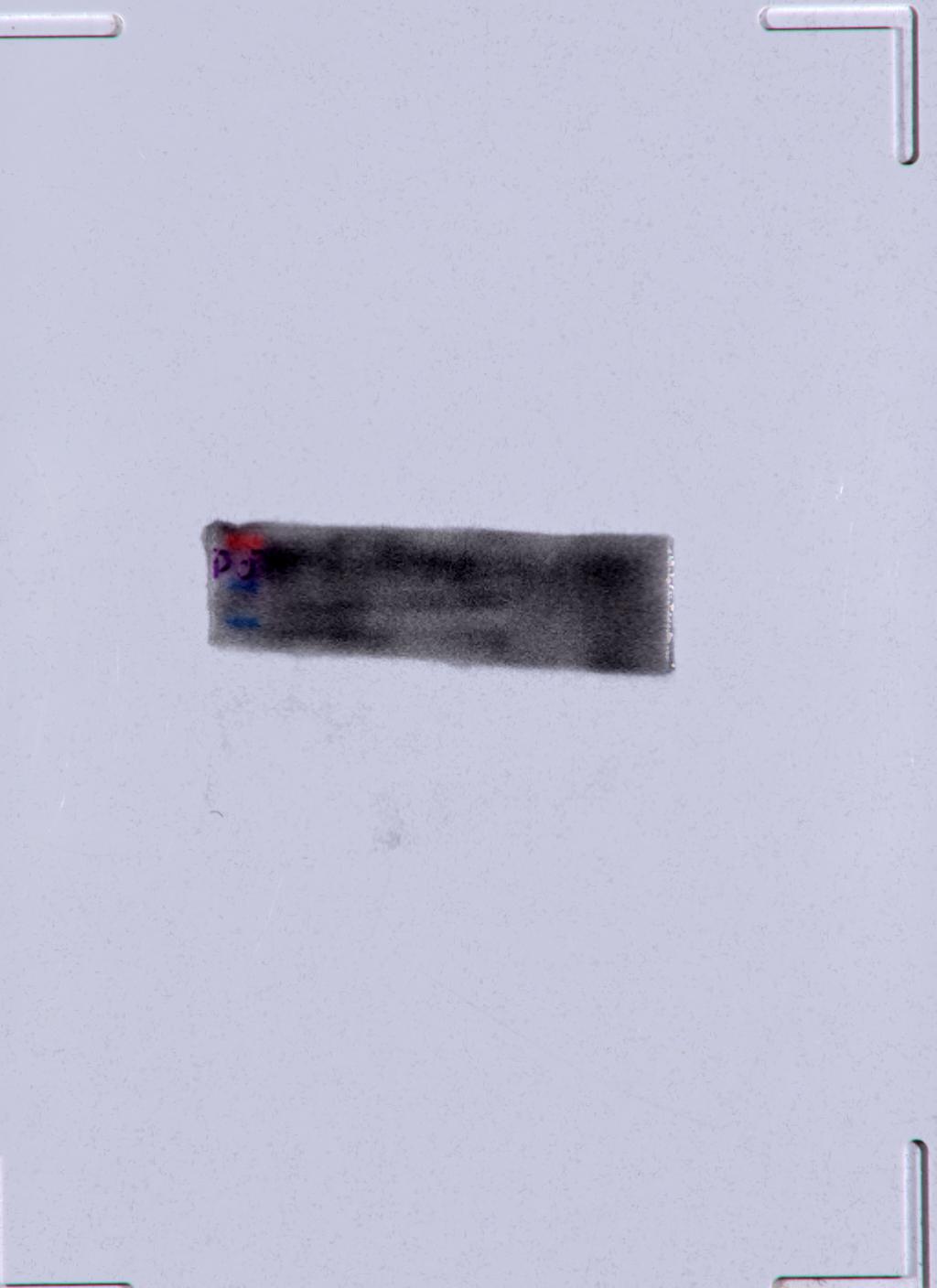

Supplement: Supplemental Information 2 [file peerj-13-19085-s002.zip › Chaetoglobosin A induces T-24 apoptosis in human bladder cancer/7.PI3K ERK pathway/p-jnk/22.5.3 p-jnk5 2022.05.03_13.49.26_Ch/22.5.3 p-jnk5 2022.05.03_13.49.26_Ch+Marker.jpg]

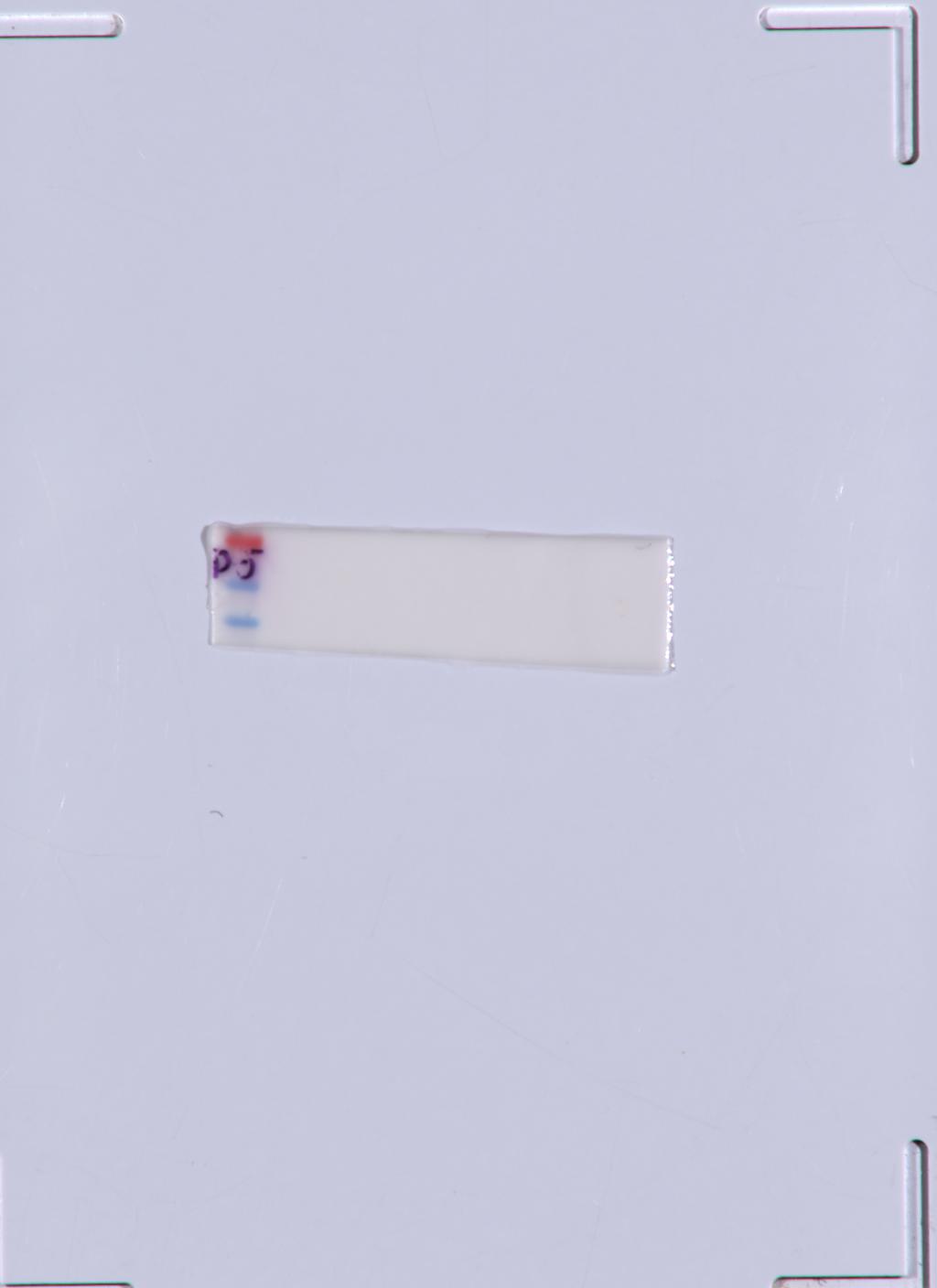

Supplement: Supplemental Information 2 [file peerj-13-19085-s002.zip › Chaetoglobosin A induces T-24 apoptosis in human bladder cancer/7.PI3K ERK pathway/p-jnk/22.5.3 p-jnk5 2022.05.03_13.49.26_Ch/22.5.3 p-jnk5 2022.05.03_13.49.26_Ch-Marker.jpg]

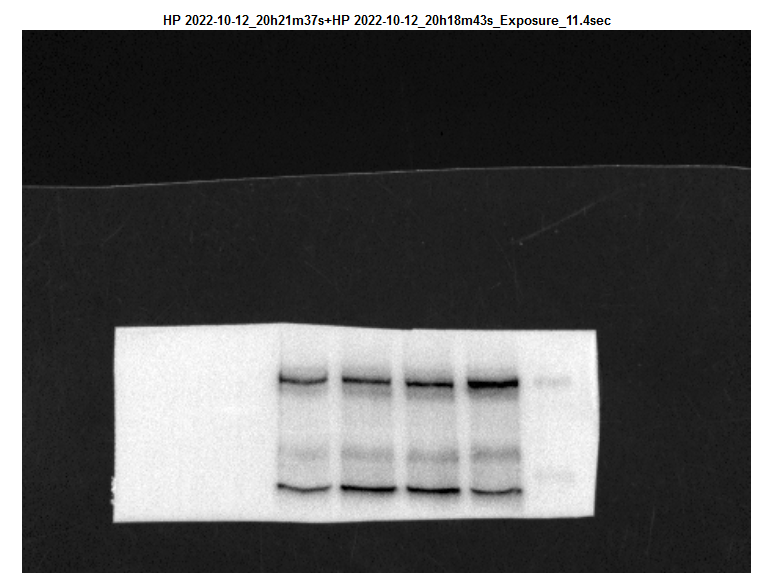

Supplement: Supplemental Information 2 [file peerj-13-19085-s002.zip › Chaetoglobosin A induces T-24 apoptosis in human bladder cancer/7.PI3K ERK pathway/p-mTOR/HP 2022-10-12_20h21m37s+HP 2022-10-12_20h18m43s_Exposure_11.4sec.tif]

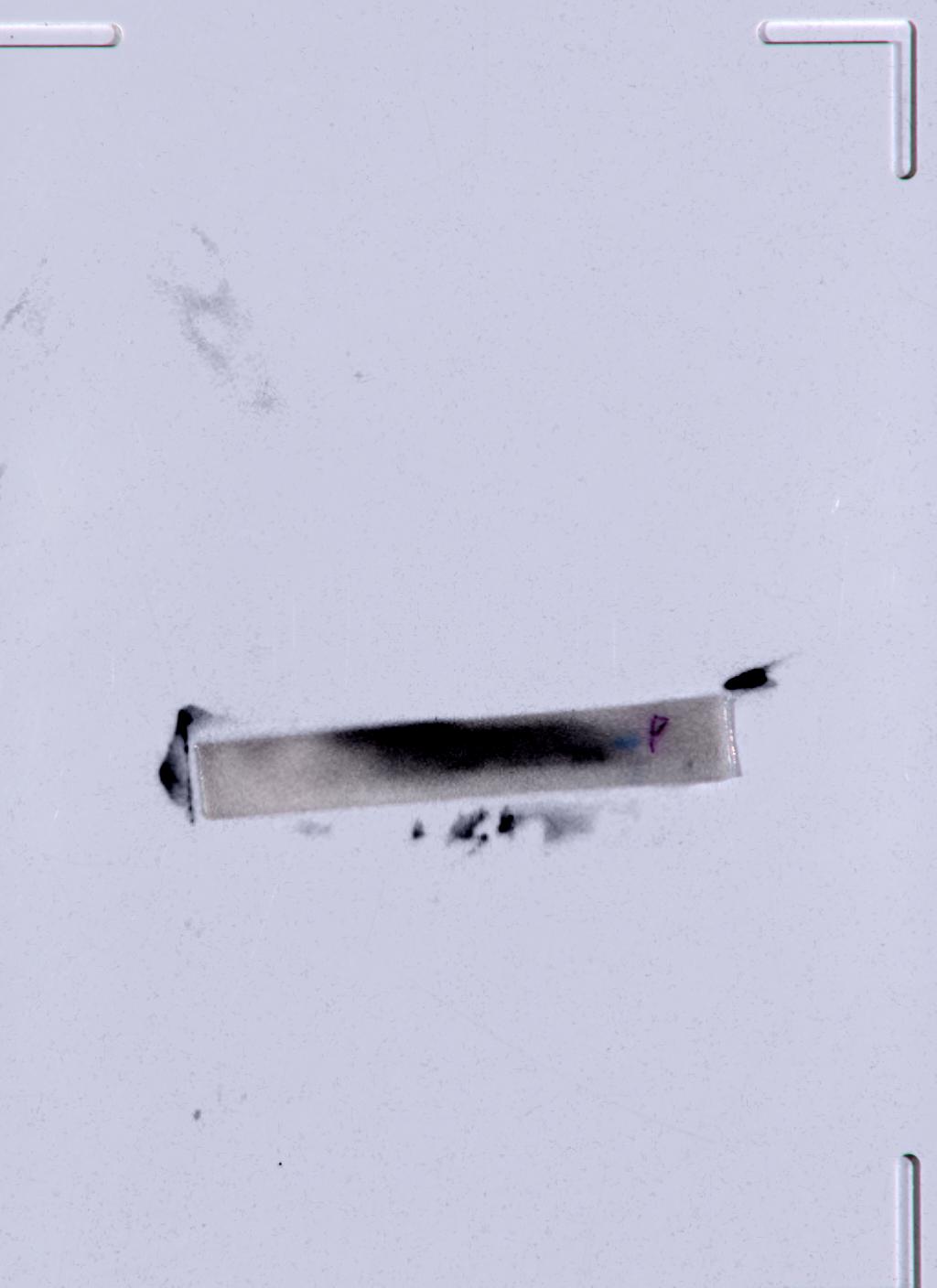

Supplement: Supplemental Information 2 [file peerj-13-19085-s002.zip › Chaetoglobosin A induces T-24 apoptosis in human bladder cancer/7.PI3K ERK pathway/p-p38/22.4.18 p-p38 2022.04.18_13.52.54_Ch/22.4.18 p-p38 2022.04.18_13.52.54_Ch+Marker.jpg]

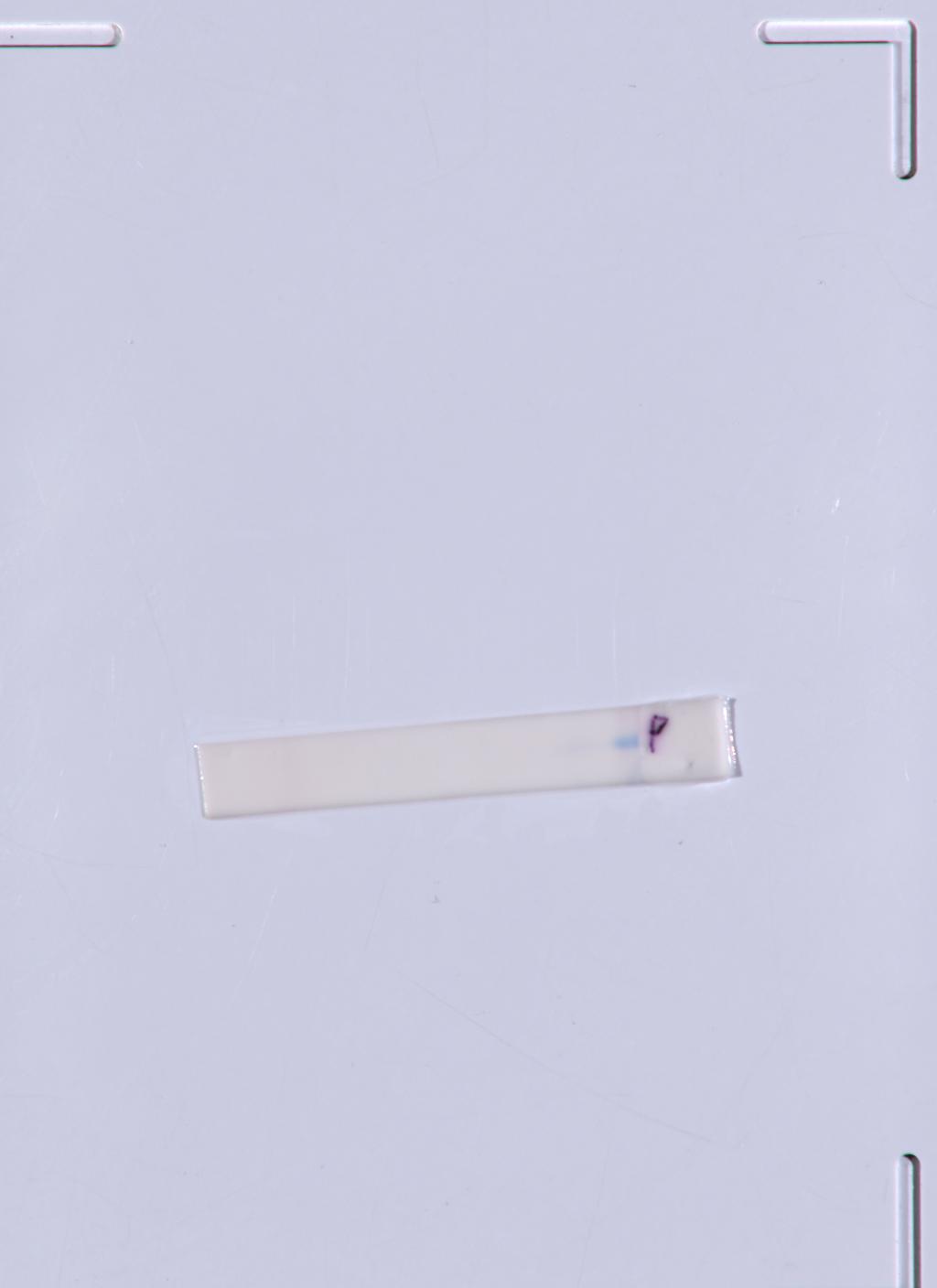

Supplement: Supplemental Information 2 [file peerj-13-19085-s002.zip › Chaetoglobosin A induces T-24 apoptosis in human bladder cancer/7.PI3K ERK pathway/p-p38/22.4.18 p-p38 2022.04.18_13.52.54_Ch/22.4.18 p-p38 2022.04.18_13.52.54_Ch-Marker.jpg]

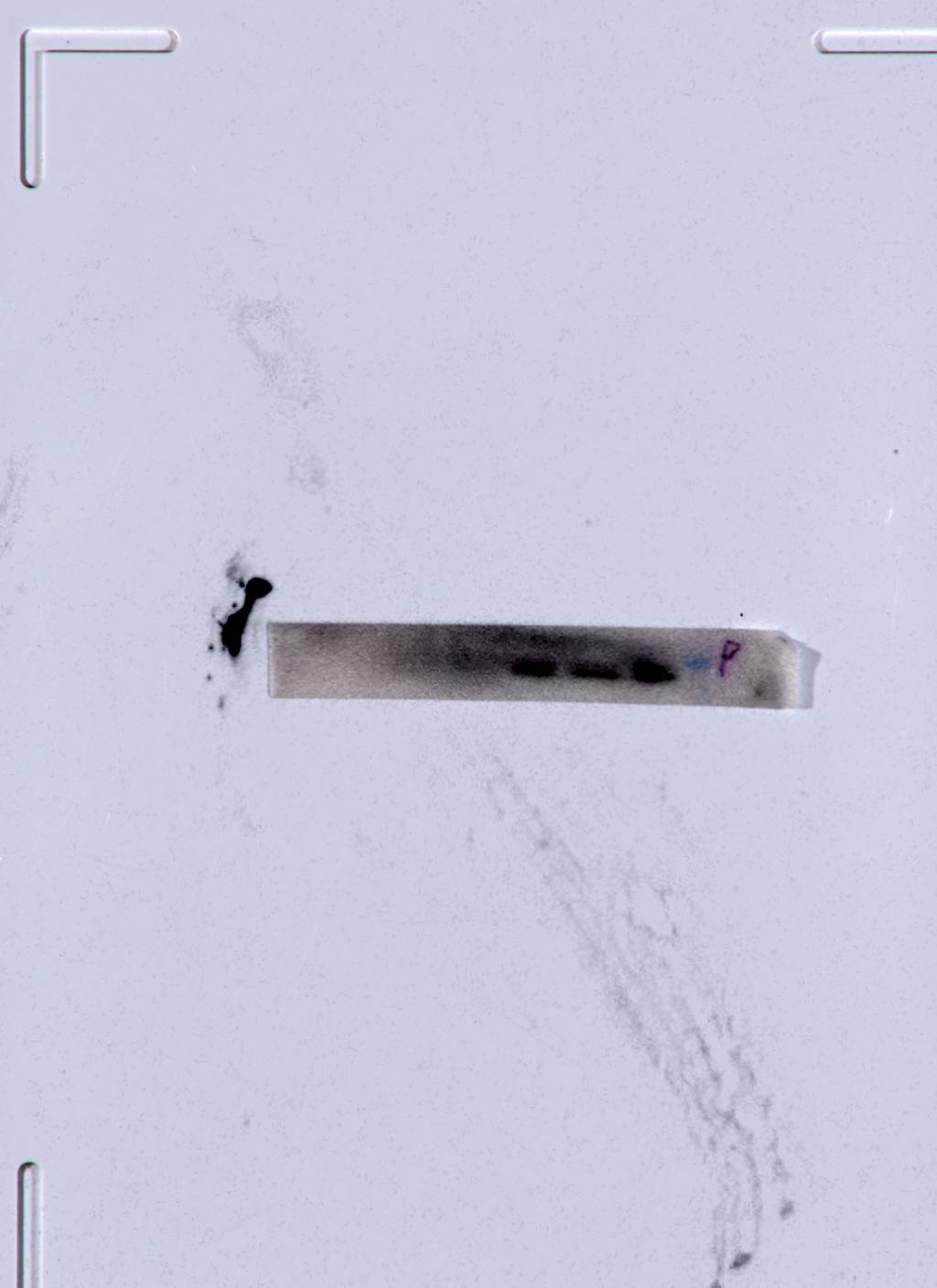

Supplement: Supplemental Information 2 [file peerj-13-19085-s002.zip › Chaetoglobosin A induces T-24 apoptosis in human bladder cancer/7.PI3K ERK pathway/p-p38/22.4.18 p-p38.2 2022.04.18_14.02.20_Ch/22.4.18 p-p38.2 2022.04.18_14.02.20_Ch+Marker.jpg]

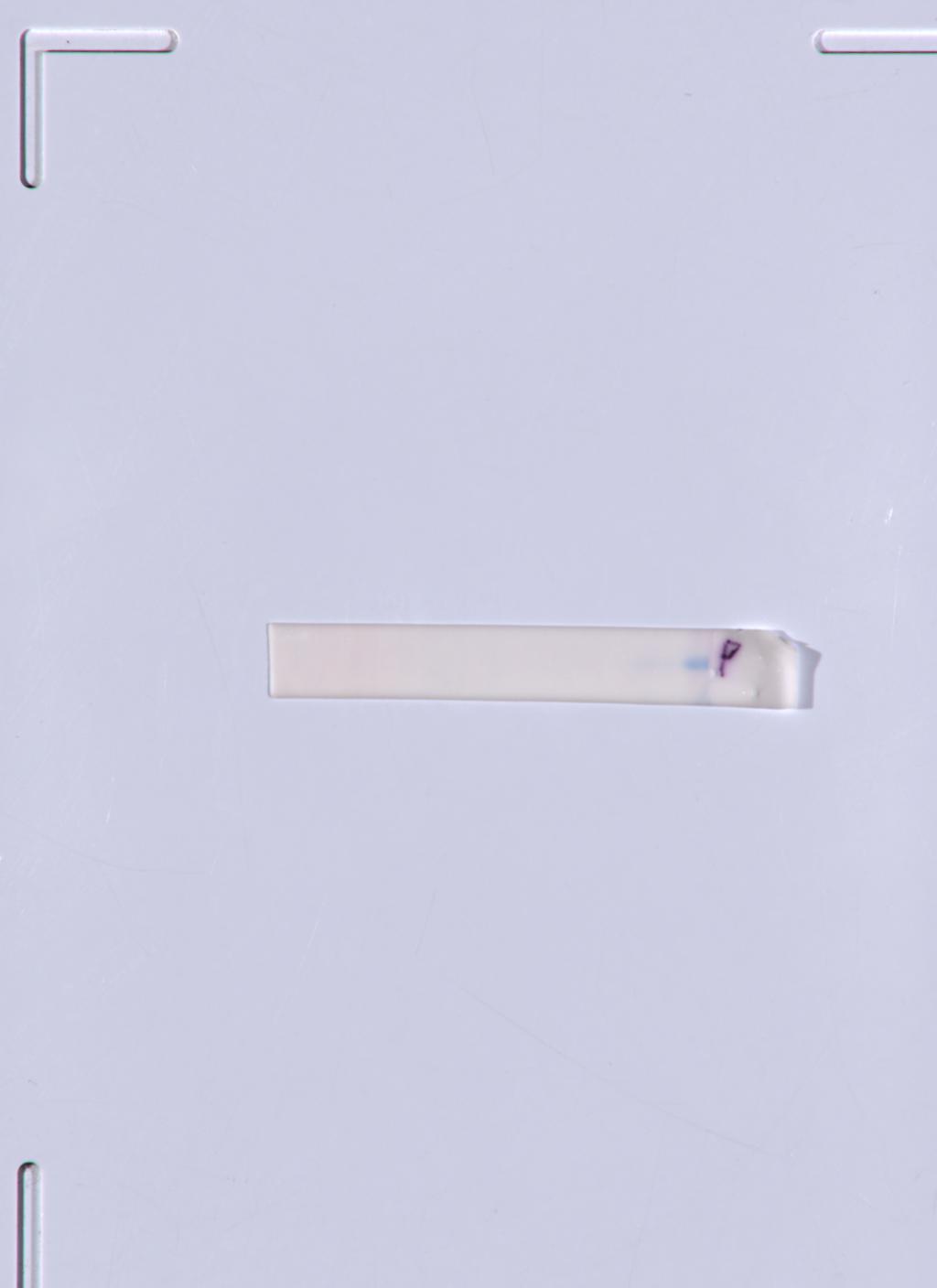

Supplement: Supplemental Information 2 [file peerj-13-19085-s002.zip › Chaetoglobosin A induces T-24 apoptosis in human bladder cancer/7.PI3K ERK pathway/p-p38/22.4.18 p-p38.2 2022.04.18_14.02.20_Ch/22.4.18 p-p38.2 2022.04.18_14.02.20_Ch-Marker.jpg]

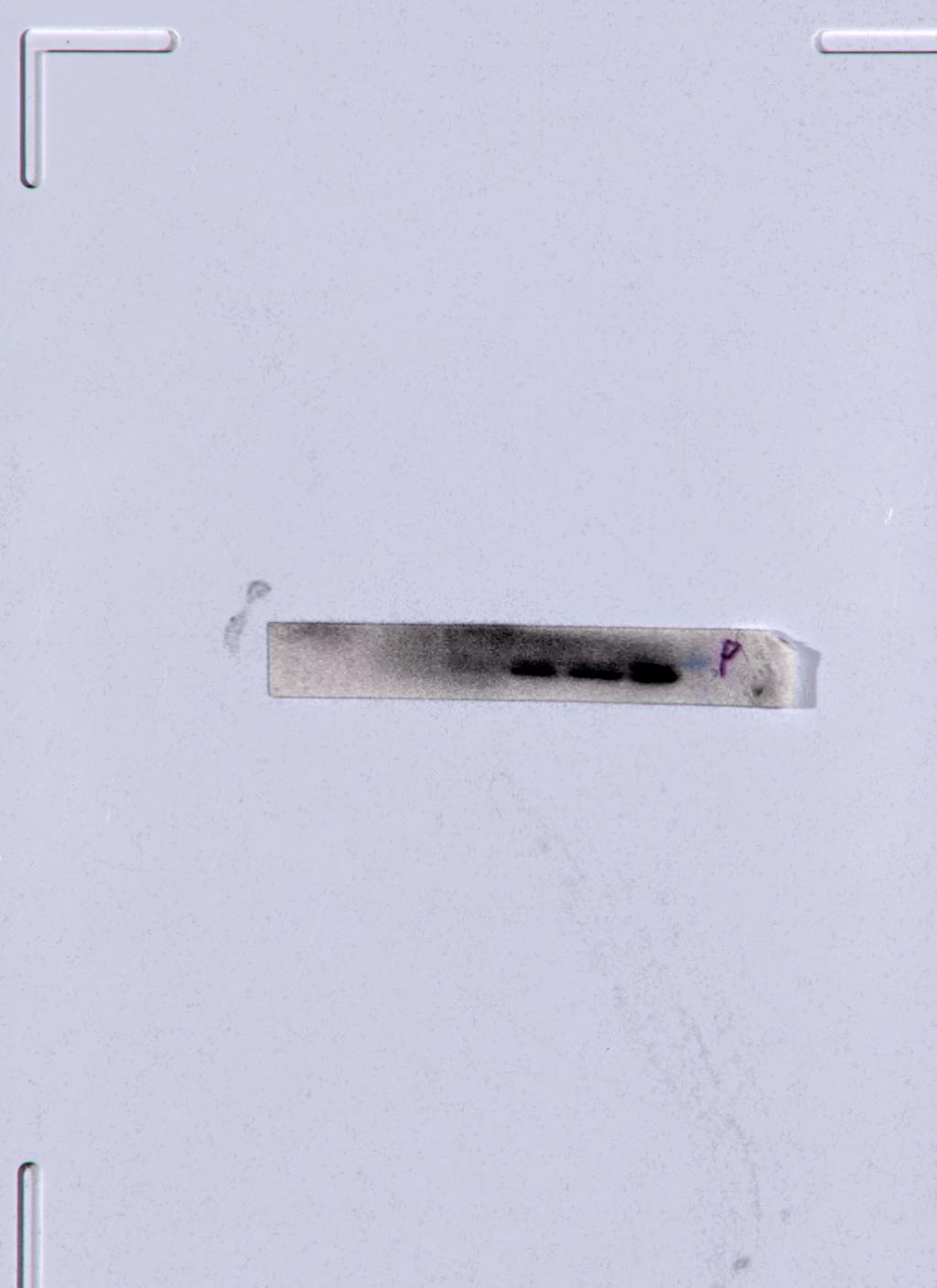

Supplement: Supplemental Information 2 [file peerj-13-19085-s002.zip › Chaetoglobosin A induces T-24 apoptosis in human bladder cancer/7.PI3K ERK pathway/p-p38/22.4.18 p-p38.3 2022.04.18_14.06.28_Ch/22.4.18 p-p38.3 2022.04.18_14.06.28_Ch+Marker.jpg]

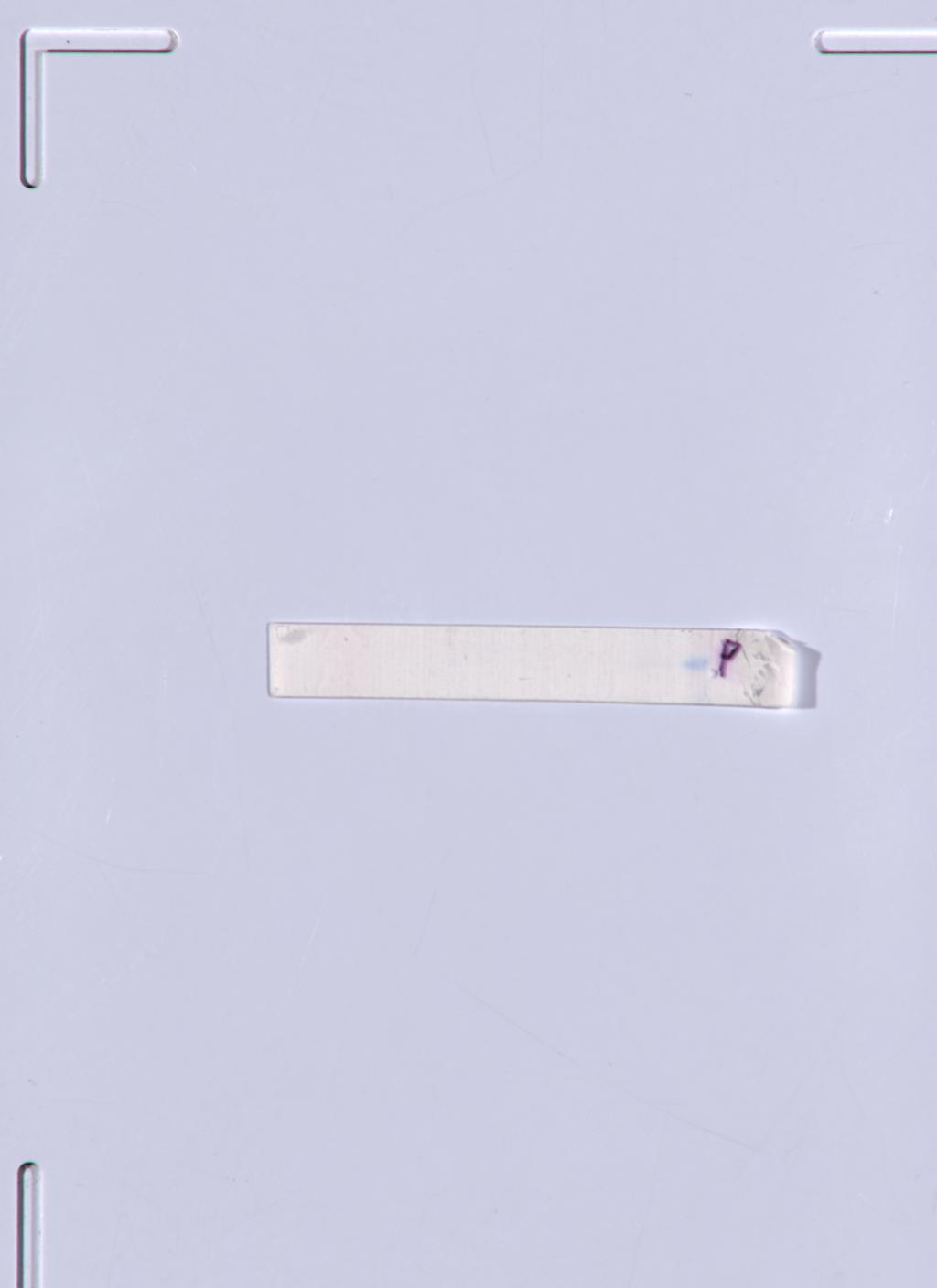

Supplement: Supplemental Information 2 [file peerj-13-19085-s002.zip › Chaetoglobosin A induces T-24 apoptosis in human bladder cancer/7.PI3K ERK pathway/p-p38/22.4.18 p-p38.3 2022.04.18_14.06.28_Ch/22.4.18 p-p38.3 2022.04.18_14.06.28_Ch-Marker.jpg]

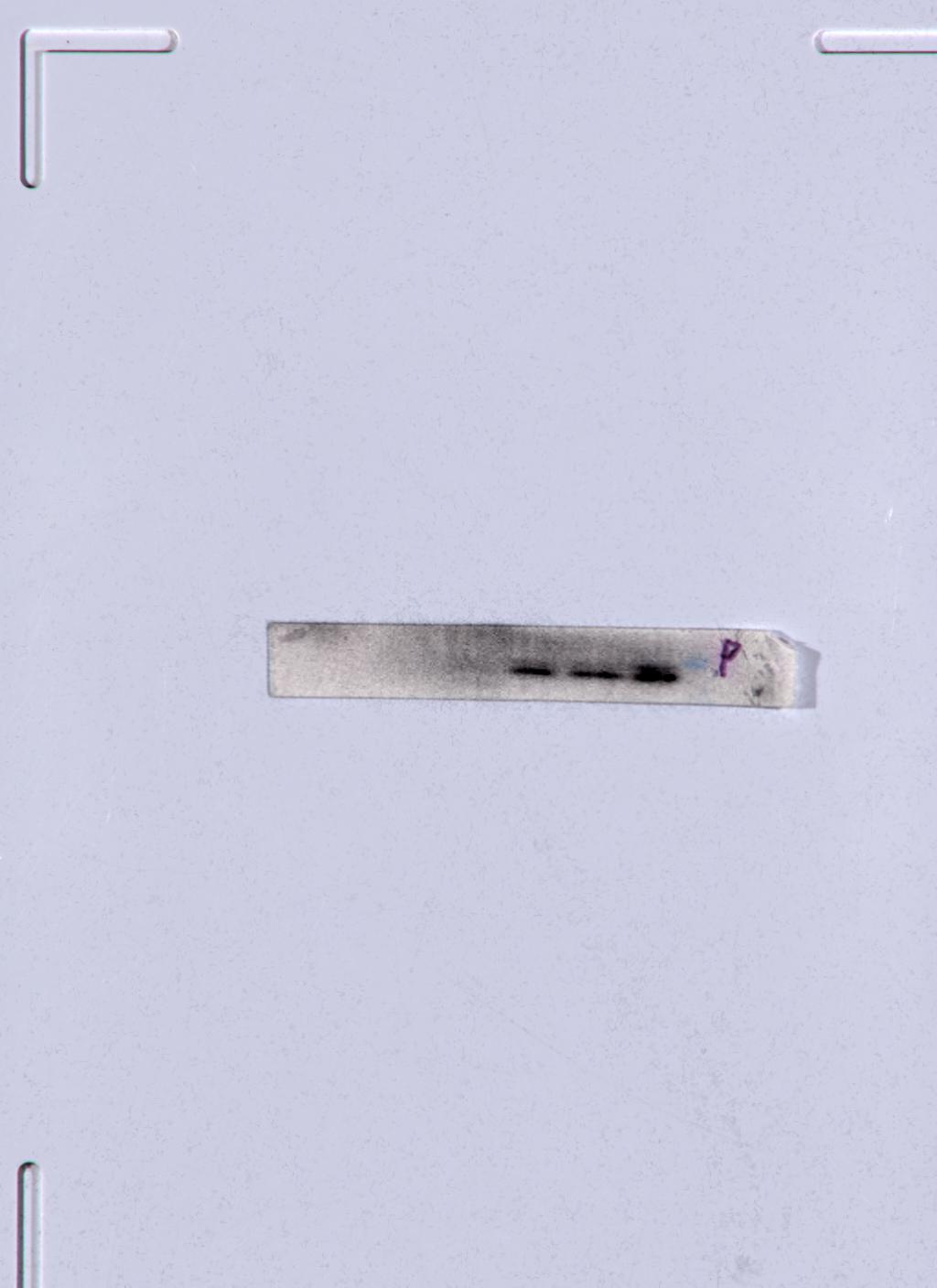

Supplement: Supplemental Information 2 [file peerj-13-19085-s002.zip › Chaetoglobosin A induces T-24 apoptosis in human bladder cancer/7.PI3K ERK pathway/p-p38/22.4.18 p-p38.4 2022.04.18_14.08.46_Ch/22.4.18 p-p38.4 2022.04.18_14.08.46_Ch+Marker.jpg]

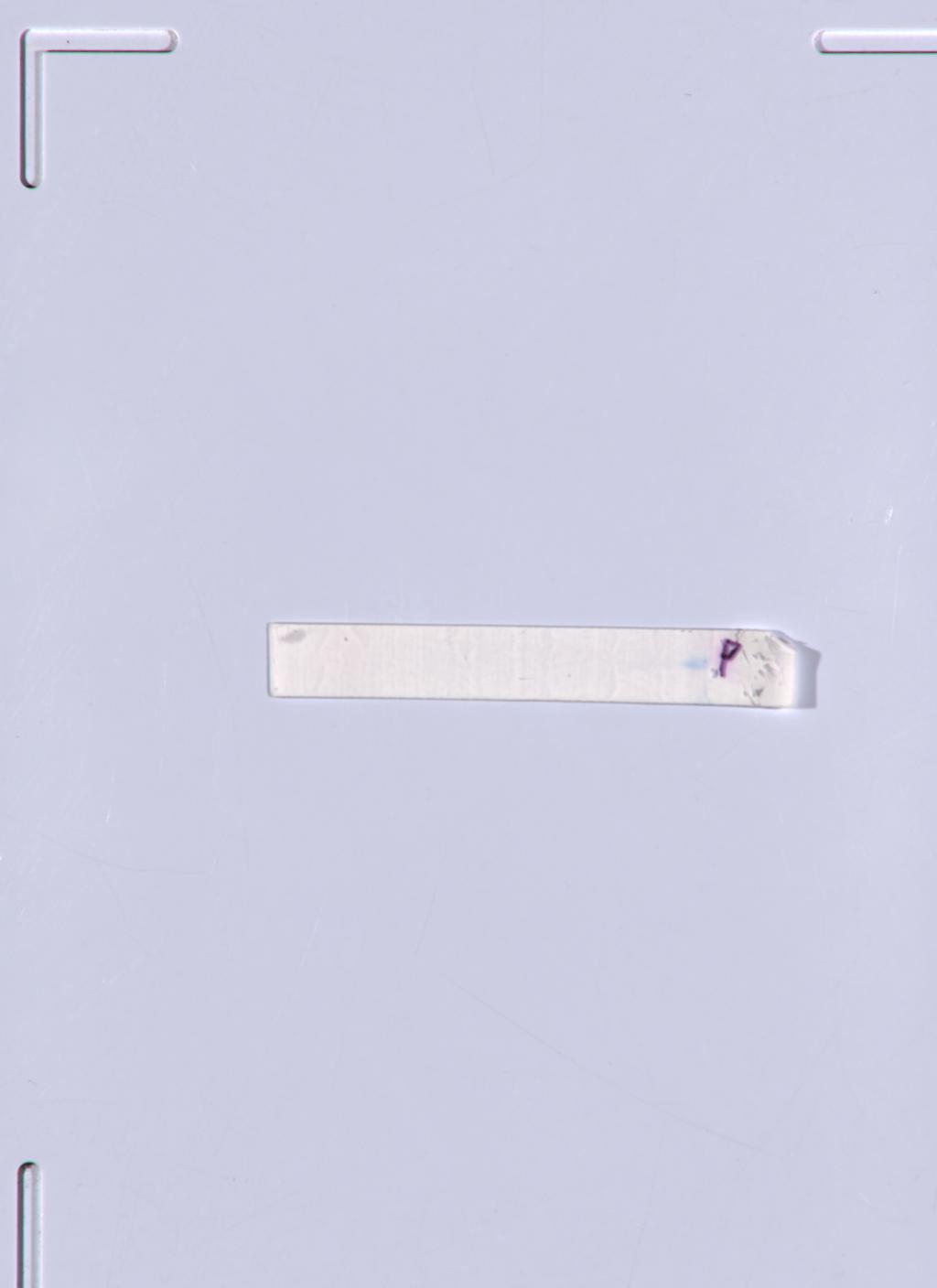

Supplement: Supplemental Information 2 [file peerj-13-19085-s002.zip › Chaetoglobosin A induces T-24 apoptosis in human bladder cancer/7.PI3K ERK pathway/p-p38/22.4.18 p-p38.4 2022.04.18_14.08.46_Ch/22.4.18 p-p38.4 2022.04.18_14.08.46_Ch-Marker.jpg]

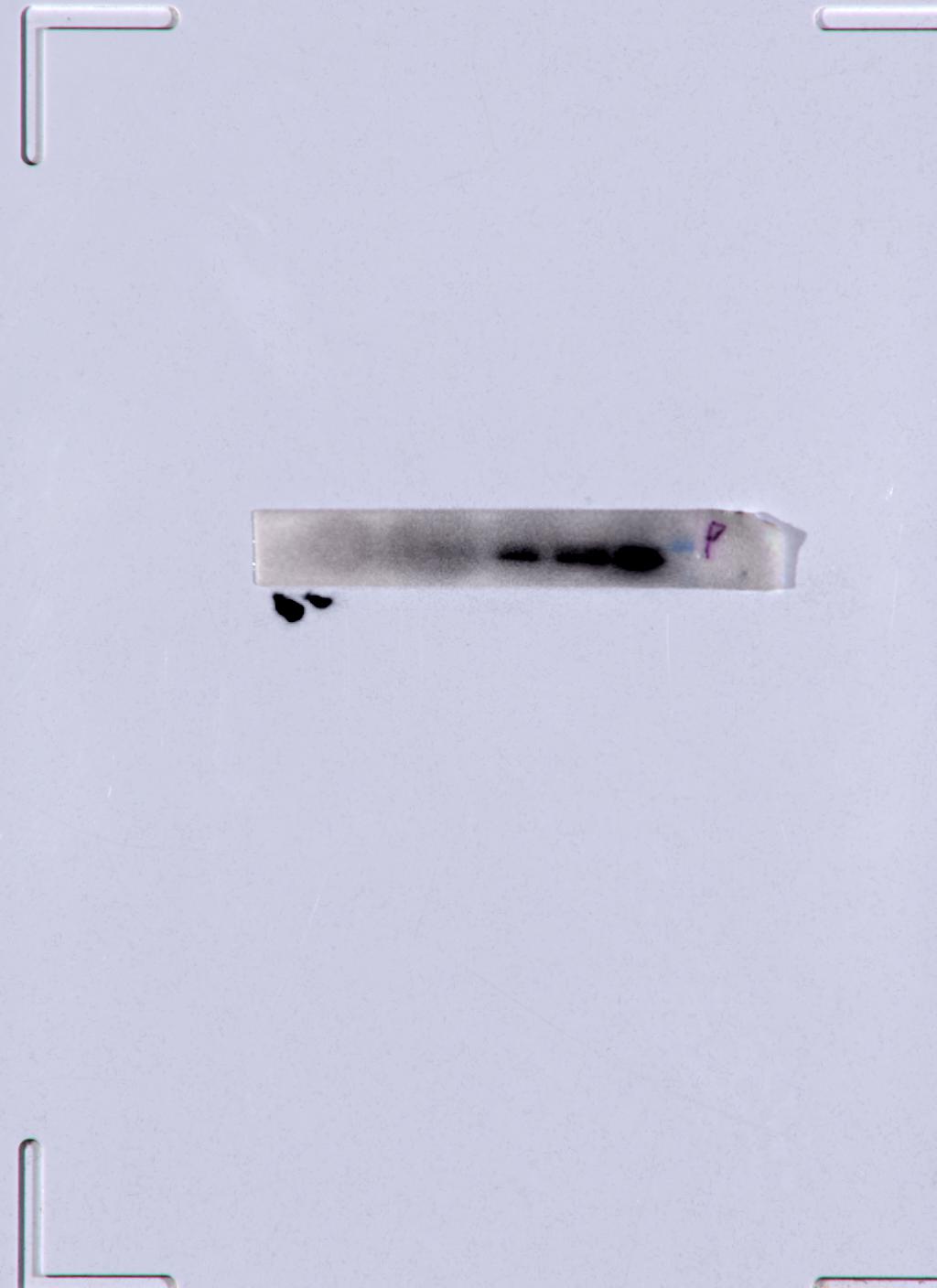

Supplement: Supplemental Information 2 [file peerj-13-19085-s002.zip › Chaetoglobosin A induces T-24 apoptosis in human bladder cancer/7.PI3K ERK pathway/p-p38/22.4.18 p-p38.5 2022.04.18_14.27.17_Ch/22.4.18 p-p38.5 2022.04.18_14.27.17_Ch+Marker.jpg]
