# Supplementary material for: Chaetoglobosin A induces apoptosis in T-24 human bladder cancer cells through oxidative stress and MAPK/PI3K-AKT-mTOR pathway
Source: PeerJ. 2025 Mar 31;13:e19085. doi: 10.7717/peerj.19085 (PMC11967413; doi:10.7717/peerj.19085)
Supplement: Supplemental Information 2 [file peerj-13-19085-s002.zip › Chaetoglobosin A induces T-24 apoptosis in human bladder cancer/3.apoptosis/2 times/20220516 diao/FITC-PI-ana-17.pdf]

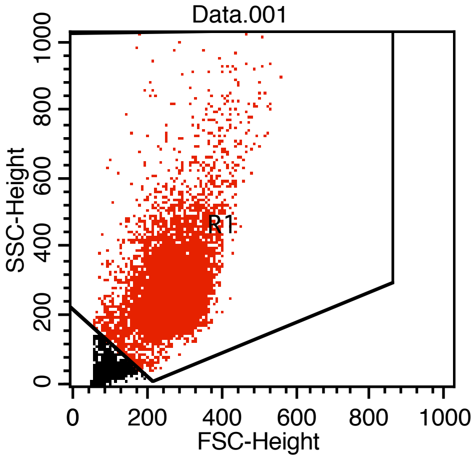

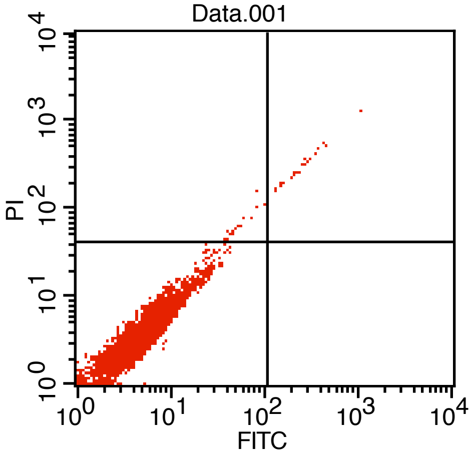

File: Data.001

Quad	Events	% Gated	% Total
UL	22	0.26	0.22
UR	26	0.31	0.26
LL	8292	99.42	82.92
LR	0	0.00	0.00

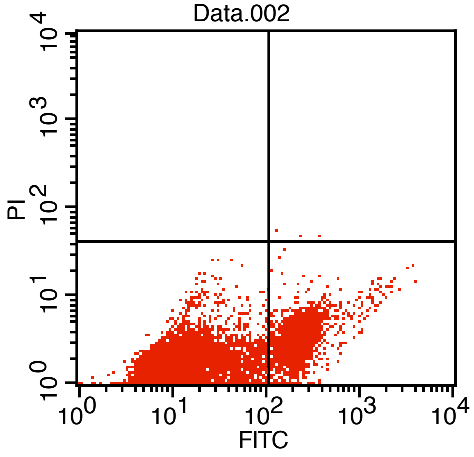

File: Data.002

Quad	Events	% Gated	% Total
UL	0	0.00	0.00
UR	3	0.03	0.03
LL	6963	77.99	69.63
LR	1962	21.98	19.62

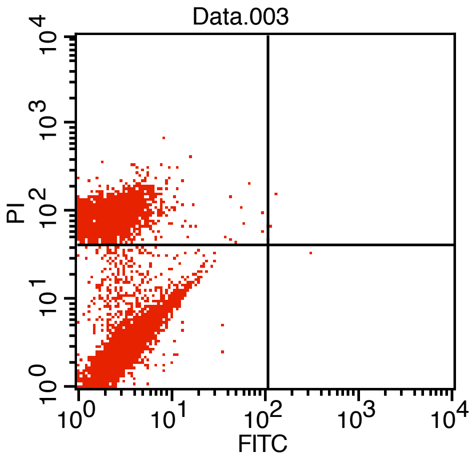

File: Data.003

Quad	Events	% Gated	% Total
UL	2736	30.75	27.36
UR	4	0.04	0.04
LL	6158	69.20	61.58
LR	1	0.01	0.01

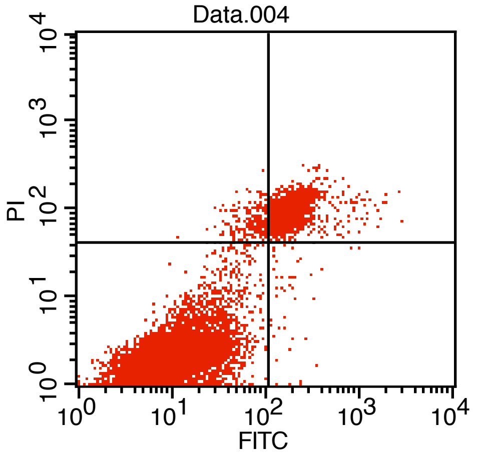

File: Data.004

Quad	Events	% Gated	% Total
UL	233	2.50	2.33
UR	1225	13.16	12.25
LL	7808	83.86	78.08
LR	45	0.48	0.45

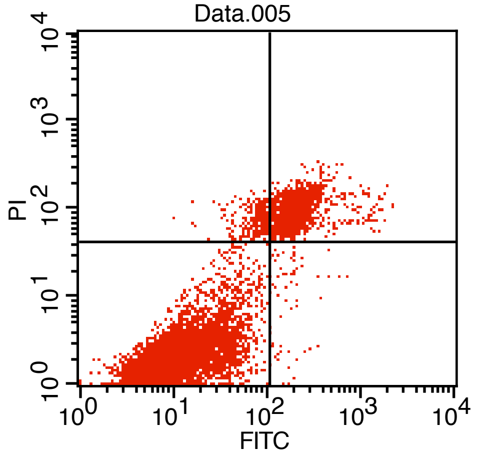

File: Data.005

Quad	Events	% Gated	% Total
UL	298	3.28	2.98
UR	1759	19.36	17.59
LL	6970	76.71	69.70
LR	59	0.65	0.59

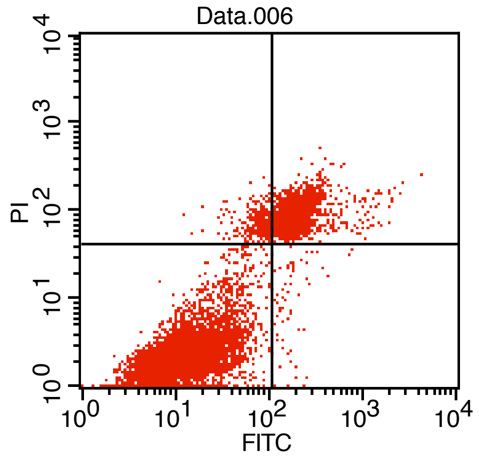

File: Data.006

Quad	Events	% Gated	% Total
UL	284	3.16	2.84
UR	1887	21.01	18.87
LL	6743	75.09	67.43
LR	66	0.73	0.66

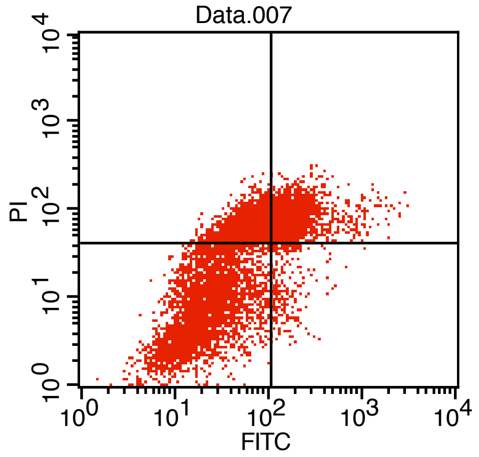

File: Data.007

Quad	Events	% Gated	% Total
UL	2393	25.55	23.93
UR	2689	28.71	26.89
LL	4011	42.83	40.11
LR	273	2.91	2.73

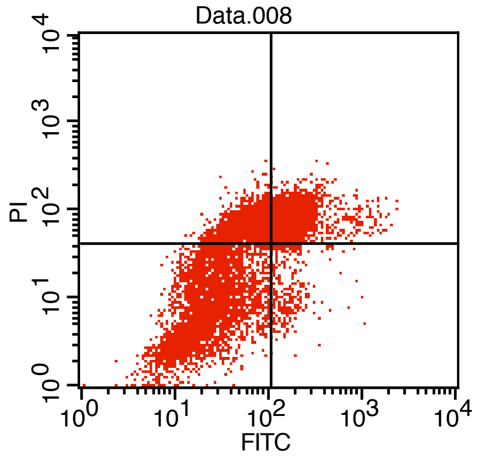

File: Data.008

Quad	Events	% Gated	% Total
UL	2387	25.72	23.87
UR	2849	30.70	28.49
LL	3709	39.97	37.09
LR	335	3.61	3.35

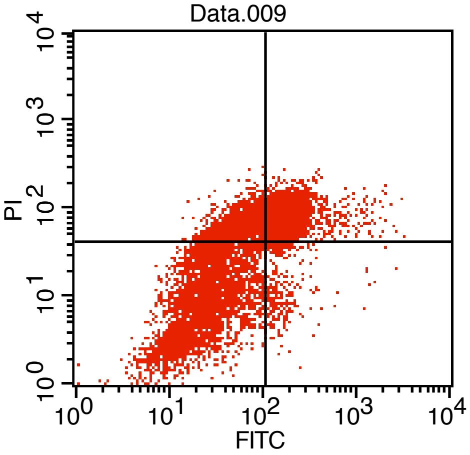

File: Data.009

Quad	Events	% Gated	% Total
UL	2299	24.95	22.99
UR	2882	31.27	28.82
LL	3688	40.02	36.88
LR	347	3.77	3.47

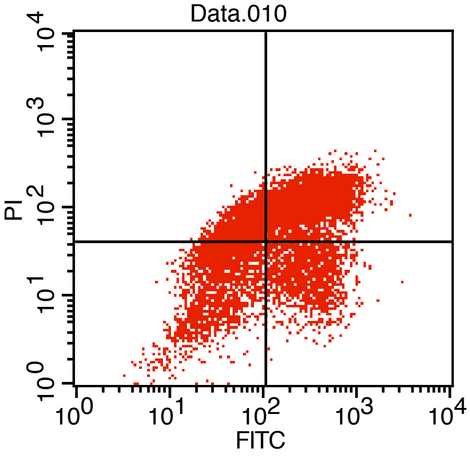

File: Data.010

Quad	Events	% Gated	% Total
UL	1801	18.98	18.01
UR	3977	41.91	39.77
LL	2210	23.29	22.10
LR	1502	15.83	15.02

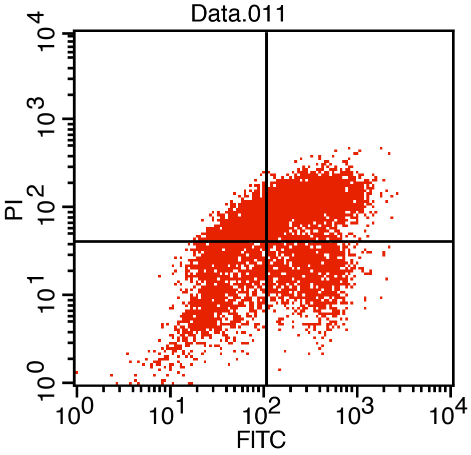

File: Data.011

Quad	Events	% Gated	% Total
UL	1811	19.07	18.11
UR	3950	41.59	39.50
LL	2152	22.66	21.52
LR	1584	16.68	15.84

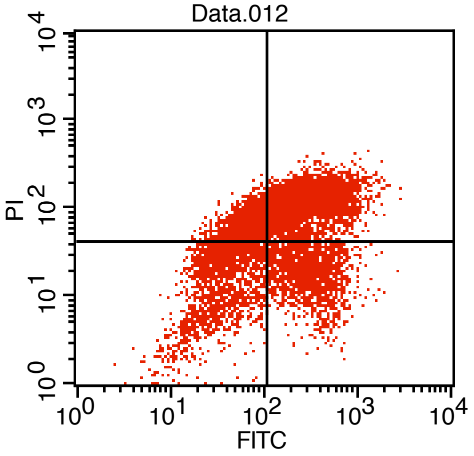

File: Data.012

Quad	Events	% Gated	% Tota
UL	1806	19.09	18.06
UR	3998	42.25	39.98
LL	1985	20.98	19.85
LR	1673	17.68	16.73

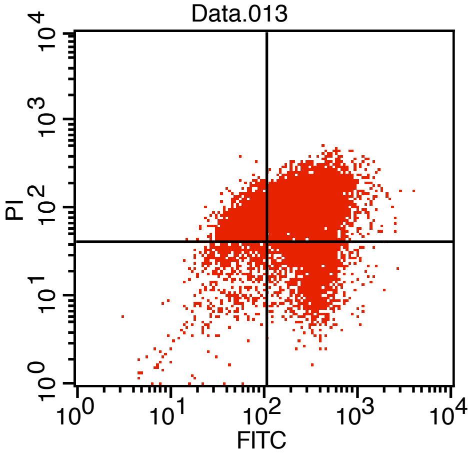

File: Data.013

Quad	Events	% Gated	% Total
UL	1581	16.68	15.81
UR	5522	58.25	55.22
LL	657	6.93	6.57
LR	1720	18.14	17.20

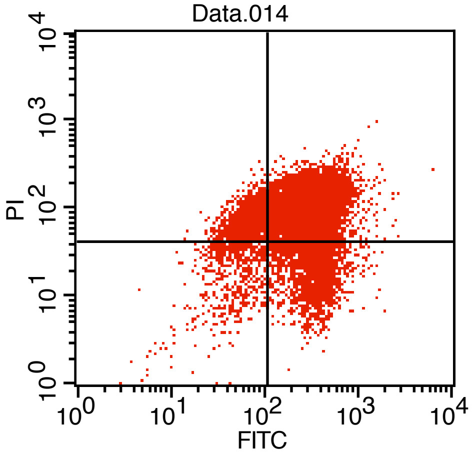

File: Data.014

Quad	Events	% Gated	% Total
UL	1491	15.88	14.91
UR	5303	56.49	53.03
LL	640	6.82	6.40
LR	1954	20.81	19.54

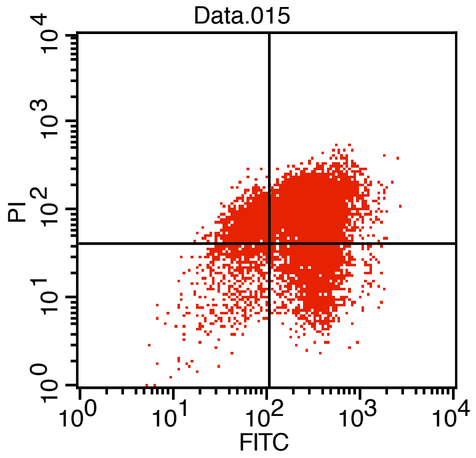

File: Data.015

Quad	Events	% Gated	% Total
UL	1467	15.85	14.67
UR	5237	56.59	52.37
LL	625	6.75	6.25
LR	1926	20.81	19.26

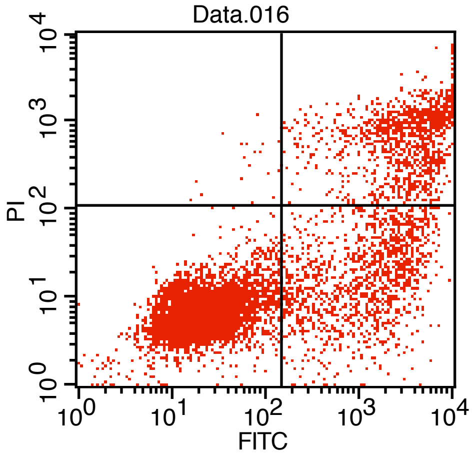

File: Data.016

Quad	Events	% Gated	% Total
UL	18	0.20	0.18
UR	1242	13.98	12.42
LL	6347	71.45	63.47
LR	1276	14.36	12.76

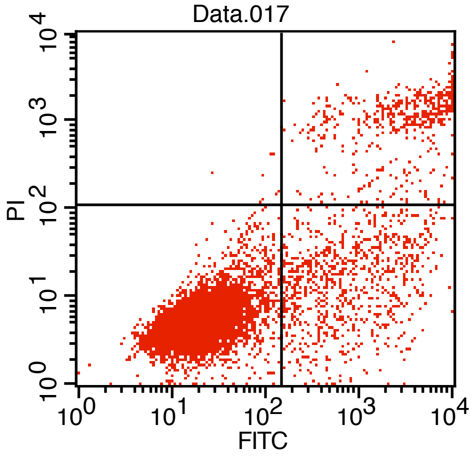

File: Data.017

Quad	Events	% Gated	% Total
UL	9	0.09	0.09
UR	487	5.12	4.87
LL	8171	85.88	81.71
LR	847	8.90	8.47
