# Supplementary material for: Chaetoglobosin A induces apoptosis in T-24 human bladder cancer cells through oxidative stress and MAPK/PI3K-AKT-mTOR pathway
Source: PeerJ. 2025 Mar 31;13:e19085. doi: 10.7717/peerj.19085 (PMC11967413; doi:10.7717/peerj.19085)
Supplement: Supplemental Information 2 [file peerj-13-19085-s002.zip › Chaetoglobosin A induces T-24 apoptosis in human bladder cancer/3.apoptosis/2 times/20221107/FITC-PI-ana-10.pdf]

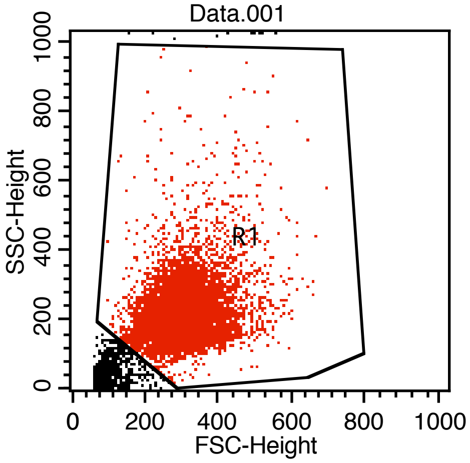

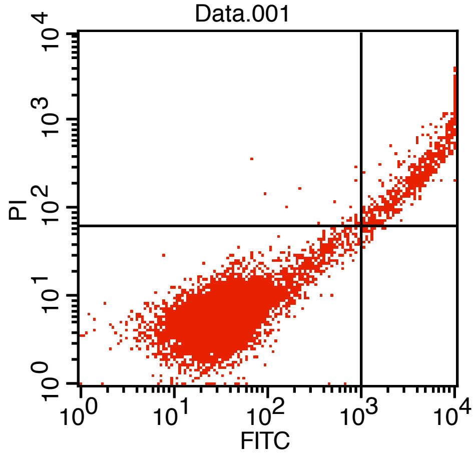

File: Data.001

Quad	Events	% Gated	% Total
UL	23	0.25	0.23
UR	668	7.22	6.68
LL	8534	92.23	85.34
LR	28	0.30	0.28

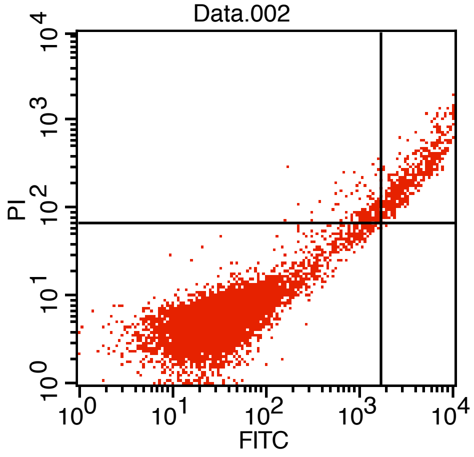

File: Data.002

Quad	Events	% Gated	% Total
UL	156	1.69	1.56
UR	515	5.59	5.15
LL	8536	92.69	85.36
LR	2	0.02	0.02

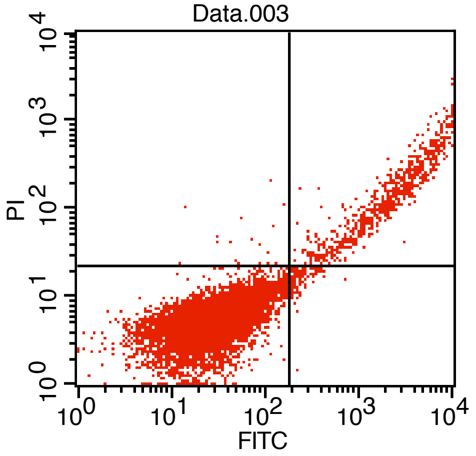

File: Data.003

Quad	Events	% Gated	% Total
UL	21	0.22	0.21
UR	704	7.39	7.04
LL	8669	91.00	86.69
LR	132	1.39	1.32

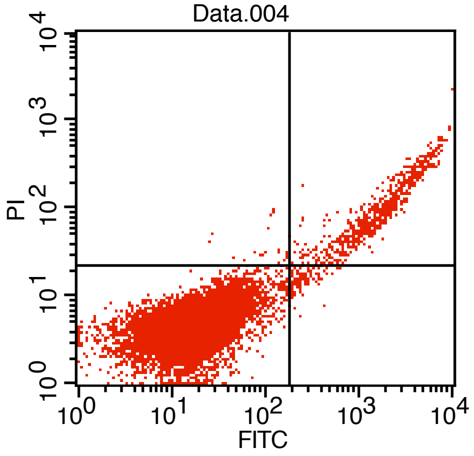

File: Data.004

Quad	Events	% Gated	% Total
UL	21	0.23	0.21
UR	666	7.21	6.66
LL	8450	91.45	84.50
LR	103	1.11	1.03

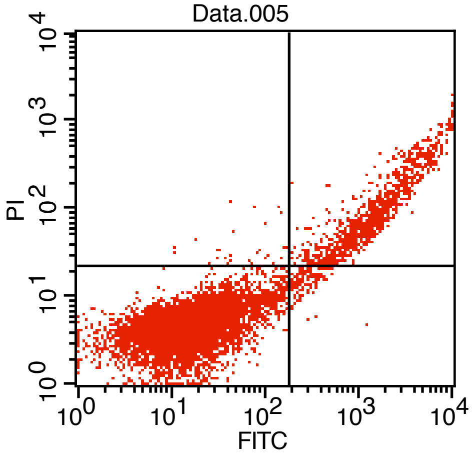

File: Data.005

Quad	Events	% Gated	% Total
UL	30	0.36	0.30
UR	1249	15.00	12.49
LL	6906	82.93	69.06
LR	143	1.72	1.43

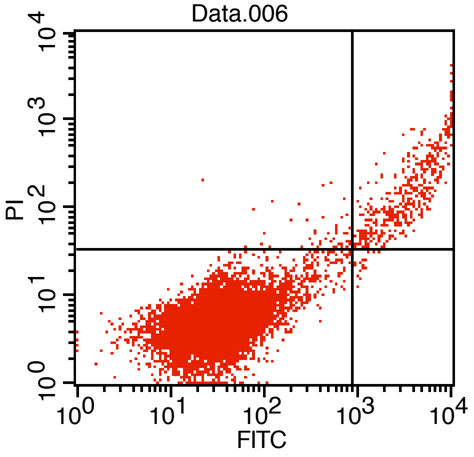

File: Data.006

Quad	Events	% Gated	% Total
UL	56	0.58	0.56
UR	455	4.72	4.55
LL	9114	94.50	91.14
LR	19	0.20	0.19

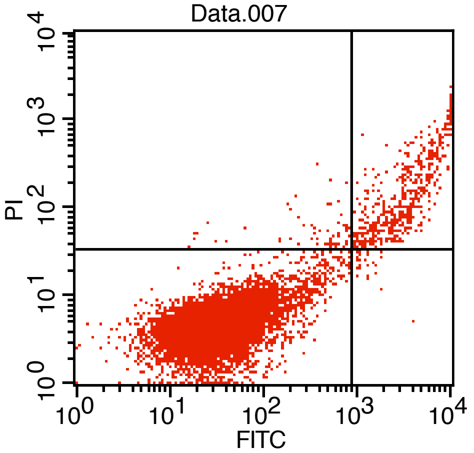

File: Data.007

Quad	Events	% Gated	% Total
UL	74	0.78	0.74
UR	513	5.38	5.13
LL	8909	93.51	89.09
LR	31	0.33	0.31

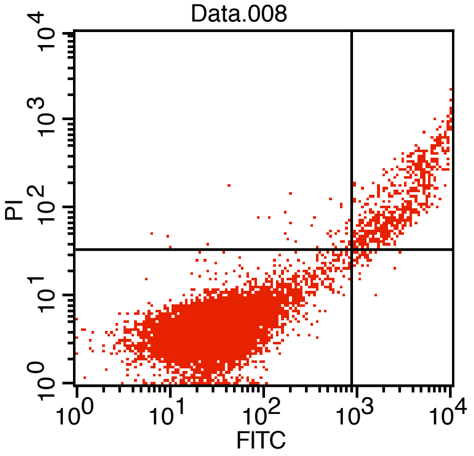

File: Data.008

Quad	Events	% Gated	% Total
UL	46	0.49	0.46
UR	616	6.53	6.16
LL	8742	92.63	87.42
LR	34	0.36	0.34

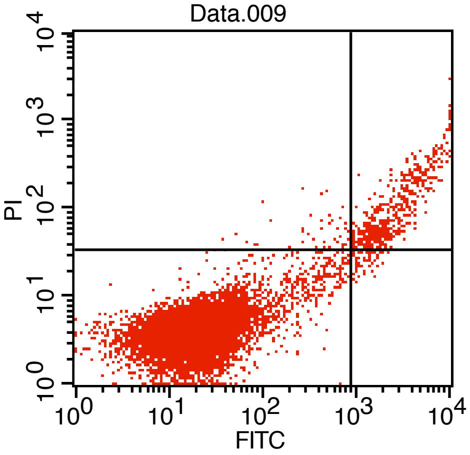

File: Data.009

Quad	Events	% Gated	% Total
UL	54	0.59	0.54
UR	522	5.73	5.22
LL	8473	92.98	84.73
LR	64	0.70	0.64

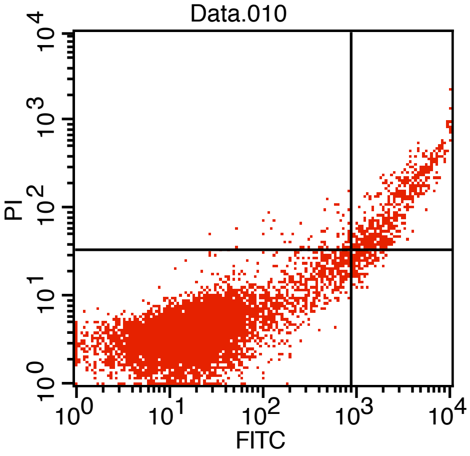

File: Data.010

Quad	Events	% Gated	% Total
UL	66	0.74	0.66
UR	574	6.45	5.74
LL	8107	91.05	81.07
LR	157	1.76	1.57
