# Supplementary material for: Chaetoglobosin A induces apoptosis in T-24 human bladder cancer cells through oxidative stress and MAPK/PI3K-AKT-mTOR pathway
Source: PeerJ. 2025 Mar 31;13:e19085. doi: 10.7717/peerj.19085 (PMC11967413; doi:10.7717/peerj.19085)
Supplement: Supplemental Information 2 [file peerj-13-19085-s002.zip › Chaetoglobosin A induces T-24 apoptosis in human bladder cancer/5.cells cycle/11.28.pdf]

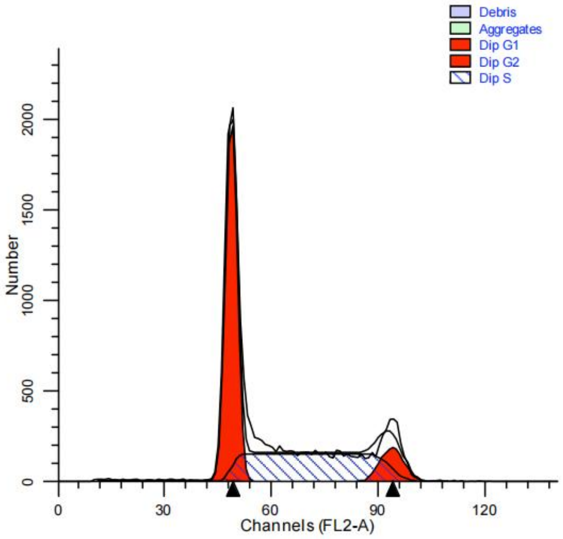

Diploid: 100.00 %
Dip G1: 49.26 % at 48.68
Dip G2: 9.23 % at 93.76
Dip S: 41.51 % G2/G1: 1.93
%CV: 3.43

Total S-Phase: 41.51 %
Total B.A.D.: 0.92 %

Apoptosis: % Mean:

Debris: 2.66 %
Aggregates: 0.28 %
Modeled events: 17844
All cycle events: 17319
Cycle events per channel: 376
RCS: 4.268

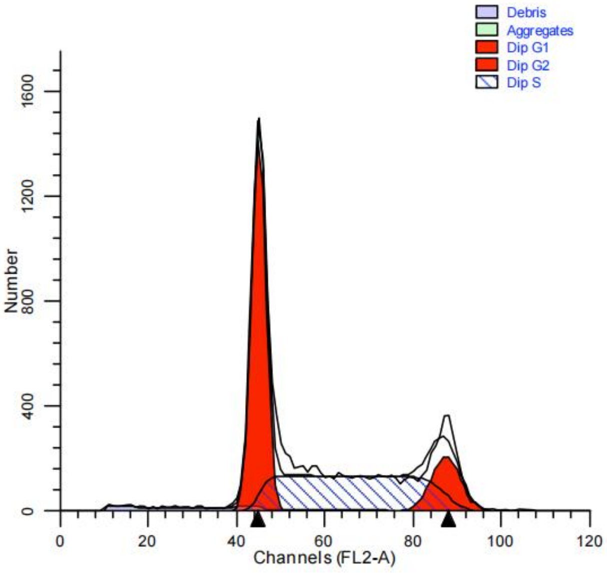

Diploid: 100.00 %
Dip G1: 45.01 % at 45.12
Dip G2: 13.16 % at 87.49
Dip S: 41.83 % G2/G1: 1.94
%CV: 3.70

Total S-Phase: 41.83 %
Total B.A.D.: 2.06 %

Apoptosis: % Mean:

Debris: 5.46 %
Aggregates: 0.17 %
Modeled events: 14190
All cycle events: 13391
Cycle events per channel: 309
RCS: 4.029

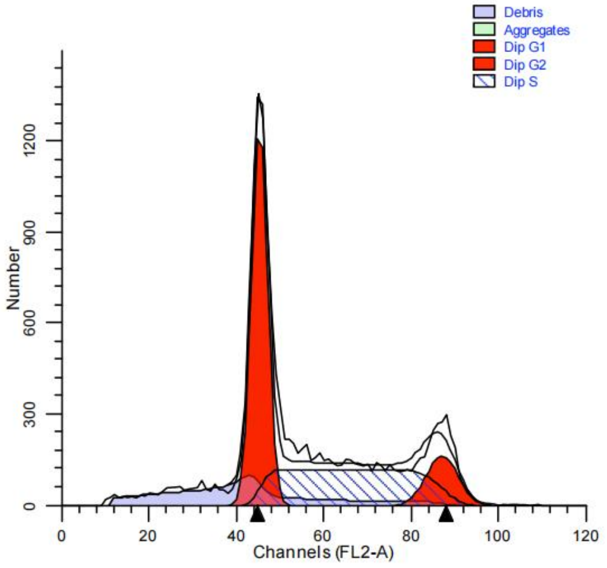

Diploid: 100.00 %
Dip G1: 47.06 % at 45.41
Dip G2: 12.42 % at 87.13
Dip S: 40.52 % G2/G1: 1.92
%CV: 4.11

Total S-Phase: 40.52 %
Total B.A.D.: 9.34 %

Apoptosis: % Mean:

Debris: 18.43 %
Aggregates: 0.25 %
Modeled events: 15350
All cycle events: 12483
Cycle events per channel: 292
RCS: 3.657

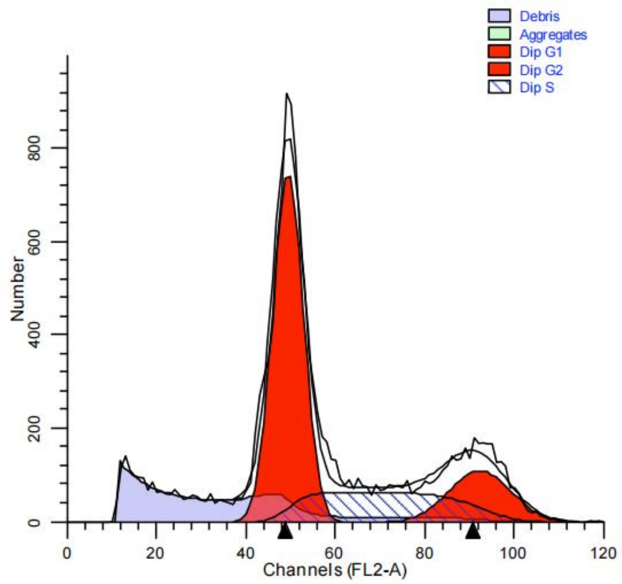

Diploid: 100.00 %
Dip G1: 58.19 % at 49.55
Dip G2: 17.39 % at 92.43
Dip S: 24.43 % G2/G1: 1.87
%CV: 7.04

Total S-Phase: 24.43 %
Total B.A.D.: 7.86 %

Debris: 21.66 %
Aggregates: 0.15 %
Modeled events: 14389
All cycle events: 11251
Cycle events per channel: 256
RCS: 2.596
