# Supplementary figures and images for: Chaetoglobosin A induces apoptosis in T-24 human bladder cancer cells through oxidative stress and MAPK/PI3K-AKT-mTOR pathway (part 2 of 2)
Source: PeerJ. 2025 Mar 31;13:e19085. doi: 10.7717/peerj.19085 (PMC11967413; doi:10.7717/peerj.19085)

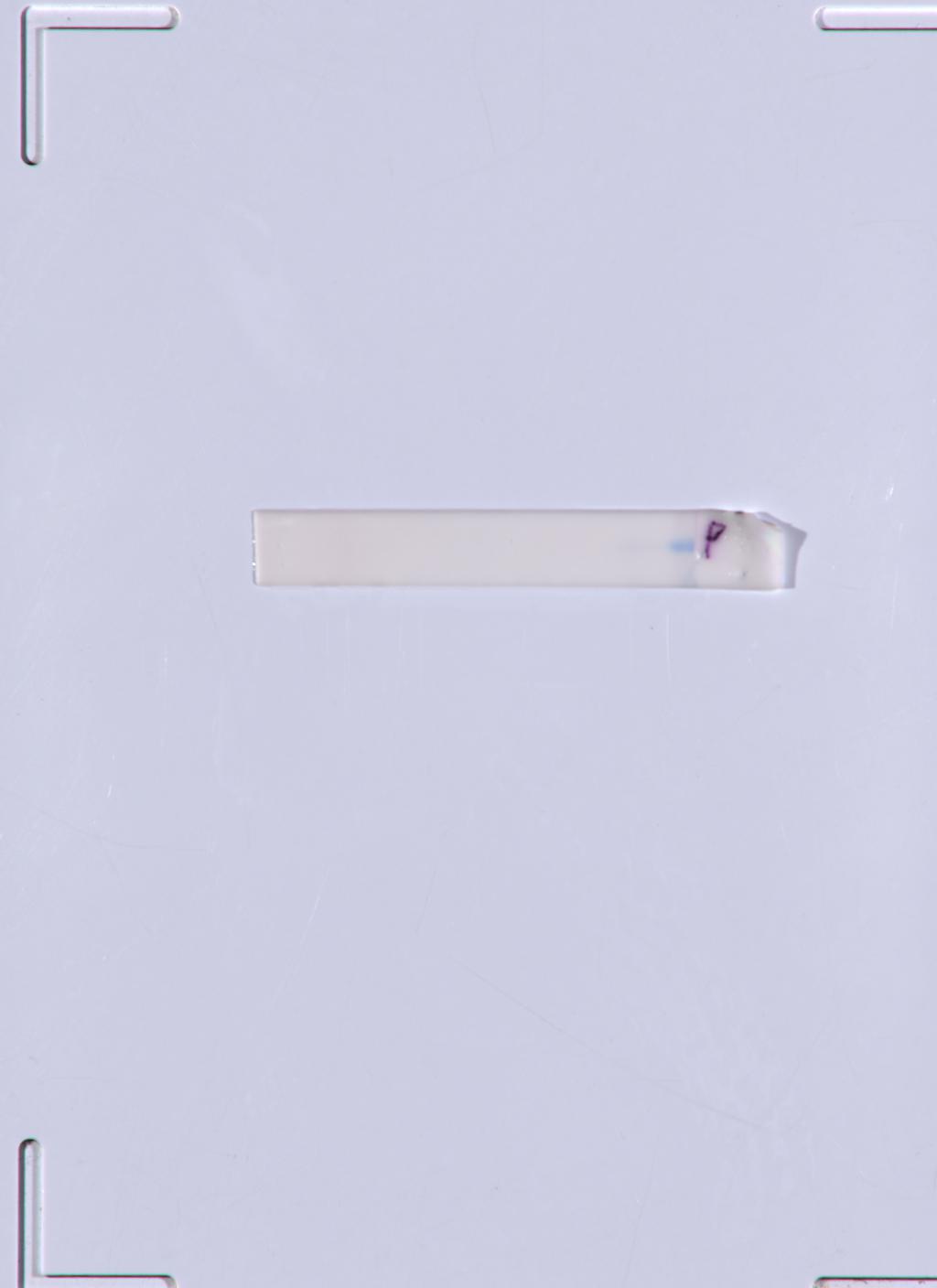

Supplement: Supplemental Information 2 [file peerj-13-19085-s002.zip › Chaetoglobosin A induces T-24 apoptosis in human bladder cancer/7.PI3K ERK pathway/p-p38/22.4.18 p-p38.5 2022.04.18_14.27.17_Ch/22.4.18 p-p38.5 2022.04.18_14.27.17_Ch-Marker.jpg]

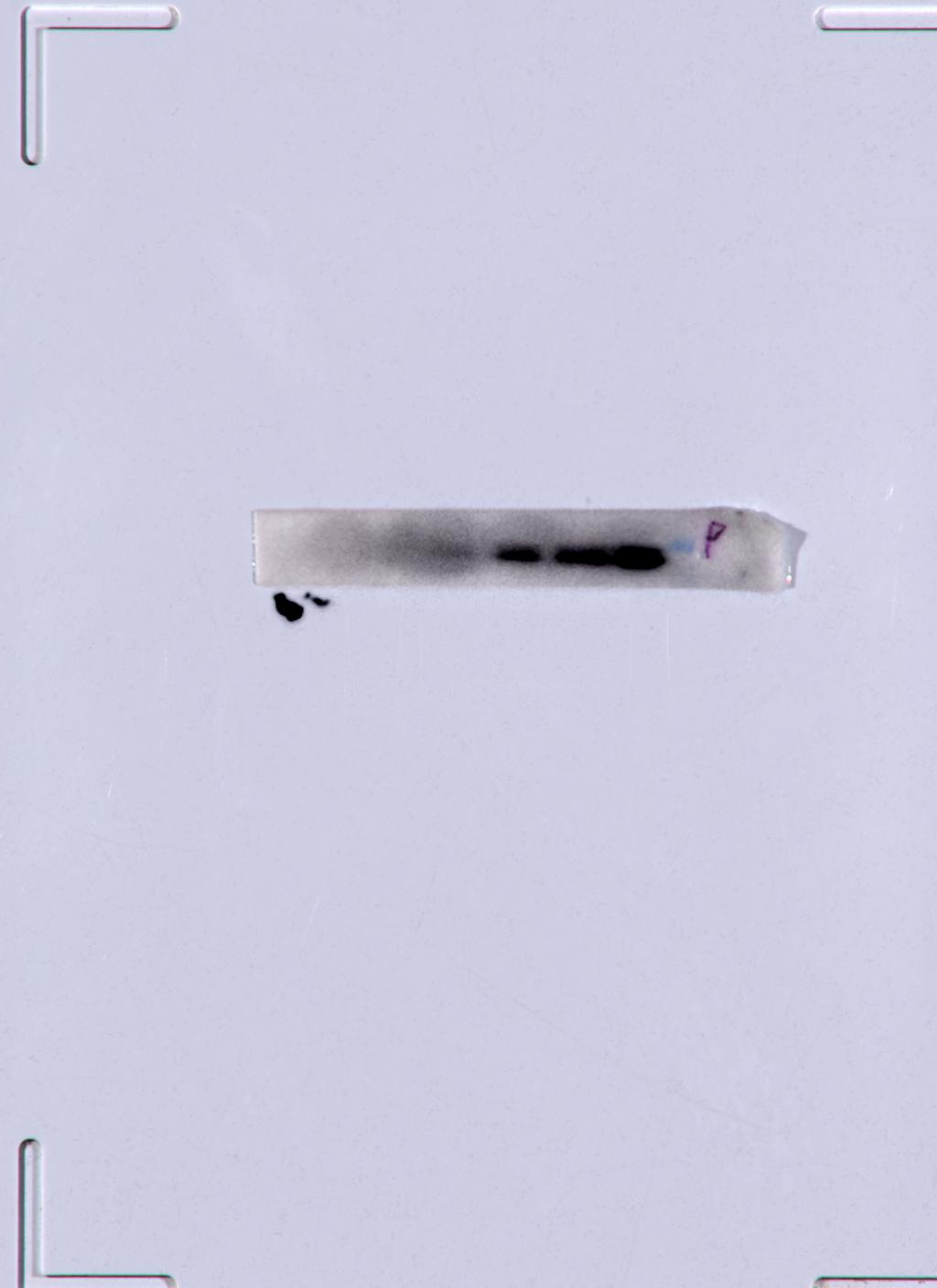

Supplement: Supplemental Information 2 [file peerj-13-19085-s002.zip › Chaetoglobosin A induces T-24 apoptosis in human bladder cancer/7.PI3K ERK pathway/p-p38/22.4.18 p-p38.6 2022.04.18_14.29.31_Ch/22.4.18 p-p38.6 2022.04.18_14.29.31_Ch+Marker.jpg]

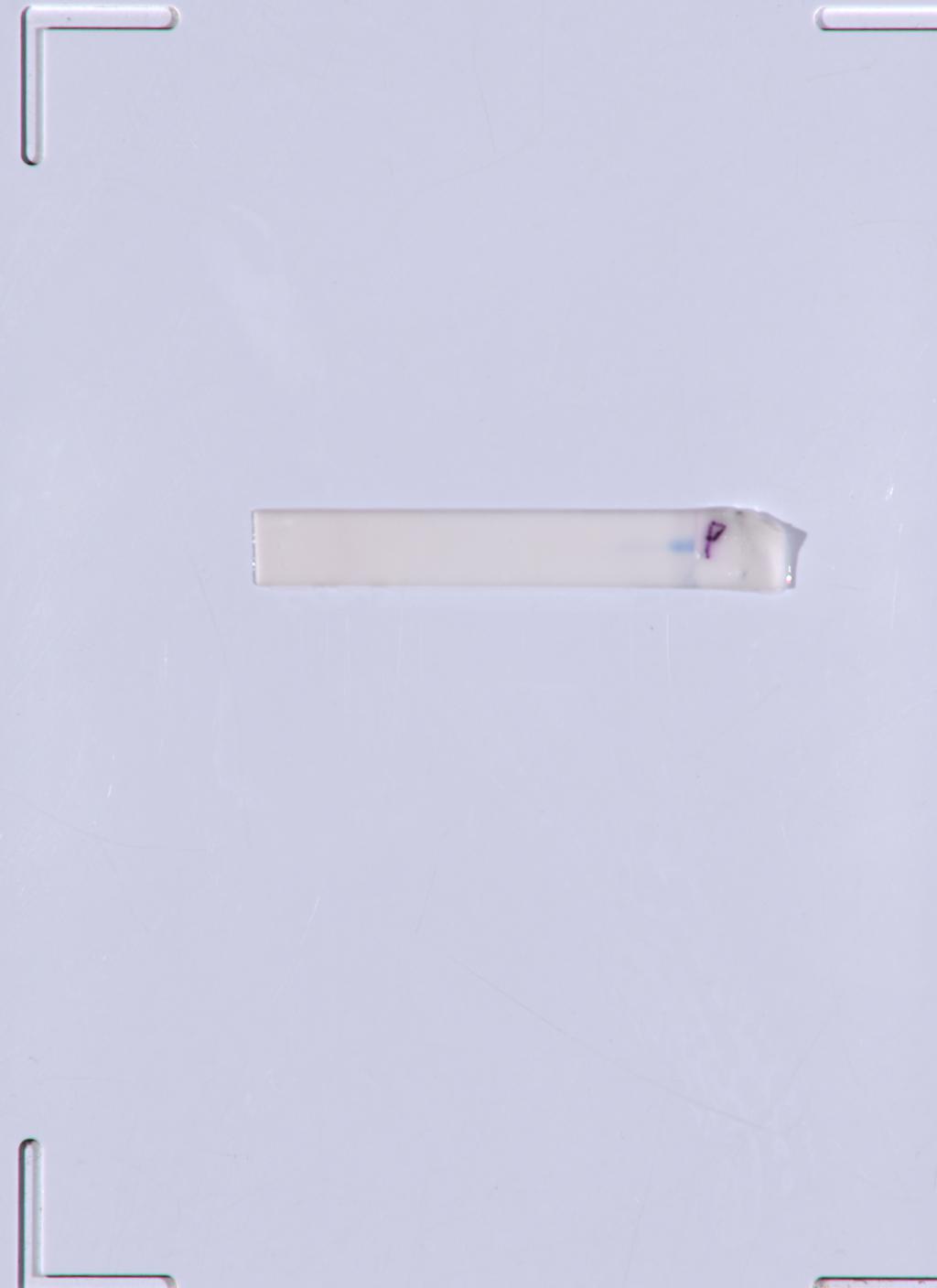

Supplement: Supplemental Information 2 [file peerj-13-19085-s002.zip › Chaetoglobosin A induces T-24 apoptosis in human bladder cancer/7.PI3K ERK pathway/p-p38/22.4.18 p-p38.6 2022.04.18_14.29.31_Ch/22.4.18 p-p38.6 2022.04.18_14.29.31_Ch-Marker.jpg]

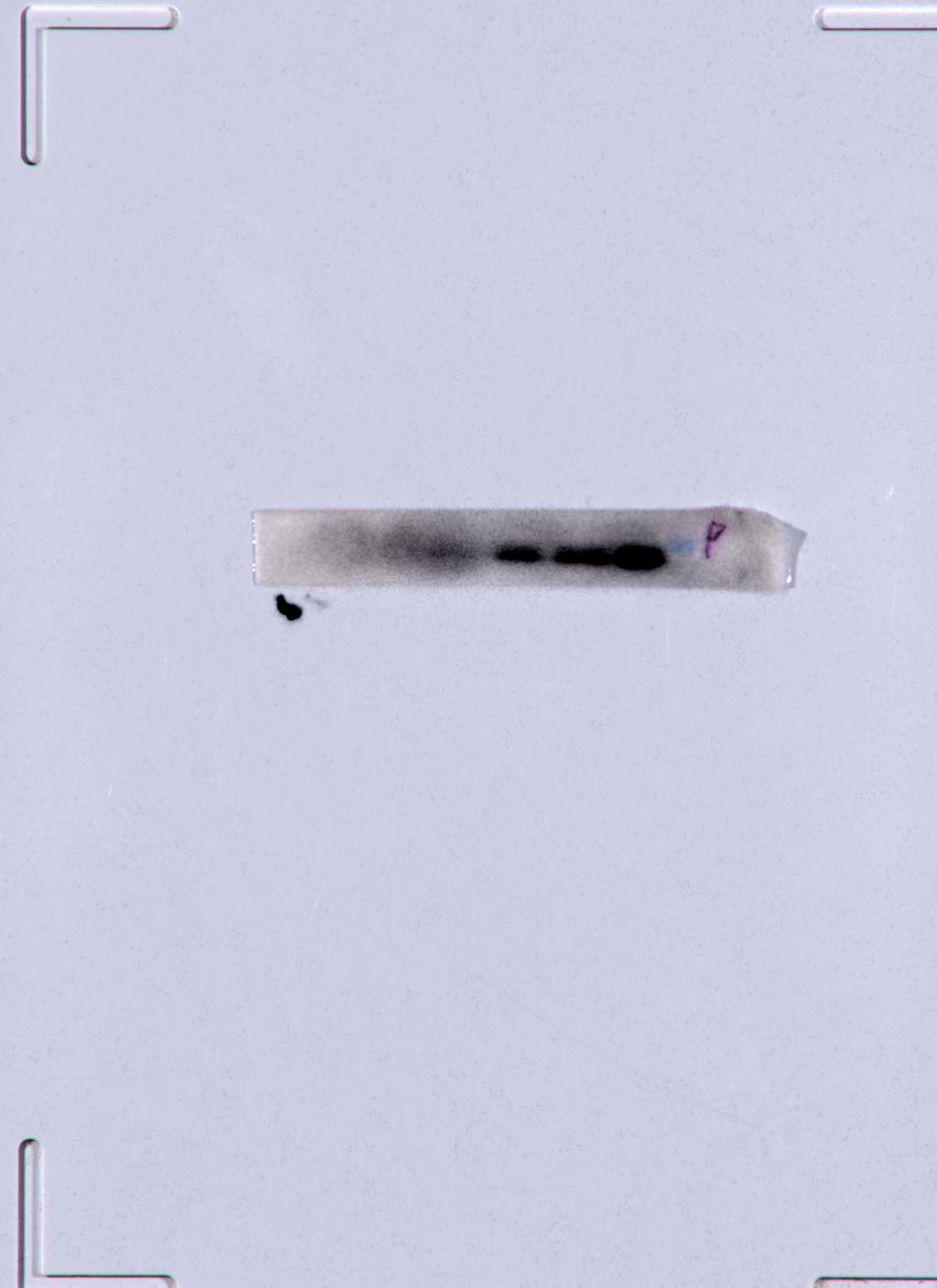

Supplement: Supplemental Information 2 [file peerj-13-19085-s002.zip › Chaetoglobosin A induces T-24 apoptosis in human bladder cancer/7.PI3K ERK pathway/p-p38/22.4.18 p-p38.7 2022.04.18_14.31.55_Ch/22.4.18 p-p38.7 2022.04.18_14.31.55_Ch+Marker.jpg]

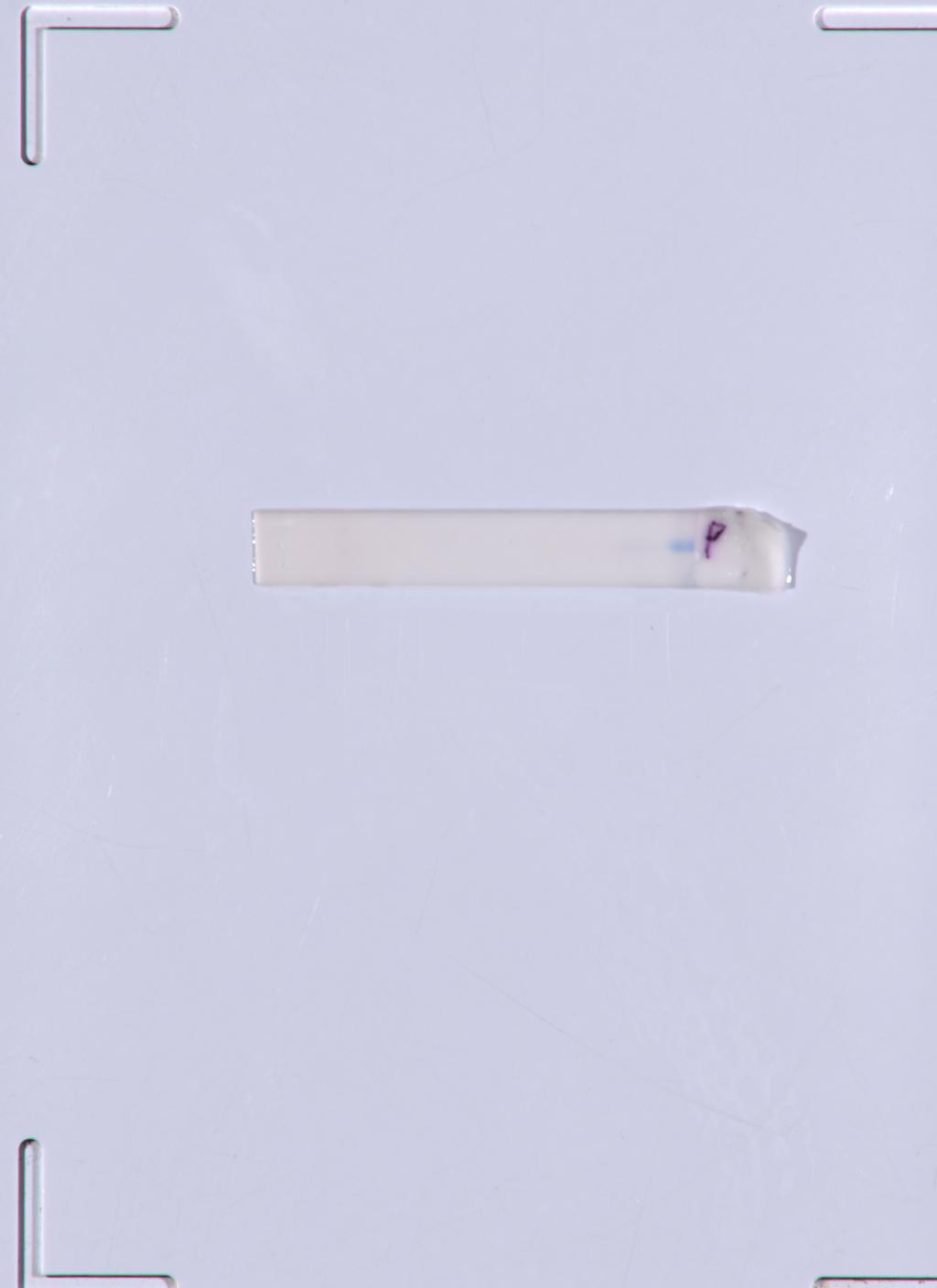

Supplement: Supplemental Information 2 [file peerj-13-19085-s002.zip › Chaetoglobosin A induces T-24 apoptosis in human bladder cancer/7.PI3K ERK pathway/p-p38/22.4.18 p-p38.7 2022.04.18_14.31.55_Ch/22.4.18 p-p38.7 2022.04.18_14.31.55_Ch-Marker.jpg]

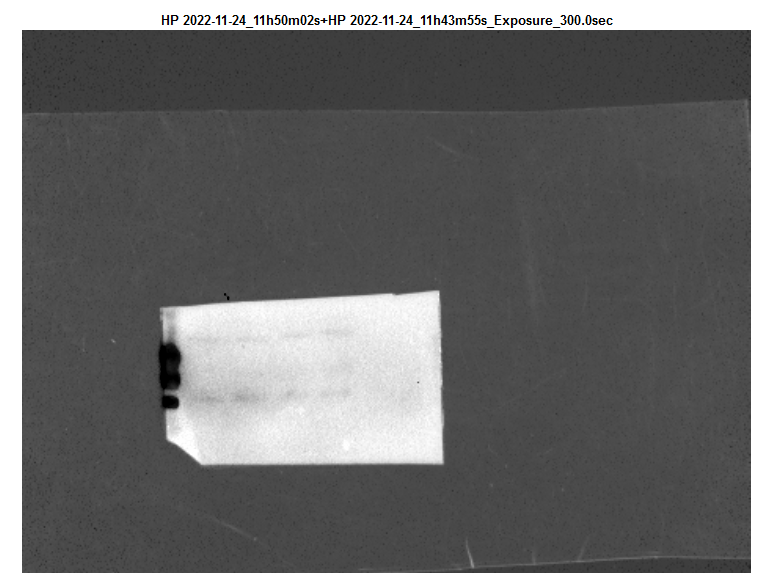

Supplement: Supplemental Information 2 [file peerj-13-19085-s002.zip › Chaetoglobosin A induces T-24 apoptosis in human bladder cancer/7.PI3K ERK pathway/p-PI3K/HP 2022-11-24_11h50m02s+HP 2022-11-24_11h43m55s_Exposure_300.0sec.tif]

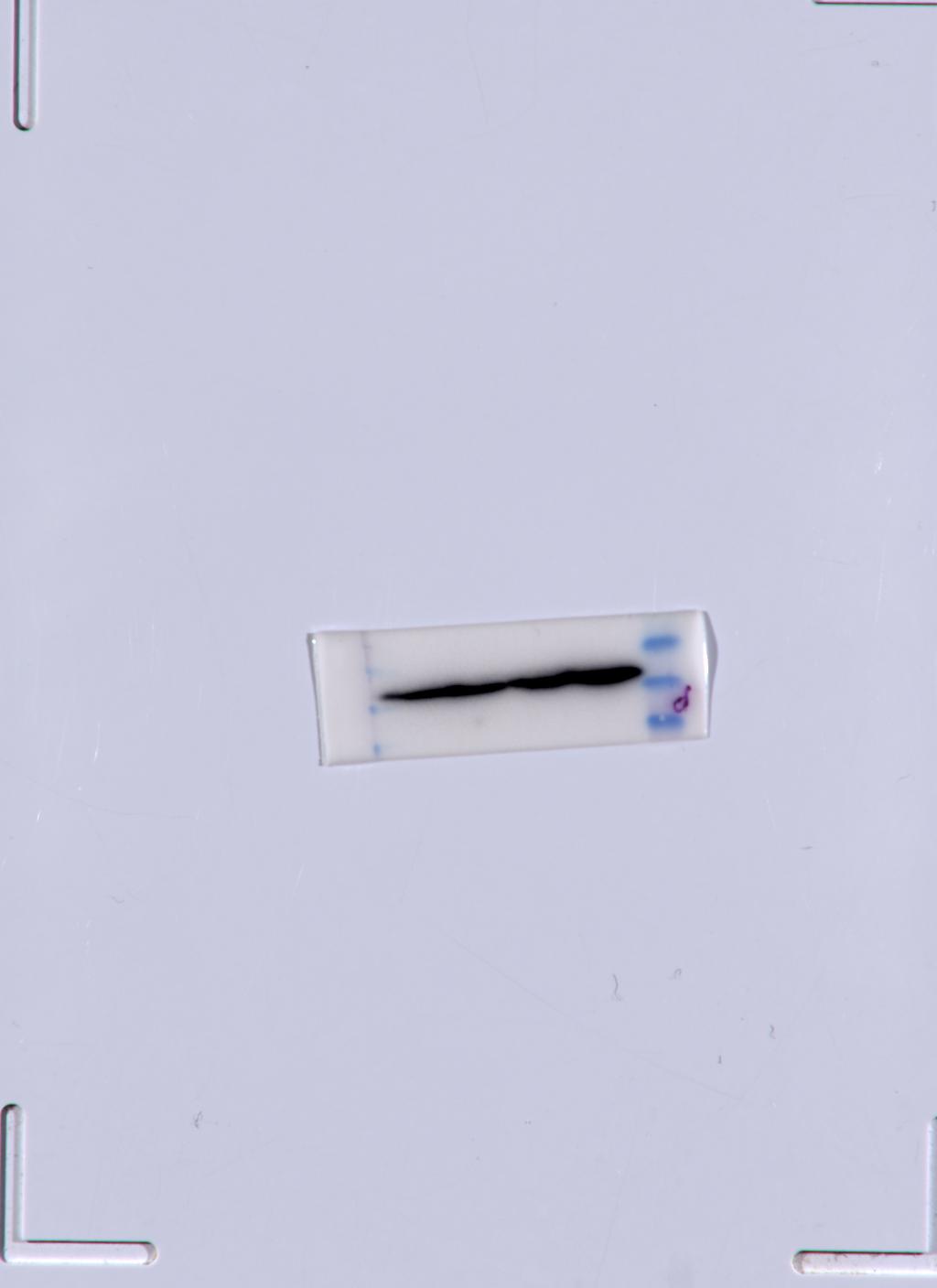

Supplement: Supplemental Information 2 [file peerj-13-19085-s002.zip › Chaetoglobosin A induces T-24 apoptosis in human bladder cancer/7.PI3K ERK pathway/p38/1.jpg]

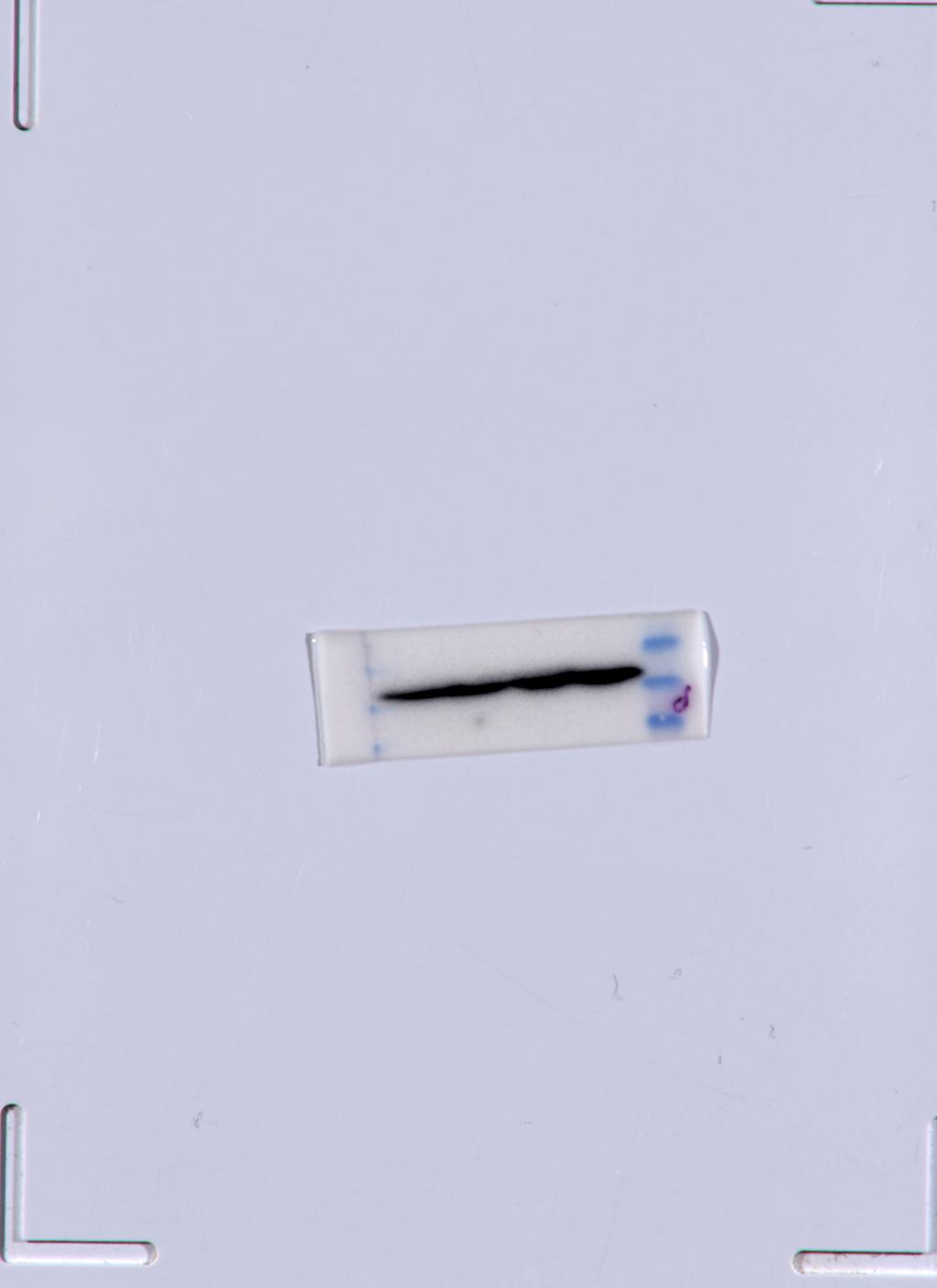

Supplement: Supplemental Information 2 [file peerj-13-19085-s002.zip › Chaetoglobosin A induces T-24 apoptosis in human bladder cancer/7.PI3K ERK pathway/p38/2.jpg]

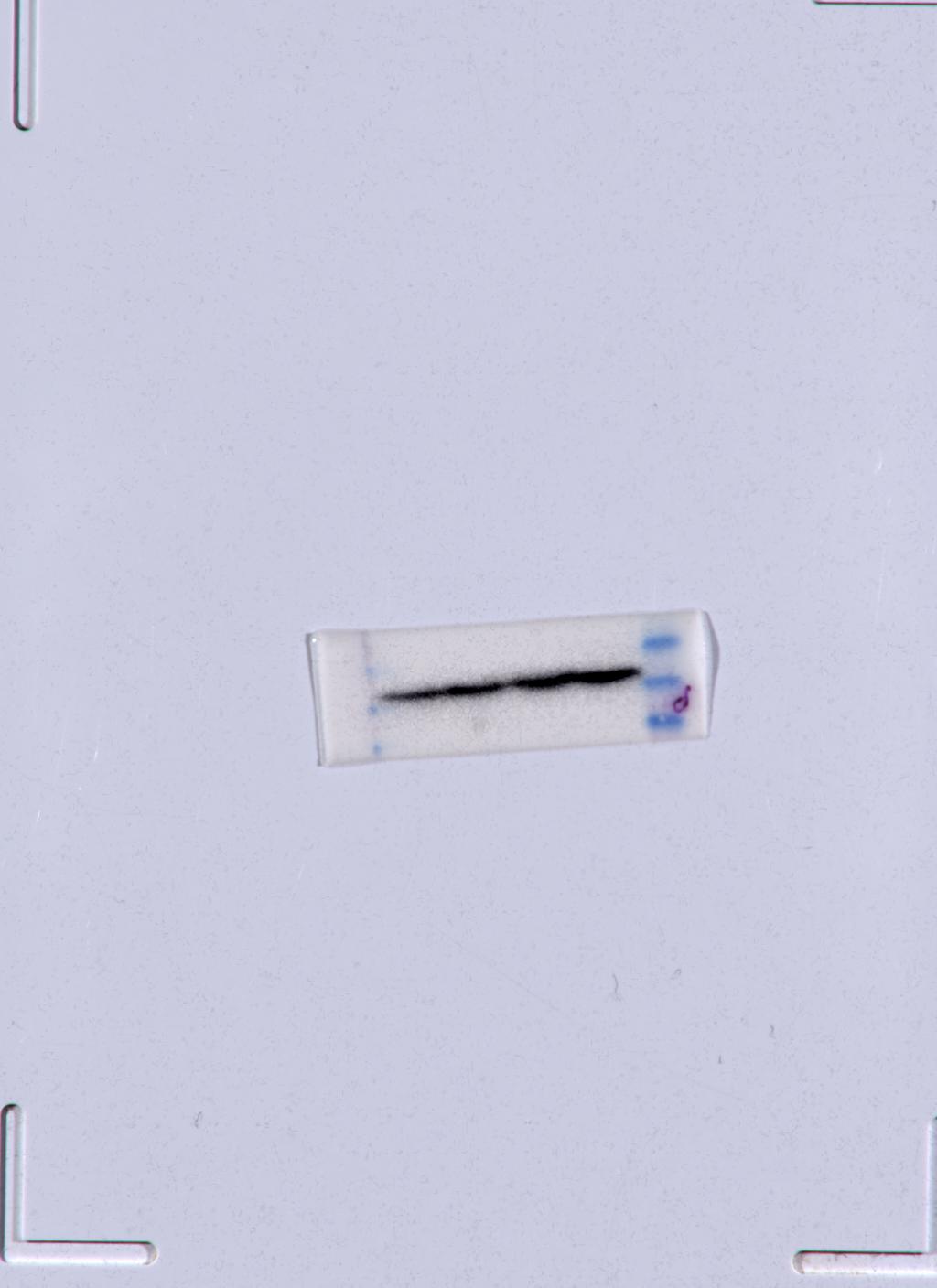

Supplement: Supplemental Information 2 [file peerj-13-19085-s002.zip › Chaetoglobosin A induces T-24 apoptosis in human bladder cancer/7.PI3K ERK pathway/p38/22.4.29 p38-5 2022.04.29_13.48.53_Ch+Marker.jpg]

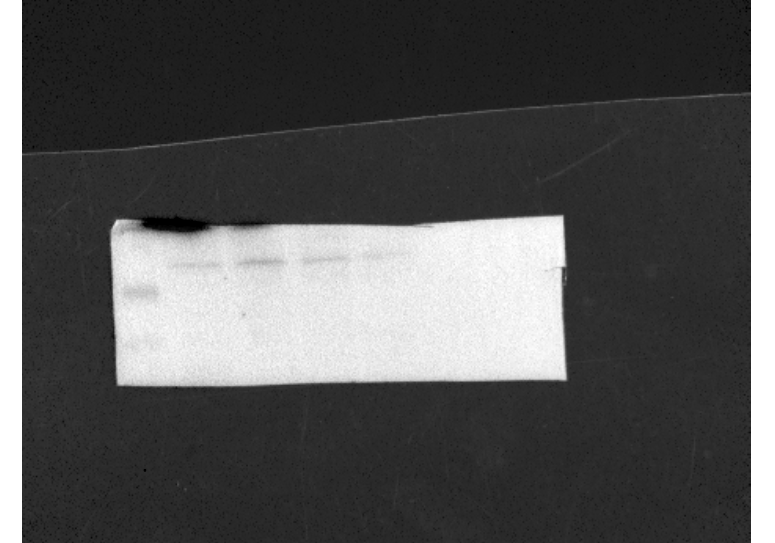

Supplement: Supplemental Information 2 [file peerj-13-19085-s002.zip › Chaetoglobosin A induces T-24 apoptosis in human bladder cancer/7.PI3K ERK pathway/PI3K/HP 2022-10-14_11h20m32s+HP 2022-10-14_11h17m54s_Exposure_120.0sec.tif]

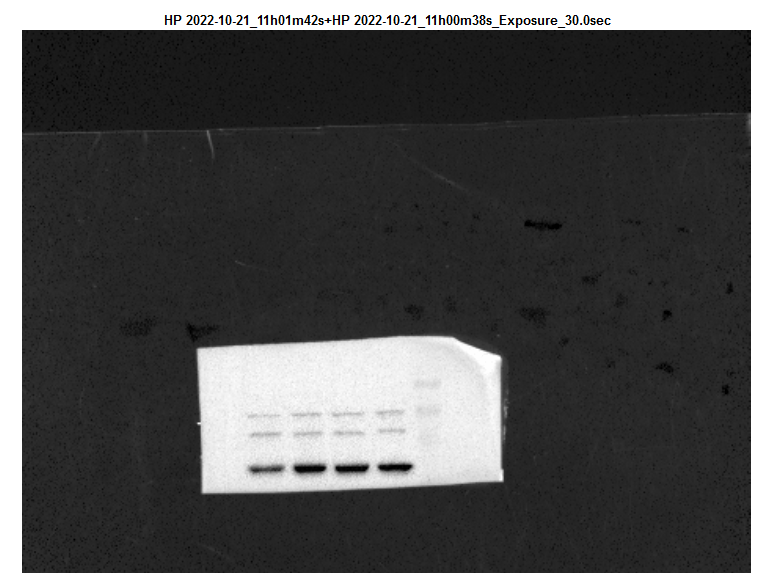

Supplement: Supplemental Information 2 [file peerj-13-19085-s002.zip › Chaetoglobosin A induces T-24 apoptosis in human bladder cancer/7.PI3K ERK pathway/PI3K/HP 2022-10-21_11h01m42s+HP 2022-10-21_11h00m38s_Exposure_30.0sec.tif]

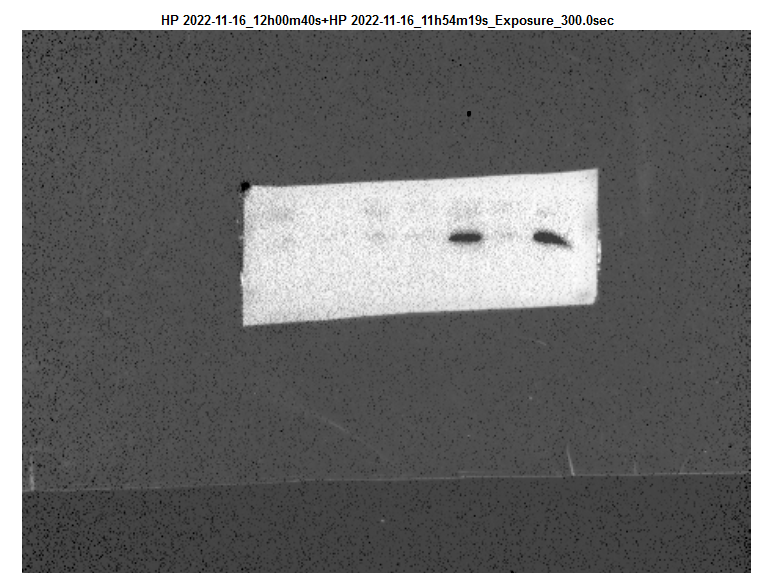

Supplement: Supplemental Information 2 [file peerj-13-19085-s002.zip › Chaetoglobosin A induces T-24 apoptosis in human bladder cancer/8.Inhibitor/Bcl-2/HP 2022-11-16_12h00m40s+HP 2022-11-16_11h54m19s_Exposure_300.0sec.tif]

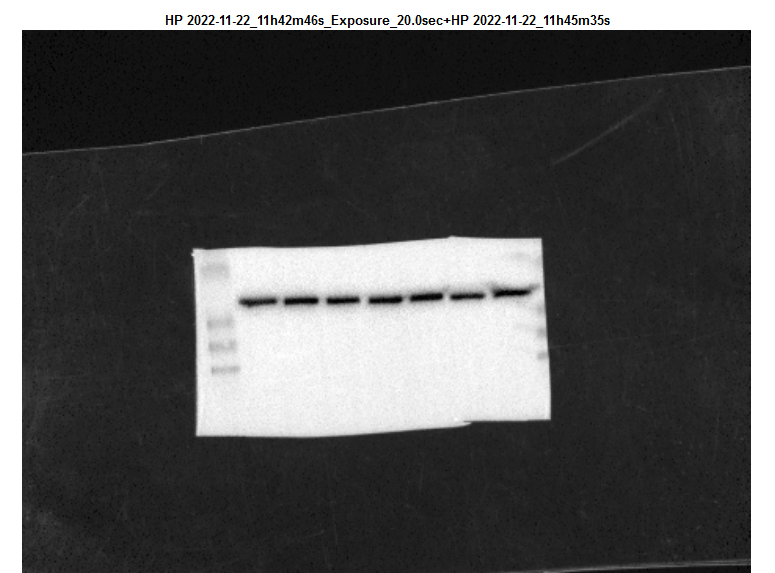

Supplement: Supplemental Information 2 [file peerj-13-19085-s002.zip › Chaetoglobosin A induces T-24 apoptosis in human bladder cancer/8.Inhibitor/Calnexin/HP 2022-11-22_11h42m46s_Exposure_20.0sec+HP 2022-11-22_11h45m35s.tif]

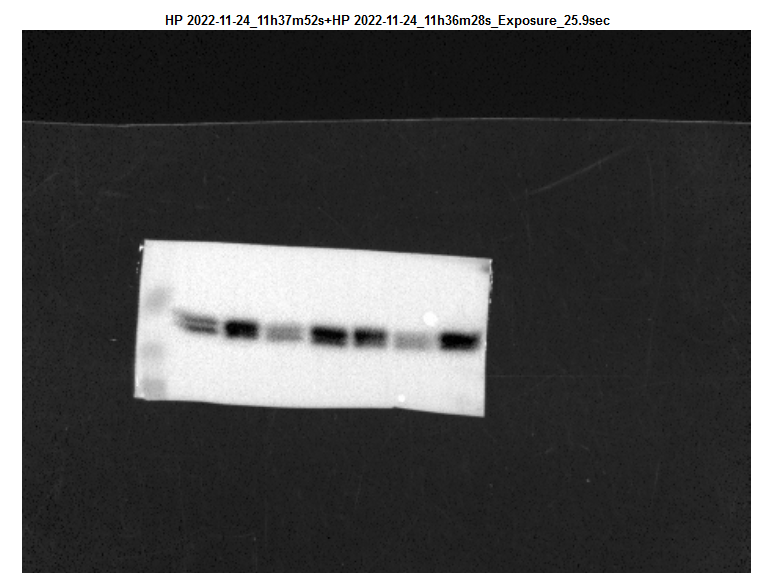

Supplement: Supplemental Information 2 [file peerj-13-19085-s002.zip › Chaetoglobosin A induces T-24 apoptosis in human bladder cancer/8.Inhibitor/ERK/HP 2022-11-24_11h37m52s+HP 2022-11-24_11h36m28s_Exposure_25.9sec.tif]

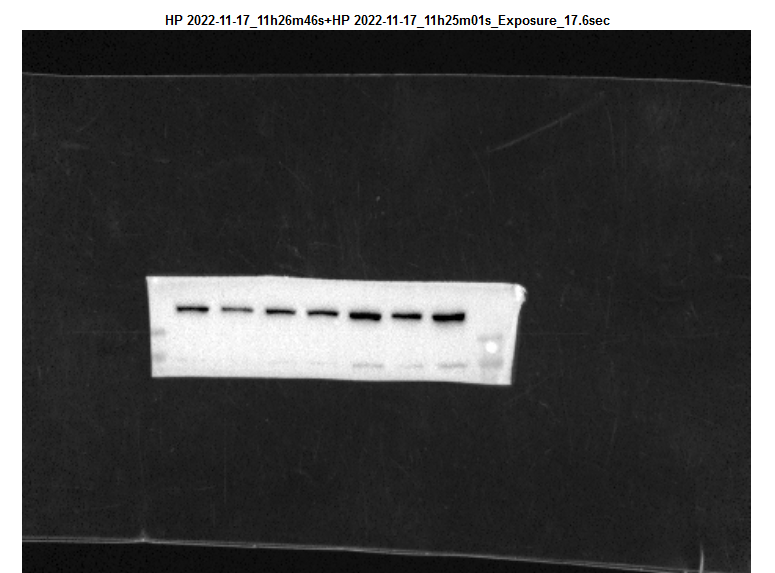

Supplement: Supplemental Information 2 [file peerj-13-19085-s002.zip › Chaetoglobosin A induces T-24 apoptosis in human bladder cancer/8.Inhibitor/mtor/HP 2022-11-17_11h26m46s+HP 2022-11-17_11h25m01s_Exposure_17.6sec.tif]

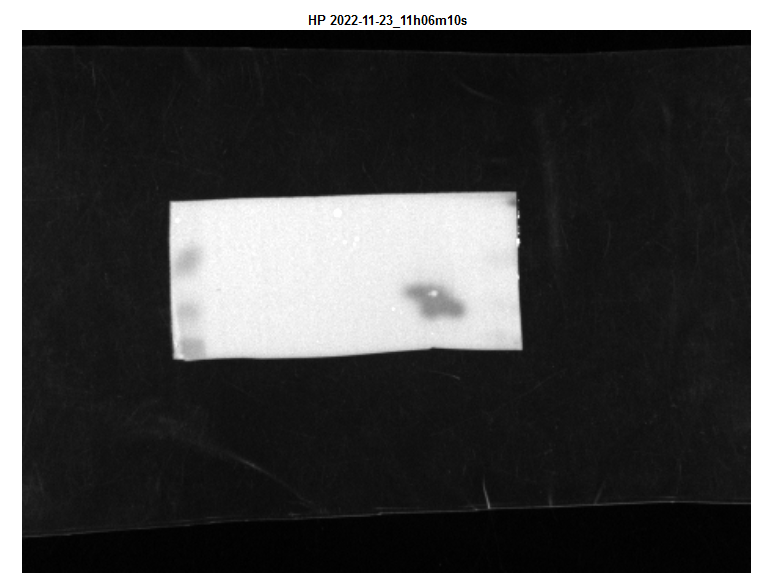

Supplement: Supplemental Information 2 [file peerj-13-19085-s002.zip › Chaetoglobosin A induces T-24 apoptosis in human bladder cancer/8.Inhibitor/p-ERK/HP 2022-11-23_11h06m10s.tif]

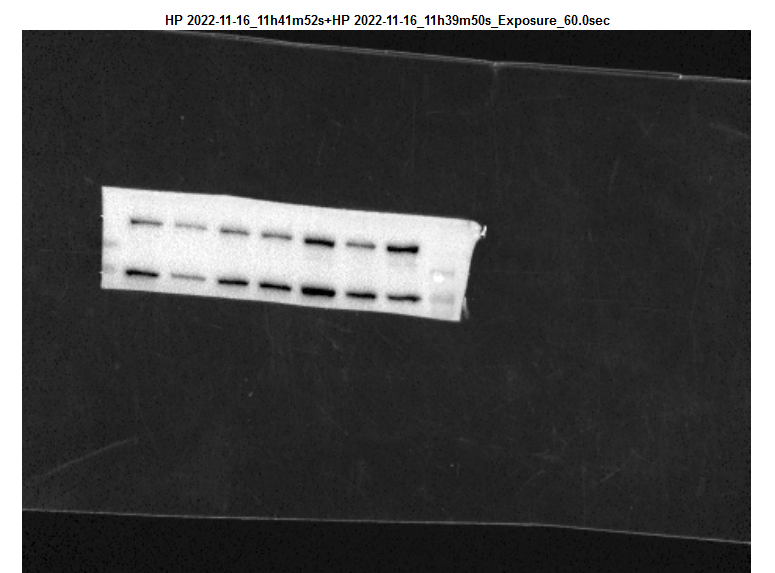

Supplement: Supplemental Information 2 [file peerj-13-19085-s002.zip › Chaetoglobosin A induces T-24 apoptosis in human bladder cancer/8.Inhibitor/p-mTOR/HP 2022-11-16_11h41m52s+HP 2022-11-16_11h39m50s_Exposure_60.0sec.tif]

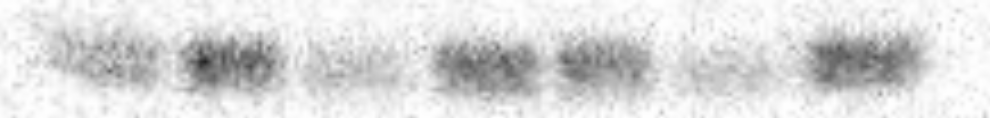

Supplement: Supplemental Information 2 [file peerj-13-19085-s002.zip › Chaetoglobosin A induces T-24 apoptosis in human bladder cancer/8.Inhibitor/p-P38/HP 2022-11-24_11h36m28s_Exposure_1.0sec.tif]

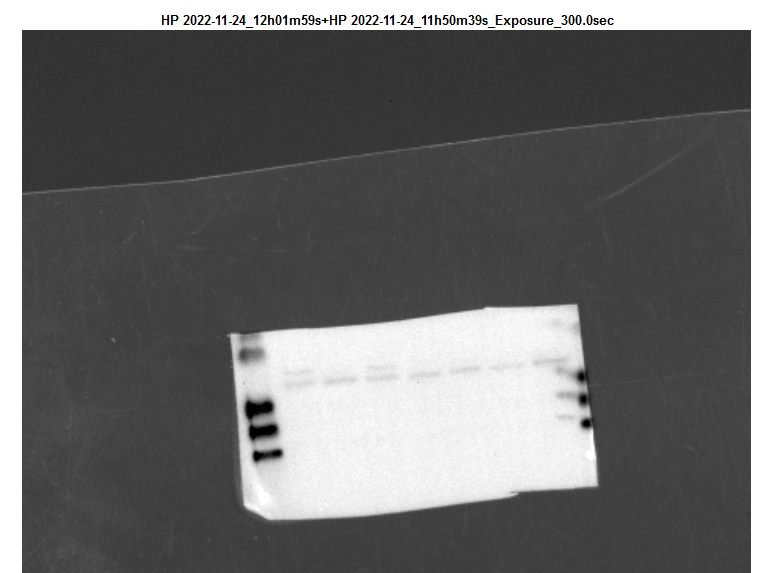

Supplement: Supplemental Information 2 [file peerj-13-19085-s002.zip › Chaetoglobosin A induces T-24 apoptosis in human bladder cancer/8.Inhibitor/p-PI3K/HP 2022-11-24_12h01m59s+HP 2022-11-24_11h50m39s_Exposure_300.0sec.tif]

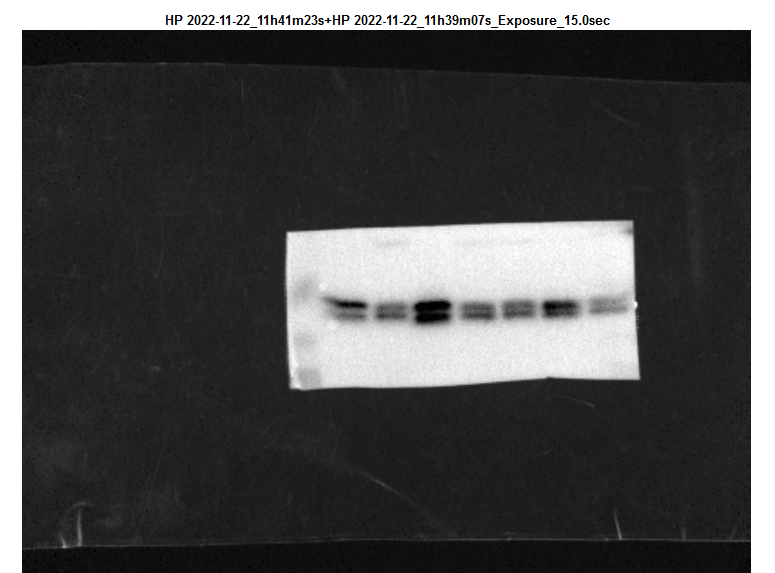

Supplement: Supplemental Information 2 [file peerj-13-19085-s002.zip › Chaetoglobosin A induces T-24 apoptosis in human bladder cancer/8.Inhibitor/P38/HP 2022-11-22_11h41m23s+HP 2022-11-22_11h39m07s_Exposure_15.0sec.tif]

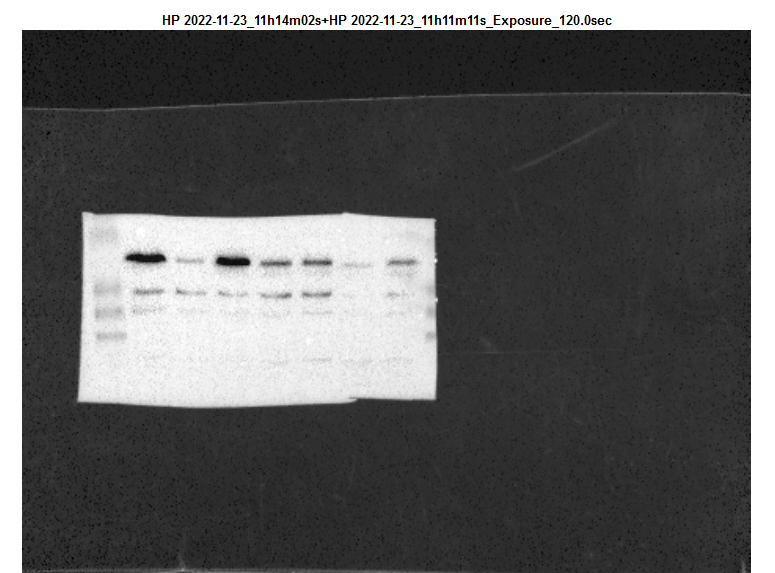

Supplement: Supplemental Information 2 [file peerj-13-19085-s002.zip › Chaetoglobosin A induces T-24 apoptosis in human bladder cancer/8.Inhibitor/PI3K/HP 2022-11-23_11h14m02s+HP 2022-11-23_11h11m11s_Exposure_120.0sec.tif]
